# Multiple Oxygen Tension Environments Reveal Diverse Patterns of Transcriptional Regulation in Primary Astrocytes

**DOI:** 10.1371/journal.pone.0021638

**Published:** 2011-06-27

**Authors:** Wayne Chadwick, John P. Boyle, Yu Zhou, Liyun Wang, Sung-Soo Park, Bronwen Martin, Rui Wang, Kevin G. Becker, William H. Wood, Yongqing Zhang, Chris Peers, Stuart Maudsley

**Affiliations:** 1 Receptor Pharmacology Unit, National Institute on Aging, National Institutes of Health, Baltimore, Maryland, United States of America; 2 Institute for Cardiovascular Research, Leeds Institute of Genetics, Health and Therapeutics, University of Leeds, Leeds, West Yorkshire, United Kingdom; 3 Metabolism Unit, National Institute on Aging, National Institutes of Health, Baltimore, Maryland, United States of America; 4 Gene Expression and Genomics Unit, Research Resources Branch, National Institute on Aging, National Institutes of Health, Baltimore, Maryland, United States of America; Universidade Federal do Rio de Janeiro, Brazil

## Abstract

The central nervous system normally functions at O_2_ levels which would be regarded as hypoxic by most other tissues. However, most in vitro studies of neurons and astrocytes are conducted under hyperoxic conditions without consideration of O_2_-dependent cellular adaptation. We analyzed the reactivity of astrocytes to 1, 4 and 9% O_2_ tensions compared to the cell culture standard of 20% O_2_, to investigate their ability to sense and translate this O_2_ information to transcriptional activity. Variance of ambient O_2_ tension for rat astrocytes resulted in profound changes in ribosomal activity, cytoskeletal and energy-regulatory mechanisms and cytokine-related signaling. Clustering of transcriptional regulation patterns revealed four distinct response pattern groups that directionally pivoted around the 4% O_2_ tension, or demonstrated coherent ascending/decreasing gene expression patterns in response to diverse oxygen tensions. Immune response and cell cycle/cancer-related signaling pathway transcriptomic subsets were significantly activated with increasing hypoxia, whilst hemostatic and cardiovascular signaling mechanisms were attenuated with increasing hypoxia. Our data indicate that variant O_2_ tensions induce specific and physiologically-focused transcript regulation patterns that may underpin important physiological mechanisms that connect higher neurological activity to astrocytic function and ambient oxygen environments. These strongly defined patterns demonstrate a strong bias for physiological transcript programs to pivot around the 4% O_2_ tension, while uni-modal programs that do not, appear more related to pathological actions. The functional interaction of these transcriptional ‘programs’ may serve to regulate the dynamic vascular responsivity of the central nervous system during periods of stress or heightened activity.

## Introduction

Normal brain function has an absolute requirement for a continuous supply of O_2_, yet it is considered particularly susceptible to oxidative damage. This has been postulated to arise because of the high levels of central nervous system (CNS) O_2_ consumption, which is employed to generate ATP primarily through oxidative phosphorylation [1]. This profound energy dependence requires high levels of mitochondrial activity which, along with the presence of excitotoxic and oxidizable neurotransmitters and large, transient Ca^2+^ fluxes, contribute to a high degree of potential oxidative stress. Such stress is imposed not only on neurons but also on glia, and although both neurons and glia have antioxidant defense mechanisms [2], the simplest approach to avoiding oxidative stress is to keep brain O_2_ levels as low as possible without compromising oxidative phosphorylation. Astrocytes are of particular importance in this regard, as they are now known to regulate local blood supply to match local neuronal activity with remarkable speed and precision [3], [4]. Thus, they are of major importance in the control of cerebral blood flow and, hence, brain O_2_ levels.

The strategy of maintaining brain O_2_ at low but sufficient levels results in reported pO_2_ values ranging from *ca.* 20–30 mmHg despite arterial pO_2_ levels being *ca.* 90 mmHg. Indeed, some estimates have suggested that some 50% of brain regions normally exist at a pO_2_ of below 10 mmHg [1], [5]. This value therefore represents normoxia for neurons and glia, yet would be considered hypoxic by most other tissues. The brain itself can experience relative hypoxia either at altitude, or as a consequence of cardiorespiratory disorders which result in perturbed O_2_ collection in the lungs or distribution by the vasculature, *e.g.* sleep apnea. If such hypoxia persists, this can predispose individuals to CNS damage, and markedly increases the likelihood of developing progressive dementias such as Alzheimer's disease (AD) [6]. Indeed, we and others have previously shown that hypoxia *in vitro* leads to increased production of amyloid peptides (Aβ) associated with AD [6], .

Local O_2_ levels, either hyperoxic or hypoxic, are now recognized as a major determinant of gene expression in all tissues examined to date. Numerous transcription factors (*e.g.* hypoxia inducible factor (HIF) and nuclear factor kappa B (NF-κB)) are known to be activated in hypoxia, and control the expression of large numbers of genes [9]. Such altered gene transcription and expression is of fundamental importance in the development of multiple tissue disorders such as cancers and dementias [10]. Given the importance of the relative abundance, or paucity, of O_2_ to such activity, and the uniquely low levels of O_2_ which can be regarded as normoxic in the brain, it is perhaps surprising that no systematic, unbiased, study of physiologically relevant levels of O_2_ on gene transcription in primary cortical astrocytic tissue has been previously conducted. We have shown previously that the relative abundance of oxygen (and oxygen-derived reactive species) can exert significant effects upon CNS pathological protein metabolism [6]–[8], as well as strongly regulate the pharmacogenomic ‘signatures’ of transmembrane receptor signaling systems [11], [12]. As the relative oxygen levels in the CNS are likely to demonstrate a high dynamic flux, an understanding of the responsiveness at the cellular level to this, may assist in the development of CNS-targeted anti-neurodegenerative therapeutic strategies. Understanding how the CNS system reacts and functions at diverse oxygen tensions may also benefit the rational development of drug-like agents with specific ‘context-sensitive’ efficacies and potencies [11], [13].

Here, we report the effects of various O_2_ levels on gene transcription and protein expression profiles in cortical astrocytes, employing levels of O_2_ which can be regarded as physiologically normoxic, hypoxic and, importantly hyperoxic, a condition which is commonly imposed on these and other primary cultured cells when typically studied *in vitro*. Our data indicates that clearly defined, specific ‘morphometric’ response patterns to varying O_2_ tensions exist, that bear distinct and functionally relevant phenotypes to CNS health. An enhanced appreciation of the alterations in the CNS environment during altered O_2_ tension will likely assist the development of novel therapies that may demonstrate specific efficacy in separate areas of the brain experiencing divergent levels of perfusion.

## Materials and Methods

### Primary astrocyte cultures

Primary cultures of rat cortical astrocytes were obtained as previously described [14]. All animal care and experimental procedures, performed under code (A(SP)A-86), followed United Kingdom Home Office Animals Scientific Procedures guidelines. Animal care and experimental procedures followed United Kingdom Home Office regulations (code A(SP)A-86) and were conducted under the project licence PPL 40/3356 held by Professor C. S. Peers, following the official United Kingdom Home Office Animals Scientific Procedures guidelines. In brief, cerebral cortices were removed from 6–8-day-old Wistar rats and placed in ice-cold phosphate-buffered solution (PBS) containing: 8 mM NaH_2_PO_4_, 2.7 mM KCl, 138 mM NaCl, and 2.7 mM KH_2_PO_4_. Multiply-dissected cortices were dispersed into the same buffer containing 0.25 mg/mL trypsin, at 37°C for 15 min. Digestion was halted by the addition of an equal volume of buffer supplemented with 16 µg/mL soy bean trypsin inhibitor (type I-S; Sigma, Poole, Dorset, UK), 20 U/mL DNase I (EC 3.1.21.1 type II from bovine pancreas; Sigma) and 1.6 mM MgSO_4_. The tissue was then pelleted by centrifugation at 1000×*g* for 1 min and the supernatant was poured off before resuspending the cell pellet in 6.8 mL of buffer solution containing 100 µg/mL soy bean trypsin inhibitor, 125 U/mL DNase I and 10 mM MgSO_4_. The tissue was subsequently triturated and, after allowing larger pieces of tissue to settle, the cell suspension was pipetted into media (Eagle's minimal essential medium supplemented with 10% fetal calf serum (v/v) and 1% (v/v) penicillin-streptomycin (Gibco, Paisley, UK)). The cell suspension was then aliquoted into 75 cm^2^ flasks. Cells were then maintained in a humidified incubator at 37°C (95% air; 5% CO_2_). Four to six hours following plating, cells were washed twice with fresh media to remove non-adherent cells. This resulted in a culture of cortical astrocytes, as confirmed by visual inspection the following day and later by glial fibrillary acidic protein immunohistochemistry (data not shown). Any cortical astrocyte culture that was not homogenous was disposed of and not used in this study. Culture medium was exchanged every 7 days and cells were grown in culture for up to 14 days. In some cases, cells were exposed to chronic hypoxia; 24 hr prior to experimentation cells were transferred to a humidified incubator or hypoxic workstation both equilibrated with 1, 4 or 9% O_2_, 5% CO_2_, and the remaining percentage gas N_2_. Control cells were maintained in a 95% air, 5% CO_2_ incubator for the same time period.

Once cortical astrocytes had reached approximately 90% confluence (75 cm^2^ flask) they were subjected to hypoxia or normoxia, as above, washed with PBS, removed from the flask base with 0.05% trypsin-EDTA (Gibco) and then gently centrifuged (500×g). The cell pellet was then re-suspended in PBS and centrifuged twice more to remove any traces of media. The cell pellet was then: a) if required for RNA analysis; triturated in 8–10 volumes of RNA*later* (Applied Biosystems), frozen and stored at −80°C until analysis; b) if required for the analysis of proteins; re-suspended in either 10–15 volumes of M-PER reagent (Pierce-Perbio, UK) supplemented with a Complete mini protease inhibitor tablet (Roche Applied Science) or an equivalent volume of a chaotropic solution (7 M Urea, 4% CHAPS, 30 mM Tris at pH 8.5). These samples were frozen and stored at −80°C until analysis.

### RNA extraction

RNA isolation was carried out using the Qiagen RNeasy Mini Kit for cell extraction (Qiagen, Inc. Valencia CA). The cells were lysed in the proprietary buffer and then centrifuged. The supernatant was transferred to a second tube and centrifuged again to clear any remaining cellular debris. The supernatant was added to 95% ethanol, mixed and added to the proprietary binding columns. The columns were centrifuged, washed several times and the bound RNA was eluted using water. The RNA quality and quantity was checked using an Agilent 2100 Bio-analyzer and the RNA 6000 nano-chips. As an index of RNA quality we assessed the mean 28S/18S ribosomal RNA values for the samples. The ideal ratio of 28S/18S for intact RNA is 2.0, our measured 28S/18S ratio was 1.98±0.06 (mean ± standard error of mean).

### Microarray hybridization and analysis

Total RNA was used to generate biotin labeled cRNA using the Illumina TotalPrep RNA Amplification Kit (Ambion; Austin, TX, cat #IL1791). In brief, 0.5 µg of total RNA was first converted into single-stranded cDNA with reverse transcriptase using an oligo-dT primer containing the T7 RNA polymerase promoter site and then copied to produce double-stranded cDNA molecules. The double stranded cDNA was cleaned and concentrated with the supplied columns and used in an overnight *in vitro* transcription reaction where single-stranded RNA (cRNA) was generated and labeled by incorporation of biotin-16-UTP. A total of 0.75 µg of biotin-labeled cRNA was hybridized at 58°C for 16 h to Illumina's Sentrix MouseRef-8 Expression BeadChips (Illumina, San Diego, CA). Each BeadChip has 24,000 well-annotated RefSeq transcripts with approximately 30-fold redundancy. The arrays were washed, blocked and the labeled cRNA was detected by staining with streptavidin-Cy3. The arrays were scanned using an Illumina BeadStation 500× Genetic Analysis Systems scanner and the image data extracted using the Illumina BeadStudio software, Version 3.0.

### Microarray data analysis

Microarray data were analyzed using DIANE 6.0, a spreadsheet-based microarray analysis program based on SAS JMP7.0 system. Raw microarray data were subjected to filtering and normalization and tested for significant changes as described previously [15]. Raw hybridization intensity data were log-transformed and normalized to yield z-scores, which in turn were used to calculate a z-ratio value for each gene transcript with respect to the control tissues. The z-ratio was calculated as the difference between the observed gene z-scores for the experimental and the control comparisons, and dividing by the standard deviation associated with the distribution of these differences [15]. Z-ratio values ≥+1.5 or ≤−1.5 were chosen as cut-off values, defining increased and decreased expression, respectively. Array data were analyzed using DIANE 6.0, a spreadsheet-based microarray analysis program based on the SAS JMP7.0 system. Raw microarray data were subjected to filtering and *Z* normalization and tested for significant changes as described previously [15]. Briefly, sample quality was analyzed by scatter plot followed by gene filtering as follows. A false discovery rate (FDR) cut-off <0.01 was applied, which controls for the expected proportion of falsely rejected hypotheses. Subsequent remaining genomic data, possessing a z-ratio of ≥1.5 (±), were further analyzed using a two-way ANOVA design with significance set at *p*≤0.05. Significantly-regulated genes that fulfilled all these criteria were assigned a selector score of ±3 (+, upregulated: −, downregulated) to indicate that they possess a z-ratio of ≥1.5(±), FDR of ≤0.01 and an ANOVA *p*≤0.05. Array data for each experimental oxygen tension condition was additionally subjected to hierarchically *k-means* clustering in DIANE 6.0/Ilumina BeadStudio Version 1.5 to investigate the presence of oxygen tension-related transitional transcriptomic patterns. We have deposited the raw data at GEO/ArrayExpress under accession number GSE29296, we can confirm all details are MIAME compliant.

### Bioinformatic geneset analysis

After identifying individual genes that were significantly regulated by different oxygen tensions, the gene lists were analyzed further using multiple forms of functional annotational clustering, *i.e.* principal component analysis and *k-means* clustering (using DIANE 6.0/JMP7.0), parametric geneset enrichment analysis (PAGE) [16] using the Broad Institute Molecular Signatures Database (MSigDB: http://www.broadinstitute.org/gsea/msigdb/) canonical signaling pathway (CanPath) or functional network prediction analysis using Ingenuity Pathway Analysis (IPA: http://www.ingenuity.com/). For PAGE/CanPath analyses similar genomic statistical criteria were employed to ensure significant CanPath and PAGE gene collection ‘population’. For each form of grouping, at least two genes were required to fill the requisite group/pathway at a significance of at least p<0.05. For CanPath functional grouping, a single amenable numeric index was created (hybrid score) by multiplying the relative enrichment (to a species-specific background set) factor (R) with the negative log_10_ of the probability of enrichment. For MSigDB annotation a cumulate Z score (positive or negative) for a specific PAGE gene collection was calculated using the sum of the z ratios of the individual genes clustering significantly into that PAGE collection. In addition to parametric geneset enrichment and IPA CanPath/functional network analysis, we also performed latent semantic indexing (LSI) textual analysis using GeneIndexer (https://computablegenomix.com/geneindexer/) as described previously [11],[12],[17].

### Western blotting

Whole cell astrocyte lysates were created using M-PER protein extraction reagent (Pierce-Thermo Scientific, Rockford IL) or an in-house developed NP-40-based lysis buffer [18]. Protein extract concentrations were measured using a standard BCA assay kit (Pierce-Thermo Scientific, Rockford IL) and then normalized to 1 mg/mL of total protein using the appropriate extraction buffer. Protein extracts (20 µg total per lane) were then resolved on Bis-Tris 4–12% gradient polyacrylamide gels (Invitrogen, Carlsbad CA) before electrotransfer to polyvinylenediflouride (PVDF) membranes (Perkin Elmer Life, Waltham, MA). PVDF membranes were then blocked with Blotto (ThermoFisher Scientific, Waltham MA) for one hour before incubation with specific primary antisera at 1∶1000 dilutions followed by five washing cycles in Tris (10 mM)-buffered saline and then incubation with a 1∶10,000 dilution of a species-specific alkaline-phosphatase conjugated secondary antisera (Sigma Aldrich, St. Louis, MO). Presence of immunoreactive bands was detected by a one minute incubation with an enzyme-linked chemifluorescent developing reagent (GE Healthcare, Piscataway, NJ) followed by scanning with a Typhoon 9410 variable-mode phosphorimager (GE Healthcare, Piscataway, NJ). Antisera for the specific proteins identified were obtained as follows: anti-*c-fos* was from Sigma Aldrich (St. Louis, MO); anti-*Flot1* (flotilin-1) was from BD Bioscience (Chicago, IL); anti-*Cst-3* (cystatin-C) was from LSBio (Seattle, WA); anti-*Gclc* (glutamate-cysteine ligase, catalytic subunit), anti-*Gclm* (glutamate-cysteine ligase, modifier subunit), anti-*Txnip* (thioredoxin interacting protein) anti-*Timp1* (TIMP metallopeptidase inhibitor 1), anti-*Adm* (adrenomedullin), anti-*Chrdl1* (chordin-like 1), anti-*Ucp2* (uncoupling protein-2), anti-*Zmpste24* (zinc metalloprotease similar to yeast Ste24p), anti-*Dcn* (decorin), anti-*Adk* (adenosine kinase) were from Abcam (Cambridge, MA); anti-*Vps34*/*Pik3c3* (phosphoinositide-3-kinase, class III) was obtained from Novus Biologicals (Littleton, CO); anti-*Cxcl12*/*Sdf1-α* (stromal cell-derived factors 1-alpha) was obtained from Abnova (Walnut, CA); anti-*Calr* (calreticulin), anti-*Gsk3-β* (glycogen synthase kinase beta), anti-*Sirt2* (silent mating type information 2-homolog), anti-*Vim* (vimentin) and anti-*Calm* (calmodulin) were from Cell Signaling Technology (Danvers, MA).

## Results

### Principal component analysis and global gene regulation

**Figure 1:**
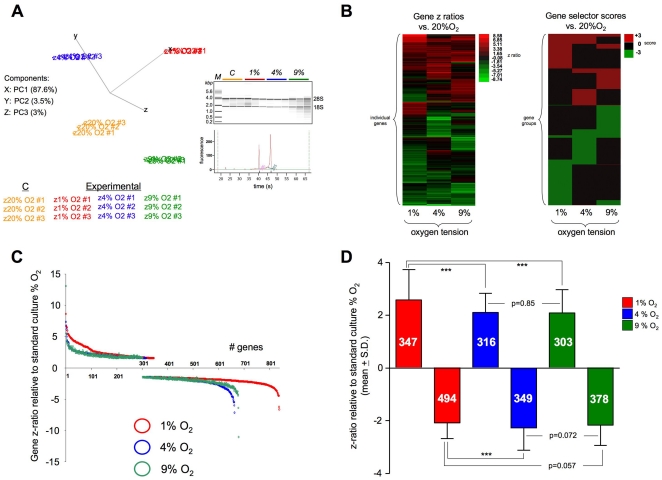
Primary transcriptional oxygen tension-dependent responses in astrocytes. (**A**) Principal component analysis of triplicate transcriptomic datasets generated from primary astrocytes exposed to 1 (z 1% O_2_ # 1, 2, 3), 4 (z 4% O_2_ # 1, 2, 3), 9 (z 9% O_2_ # 1, 2, 3) or 20% (z 20% O_2_ # 1, 2, 3) ambient oxygen conditions for 24 hours. Inset panel presents an RNA electropherogram of isolated RNA from the various samples (kilo-basepair (kbp) mass – M; control – C; 1% O_2_ – 1%; 4% O_2_ – 4%; 9% O_2_ – 9%) as well as a representative 28S(green)/18S(pink) fluorescence quantification trace employed for RNA quality control. (**B**) Left panel, individual gene transcriptional z ratios (with associated heatmap key) and gene selector scores (based on criteria mentioned in Methods: right panel) for gene array datasets from astrocytes experiencing 1, 4, 9% O_2_ conditions relative to the expression level at 20% O_2_. Gene selector scores of ±3 indicate compliance with stringent limitations of z ratio, FDR and probability. (**C**) z ratio expression profile of significantly regulated transcripts in astrocytes exposed to 1% (red circles), 4% (blue) or 9% (green) ambient O_2_ conditions. (**D**) Mean ± standard deviations of z ratios for significantly, up- or down-regulated, genes after exposure to 1, 4, or 9% O_2_ conditions. The specific numbers of up or downregulated genes are indicated in white in each histogram bar. Statistical analysis, Student's t-test, was performed using GraphPad Prism version 5.02 (GraphPad Software Inc.). *p*≤0.05 = *, *p*≤0.01 = **, *p*≤0.001 = ***. Non-significant differences are indicated by numerical p values.

At the most basic analytical level, it was evident from the primary genomic principal component analysis (PCA) that the transcriptomic profile of primary rat astrocytes is highly distinct at differing culturing oxygen tensions (Figure 1A). Each of the transcription profiles occupied a close-knit and discrete grouping in three dimensional PCA space. The PCA component breakdown into the respective X, Y and Z vectors is depicted in the inset panel in Figure 1A. The inset panel in Figure 1A indicates the quality of extracted RNA (18S, 28S RNA) across samples. A representative quantitation of the 28S/18S ratio is included in the inset. The individual, significantly regulated, gene lists for each experimental oxygen tension, compared to the standard control condition (atmospheric 20% O_2_), are listed in Tables S1 (1% O_2_), S2 (4% O_2_) and S3 (9% O_2_). Employing a z ratio cut-off (±1.5 compared to the mean) the z ratios of the genes significantly altered (relative to 20% O_2_ control) by the different oxygen tensions are depicted in the heatmap in Figure 1B. In addition to the z ratio heatmap, using the three-way significant criteria described in Materials and Methods a selector score for all the genes that satisfy this criteria is indicated (upregulated +3, downregulated −3). All of the experimental oxygen tensions (1, 4, 9% O_2_) caused the significant alteration of hundreds of genes to generally similar z-ratio extents, ranging from a 11-fold (logarithmic) increase to a similar level of decrease. The highest number of significantly altered genes was noted in response to the most extreme level of oxygen tension (1% O_2_) reduction compared to control (Figure 1C). Upon inspection of the relative z ratio extent of gene regulation, each of the experimental oxygen tensions induced a similar mean up or downregulation of genes (Figure 1D), however the 1% oxygen tension did generate the greatest mean elevation in gene transcription (1% O_2_-2.58, 4% O_2_-2.11, 9% O_2_-2.09). In each ambient oxygen tension paradigm however, it was noted that a greater number of genes were significantly downregulated, compared to those upregulated when compared to control (20% O_2_) conditions.

### Basic oxygen tension-dependent transcriptomic analysis

With respect to specific gene regulation induced by switching to 1% O_2_ tension from the cell culture-standard 20% O_2_ tension, we identified multiple highly upregulated genes that have previously been linked to hypoxia, *e.g. Vegfa* [19], *hypoxia-induced gene 1* [20], *hexokinase* [21] and *Insig1* [22] (Table S1). It was evident that many of the genes highly upregulated by the 1% O_2_ environment were directed to ribosomal activity (*e.g. Rpl10*, *Rpl41*, *Rps10*, *Rps18*, *Rpl29*, *Rps27*), heat shock factor expression (*Hspa8*, *Hspb1*, *Hspca*) and energy regulation/metabolism (*Pgk1*, *Tpi1*, *Pdk1*, *Pygl*). In addition to these transcripts, multiple iron-management factors were also highly upregulated, *e.g.* ferritin light chain 1 and 2 (*Ftl1*, *Ftl2*) and ferritin heavy polypeptide (*Fth1*). One of the most highly downregulated factors was gap junction membrane channel protein beta 2 (*Gjb2*), potentially suggesting a link to alterations in blood flow mechanisms [23].

Inspecting the gene regulation at the 4% O_2_ tension compared to the 20% O_2_ tension (Table S2), typical hypoxia-regulated factors such as carbonic anhydrase 3, lipocalin-2 [24] and prostacyclin synthase [25] were all strongly upregulated (Table S2). As with the 1% O_2_ tension geneset, there was a strong ribosomally-directed component, as well as a profound heat shock/chaperone-directed component. Among the relatively novel genes demonstrated to be responsive to this O_2_ level, we noted a significant transcriptional potentiation of jagged 1 (*Jag1*) [26] and pre-proenkephalin (*Penk-rs*) [27]. Significant downregulation of several transcripts linked to functional hypoxia was also observed, *e.g.* aurora kinase B [28] and uncoupling protein 2 [29]. In addition, the lipid raft associated protein, flotilin-1, was also noted to be down regulated at the 4% O_2_ level relative to 20% O_2_. At the 9% O_2_ tension, markers of functional hypoxia were again evident, *i.e.* carbonic anhydrase 3, prostacyclin synthase and *Hig-1* (Table S3). In addition to these factors, we also demonstrated a profound upregulation of the WNT1 inducible signaling pathway protein 2 (*Wisp2*) [30] and basigin (*Bsg*) [31]. With regards to the downregulated factors at the 9% O_2_ tension level, we noted a strong chronological timing component, as *Per1*, *Per2* and *Cry1* were all significantly downregulated. The heatshock/immune function gene, *Schlafen-3* was also significantly downregulated.

### Multiple oxygen tension-dependent transcriptional activity relationships

**Figure 2:**
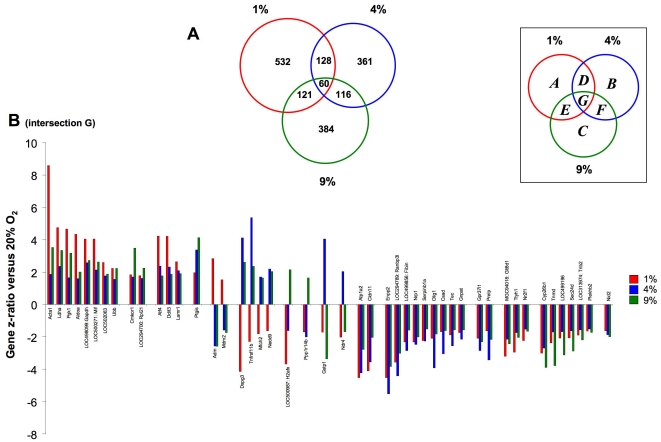
Venn diagram analysis of oxygen tension-dependent transcriptional responses in primary astrocytes. (**A**) Three-way proportional Venn diagram for transcriptional responses (compared to ambient 20% O_2_) for 24 hour exposure to 1% (red circle), 4% (blue circle) and 9% (green circle) ambient oxygen tension. The intersection subsets (A–G) are specified in the associated boxed key. (**B**) Oxygen tension-dependent variations in transcriptional regulation of multi-tension responsive genes from intersection G (1% O_2_, red; 4% O_2_, blue; 9% O_2_, green).

We have demonstrated that the O_2_ responsive genesets, at our three experimental tensions, indicate significant changes in both classical and novel oxygen-sensitive factors in the primary cortical astrocytes. We next investigated the relationships between the identities of the genes uniquely regulated, or regulated by more than one oxygen tension using Venn diagram analysis. The resultant proportional Venn diagram for the gene set intersections between the specific oxygen tension-regulated dataset is depicted in Figure 2A. The gene identities in the respective unique (A, B, C) and intersected subsets (D, E, F, G) are listed in Table S4. Using this simple Venn diagram separation, it was evident that for each experimental oxygen tension, a relatively unique overall transcriptomic phenotype existed (Figure 2A). For example, at each specific experimental oxygen tension the majority of the significantly regulated transcripts were unique to that oxygen tension, *i.e.* 63.2%, 54.2% and 56.4% of the transcripts were uniquely regulated at 1%, 4% or 9% O_2_ respectively (Figure 2A, sets A, B, C according to associated key). This primarily unique oxygen tension response reinforces the highly distinguished PCA clustering observed in Figure 1A. The number of shared (between at least two individual tensions) significantly-regulated transcripts between the three experimental oxygen tensions were relatively similar (Figure 2A). The smallest intersection set was the group of transcripts common to all the experimental oxygen conditions (Figure 2A).

With respect to the analyses of the genes compartmentalized into the respective intersections, we rationalized the subsets into the following hypothetical groups. Specific subsets of transcripts may regulate astrocyte responsiveness to broad-range dynamic changes in ambient oxygen tensions: ‘*multi-tension*’ responsive genes (Figure 2A, intersection G: altered in response to 1%, 4% and 9% O_2_ relative to 20% O_2_); ‘*low tension-responsive*’ genes (Figure 2A, Table S5, intersection D: significantly regulated in 1–4% O_2_); ‘*intermediate-tension*’ oxygen responsive (Figure 2A, Table S6, intersection F: significantly regulated in 4–9% O_2_); ‘*cross-tension*’ responsive (Figure 2A, Table S7, intersection E: significantly regulated in 1–9% O_2_). In each of the Venn intersections in Figure 2, the majority of transcripts common to two or more oxygen tensions demonstrated similar directions of regulation (up- or downregulation). The percentage of significantly regulated transcripts identified at all oxygen tensions (Figure 2A, intersection G) that demonstrated diverse polarities of regulation was only 16.7% (10 transcripts out of 60: Figure 2B). Similar percentages of transcripts, common between at least two oxygen tensions but with differing regulation polarities, were observed for the other Venn diagram intersections in Figure 2: 1–4% - 14 out of 128 (10.9%); 1–9% - 28 out of 121 (23.1%); 4–9% - 17 out of 116 (14.6%). Therefore it seemed that reversal of transcriptional regulation in astrocytes between different oxygen tensions is a relatively rare event. The large number of transcripts that are uniquely and significantly regulated at only one O_2_ tension (Figure 2A, sets A, B, C) may represent discrete functional gene groups that control steady-state cellular function during constant O_2_ tensions. Conversely, the significantly-regulated transcripts in the intersections, D, E, F and G may therefore control dynamic responses to fluctuations in ambient O_2_ tensions.

With respect to the upregulated *multi-tension* responsive group (Table S4, G: Figure 2B), notable inclusions were the chemokine orphan receptor 1 (*Cmkor1*), prostacyclin synthase (*Ptgis*), glyceraldehyde 3 phosphate dehydrogenase (*Gapdh*), laminin receptor 1 (*Lamr1*) and phosphoglycerate kinase 1 (*Pgk1*). The expression of the chemokine orphan receptor 1 (also known as CXCR7) has recently been demonstrated to be controlled by oxygen levels in the central nervous system [32]. However, ours is the first demonstration of coherent oxygen tension-mediated regulation of this transcript in rat cortical astrocytic cells. As with our previous global transcription analysis (Tables S1, S2, S3), we noted that a specifically upregulated ribosomal activity signature was present in this specific intersection (ribosomal protein L21, *Rpl21*: Figure 2B). Among this *multi-tension* group we also noted that there were multiple hypoxia-related factors that demonstrated divergent polarities of transcript regulation between 1-4-9% O_2_ tensions, *e.g.* adrenomedullin (*Adm*) [33], [34], *Mdm2* (mouse double minute 2) [35], *Tnfrsf11b* (tumor necrosis factor receptor superfamily, member 11b) [36] and *Nedd9* (neural precursor cell expressed, developmentally down-regulated 9) [37] (Figure 2B). In addition to these hypoxia-associated factors, several other novel transcripts demonstrated this divergent regulatory behavior, *e.g. Dspg3* (dermatan sulphate proteoglycan 3) and the neuronally-responsive obesity-related gene *Mtch2* [38]. Interestingly, two factors demonstrated an upregulation at 4% O_2_ but downregulation at the other oxygen tensions, *i.e.* the neurotrophin receptor signaling modulator *Ndr4* (*Ndrg4*) [39], [40] and the hypoxemia-associated *Gstp1* (glutathione s-transferase pi 1) [41]. Our data also suggest the presence of entirely novel astrocytic oxygen-responsive factors, such as Tec tyrosine kinase (*Tec*) [42], nidogen 2 (*Nid2*) [43], tweety (*Ttyh1*) [44] and tenomodulin (*Tnmd*) [45].

The *low tension*-responsive genesets (regulated at both 1 and 4% O_2_: 5) demonstrated a significant divergence of functional output, as the upregulated genes in this subset were again profoundly dominated by ribosomal factors, *i.e.* 59.1% of upregulated shared transcripts were ribosomal genes (*e.g*. *Rpl41*, *Rps8*, *Rpl29*, *Rps27 etc.*), while there were no downregulated ribosomally-related transcripts. Among the upregulated transcripts there were also factors associated with prolyl amino acid metabolism and neurodegeneration such as *Pin1* (peptidyl prolylisomerase). The downregulated *low tension*-responsive transcripts were more functionally diverse than the corresponding upregulated subset, *i.e.* significant downregulation of genes controlling G protein-coupled receptor function (*Gprasp1*-GPCR-associated sorting protein-1, *Gpr23*-G protein-coupled receptor23, *Gpr51*-G protein-coupled receptor 51), cell viability (*Sesn1*-sestrin-1, *Tsn*-translin), cell signaling (*Stk6/Aurka*-serine/threonine kinase 6, *Ptgds*-prostaglandin d-synthase), and perhaps most importantly considering the primary functions of astrocytes, cell to cell communication/junction formation (*Aqp1*-aquaporin, *Gjb2*-gap junction membrane channel protein beta 2, *Vezt*-vezatin, *Dcn*-decorin).

The *intermediate tension*-responsive subsets (regulated at both 4 and 9% O_2_: Table S6) also demonstrated a significant enrichment of ribosomally-connected upregulated transcripts (17% of upregulated transcripts were ribosomally-associated). In addition to the multiple ribosomal transcripts, several factors directly linked to hypoxic environments, *e.g.* carbonic anhydrase (*Ca3*), lipocalin 2 (*Lcn2*) [24] and biglycan (*Bgn*) [46] were significantly upregulated in this subset. Along with these transcripts we noted a significant upregulation in latexin (*Lxn*), a specific inhibitor of zinc-dependent metallocarboxypeptidases, and the growth factor *Gdf10*. Within the downregulated *intermediate tension*-responsive geneset there were several genes involved in maintenance of endothelial function (*Esm1*-endothelial cell-specific molecule 1: *Edg2*-lysophosphatidic acid receptor) and a series of genes controlling cell surface receptor expression (*Arl6ip6*-ADP-ribosylation-like factor 6-interacting protein 6, *Sorl1*-sortilin-related receptor, *P2rxl1*-purinergic receptor P2X-like 1, *Tfr*-transferrin, *Dnai2*-dynein).

The *cross-tension* responsive gene subset (regulated at both 1% and 9% O_2_: Table S7) was again strongly divided between up and downregulation with respect to ribosomal activity. The upregulated *cross-tension* subset again demonstrated a strong ribosomal phenotype (20.4% of transcripts ribosomally-linked). The downregulated *cross-tension* responsive subset however contained no ribosomally-related genes. The *cross-tension* subset was heavily populated by genes involved in energy regulation (*Tpi1*-triosephosphate isomerase, *Ldh*-lactate dehydrogenase, *Pygb*-glycogen phosphorylase, *Gapdh*, *Gpi1*-glucose phosphate isomerase, *Pfkl*-phosphofructokinase) and protein chaperoning (*Hsp27*-heat shock protein 27 kDa, *Gadd45b*-growth arrest and DNA-damage-inducible 45-β, *Hsp1*-heat shock protein 1, *Hsp8*-heat shock protein 8). Interestingly, as with the upregulation of peptidyl prolylisomerase in the *low tension* geneset, again a small group of transcripts, involved in proline-directed peptide modification, were also upregulated (*Dpp7*-dipeptidylpeptidase 7, *P4hb*-prolyl-4 hydroxylase). With respect to the downregulated *cross-tension* responsive subset we noted that stanniocalcin (*Stc1*), typically upregulated in peripheral tissues such as the heart [47], was downregulated in the cortical astrocytes. However, it should be noted that in the Westberg study [47], the stanniocalcin transcriptional upregulation was induced by a hypoxic preconditioning action, rather than a chronic exposure, as in our paradigm. Amongst the downregulated transcripts in the *cross-tension* subset (Table S7), there were functional groups involved with transcriptional regulation (*Zfp426*-zinc finger protein 426, zinc finger, *Morc2*-CW-type with coiled-coil domain 1, *Zfp36*-zinc finger protein 36), protein trafficking (*Snx14*-sorting nexin 14, *Srpr*-signal recognition particle receptor) and structural/connective component regulators (*Adamts9*-A disintegrin-like and metalloprotease (reprolysin type) with thrombospondin type 1 motif 9, *Srrm2*-serine/arginine repetitive matrix 2, *Fbn2*-fibrillin 2, *Agrn*-agrin). Downregulation of several receptor-related factors linked to the amelioration of cell damage or hypoxic stress was also observed in the *cross-tension* subset, *e.g.* aryl hydrocarbon receptor (*Ahr*), which was recently linked to hypoxic regulation of carbonic anhydrases [48]. In addition to downregulation of this receptor we also noted reductions in the levels of the latrophilin (*Lphn1*) and platelet-derived growth factor (PDGF) (*Pdgfra*) receptors. It has been previously shown that α-latrotoxin, the active component in black widow spider venom, can selectively stimulate astrocytic cells, inducing death [49], therefore *Lphn1* downregulation would be beneficial in times of stress. G protein-coupled receptors can transactivate PDGF receptors [50], and GPCR ligands such as angiotensin appear to control astrocyte growth via PDGF receptors [51]. Therefore, during periodic hypertension or ischemic stress, alterations in PDGF receptor signaling may control astrocyte proliferation or activation of downstream transcription factors such as myc. Reinforcing this concept, was our observation that N-myc downstream-regulated 4 (*Ndr4*) was also significantly downregulated in this *cross-tension* subset.

### Parametric geneset enrichment analysis

To fully exploit the depth of transcriptomic information contained within any specific geneset, it is crucial to perform as great a functional correlational analysis between individual genes as possible. It is clear that concerted gene group responses underpin multiple and diverse areas of biology [15], [52]. Therefore computational analysis and clustering of genes into specific and unbiased functional groups is vital to fully appreciate the subtleties of transcriptional responses to environmental or experimental perturbations and place a physiological relevance to the experimental regulation of individual genes.

**Figure 3:**
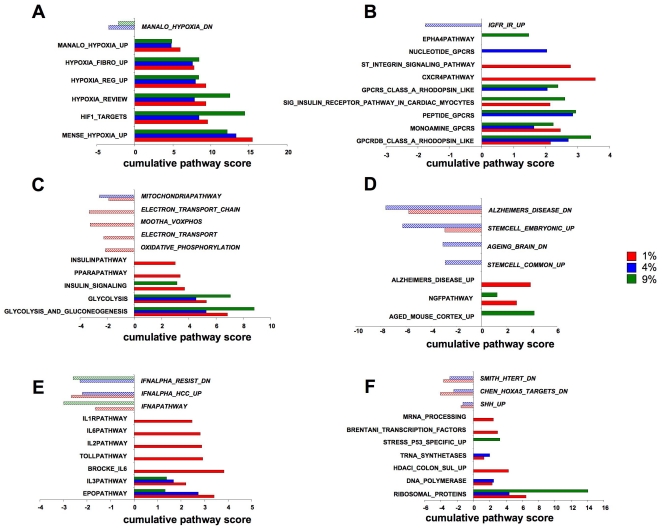
Focused MSigDB PAGE pathway collection analysis of oxygen tension-dependent gene transcription. (**A**) Oxygen-tension related PAGE gene pathway collections generated by the multiple oxygen tension environments. (**B**) Receptor signaling-related PAGE gene pathway collections generated by the multiple oxygen tension environments. (**C**) Energy regulation-related PAGE gene pathway collections generated by the multiple oxygen tension environments. (**D**) Aging- related PAGE gene pathway collections generated by the multiple oxygen tension environments. (**E**) Cytokine-related PAGE gene pathway collections generated by the multiple oxygen tension environments. (**F**) Translation and stress-response- related PAGE gene pathway collections generated by the multiple oxygen tension environments. The magnitude of the specific pathway collection cumulative z-scores are indicated in the scale at the bottom of each histogram (**A**–**F**). Filled color bars indicate PAGE collections populated with genes with a positive cumulated z-score, striped bars indicate PAGE collections populated with genes with a negative cumulated z-score. Each PAGE pathway collection group was significantly populated by at least two separate genes with a co-probability of p≤0.05.

To investigate higher-order transcriptional functionality, parametric geneset enrichment analysis (PAGE) was performed using the MSigDB gene collections (http://www.broad.mit.edu/gsea/msigdb/index.jsp). Significant gene population of a given PAGE geneset collection is created by the inclusion of multiple genes (≥2 per PAGE pathway collection: p≤0.05) with the resultant z ratio scores being summated to create a total PAGE pathway collection overall Z-score. Therefore signaling pathway groups, possessing many co-upregulated genes will possess a strong positive Z-score, and those significantly populated by multiple correlated downregulated genes will possess a negative Z-score. The complete significantly-populated PAGE pathway collection diagrams generated by the three specific O_2_ tension genesets (compared to 20% O_2_) are represented in Figure S1, and summarized in Tables S8, S9, S10 (Figure S1A-1% O_2_, Table S8; Figure S1B-4% O_2_, Table S9; Figure S1C-9% O_2_, Table S10). To reduce the inherent complexity of multiple PAGE analyses, we grouped related functional pathways (10 highest-scoring) to highlight their specific relationship to the divergent oxygen tensions (Figure 3). PAGE pathways that demonstrated significant (p≤0.05) transcript population were grouped into functional areas controlling: oxygen tension responses (Figure 3A); receptor-mediated processes (Figure 3B); energy metabolism (Figure 3C); neurophysiology and pathology (Figure 3D); cytokine physiology (Figure 3E) and transcriptional/translational activity (Figure 3F). Demonstrating the strong *in vivo* physiological correlation of our astrocytic culture conditions, we identified multiple oxygen-responsive pathways strongly represented in our datasets (Figure 3A: *MENSE HYPOXIA UP*, *HYPOXIA REVIEW*, *HYPOXIA REG UP*, *HYPOXIA FIBRO UP*, *HIF1 TARGETS*). In addition to the positive cumulative pathway Z-scores of these sets, we also observed a negative score (indicative of multiple down-regulated genes operating in a concerted manner) for PAGE collections typically downregulated with hypoxia (*MANALO_HYPOXIA_DN*). While hypoxia and oxidative stresses may be considered biophysical phenomena, many of the functional effects of such conditions appear to bear signaling similarities to receptor-mediated effects [12]. In line with this, a strong linkage of the oxygen tension-modulated sets was observed with receptor functions (Figure 3B), including G protein coupled (*GPCRS_CLASS_A_RHODOPSIN_LIKE*, *CXCR4PATHWAY*), integrin-related (*ST_INTEGRIN_SIGNALING_PATHWAY*) and receptor tyrosine kinase receptors (*EPHA4PATHWAY*). Oxidative stresses may disrupt cellular energetics by causing a mechanistic shift away from mitochondrial oxidative phosphorylation to other forms of cellular energy production [11]. Indeed, we observed a strong clustering of upregulated energy-regulatory pathways associated with alternative energy production mechanisms (Figure 3C: *GLYCOLYSIS_AND_GLUCONEOGENESIS*, *PPARAPATHWAY*), with a concomitant down-regulation of the mitochondrial oxidative phosphorylation functions (*ELECTRON_TRANSPORT*, *MOOTHA_VOXPHOS*, *MITOCHONDRIA_PATHWAY*). As oxidative and hypoxic events are often associated with the aging process and pathology [2], [7], [8], [11] we extracted PAGE pathways associated with these functions (Figure 3D). We noted that there was strong positive transcript population of age-related pathways (*AGED_MOUSE_CORTEX_UP*, *ALZHEIMERS_DISEASE_UP*), with a coherent negative population of converse pathways (*ALZHEIMERS_DISEASE_DN*, *AGEING_BRAIN_DN*). In addition to oxidative events, nervous system aging has also been strongly associated with attenuation of functional stem cell capacity [53]. In accordance with this, we noted that hypoxic conditions downregulated transcripts controlling the upregulation of stem cell populations (Figure 3D; *STEM_CELL_COMMON_DN*, *STEMCELL_EMBRYONIC_UP*), indicating a potential ‘pro-aging’ phenotype of the datasets. Interestingly, we also noted that there were several other aging–related groups (*HG PROGERIA DN*, *OLDAGE DN*: Table S9, Table S10) that were profoundly populated (with negative cumulative Z-scores) suggesting that an effect of higher oxygen tension may be to prematurely age cell populations.

In addition to oxidative events and alterations in stem cell populations, advancing age and neurological disorders also are linked to cytokine-related inflammatory processes [54]. Specific cytokine functional groups were also demonstrated to possess strong positive transcript population (Figure 3E: *IL3PATHWAY*, *IL6PATHWAY*), while interferon-controlling transcript PAGE collections demonstrated significant negative transcript population (Figure 3E: *IFNAPATHWAY*, *IFNALPHA_HCC_UP*). The various oxygen tensions appear to induce a potent stress response in the astrocytic cells, perhaps indicating their importance as damage-mitigating entities in the central nervous system. We therefore extracted PAGE pathways linked to stress responses and translational activity, functions that may be required to replace/correct tissue damage (Figure 3F). With the various oxygen tensions we identified a strong positive transcript population of protein synthetic (*RIBOSOMAL_PROTEINS*, *TRNA_SYNTHETASES*) and stress-response pathways (*STRESS_P53_SPECIFIC_UP*), cellular development pathways were inhibited (negative transcript population of *SHH_UP*) and cell cycle inhibitory pathways were antagonized (*SMITH_HTERT_DN*) (Figure 3F).

**Figure 4:**
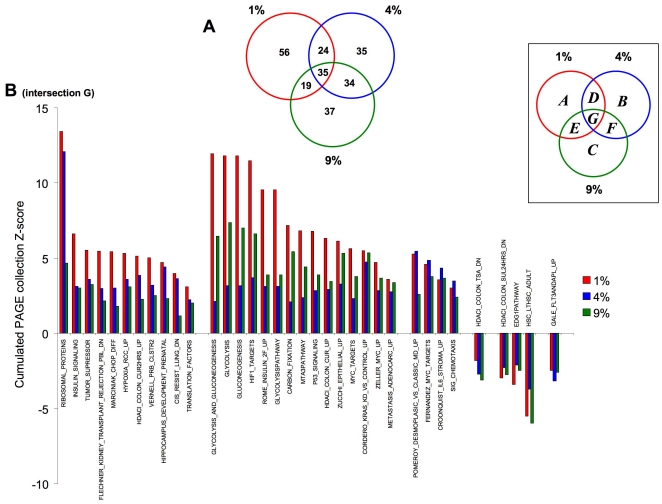
Venn diagram analysis of oxygen tension-dependent transcriptional PAGE gene collection population in primary astrocytes. (**A**) Three-way proportional Venn diagram for transcriptional responses (compared to ambient 20% O_2_) for 24 hour exposure to 1% (red circle), 4% (blue circle) and 9% (green circle) ambient oxygen tension. The intersection subsets (A–G) are specified in the associated boxed key. (**B**) Oxygen tension-dependent variations in transcriptional regulation of multi-tension responsive genes from intersection G (1% O_2_, red; 4% O_2_, blue; 9% O_2_, green).

We next analyzed the transitional relationships of PAGE collections between oxygen tensions (Figure 4), in a similar manner to the primary gene transcripts. The Venn diagram distribution of the PAGE pathway collections demonstrated a greater functional similarity, compared to gene transcripts, between PAGE collections across the different O_2_ tensions. Hence, the percentages of PAGE collections unique to each tension were lower than those for the gene distribution (Figure 2A) (1% O_2_-41.8%; 4% O_2_-27.3%; 9% O_2_-29.6%), suggesting a degree of functional convergence between datasets. The specific PAGE collections found in the annotated intersections (Figure 4, A–G) are listed in Table S11. The *low tension* responsive PAGE group (Table S11, D) was primarily composed of growth factor and developmental PAGE collections (*HIPPOCAMPUS DEVELOPMENT POSTNATAL*, *EGF HDMEC UP*, *IFN BETA GLIOMA DN*, *ST INTEGRIN SIGNALING PATHWAY*). The *intermediate tension* responsive group (Table S11, F) again was interestingly devoid of energetic, hypoxic or ribosomal pathways, yet possessed a strong representation of cytoskeletal pathways (*RHOPATHWAY*, *SIG REGULATION OF THE ACTIN CYTOSKELETON BY RHO GTPASES*). The *cross-tension* responsive group (Table S11, E) also failed to demonstrate any strong ribosomal or energy regulation bias but did contain several pathways linked to cellular signaling (*P38MAPKPATHWAY*, *CALCINEURIN NF AT SIGNALING*, *PTDINSPATHWAY*).

In sharp contrast to the primary gene distribution (Figure 2A), there were proportionately a greater number of *multi-tension* PAGE collections common between all three experimental oxygen tensions (Table S11, G). Out of these, many were directly related to hypoxic responses, ribosomal activity or glucose regulation, suggesting that the astrocytes may possess several transcriptomic ‘programs’ that can coherently respond across a wide range of ambient oxygen tensions. In a profound contrast to this *multi-tension* group, the other major intersections of upregulated pathways did not share such a diverse functional ‘signature’. The specific transitional nature of PAGE collection population for this *multi-tension* set is depicted in Figure 4B. Multiple PAGE collections in this intersection-G (31% of *multi-tension* PAGEs) demonstrated an ascending increase of Z-score with reduced oxygen tension (*e.g. RIBOSOMAL PROTEINS*, *INSULIN SIGNALING*, *TUMOR SUPPRESSION*, *HYPOXIA RCC UP*). However the most consistent oxygen tension-dependent trend of regulation, was increased Z scores at 1% or 9% O_2_, pivoting around the 4% O_2_ level (43% of the *multi-tension* PAGEs in intersection G). This pattern of PAGE regulation included important pathways involved in energy regulation (*GLYCOLYSIS AND GLUCONEOGENESIS*, *GLYCOLYSIS PATHWAY*, *CARBON FIXATION*), cell growth and development (*P53 SIGNALING*, *MYC TARGETS*, *METASTASIS ADENOCARC UP*) and classical hypoxia-related factors (*HIF1 TARGETS*). As there seemed to be important transitional changes, *e.g.* pivoting around 4% O_2_, in signaling pathways (composed of multiple, functionally-related transcripts) between the diverse oxygen environments we decided to evaluate the importance of these transitional regulatory patterns using *k-means* clustering [55] across the multiple O_2_ tensions.

### Morphometric analysis of oxygen tension transitional responses in primary cortical astrocytes

**Figure 5:**
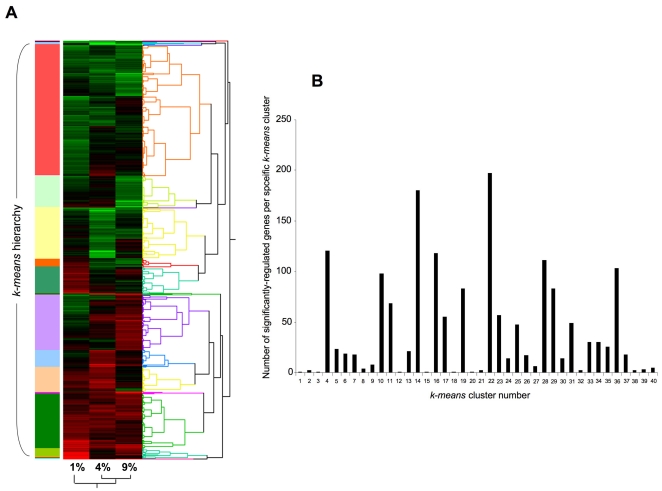
*K-means* clustering analysis of oxygen tension-dependent transcriptional responses in rat primary astrocytes. (**A**) Hierarchical *K-means* clustering of gene transcriptional responses in astrocytes to 24 hour exposure to 1, 4 or 9% O_2_ conditions relative to the transcriptional expression levels at 20% O_2_ tension. *K-means* clustering attempts to group together transcriptional activity with similar trends across the multiple O_2_ tensions. Colored blocks and the associated dendrogram structure indicate the gross nature of the identified hierarchical *k-means* functional activity clusters. (**B**) Histogram represents the numbers of significantly regulated genes in the most distinct gene transcriptional activity clusters numbered 1 to 40. Hierarchical clustering was performed and mathematically assessed using DIANE 6.0/JMP across the three oxygen tensions.

**Figure 6:**
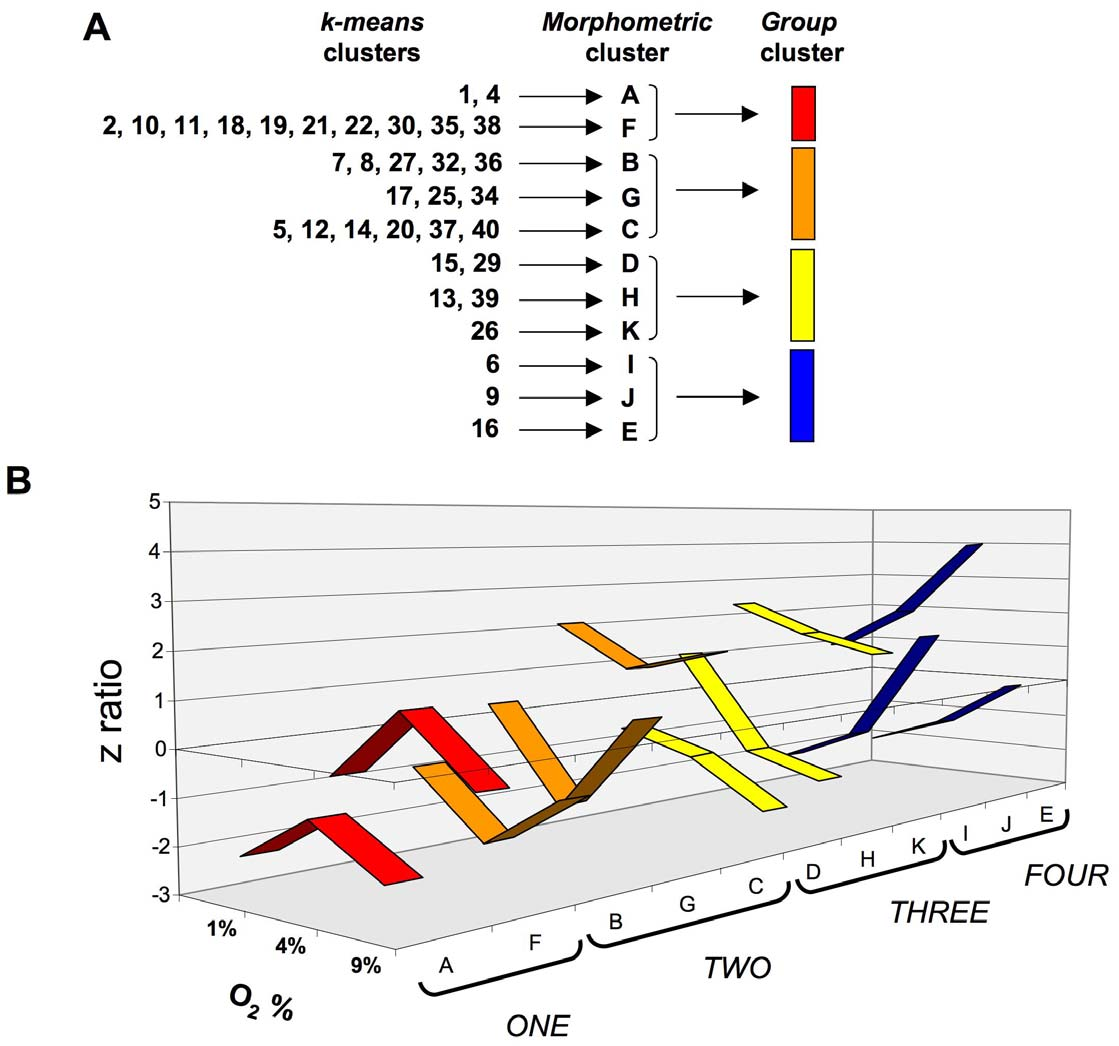
Clustering compression for primary oxygen tension transitional *k-means* analysis. (**A**) Specific description of compression of *k-means* clusters into morphometric clusters (A–K) and eventually four group clusters (*ONE*-*FOUR*). (**B**) Numerical z ratio comparisons of individual morphometric clusters that comprise the larger group clustering (*ONE*-*FOUR*).

**Figure 7:**
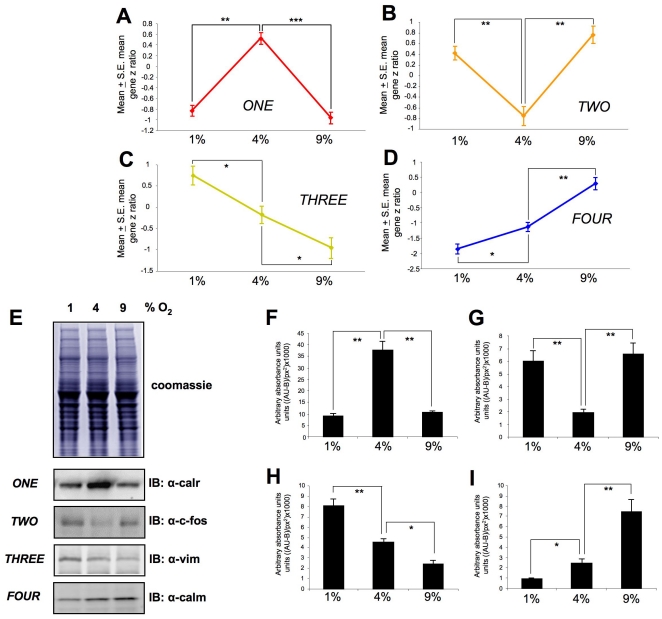
Astrocyte oxygen tension-dependent transcript group cluster structure. Panels (**A**) (group *ONE*), (**B**) (group *TWO*), (**C**) (group *THREE*) and (**D**) (group *FOUR*) represent the mean ± standard error, group cluster z ratios generated from *k-means* clustering across the three experimental oxygen tensions. (**E**) Coomassie-stained gel demonstrating equal total protein loading from astrocyte cultures exposed to the multiple oxygen tensions. Representative western blots for exemplar proteins from each group are depicted and additionally quantified in panels (**F**–**I**). Protein expression is represented via measurement of immunoreactive absorbance units with background subtraction per square pixel ((AU-B)/px^2^). Statistical significance was estimated with a Student's t-test using GraphPad Prism version 5.02 (GraphPad Software Inc.). *p*≤0.05 = *, *p*≤0.01 = **, *p*≤0.001 = ***.

Hierarchical *k-means* clustering was performed using the primary transcriptomic datasets generated from the three different oxygen tension conditions compared to the 20% control O_2_. Simultaneous clustering of all three experimental datasets, using DIANE-6.0/SAS-JMP7.0, into clusters that possessed similar directions of transcription changes across the three experimental oxygen tensions (relative to 20% O_2_) generated 40 specific gene clusters (Figure 5A, Table S12). These 40 *k-means* clusters were highly divergent with respect to the number of significantly-regulated genes contained within them, *i.e.* ranging between only one gene to 197 genes (Figure 5B). The average number of transcripts in each cluster was 40.9±7.98 (standard error of mean). Due to this high diversity of *k-means* clusters present within the transcript datasets, we chose to simplify the multiple *k-means* clusters through pattern analysis. The initial 40 clusters were therefore first contracted into multiple, morphometrically-classed clusters (morphometric clusters: A–K), based on the transitional gene regulation polarity across the O_2_ tensions, and then into color-coded, larger group clusters (*ONE*-*TWO*-*THREE*-*FOUR*) (Figure 6A, B). The oxygen tension transitional patterning of these morphometric and group clusters, across the experimental oxygen tensions, is depicted in Figure 6B. Hence group cluster *ONE* demonstrates transcript elevation at 4% O_2_ compared to 1% and 9% O_2_. Group cluster *TWO* demonstrates transcript reduction at 4% O_2_ compared to 1% and 9% O_2_. Group cluster *THREE* represents consistent potentiation of transcription with increasing hypoxia, while group cluster *FOUR* demonstrates the opposite pattern to cluster *THREE*. To demonstrate the strong morphometric relationship of all the original 40 *k-means* clusters that were compressed into the group clusters *ONE*-*FOUR*, we averaged (± standard error) the mean z ratios across the three experimental tensions for all the genes in the *k-means* clusters (Figure 7A–D). This analysis demonstrated a strong and significant coherency of the four distinct group clusters of oxygen-sensitive transcripts. We additionally performed western blot analysis, for the gene products of exemplars from each group cluster (*ONE* to *FOUR*), upon protein lysates from astrocytes exposed to the various oxygen tensions, to confirm the transcriptional variation seen with our array data (Figure 7E–I). Each of the exemplar gene products assessed (*c-fos*, *Calr*, *Vim*, *Calm*) demonstrated a similar oxygen tension response pattern of protein expression, when compared to the group cluster mean gene transcript regulation (Figure 7E–I).

### Physiological and phenotypic characterization of oxygen-tension sensitive astrocyte transcriptome clusters

**Figure 8:**
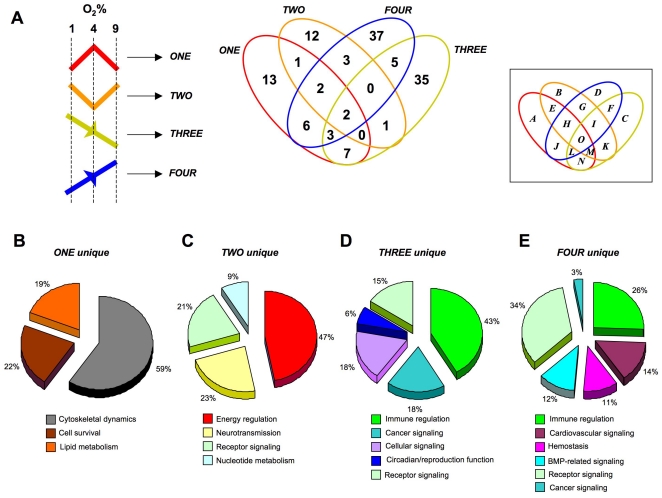
Canonical signaling pathway analysis of group cluster composition. (**A**) Four way Venn diagram analysis of significantly populated canonical signaling pathways populated by group clusters *ONE*, *TWO*, *THREE* and *FOUR*. (**B**–**E**) Proportional pie charts created from multiple associated canonical signaling pathway *hybrid* scores generated by the group cluster-unique (**B**-*ONE* unique; **C**-*TWO* unique; **D**-*THREE* unique; **E**-*FOUR* unique) signaling pathways populated by at least two genes with a co-probability of at least ≤0.05. Associated percentage values indicate the relative distribution of the respective pathway scores for each unique group cluster.

**Figure 9:**
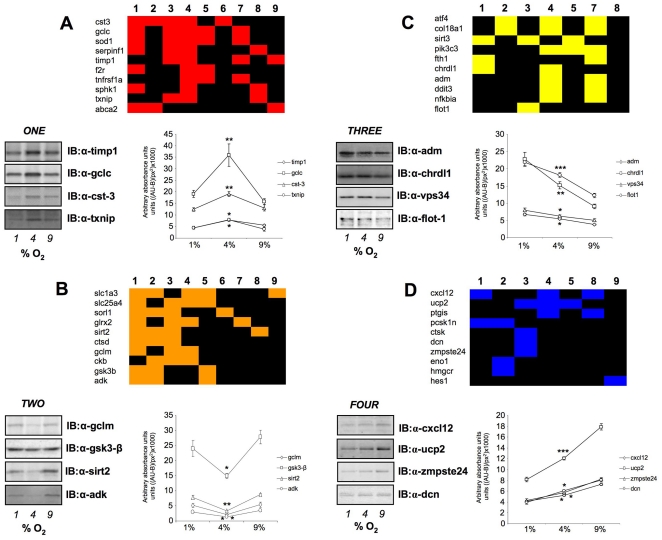
Latent Semantic Indexing analysis of group clusters ONE, TWO, THREE, FOUR with astrocytic and neurophysiological activities. Significantly regulated transcripts from the four respective group clusters (*ONE*-*FOUR*) were individually analyzed by latent semantic indexing (LSI) using GeneIndexer with the following user-defined interrogation terms: 1-*neurodegeneration*; 2-*Alzheimer's*; 3-*aging*; 4-*ischemia*; 5-*neuroprotective*; 6-*cognition*; 7-*hyperoxia*; 8-*hypoxia*; 9-*astrocyte*. (**A**) Block matrix of the ten highest LSI correlating genes from group *ONE* according to each interrogation term (numbered 1–9 atop the block). Four selected genes were verified from each group cluster LSI block. The quantitative and statistical analysis of at least three separate westerns for each chosen protein is indicated in each panel. Statistical significance of the variation of the 1% and 9% datapoints from the 4% O_2_ datapoint is demonstrated in a manner similar to that previously performed (Figure 7A–D). This similar form of statistical analysis was subsequently performed for panels B–D. (**B**) Block matrix of the ten highest LSI correlating genes from group *TWO*. (**C**) Block matrix of the ten highest LSI correlating genes from group *THREE*. (**D**) Block matrix of the ten highest LSI correlating genes from group *FOUR*. Input interrogation terms not implicitly correlating with any significantly regulated transcripts in panels (**C**) and (**D**) were omitted from the matrix. Statistical significance was estimated with a Student's t-test using GraphPad Prism version 5.02 (GraphPad Software Inc.). *p*≤0.05 = *, *p*≤0.01 = **, *p*≤0.001 = ***.

To create a generalized assessment of the physiological signaling significance of the specific group clusters (and regulated genes therein) identified within the astrocyte datasets (*ONE* to *FOUR*), we performed an unbiased canonical signaling pathway analysis (Ingenuity Pathway Analysis v. 8.5). Venn diagram delineation of the significantly populated (p≤0.05, at least two genes per pathway) canonical pathways demonstrated that the four group clusters contained primarily distinct signaling functions, with only a small degree of cross-over (Figure 8A: Table S13). Clustering of the significantly-regulated signaling pathways unique to group cluster *ONE* (Table S14), were primarily concerned with cytoskeletal architecture control, cell death/survival control and lipid metabolism (Figure 8B). The pathways unique to group cluster *TWO* (the morphometric inverse of group cluster *ONE*), unsurprisingly demonstrated a distinct signaling phenotype, *i.e.* the signaling pathways were associated with energy regulation, nucleotide metabolism and inhibitory neurotransmission (Figure 8C: Table S14). As with group clusters *ONE* and *TWO*, groups *THREE* and *FOUR* were characterized by their opposite oxygen tension-regulated behavior. Superficially however, we noted some similarities in the signaling pathway output of these groups, *i.e.* both involved cellular signaling pathways associated with immune regulation and receptor signaling (Figure 8D, E). The specific predicted nature of the immune regulation and receptor signaling pathways however demonstrated a significant functional divergence (Table S14). For example, the receptor signaling profile of cluster *THREE* consisted of *glucocorticoid*, *endothelin-1*, *angiopoietin*, *erythropoeitin*, *neurotrophin* and *estrogen* receptor signaling, while the receptor profile in group FOUR consisted of *dopamine*, *melatonin*, *glutamate*, *chemokine (Cxcr4)*, *insulin-like growth factor-1*, *corticotropin-releasing hormone* and *androgen* receptor signaling instead (Table S14). The largest gross functional differences observed between group clusters *THREE* and *FOUR* involved the significantly larger representation of cancer-related pathways in group *THREE*, compared to *FOUR*, as well as the presence of hemostatic, bone-metabolism and cardiovascular signaling groups specifically in group cluster *FOUR*. It therefore appeared that, along with their morphometric divergences, *i.e.* group clusters *ONE*/*TWO* versus *THREE*/*FOUR*, their predicted signaling profile was quite distinct as well. In general terms it appeared that group clusters *ONE* and *TWO* were more strongly associated with more physiological processes associated with astrocytes, while group clusters *THREE* and *FOUR* were more linked to pathology-associated actions. This partitioning may be due to morphometric basis of the dynamic responsiveness of these groups, *i.e. ONE* and *TWO* demonstrate bi-modal regulation and a fulcrum around 4% O_2_, while *THREE* and *FOUR* seem merely responsive to progressive and non-fluctuating changes in oxygen tension. To test this hypothesis we utilized GeneIndexer to perform latent semantic indexing (LSI), a bioinformatic process that allows multidimensional correlation of user-defined ‘interrogation’ terms with the significantly regulated transcripts within a dataset [11], [12], [17]. Using LSI we interrogated the transcripts contained in the four group clusters (*ONE* to *FOUR*) with terms related to potential astrocyte functions as well as neurological activity (terms 1–9: Figure 9). LSI correlation scores for genes in the group cluster lists with the input interrogation terms were only considered if they were >0.1 (*i.e.* demonstrating implicit correlation). Two-way matrices of the implicitly correlated transcripts within the group clusters (indicated with colored blocks in the matrix) with the specific input interrogation terms are depicted in Figure S2 (*ONE*), Figure S3 (*TWO*), Figure S4 (*THREE*) and Figure S5 (*FOUR*). The ten most highly correlated (greatest LSI correlation score across multiple interrogation terms) transcripts from each group cluster LSI matrix are indicated in Figure 9 (9A-*ONE*, 9B-*TWO*, 9C-*THREE*, 9D-*FOUR*), along with specific western blot protein validation of multiple significantly-regulated transcripts from each group cluster. Each of the gene products assessed, from the four group clusters, demonstrated similar oxygen tension response patterns to their original group cluster designation. Interestingly, multiple examples of the transcripts, that were in the top ten highest correlations to the input GeneIndexer interrogation terms (Figure 9A–D), also formed key nodes within the most statistically reliable, functional gene networks created using the Ingenuity Pathway Analysis (IPA) network-creating algorithm (Figure S6; Table S15). This may suggest that the LSI analysis has extracted highly relevant and functionally co-related genes in each of the group clusters. For example, in cluster *ONE*, both *Cst-3* and *Timp1* were in the top ten of LSI correlations and were present in the highest ranking functional interaction network (Figure 9A, Figure S6A). In cluster *TWO*, both *Adk* and *Sirt2* were identified by both techniques (Figure 9B, Figure S6B). For cluster *THREE*, we co-identified three separate transcripts using combined LSI and IPA-network analysis, *i.e. Vps34*/*Pik3c3*, *Ddit3* and *Col18a1* (Figure 9C, Figure S6C). With respect to group cluster FOUR, using both LSI and IPA network analysis, we co-identified *Dcn*, *Cxcl12* and *Ucp2* (Figure 9D, Figure S6D).

In agreement with our previous analyses, the general predicted functions of the highest scoring gene networks (generated using IPA algorithms) also displayed a variance between clusters *ONE*/*TWO* and *THREE*/*FOUR*. Clusters *ONE* and *TWO* demonstrated a functional linkage, as both were associated with physiological homeostatic and synthetic regulatory mechanisms (top predicted network functions: *ONE*-cellular assembly and organization, cellular function and maintenance, protein synthesis: *TWO*-cellular development, protein synthesis, carbohydrate metabolism) (Figure S6; Table S15). In contrast, the highest scoring gene interaction networks present in clusters *THREE* and *FOUR*, were more closely associated with pathology and homeostatic disruption (Top predicted network functions: *THREE*-metabolic disease, renal and urological disease, cell death: *FOUR*-endocrine system disorders, cellular assembly and organization, metabolic disease) (Figure S6; Table S15).

**Figure 10:**
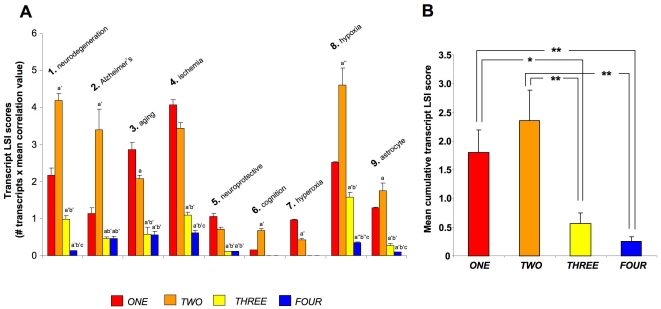
Comparative analysis of group cluster gene correlation scores to astrocytic and neurophysiological activity terms. (**A**) Representation of the mean transcript LSI score for the four group cluster genesets analyzed with nine separate LSI GeneIndexer interrogation terms. The numerical value of each bar was calculated by the multiplication of the number of implicitly correlated genes for each group cluster (*ONE*-*FOUR*)-interrogation term (1–9) pair, with the number of implicitly-correlated genes present for each group cluster-interrogation term pair. Statistical significance is indicated as follows: *TWO*, *THREE*, *FOUR* compared to *ONE*, *p*≤0.05 = a, *p*≤0.01 = a′, *p*≤0.001 = a″; *THREE*, *FOUR*, compared to *TWO*, *p*≤0.05 = b, *p*≤0.01 = b′, *p*≤0.001 = b″; *FOUR* compared to *THREE*, *p*≤0.05 = c, *p*≤0.01 = c′, *p*≤0.001 = c″. (**B**) Mean cumulative transcript LSI scores (± standard error) for all of the nine interrogation terms for each complete group cluster. Statistical significance was estimated with a Student's t-test using GraphPad Prism version 5.02 (GraphPad Software Inc.). *p*≤0.05 = *, *p*≤0.01 = **, *p*≤0.001 = ***.

To further investigate these potential gross functional differences in the four group clusters we compared, using a novel methodology, the cumulative cluster transcript LSI scores. Cumulative transcript LSI scores were calculated as follows: the number of implicitly correlated transcripts in each group cluster linked to a specific interrogation term (Table S16), were multiplied by the mean correlation score of the transcripts implicitly associated with the input interrogation term (Table S17). It was evident from this comparison that clusters *ONE* and *TWO* demonstrated a much stronger association with astrocyte-related functions as well as neurological activities, compared to group clusters *THREE* and *FOUR* (Figure 10A). Averaging across the multiple transcript/interrogation term correlations, we demonstrated that group clusters *ONE* and *TWO* possessed a statistically significantly greater cumulative transcript LSI score compared clusters *THREE* and *FOUR* (Figure 10B). Therefore it seems that the group clusters characterized by bi-modal sensitivities to oxygen tension, that pivot around 4% O_2_, appear to be more associated with physiological responses when compared to the *THREE*/*FOUR* uni-modal clusters. These findings may suggest that distinct, and physiologically specific, transcriptional programs can be elicited by subtle differences in the dynamic nature of oxygen tension modulation in astrocytes. Therefore, from our transcriptional-transitional profile analysis, it appears that cortical astrocytes potentially possess multiple modes of response to their ambient oxygen tensions and that these can entrain distinct, functional activities mediated by diverse, yet synergistic, cellular signaling systems.

## Discussion

In this study we have investigated, in a multidimensional manner, how alterations in ambient oxygen tension can affect the transcriptional activity of primary rat astrocytes. With the application, and transition between, multiple ambient oxygen tension environments, we have noted that primary cortical astrocytic cells generate considerable transcriptomic changes. Many of the patterns of transcriptional activity suggest the presence of underlying ‘programs’ of co-regulated transcripts, that are differentially sensitive to multiple levels of ambient oxygen tension.

Astrocytes form a specific population of glial cells that not only create part of the blood brain barrier, but also represent a point of control of the global cerebral vascular blood flow. Astrocytes are often found in close intimate contact with microvessels in the brain and their ability to release vasodilatory or vasoconstrictory factors in response to neuronal activity allows them to divert away, or increase perfusion of blood to different areas of the brain [4]. Additionally, in response to local vascular injury it would be beneficial to reduce the flow to that region if there is considerable vascular damage. In the converse scenario, astrocytes may also be required to increase perfusion to areas of the brain that may be temporarily ischemic. With respect to these functional attributes it is not surprising that astrocytes have been demonstrated to be exquisitely sensitive to ambient oxygen tensions [56]. We have exposed primary cortical rat astrocytes to multiple levels of ambient oxygen tension in our experimental paradigms, *i.e.* 1%, 4%, 9% and the standard atmospheric oxygen tension, 20% O_2_. These levels were selected as we considered 4% O_2_ to potentially represent physiological normoxia for the CNS [1], [5], and so 1% O_2_ represents relative CNS hypoxia, elative hyperoxia was examined at 9% O_2_ (considered normoxic in other tissues) and 20%, which is the standard condition for tissue culture. We have demonstrated that there are highly significant, and contextually intricate, variances in the transcriptomic responses of cortical astrocytes at these different oxygen tensions. This finding is crucial with respect to many of the current experimental conditions employed by the majority of research groups. Thus, *in vitro* culture of neurons and astrocytes is commonly conducted using humidified atmospheres of 95% air, 5% CO_2_, *i.e.* 20% O_2_, clearly a hyperoxic situation compared to *in vivo* “normoxia”. Thus, we contend that there will be profound differences in cellular physiology between cells maintained at the ambient oxygen tension and oxygen tensions more likely to occur in the central nervous tissue.

With regards to specific transcriptomic changes induced by different oxygen environments, relative to the standard 20% O_2_ culture condition, we noted the profound downregulation of *Gjb2* (gap junction membrane channel protein beta 2), in response to 1% O_2_ exposure. This gene encodes for the protein connexin 26 which has been linked to congenital deafness that is often induced by congenital anoxia [57]. Interestingly, with respect to the involvement of hypoxic conditions in potential neurodegenerative mechanisms, we detected a profound downregulation of the beta amyloid binding precursor (*Bbp*) [58] and the neuronal regeneration-related protein (*Nrep*) [59] with exposure to 1% O_2_. The modulation of expression of the lipid raft marker, flotillin-1, may also be important both for synaptic transmission (post-synaptic densities are commonly associated with lipid-rich areas) and organization/control of amyloid precursor protein metabolism, as the Aβ-forming gamma secretase enzyme complex is also enriched in lipid raft areas [60]. In addition, flotillin-1 was also recently identified as a functional binding partner for the reversible oxygen binding protein neuroglobin [61].

Correlating to the oxygen tension-modulated alteration of clock-related transcripts, *Per1*, *Per2* and *Cry1* and those linked to protein chaperoning and immune function (*e.g. Schlafen-3*) (downregulated at 9% O_2_ compared to 20% O_2_ control: Table S3) we also observed a significant population of many cell cycle/cancer-related and immune-related canonical signaling pathways (Table S14), suggesting strong causative links between oxygen tension exposure in astrocytes and potential cancer/cell development/cell cycle regulatory issues [62]. Indeed, a re-entry into the cell cycle of even post-mitotic neurons has been suggested as a potential pro-neurodegenerative mechanism [63].

When investigating transcripts that demonstrated cross-oxygen tension regulation patterns, we noted several factors that could functionally control neurological functions via their responsivity to ambient oxygen conditions. In our observed *low tension*-responsive set (Table S4), we identified the upregulation of peptidyl prolylisomerase gene (*Pin1*), which has been strongly implicated in modification of the pathology of Alzheimer's disease [60], [63]; connective tissue growth factor (*Ctgf*) whose expression and effects upon cellular morphology and rearrangement are oxygen sensitive [64] and metallothienein (*Mt1a*), that has been previously shown to control anti-apoptotic mechanisms induced by anoxic/hypoxic conditions [65]. In our observed *intermediate tension*-responsive set (Table S4, F), several other important neurological factors were revealed, *e.g.* latexin (*Lxn*) and growth differentiation factor 10 (*Gdf10*) were both seen to be upregulated in this subset. Latexin, a zinc-dependent metallocarboxypeptidase, has recently been demonstrated to possess the capacity to form long-lasting and highly-stable multioligomeric stable structures in response to stress, in a similar manner to beta-amyloid protein [66], while *Gdf10* (a controller of cellular development, also known as bone morphogenic protein 3B) expression is positively regulated by both Sox-9 and hypoxic mechanisms [67].

Performing PAGE analysis, to generate a higher order of appreciation of the oxygen-dependent transcriptomic responses, we were able to appreciate the broader picture of the functional relevance of the multiple genetic observations recorded. One interesting finding was the clear demonstration of a protein synthetic response ‘pivot’ for cortical astrocytes, (Figure S1B, compared to S1A and S1C). In addition to this protein synthetic pivot, a strong tumor-suppressor and cytokine-related functional phenotype was dominant at the 4% O_2_ tension (Figure 3). Importantly, this oxygen tension may indeed be indicative of the true normoxic condition in central nervous astrocytes [1]. We selected 4% O_2_ as an experimental oxygen tension since the majority of brain tissue normally exists at or below this level: 1% O_2_ therefore represents hypoxia to most nervous tissue whilst 9% O_2_ may represent small arteriolar levels and 20% O_2_ represents levels experienced by most cells in conventional tissue culture. Using morphometric-based clustering of the transcriptomic data (Figures 6, 7), we were able to further characterize important response profiles of the astrocytes in a manner that indicates a potentially wide degree of functional sensitivity in these cells. It was interesting to note that each of the larger morphometric group clusters we statistically identified (*ONE*, *TWO*, *THREE*, *FOUR*), when analyzed for their potential cell signaling activity, demonstrated relatively unique phenotypes (Figure 8). The two most different, from a signaling perspective, were interestingly the two ‘pivoting’ groups (*ONE* and *TWO*). Group *ONE* (and the regulated transcripts therein) seemed likely to manage cytoskeletal dynamics and lipid metabolism, which could potentially indicate a strong role of these genes in modulating neurotransmission in the central nervous system [60], [61]. Group *TWO*, also possessing a similar regulation polarity ‘pivot’ around the 4% O_2_ tension, was primarily linked to energy regulation and inhibitory mechanisms in neurotransmission (*e.g.* GABA receptor signaling, Table S14). As with our previous data, it appears that astrocytes display a form of ‘stability sensor’ for 4% O_2_, and thus coordinate distinct signaling pathways dependent on any subsequent change in ambient O_2_ tension with unique and diverse genetic programs (Figures 9, 10).

Therefore, using successive unbiased clustering techniques, we have identified four morphometrically-distinct transcription ‘programs’ in cortical astrocytes (*ONE*-*FOUR*) (Figure 6). As we have seen, these four transcript clusters presented two distinct morphometric patterns, *i.e.* those with a transcriptional polarity pivot around the 4% O_2_ tension (presumed normoxic: (*ONE* and *TWO*) and those with a uni-modal transcriptional progression (*THREE* and *FOUR*) (Figure 7). A simple interpretation of such transcription models may suggest that the bi-modal groups represent ‘programs’ of transcripts important for homeostasis of the astrocytic function, while the uni-modal programs indicate responses to continuous or uncontrolled oxidative changes in the CNS. Potentially reinforcing this hypothesis we found, with application of multiple and diverse bioinformatic techniques, that the bi-modal clusters (*ONE* and *TWO*) actually demonstrated a functional homeostatic and physiological phenotype (Figures 8, 10) while the uni-modal clusters (*THREE* and *FOUR*) possessed a more pathology-related phenotype. Therefore it may be likely that rather than regulating individual transcripts randomly across multiple oxygen environments, astrocytes needing to regulate local vascular tone and neuronal activity, possess concerted transcriptional ‘programs’ that are engaged in a transitional-activity-dependent manner. Such clustering of transcriptional responses would likely aid rapid minute-to-minute control of complex cellular functions such as release of vasoconstrictory agents and neuroprotective behavior. Analysis of the nature of the specific transcripts clustered together in these different group clusters, indicated several potentially interesting aspects to the oxygen tension regulation of cortical astrocyte function. For example, in cluster *ONE* and *TWO* respectively, we confirmed the presence and regulation of both *Gclc* (glutamate-cysteine ligase catalytic subunit) and *Gclm* (Glutamate-cysteine ligase modifier subunit). These two factors have both recently been intricately implicated in astrocytic stress response mechanisms involving the Nrf2 signaling pathway [68]. The silent mating type information regulation 2, homolog (*Sirt2*), confirmed in cluster *TWO*, has been previously demonstrated to play a crucial role in stress response activities associated with aging [69]. *Sirt2* has also been recently implicated in oxygen tension-related homeostasis, through a regulatory control of the 14-3-3 scaffolding protein and the apoptosis-related factor *Bad* [70]. In addition, we identified and confirmed, in cluster *THREE*, the regulation profile of chordin-like 1 (*Chrdl1*). *Chrdl1* is a bone morphogenetic protein-4 antagonist [71], that has also been demonstrated to act as a regulator of blood vessel dynamics and angiogenesis in retinal pericytes [72]. The progressive increase of expression of *Chrdl1* with ascending hypoxia, therefore may also indicate a similar role in cortical astrocytic tissue. In cluster *FOUR* we found that *Cxcl12* expression was successively reduced by increasing hypoxia. The chemokine CXC motif, ligand 12 (*Cxcl12*), also known as stromal cell-derived factor 1, is a member of the secreted small cytokine family that is considered to be a strong developmental factor in the central nervous system. With respect to its astrocytic activity, *Cxcl12* has also recently been implicated in cytotoxic pathways in astrocytes linked to NF-κB and cyclooxygenase-2 [73], suggesting a potentially complex functional role for this protein in the CNS.

While our data has assisted in generating a broader appreciation of the dynamic astrocyte responses to ambient oxygen tensions, we have also demonstrated that gross significant changes can be created, between potentially physiological-CNS oxygen tensions, and the cell culture standard of 20% O_2_. We repeatedly identified that progressive reductions in oxygen tension were correlated with concerted increases in the ribosomal component of the astrocyte transcriptome, suggesting that there may be profound global differences in the resultant protein expression profiles between the ambient oxygen tension employed in typical cell culture experimental paradigms. This may predict that physiological ligand responses may be significantly different between these conditions. Such distinctions may form the basis of many future investigations that could illuminate such potential proteomic shifts between culture conditions. In addition to this general aspect, significant and specific changes in the energy regulatory profile, response to inflammatory cytokines and immune modulators, the control of cytoskeletal dynamics, receptor tyrosine kinase activity, cancer/cell-cycle-related and cardiovascular signaling activity were observed in response to dynamic transitional changes in astrocyte oxygen environment. While we have attempted to comprehensively assess astrocyte responsivity to oxygen tensions it is however likely that even in primary cultures of rat cortical astrocytes there is astrocytic cell heterogeneity that may affect specific cellular responses. Hence extracted astrocytes that previously possessed a close proximity to the cerebral vasculature may display variable responses to O_2_ tension and transcriptional activity. In future investigations specific microdissection of vessel-proximal or vessel-distal astrocytes may further illuminate the intricacies of oxygen-dependent astrocyte functions. There may therefore be a complex interplay between oxygen tension gradients, created by physical juxtaposition of tissues with distinct metabolic activities, *e.g.* neurons and microvasculature, and control of astrocytic transcriptional functionality. For example, recent research has suggested that glial cells, neurons and microvascular endothelial cells could be considered to constitute a singular ‘neurovascular unit’. This tight functional association of tissues, sensitive to factors pre-disposing to pathology (*e.g.* hypoxia, Aβ release, inflammation) may constitute a system for stressor detection but may also be responsible for pathological “spreading effects” characteristic of progressive neurodegenerative disorders such as AD [74].

In addition to illuminating these potential functional aspects of astrocytic function, our current findings though may still bear an important message for *in vitro* culturing techniques, as it is clear that complex and coherent changes in multiple genomic and signaling systems are likely to occur and may impact any resultant findings from cells/tissue in such conditions.

## Supporting Information

Figure S1 **MSigDB PAGE collection analysis of oxygen tension-dependent gene transcription.** (**A**) Significantly regulated PAGE gene collections generated by the transcriptional dataset induced by 24 hour exposure to 1% O_2_ tension. (**B**) Significantly regulated PAGE gene collections generated by the transcriptional dataset induced by 24 hour exposure to 4% O_2_ tension. (**C**) Significantly regulated PAGE gene collections generated by the transcriptional dataset induced by 24 hour exposure to 9% O_2_ tension. The magnitude of the specific collection Z scores are indicated in the scale at the bottom of each histogram (A–C).
(TIF)

Figure S2 **Latent Semantic Indexing gene-interrogation term matrix for cluster** ***ONE*** **.** Each colored block represents a latent semantic indexing correlation score (≥0.1) for the specific gene-interrogation term pair in the matrix. The user-defined interrogation terms used were as follows: 1-*neurodegeneration*; 2-*Alzheimer's*; 3-*aging*; 4-*ischemia*; 5-*neuroprotective*; 6-*cognition*; 7-*hyperoxia*; 8-*hypoxia*; 9-*astrocyte*.
(TIF)

Figure S3 **Latent Semantic Indexing gene-interrogation term matrix for cluster** ***TWO*** **.** Each colored block represents a latent semantic indexing correlation score (≥0.1) for the specific gene-interrogation term pair in the matrix. The user-defined interrogation terms used were as follows: 1-*neurodegeneration*; 2-*Alzheimer's*; 3-*aging*; 4-*ischemia*; 5-*neuroprotective*; 6-*cognition*; 7-*hyperoxia*; 8-*hypoxia*; 9-*astrocyte*.
(TIF)

Figure S4 **Latent Semantic Indexing gene-interrogation term matrix for cluster** ***THREE*** **.** Each colored block represents a latent semantic indexing correlation score (≥0.1) for the specific gene-interrogation term pair in the matrix. The user-defined interrogation terms used were as follows: 1-*neurodegeneration*; 2-*Alzheimer's*; 3-*aging*; 4-*ischemia*; 5-*neuroprotective*; 6-*cognition*; 7-*hyperoxia*; 8-*hypoxia*; 9-*astrocyte*.
(TIF)

Figure S5 **Latent Semantic Indexing gene-interrogation term matrix for cluster** ***FOUR*** **.** Each colored block represents a latent semantic indexing correlation score (≥0.1) for the specific gene-interrogation term pair in the matrix. The user-defined interrogation terms used were as follows: 1-*neurodegeneration*; 2-*Alzheimer's*; 3-*aging*; 4-*ischemia*; 5-*neuroprotective*; 6-*cognition*; 7-*hyperoxia*; 8-*hypoxia*; 9-*astrocyte*.
(TIF)

Figure S6 **Functional network analysis of astrocyte group clusters** ***ONE*** **,** ***TWO*** **,** ***THREE*** **and** ***FOUR*** **.** The highest scoring functional interaction network, ranked by the greatest inclusion of genes identified both in the input dataset and those found in the functional predicted network. Network scores were calculated using Ingenuity Pathway Analysis version 8.5. the highest scoring networks for group cluster *ONE*, *TWO*, *THREE* and *FOUR* are depicted in panels (**A**), (**B**), (**C**) and (**D**) respectively.
(TIF)

Table S1 **Significantly regulated (p<0.05) genes in rat primary astrocytes exposed to 1% ambient O_2_ tension compared to 20% O_2_ tension.** Z ratios were calculated as described in Materials and Methods.
(DOC)

Table S2 **Significantly regulated (p<0.05) genes in rat primary astrocytes exposed to 4% ambient O_2_ tension compared to 20% O_2_ tension.** Z ratios were calculated as described in Materials and Methods.
(DOC)

Table S3 **Significantly regulated (p<0.05) genes in rat primary astrocytes exposed to 9% ambient O_2_ tension compared to 20% O_2_ tension.** Z ratios were calculated as described in Materials and Methods.
(DOC)

Table S4 **Venn diagram analysis output for significant gene regulation between 1, 4, 9% O_2_ tensions versus 20% O_2_.** Official gene symbols are employed to demonstrate the significantly regulated genes populating the Venn diagram intersections, A–G, depicted in Figure 2.
(DOC)

Table S5 **Significantly regulated transcripts common between 1% and 4% O_2_.** Official gene symbols are employed to demonstrate the significantly regulated genes populating the Venn diagram intersections D, depicted in Figure 2. Positive z ratios indicate upregulation compared to 20% O_2_ and negative z ratios indicate downregulation compared to 20% O_2_.
(DOC)

Table S6 **Significantly regulated transcripts common between 4% and 9% O_2_.** Official gene symbols are employed to demonstrate the significantly regulated genes populating the Venn diagram intersections F, depicted in Figure 2. Positive z ratios indicate upregulation compared to 20% O_2_ and negative z ratios indicate downregulation compared to 20% O_2_.
(DOC)

Table S7 **Significantly regulated transcripts common between 1% and 9% O_2_.** Official gene symbols are employed to demonstrate the significantly regulated genes populating the Venn diagram intersections E, depicted in Figure 2. Positive z ratios indicate upregulation compared to 20% O_2_ and negative z ratios indicate downregulation compared to 20% O_2_.
(DOC)

Table S8 **Significantly populated PAGE gene collections created with transcripts responding to 1% O_2_ tension compared to control 20% O_2_ condition.** Significantly-regulated genes were used to populate the specified MSigDB collections. ‘# genes in collection’ describes the total gene count of the specific MSigDB collection and the ‘# exp genes in collection’ describes the number of genes from the input experimental set that were able to significantly populate the specific MSigDB collection. The Z score is calculated based upon the cumulative z ratios of the respective genes from the experimental dataset that populated the specific MSigDB collection.
(DOC)

Table S9 **Significantly populated PAGE gene collections created with transcripts responding to 4% O_2_ tension compared to control 20% O_2_ condition.** Significantly-regulated genes were used to populate the specified MSigDB collections. ‘# genes in collection’ describes the total gene count of the specific MSigDB collection and the ‘# exp genes in collection’ describes the number of genes from the input experimental set that were able to significantly populate the specific MSigDB collection. The Z score is calculated based upon the cumulative z ratios of the respective genes from the experimental dataset that populated the specific MSigDB collection.
(DOC)

Table S10 **Significantly populated PAGE gene collections created with transcripts responding to 9% O_2_ tension compared to control 20% O_2_ condition.** Significantly-regulated genes were used to populate the specified MSigDB collections. ‘# genes in collection’ describes the total gene count of the specific MSigDB collection and the ‘# exp genes in collection’ describes the number of genes from the input experimental set that were able to significantly populate the specific MSigDB collection. The Z score is calculated based upon the cumulative z ratios of the respective genes from the experimental dataset that populated the specific MSigDB collection.
(DOC)

Table S11 **Venn diagram analysis output for significant PAGE gene collection population between 1, 4, 9% O_2_ tensions versus 20% O_2_.** PAGE gene collections populated by significantly regulated genes with a positive Z score are denoted in normal text while PAGE gene collections populated by significantly regulated genes with a negative Z score are denoted in italics. The PAGE collections are organized into the specific Venn diagram subsets, A–G, depicted in Figure 4.
(DOC)

Table S12 ***K-means*** **clusters for 1, 4, 9% O_2_ tension versus 20% control O_2_ tension.** The respective z ratios (z-r) for the specific oxygen tension versus 20% O_2_ for each transcript are displayed alongside the *k-means* generated cluster number.
(DOC)

Table S13 **Venn diagram analysis of canonical signaling pathway analysis for the four classified group clusters.** The four-way Venn diagram describes the relationships between the statistically significant canonical signaling pathways populated by the multiple transcripts contained in the four group clusters, *ONE*, *TWO*, *THREE*, *FOUR*. The intersection subsets, A to O, of significantly populated signaling pathways are depicted in Figure 7.
(DOC)

Table S14 **Physiological relevance of group clusters** ***ONE*** **-,** ***TWO*** **-,** ***THREE*** **- and** ***FOUR*** **-unique signaling pathways.** For each canonical signaling pathway significantly populated using the group cluster input genes a hybrid score was created that was the generated by multiplication of the negative log_10_ of the probability of enrichment of the genes in the respective signaling pathway with the gene enrichment ratio relative to a species-specific background geneset. For each significantly-populated signaling pathway the genes from the input dataset that generated the associated hybrid score are denoted.
(DOC)

Table S15 **Highest scoring gene networks present within group clusters** ***ONE*** **,** ***TWO*** **,** ***THREE*** **and** ***FOUR*** **.** For each group cluster geneset, functional interaction networks were created using Ingenuity Pathway Analysis (IPA). Network scores are created by the structural strength of the network in addition to the number of focus genes (genes present in network also present in the input experimental dataset: focus genes are indicated in bold). The IPA-predicted primary functions of the network are indicated for each of the four group clusters of genes.
(DOC)

Table S16 **Number of implicitly correlated genes, from group clusters** ***ONE, TWO, THREE and FOUR*** **, associated with each LSI interrogation term.** The number of genes from each group cluster dataset that demonstrated at least an implicit correlation (score ≥0.1) to the specific LSI interrogation term 1–9 is represented.
(DOC)

Table S17 **Mean Latent Semantic Indexing correlation scores for group clusters** ***ONE*** **,** ***TWO*** **,** ***THREE*** **and** ***FOUR*** **.** The mean LSI correlation score of all the genes that demonstrated an individual implicit correlation (score ≥0.1) to the specific LSI interrogation term 1–9.
(DOC)
