# Supplementary figures and images for: Multiple Oxygen Tension Environments Reveal Diverse Patterns of Transcriptional Regulation in Primary Astrocytes
Source: PLoS One. 2011 Jun 27;6(6):e21638. doi: 10.1371/journal.pone.0021638 (PMC3124552; doi:10.1371/journal.pone.0021638)

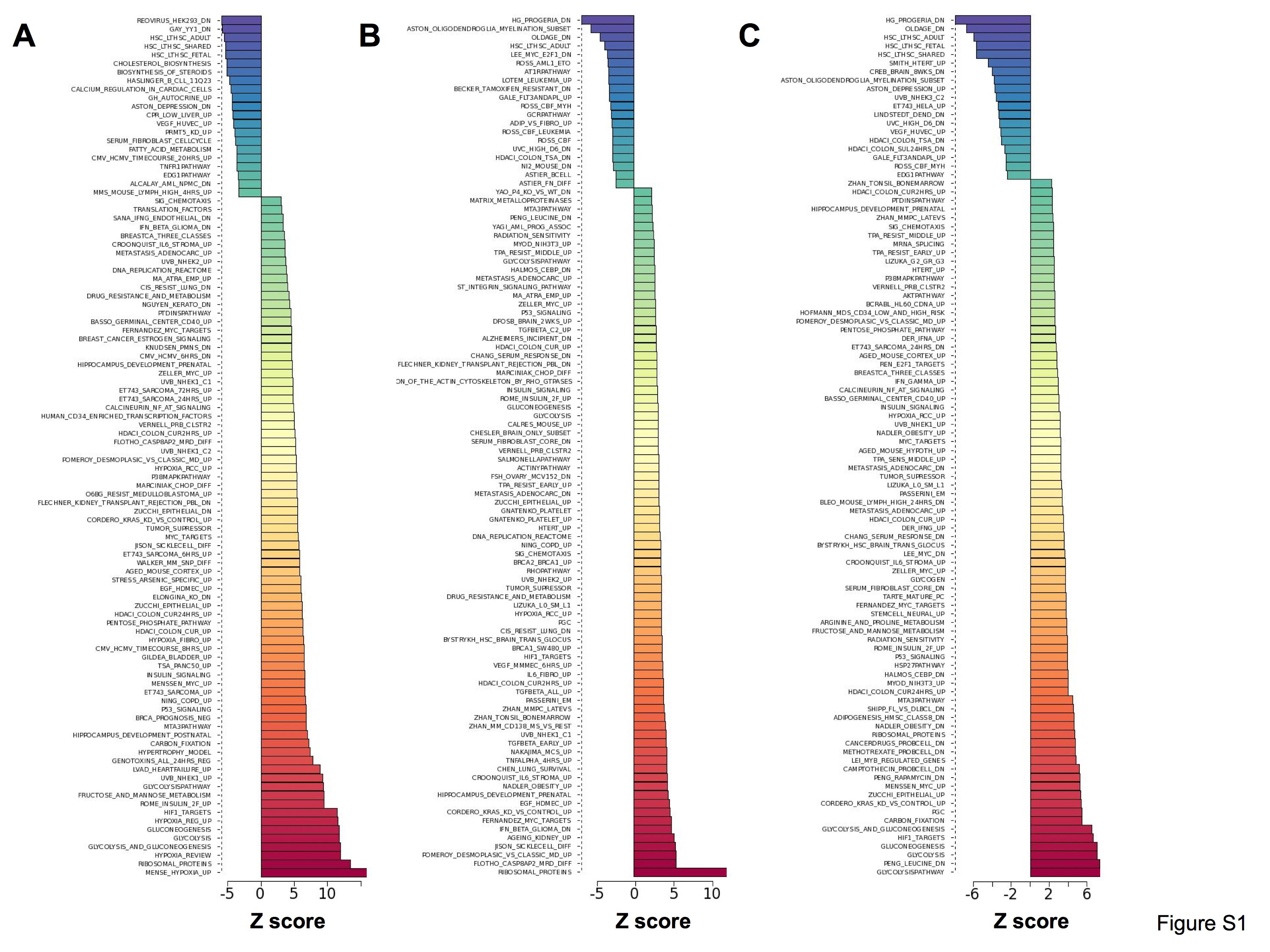

Supplement: Figure S1 — MSigDB PAGE collection analysis of oxygen tension-dependent gene transcription. (A) Significantly regulated PAGE gene collections generated by the transcriptional dataset induced by 24 hour exposure to 1% O2 tension. (B) Significantly regulated PAGE gene collections generated by the transcriptional dataset induced by 24 hour exposure to 4% O2 tension. (C) Significantly regulated PAGE gene collections generated by the transcriptional dataset induced by 24 hour exposure to 9% O2 tension. The magnitude of the specific collection Z scores are indicated in the scale at the bottom of each histogram (A–C). (TIF) [file pone.0021638.s001.tif]

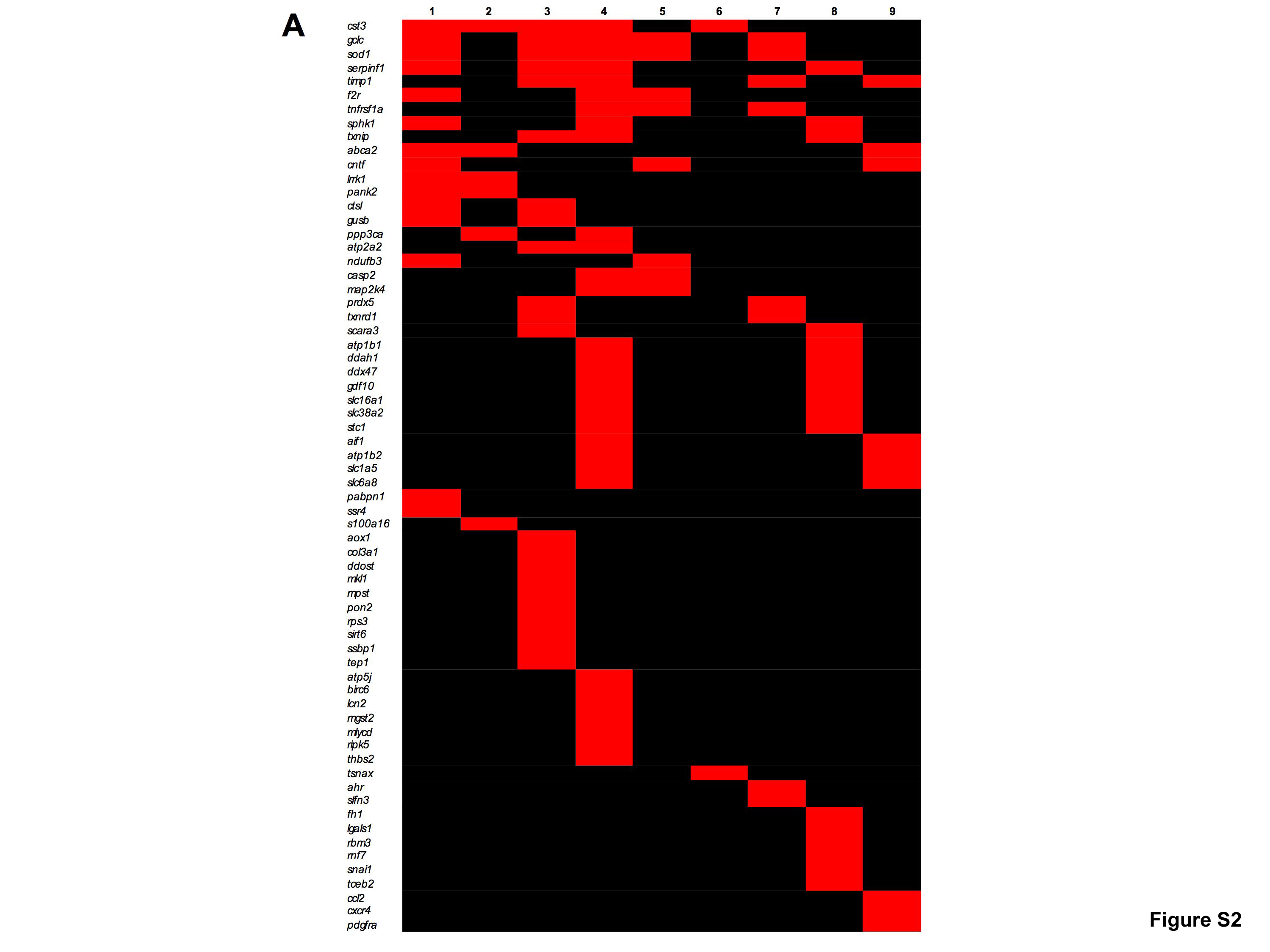

Supplement: Figure S2 — Latent Semantic Indexing gene-interrogation term matrix for cluster ONE . Each colored block represents a latent semantic indexing correlation score (≥0.1) for the specific gene-interrogation term pair in the matrix. The user-defined interrogation terms used were as follows: 1-neurodegeneration; 2-Alzheimer's; 3-aging; 4-ischemia; 5-neuroprotective; 6-cognition; 7-hyperoxia; 8-hypoxia; 9-astrocyte. (TIF) [file pone.0021638.s002.tif]

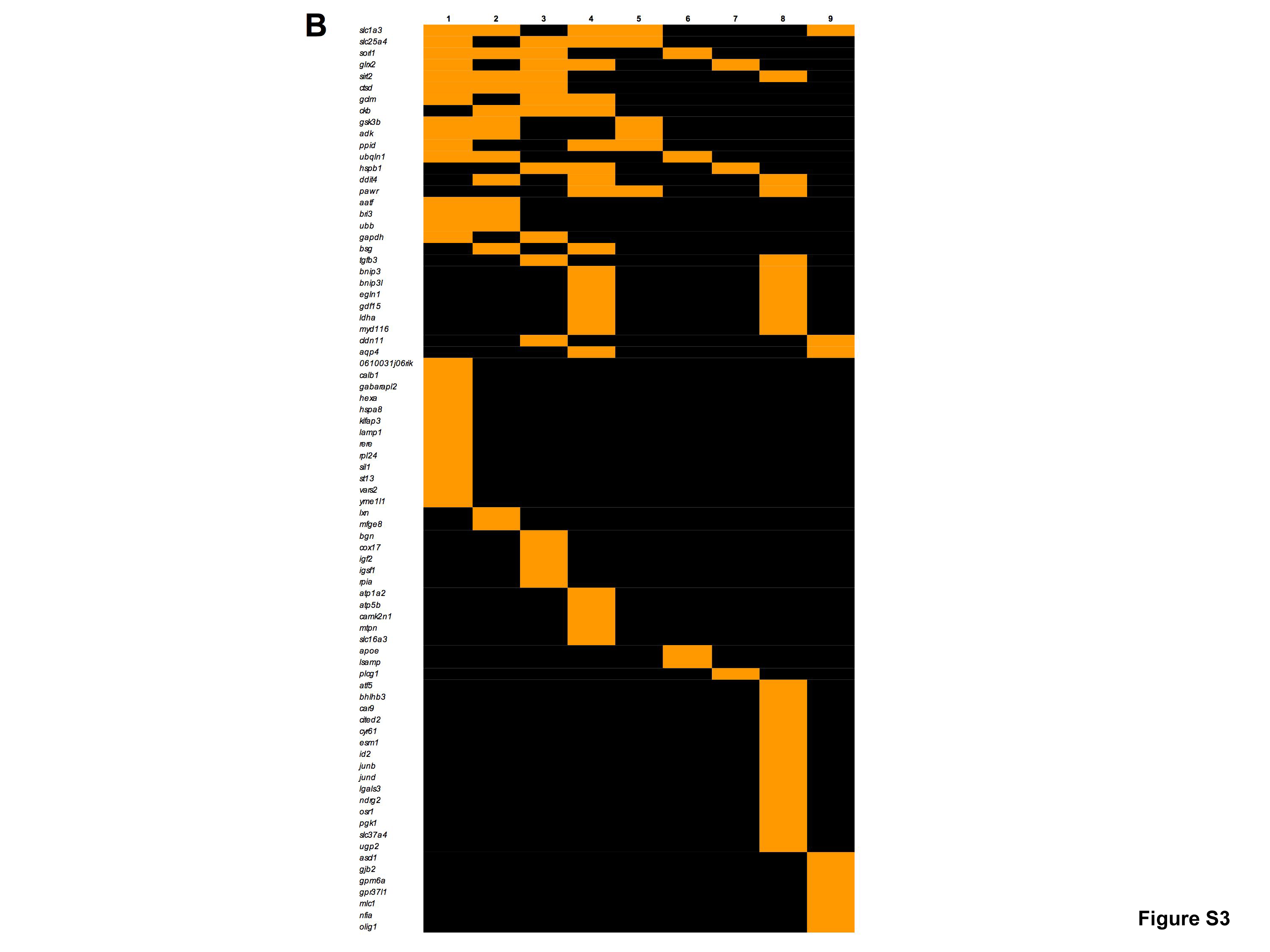

Supplement: Figure S3 — Latent Semantic Indexing gene-interrogation term matrix for cluster TWO . Each colored block represents a latent semantic indexing correlation score (≥0.1) for the specific gene-interrogation term pair in the matrix. The user-defined interrogation terms used were as follows: 1-neurodegeneration; 2-Alzheimer's; 3-aging; 4-ischemia; 5-neuroprotective; 6-cognition; 7-hyperoxia; 8-hypoxia; 9-astrocyte. (TIF) [file pone.0021638.s003.tif]

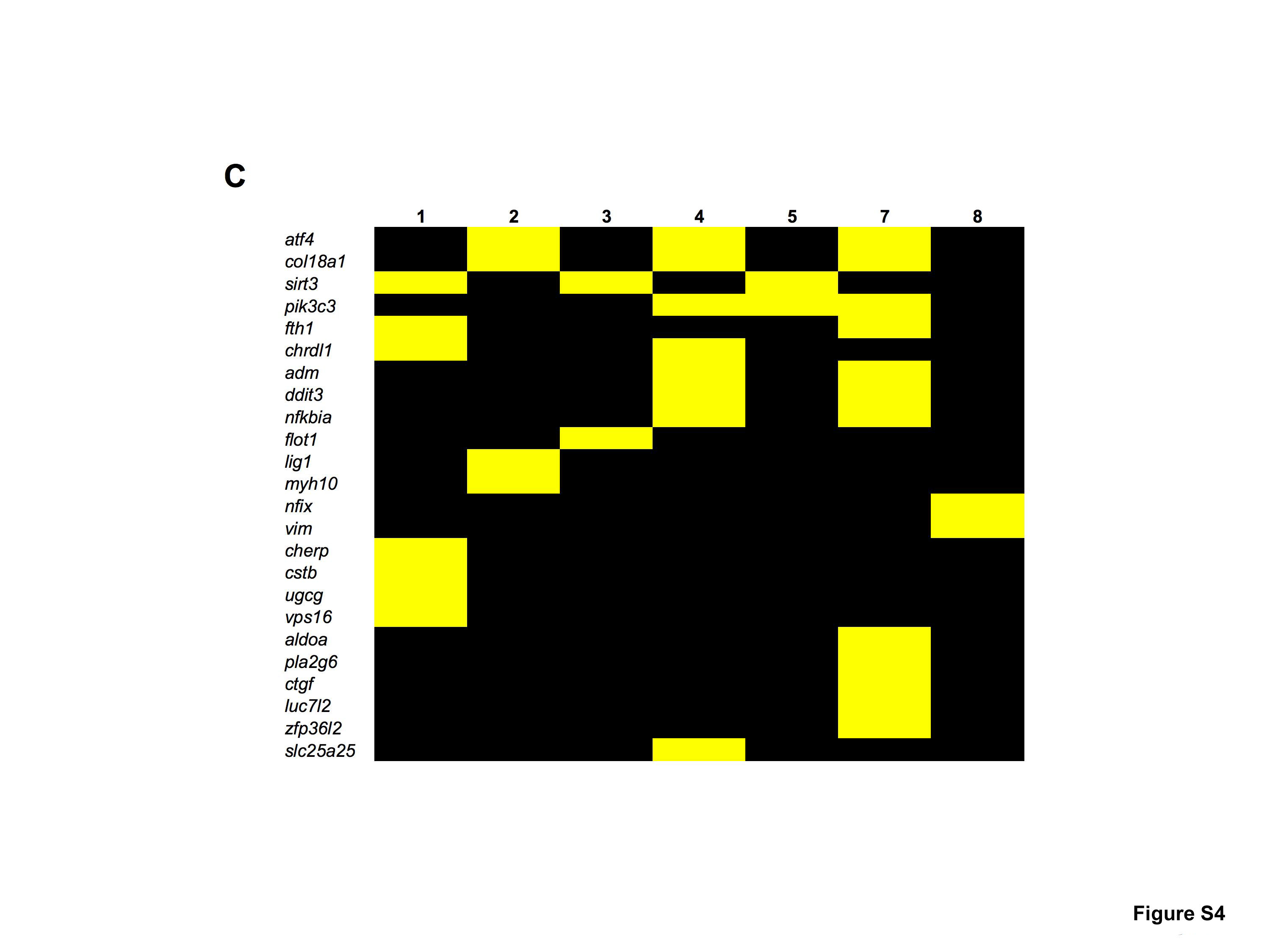

Supplement: Figure S4 — Latent Semantic Indexing gene-interrogation term matrix for cluster THREE . Each colored block represents a latent semantic indexing correlation score (≥0.1) for the specific gene-interrogation term pair in the matrix. The user-defined interrogation terms used were as follows: 1-neurodegeneration; 2-Alzheimer's; 3-aging; 4-ischemia; 5-neuroprotective; 6-cognition; 7-hyperoxia; 8-hypoxia; 9-astrocyte. (TIF) [file pone.0021638.s004.tif]

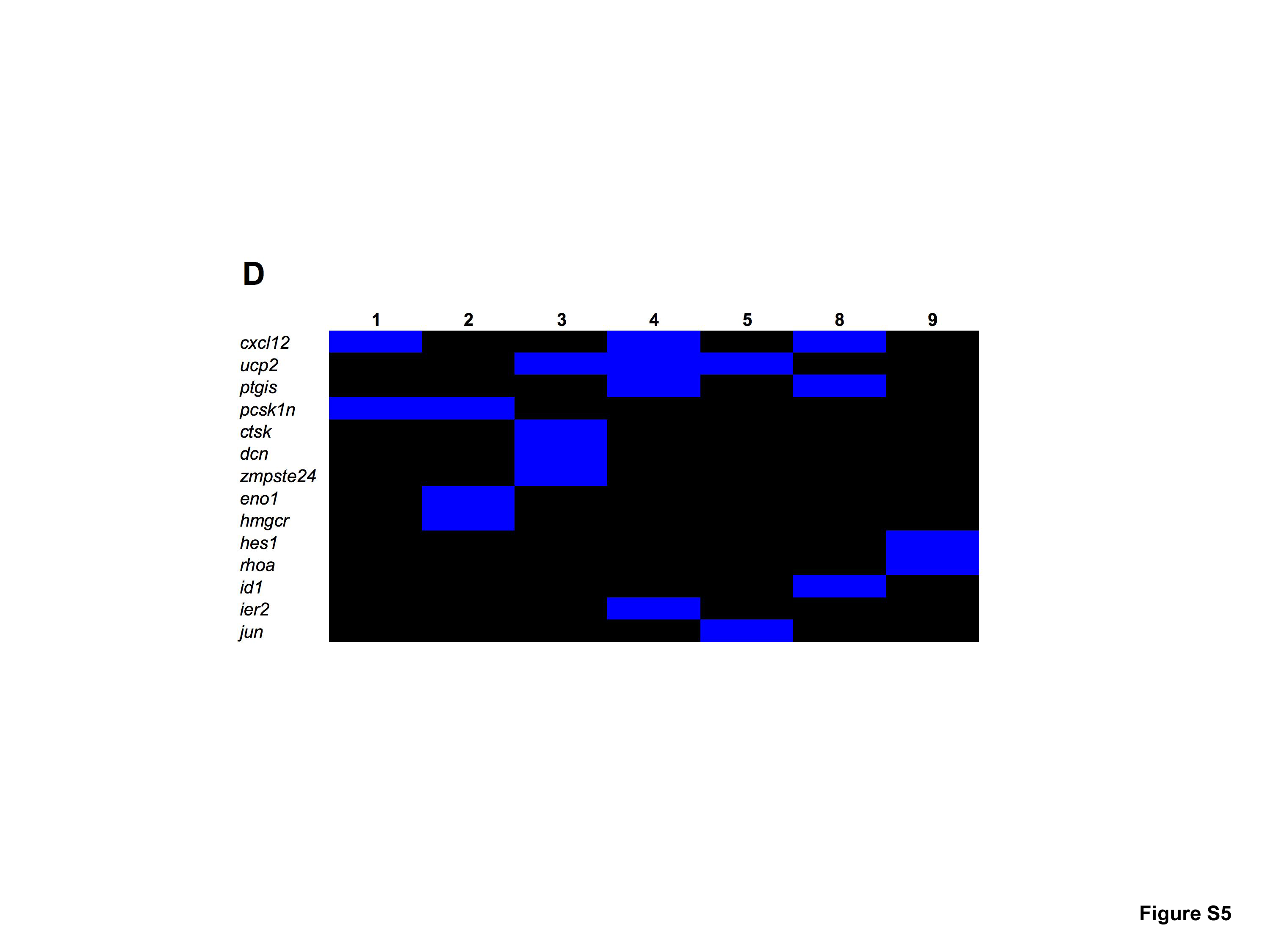

Supplement: Figure S5 — Latent Semantic Indexing gene-interrogation term matrix for cluster FOUR . Each colored block represents a latent semantic indexing correlation score (≥0.1) for the specific gene-interrogation term pair in the matrix. The user-defined interrogation terms used were as follows: 1-neurodegeneration; 2-Alzheimer's; 3-aging; 4-ischemia; 5-neuroprotective; 6-cognition; 7-hyperoxia; 8-hypoxia; 9-astrocyte. (TIF) [file pone.0021638.s005.tif]

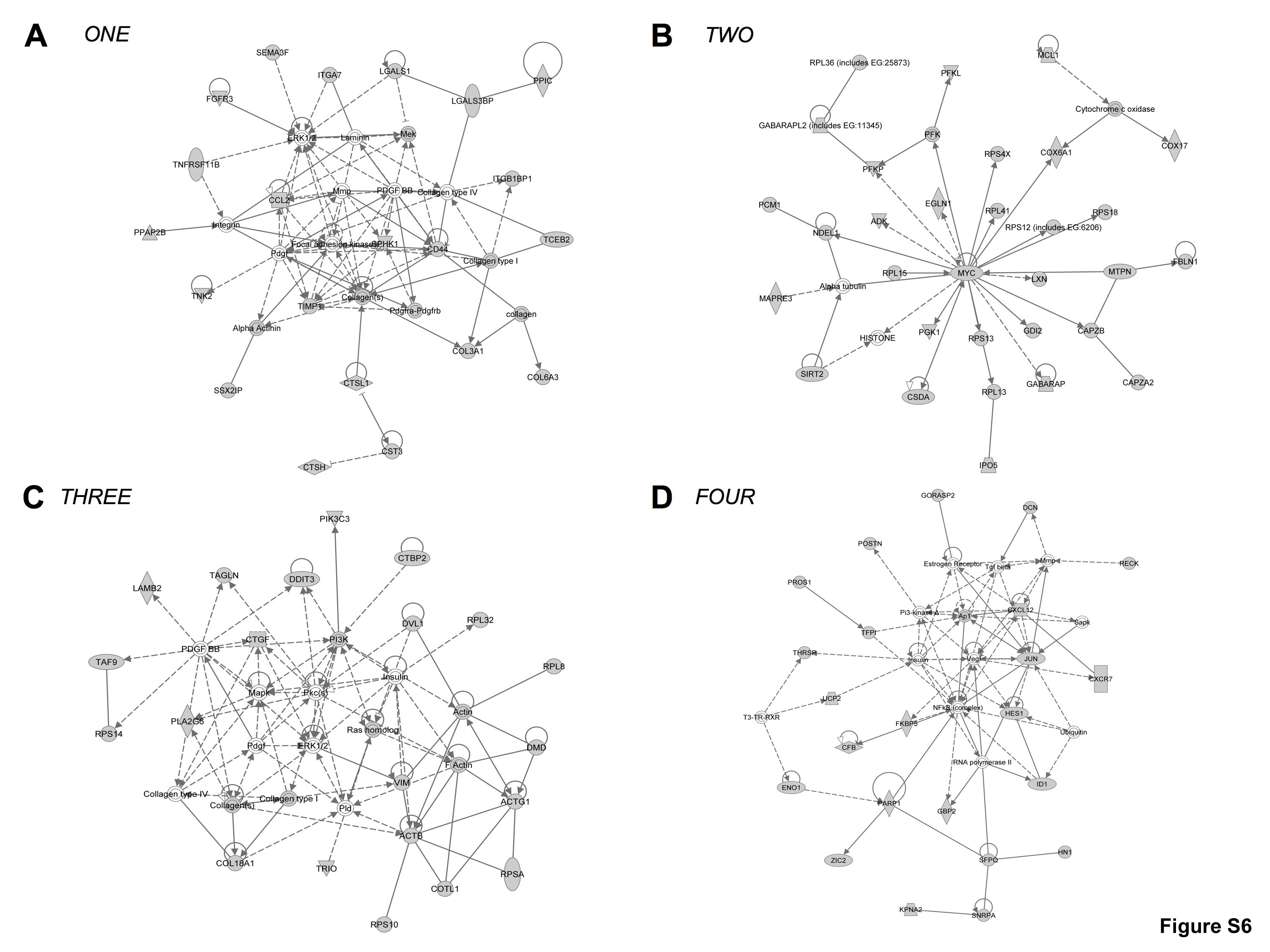

Supplement: Figure S6 — Functional network analysis of astrocyte group clusters ONE , TWO , THREE and FOUR . The highest scoring functional interaction network, ranked by the greatest inclusion of genes identified both in the input dataset and those found in the functional predicted network. Network scores were calculated using Ingenuity Pathway Analysis version 8.5. the highest scoring networks for group cluster ONE, TWO, THREE and FOUR are depicted in panels (A), (B), (C) and (D) respectively. (TIF) [file pone.0021638.s006.tif]
