# Supplementary material for: Multiple Oxygen Tension Environments Reveal Diverse Patterns of Transcriptional Regulation in Primary Astrocytes
Source: PLoS One. 2011 Jun 27;6(6):e21638. doi: 10.1371/journal.pone.0021638 (PMC3124552; doi:10.1371/journal.pone.0021638)
Supplement: Table S1 — Significantly regulated (p<0.05) genes in rat primary astrocytes exposed to 1% ambient O2 tension compared to 20% O2 tension. Z ratios were calculated as described in Materials and Methods. (DOC) [file pone.0021638.s007.doc]

**Table S1. Significantly regulated (p<0.05) genes in rat primary astrocytes exposed to 1% ambient O_2_ tension compared to 20% O_2_ tension.** Z ratios were calculated as described in Materials and Methods.

| **Symbol** | **Gene Definition** | **z ratio** |
| --- | --- | --- |
| Acta1 | Rattus norvegicus actin, alpha 1, skeletal muscle | 8.58 |
| Bhlhb2 | Rattus norvegicus basic helix-loop-helix domain containing, class B2 | 6.73 |
| Vegfa | Rattus norvegicus vascular endothelial growth factor A | 6.54 |
| Hk2 | Rattus norvegicus hexokinase 2 | 6.49 |
| Pim1 | Rattus norvegicus proviral integration site 1 | 6.45 |
| Ptp4a1 | Rattus norvegicus protein tyrosine phosphatase 4a1 | 6.36 |
| Dusp8 | Rattus norvegicus dual specificity phosphatase 8 | 5.94 |
| Hmox1 | Rattus norvegicus heme oxygenase | 5.9 |
| Ftl1 | Rattus norvegicus ferritin light chain 1 | 5.41 |
| LOC499244 | Rattus norvegicus similar to ferritin light chain | 5.37 |
| LOC501644 | Rattus norvegicus similar to Ferritin light chain 2 | 5.34 |
| LOC302402 | Rattus norvegicus similar to hypoxia induced gene 1 | 5.25 |
| Gadd45g | Rattus norvegicus growth arrest and DNA-damage-inducible 45 gamma | 5.18 |
| G0s2 | Rattus norvegicus G0/G1 switch gene 2 | 5.11 |
| LOC500804 | Rattus norvegicus similar to Ferritin light chain | 5.05 |
| Bnip3 | Rattus norvegicus BCL2/adenovirus E1B 19 kDa-interacting protein 3 | 5 |
| Timp3 | Rattus norvegicus tissue inhibitor of metalloproteinase 3 | 4.94 |
| Ak3l1 | Rattus norvegicus adenylate kinase 3-like 1 | 4.9 |
| Insig1 | Rattus norvegicus insulin induced gene 1 | 4.87 |
| Nr4a3 | Rattus norvegicus nuclear receptor subfamily 4, group A, member 3 | 4.76 |
| LOC500965 | Rattus norvegicus similar to L-lactate dehydrogenase A chain | 4.75 |
| Cebpb | Rattus norvegicus CCAAT/enhancer binding protein | 4.69 |
| LOC501140 | Rattus norvegicus similar to BCL2/adenovirus E1B 19 kDa-interacting protein 3 | 4.67 |
| Pgk1 | Rattus norvegicus phosphoglycerate kinase 1 | 4.67 |
| Tpi1 | Rattus norvegicus triosephosphate isomerase 1 | 4.62 |
| LOC498731 | Rattus norvegicus similar to Tpi1 protein | 4.53 |
| Nr1d1 | Rattus norvegicus nuclear receptor subfamily 1, group D, member 1 | 4.5 |
| Hspb1 | Rattus norvegicus heat shock 27kDa protein 1 | 4.47 |
| Eno2 | Rattus norvegicus enolase 2, gamma | 4.44 |
| Mif | Rattus norvegicus macrophage migration inhibitory factor | 4.36 |
| Aldoa | Rattus norvegicus aldolase A | 4.35 |
| Fth1 | Rattus norvegicus ferritin, heavy polypeptide 1 | 4.34 |
| LOC500959 | Rattus norvegicus similar to triosephosphate isomerase | 4.29 |
| Atf4 | Rattus norvegicus activating transcription factor 4 | 4.23 |
| LOC307731 | Rattus norvegicus similar to L-lactate dehydrogenase A chain | 4.23 |
| Ddit3 | Rattus norvegicus DNA-damage inducible transcript 3 | 4.21 |
| Egln1 | Rattus norvegicus EGL nine homolog 1 | 4.2 |
| LOC363377 | Rattus norvegicus similar to RIKEN cDNA 2410116I05 | 4.2 |
| Ldha | Rattus norvegicus lactate dehydrogenase A | 4.16 |
| P4ha1 | Rattus norvegicus procollagen-proline, 2-oxoglutarate 4-dioxygenase | 4.11 |
| LOC500983 | Rattus norvegicus similar to glyceraldehyde-3-phosphate dehydrogenase | 4.08 |
| LOC500271 | Rattus norvegicus similar to macrophage migration inhibitory factor | 4.05 |
| LOC498099 | Rattus norvegicus similar to glyceraldehyde-3-phosphate dehydrogenase | 4.05 |
| Mt1a | Rattus norvegicus Metallothionein | 4.02 |
| Arbp | Rattus norvegicus acidic ribosomal phosphoprotein P0 | 4.02 |
| LOC291715 | Rattus norvegicus similar to L-lactate dehydrogenase A chain | 3.98 |
| Vim | Rattus norvegicus vimentin | 3.98 |
| Pdk1 | Rattus norvegicus pyruvate dehydrogenase kinase 1 | 3.97 |
| Pygl | Rattus norvegicus liver glycogen phosphorylase | 3.96 |
| Ctgf | Rattus norvegicus connective tissue growth factor | 3.91 |
| Pbef1 | Rattus norvegicus pre-B-cell colony enhancing factor 1 | 3.89 |
| LOC364048 | Rattus norvegicus similar to macrophage migration inhibitory factor | 3.87 |
| Atf3 | Rattus norvegicus activating transcription factor 3 | 3.84 |
| Ppp1r3c | Rattus norvegicus protein phosphatase 1, regulatory | 3.83 |
| Gapd | Rattus norvegicus glyceraldehyde-3-phosphate dehydrogenase | 3.81 |
| Slc16a3 | Rattus norvegicus monocarboxylate transporter | 3.78 |
| LOC497803 | Rattus norvegicus hypothetical gene supported by NM_178095 | 3.78 |
| Gdf15 | Rattus norvegicus growth differentiation factor 15 | 3.78 |
| LOC500506 | Rattus norvegicus similar to glyceraldehyde-3-phosphate dehydrogenase | 3.77 |
| Hig1 | Rattus norvegicus hypoxia induced gene 1 | 3.71 |
| LOC295452 | Rattus norvegicus similar to Glyceraldehyde-3-phosphate dehydrogenase | 3.67 |
| Rragd | Rattus norvegicus Ras-related GTP binding D | 3.66 |
| LOC290634 | Rattus norvegicus similar to Glyceraldehyde-3-phosphate dehydrogenase | 3.65 |
| LOC366205 | Rattus norvegicus similar to fibronectin leucine rich transmembrane protein 3 | 3.63 |
| LOC497684 | Rattus norvegicus hypothetical gene supported by NM_017314 | 3.63 |
| LOC292656 | Rattus norvegicus similar to Macrophage migration inhibitory factor | 3.53 |
| LOC499433 | Rattus norvegicus similar to glyceraldehyde-3-phosphate dehydrogenase | 3.53 |
| LOC499178 | Rattus norvegicus LOC499178 | 3.52 |
| Gadd45b | Rattus norvegicus growth arrest and DNA-damage-inducible 45 beta | 3.5 |
| Actg | Rattus norvegicus actin, gamma, cytoplasmic | 3.49 |
| Rere | Rattus norvegicus arginine-glutamic acid dipeptide | 3.46 |
| Tagln | Rattus norvegicus transgelin | 3.4 |
| LOC365954 | Rattus norvegicus similar to glyceraldehyde-3-phosphate dehydrogenase | 3.39 |
| LOC290704 | Rattus norvegicus similar to palladin | 3.39 |
| Actb | Rattus norvegicus actin, beta | 3.39 |
| LOC295423 | Rattus norvegicus similar to glyceraldehyde-3-phosphate dehydrogenase | 3.38 |
| Cryab | Rattus norvegicus crystallin, alpha B | 3.38 |
| LOC364848 | Rattus norvegicus similar to Glyceraldehyde-3-phosphate dehydrogenase | 3.38 |
| LOC499201 | Rattus norvegicus hypothetical gene supported by BC061525 | 3.34 |
| Uap1l1 | Rattus norvegicus UDP-N-acteylglucosamine pyrophosphorylase 1-like 1 | 3.34 |
| Mak3 | Rattus norvegicus Mak3 homolog | 3.33 |
| Slc5a3 | Rattus norvegicus solute carrier family 5 | 3.33 |
| Gpi | Rattus norvegicus glucose phosphate isomerase | 3.32 |
| Cpeb4 | Rattus norvegicus cytoplasmic polyadenylation element binding protein 4 | 3.29 |
| LOC498618 | Rattus norvegicus similar to glyceraldehyde-3-phosphate dehydrogenase | 3.27 |
| Nfil3 | Rattus norvegicus nuclear factor, interleukin 3 regulated | 3.25 |
| Pfkl | Rattus norvegicus phosphofructokinase, liver, B-type | 3.24 |
| LOC498881 | Rattus norvegicus similar to glyceraldehyde-3-phosphate dehydrogenase | 3.21 |
| Dyrk2 | Rattus norvegicus dual-specificity tyrosine- | 3.18 |
| LOC497927 | Rattus norvegicus similar to Phosphoglycerate mutase 1 | 3.17 |
| LOC498019 | Rattus norvegicus similar to glyceraldehyde-3-phosphate dehydrogenase | 3.12 |
| LOC500104 | Rattus norvegicus similar to Glyceraldehyde-3-phosphate dehydrogenase | 3.08 |
| Cyr61 | Rattus norvegicus cysteine rich protein 61 | 2.99 |
| LOC498038 | Rattus norvegicus similar to colon carcinoma related protein | 2.98 |
| LOC502770 | Rattus norvegicus similar to glyceraldehyde-3-phosphate dehydrogenase | 2.96 |
| Xpo1 | Rattus norvegicus exportin 1, CRM1 homolog | 2.95 |
| Sv2b | Rattus norvegicus synaptic vesicle glycoprotein 2b | 2.92 |
| Flcn | Rattus norvegicus folliculin | 2.9 |
| Eef1a1 | Rattus norvegicus eukaryotic translation elongation factor 1 alpha 1 | 2.88 |
| Copeb | Rattus norvegicus core promoter element binding protein | 2.85 |
| Adm | Rattus norvegicus adrenomedullin | 2.84 |
| LOC298169 | Rattus norvegicus similar to actin alpha 1 skeletal muscle protein | 2.81 |
| LOC499794 | Rattus norvegicus similar to ribosomal protein L10 | 2.76 |
| Car9 | Rattus norvegicus carbonic anhydrase 9 | 2.73 |
| Maff | Rattus norvegicus v-maf musculoaponeurotic fibrosarcoma oncogene family, protein F | 2.68 |
| LOC501206 | Rattus norvegicus similar to 60S ribosomal protein L5 | 2.68 |
| Lamr1 | Rattus norvegicus laminin receptor 1 | 2.65 |
| LOC502063 | Rattus norvegicus LOC502063 | 2.6 |
| Rpl41 | Rattus norvegicus ribosomal protein L41 | 2.6 |
| Pfkp | Rattus norvegicus phosphofructokinase, platelet | 2.59 |
| LOC316842 | Rattus norvegicus similar to cDNA sequence BC019776 | 2.59 |
| Hspca | Rattus norvegicus heat shock protein 1, alpha | 2.57 |
| MGC94288 | Rattus norvegicus similar to 4632419K20Rik protein | 2.56 |
| LOC361026 | Rattus norvegicus similar to 60S ribosomal protein L7a | 2.56 |
| Pawr | Rattus norvegicus PRKC, apoptosis, WT1, regulator | 2.55 |
| LOC310395 | Rattus norvegicus similar to carbon catabolite repression 4 protein homolog | 2.53 |
| Slc38a1 | Rattus norvegicus solute carrier family 38, member 1 | 2.52 |
| Rps10 | Rattus norvegicus ribosomal protein S10 | 2.52 |
| Wsb1 | Rattus norvegicus WD repeat and SOCS box-containing 1 | 2.51 |
| Dctn4 | Rattus norvegicus dynactin 4 | 2.5 |
| osr1 | Rattus norvegicus oxidative stress responsive 1 | 2.48 |
| LOC498143 | Rattus norvegicus similar to ribosomal protein L15 | 2.47 |
| LOC306115 | Rattus norvegicus similar to glyceraldehyde-3-phosphate dehydrogenase | 2.46 |
| Rps18 | Rattus norvegicus ribosomal protein S18 | 2.45 |
| LOC500547 | Rattus norvegicus similar to 60S ribosomal protein L37a | 2.44 |
| Mgea5 | Rattus norvegicus meningioma expressed antigen 5 | 2.43 |
| Prss15 | Rattus norvegicus protease, serine, 15 | 2.41 |
| LOC288065 | Rattus norvegicus hypothetical LOC288065 | 2.4 |
| LOC309161 | Rattus norvegicus similar to Delta-interacting protein A | 2.4 |
| LOC304035 | Rattus norvegicus similar to 60S ribosomal protein L7a | 2.4 |
| Luc7l | Rattus norvegicus LUC7-like | 2.39 |
| LOC299622 | Rattus norvegicus similar to glyceraldehyde-3-phosphate dehydrogenase | 2.39 |
| Hspcal3 | Rattus norvegicus heat shock 90kDa protein 1, alpha-like 3 | 2.39 |
| Slc3a2 | Rattus norvegicus solute carrier family 3 | 2.39 |
| Hspbap1 | Rattus norvegicus Hspb associated protein 1 | 2.38 |
| Bhlhb3 | Rattus norvegicus basic helix-loop-helix domain containing, class B3 | 2.38 |
| Id2 | Rattus norvegicus Inhibitor of DNA binding 2, dominant negative helix-loop-helix protein | 2.36 |
| Rpl29 | Rattus norvegicus ribosomal protein L29 | 2.34 |
| Lgals3 | Rattus norvegicus lectin, galactose binding, soluble 3 | 2.33 |
| LOC500660 | Rattus norvegicus similar to ribosomal protein L21 | 2.31 |
| Ugp2 | Rattus norvegicus UDP-glucose pyrophosphorylase 2 | 2.3 |
| LOC499523 | Rattus norvegicus similar to 60S ribosomal protein L23a | 2.3 |
| Fn1 | Rattus norvegicus fibronectin 1 | 2.3 |
| Rpl3 | Rattus norvegicus ribosomal protein L3 | 2.28 |
| LOC499171 | Rattus norvegicus similar to BTEB3 protein | 2.27 |
| LOC361797 | Rattus norvegicus hypothetical LOC361797 | 2.26 |
| RGD1307599 | Rattus norvegicus similar to Mitogen-inducible gene 6 protein homolog | 2.26 |
| Rps27 | Rattus norvegicus ribosomal protein S27 | 2.26 |
| LOC293642 | Rattus norvegicus similar to ribosomal protein L21 | 2.26 |
| LOC499906 | Rattus norvegicus similar to Eukaryotic translation initiation factor 1 | 2.25 |
| Rnpc2 | Rattus norvegicus RNA-binding region | 2.25 |
| LOC500242 | Rattus norvegicus similar to Poly | 2.25 |
| Rpl21 | Rattus norvegicus ribosomal protein L21 | 2.24 |
| Rplp1 | Rattus norvegicus ribosomal protein, large, P1 | 2.24 |
| Ubb | Rattus norvegicus polyubiquitin | 2.24 |
| Rpl27a | Rattus norvegicus ribosomal protein L27a | 2.23 |
| Tpt1 | Rattus norvegicus tumor protein, translationally-controlled 1 | 2.23 |
| Ddr1 | Rattus norvegicus discoidin domain receptor family, member 1 | 2.22 |
| Mrpl45 | Rattus norvegicus mitochondrial ribosomal protein L45 | 2.22 |
| LOC500669 | Rattus norvegicus similar to 60S ribosomal protein L7a | 2.22 |
| Cxcl10 | Rattus norvegicus chemokine | 2.21 |
| LOC310365 | Rattus norvegicus similar to ribosomal protein L21 | 2.21 |
| C3orf6h | Rattus norvegicus putative C3orf6 protein homolog | 2.2 |
| LOC365699 | Rattus norvegicus similar to hypothetical protein FLJ30596 | 2.19 |
| Rpl14 | Rattus norvegicus ribosomal protein L14 | 2.19 |
| Rps14 | Rattus norvegicus ribosomal protein S14 | 2.19 |
| Bri3 | Rattus norvegicus brain protein I3 | 2.17 |
| Pabpc1 | Rattus norvegicus poly | 2.17 |
| LOC296870 | Rattus norvegicus similar to ribosomal protein L34 | 2.17 |
| LOC499845 | Rattus norvegicus similar to ribosomal protein L21 | 2.17 |
| Aatf | Rattus norvegicus apoptosis antagonizing transcription factor | 2.16 |
| Dpp7 | Rattus norvegicus dipeptidylpeptidase 7 | 2.16 |
| Umpk | Rattus norvegicus uridine monophosphate kinase | 2.15 |
| LOC500885 | Rattus norvegicus similar to 40S ribosomal protein S19 | 2.15 |
| Rora | Rattus norvegicus RAR-related orphan receptor alpha | 2.14 |
| Myd116 | Rattus norvegicus myeloid differentiation primary response gene 116 | 2.14 |
| Hnrpdl | Rattus norvegicus heterogeneous nuclear ribonucleoprotein D-like | 2.13 |
| Rpl32 | Rattus norvegicus ribosomal protein L32 | 2.13 |
| Dre1 | Rattus norvegicus Dre1 protein | 2.11 |
| Cited2 | Rattus norvegicus Cbp/p300-interacting transactivator, with Glu/Asp-rich carboxy-terminal domain, 2 | 2.11 |
| LOC295439 | Rattus norvegicus similar to ribosomal protein L21 | 2.11 |
| LOC291308 | Rattus norvegicus similar to ribosomal protein L21 | 2.11 |
| Rps27a | Rattus norvegicus ribosomal protein S27a | 2.11 |
| LOC500914 | Rattus norvegicus similar to basic transcription factor 3 | 2.1 |
| LOC503284 | Rattus norvegicus similar to ribosomal protein L19 | 2.1 |
| Eno1 | Rattus norvegicus enolase 1, alpha | 2.09 |
| LOC499423 | Rattus norvegicus similar to pyruvate kinase | 2.09 |
| Rps4x | Rattus norvegicus ribosomal protein S4, X-linked | 2.09 |
| LOC498998 | Rattus norvegicus similar to 60S ribosomal protein L26 | 2.08 |
| LOC298785 | Rattus norvegicus similar to ribosomal protein S26 | 2.08 |
| Tbc1d15 | Rattus norvegicus TBC1 domain family, member 15 | 2.07 |
| LOC310585 | Rattus norvegicus similar to Eno1 protein | 2.07 |
| Naca | Rattus norvegicus nascent-polypeptide-associated complex alpha polypeptide | 2.07 |
| Rpl19 | Rattus norvegicus ribosomal protein L19 | 2.07 |
| LOC499803 | Rattus norvegicus similar to 40S ribosomal protein S3 | 2.07 |
| Eif4ebp1 | Rattus norvegicus eukaryotic translation initiation factor 4E binding protein 1 | 2.06 |
| Rpl37a | Rattus norvegicus ribosomal protein L37a | 2.06 |
| Rps11 | Rattus norvegicus ribosomal protein S11 | 2.05 |
| Gnl3 | Rattus norvegicus guanine nucleotide binding protein-like 3 | 2.04 |
| LOC301068 | Rattus norvegicus similar to translation factor sui1 homolog | 2.04 |
| Vars2 | Rattus norvegicus valyl-tRNA synthetase 2 | 2.03 |
| LOC302445 | Rattus norvegicus similar to 60S ribosomal protein L32 | 2.03 |
| Ddit4 | Rattus norvegicus DNA-damage-inducible transcript 4 | 2.02 |
| LOC500859 | Rattus norvegicus similar to 60S ribosomal protein L7a | 2.01 |
| LOC303554 | Rattus norvegicus similar to Nbr1 | 2 |
| Nfe2l2 | Rattus norvegicus nuclear factor, erythroid derived 2, like 2 | 2 |
| LOC366193 | Rattus norvegicus similar to 40S ribosomal protein S3a | 2 |
| Icam1 | Rattus norvegicus intercellular adhesion molecule 1 | 1.99 |
| LOC361115 | Rattus norvegicus similar to tumor protein, translationally-controlled 1 | 1.99 |
| Rpl9 | Rattus norvegicus ribosomal protein L9 | 1.98 |
| Cstb | Rattus norvegicus cystatin B | 1.97 |
| Rpl4 | Rattus norvegicus ribosomal protein L4 | 1.97 |
| Rps15a | Rattus norvegicus ribosomal protein S15a | 1.97 |
| Ptgis | Rattus norvegicus prostaglandin I2 | 1.96 |
| Ndel1 | Rattus norvegicus nudE nuclear distribution gene E homolog like 1 | 1.96 |
| LOC293860 | Rattus norvegicus similar to Filamin A | 1.96 |
| Rpl10 | Rattus norvegicus ribosomal protein L10 | 1.96 |
| Rps8 | Rattus norvegicus ribosomal protein S8 | 1.95 |
| LOC362181 | Rattus norvegicus similar to Ac2-210 | 1.94 |
| LOC502629 | Rattus norvegicus similar to 60S ribosomal protein L9 | 1.93 |
| Ewsr1 | Rattus norvegicus Ewing sarcoma breakpoint region 1 | 1.92 |
| LOC295472 | Rattus norvegicus similar to ribosomal protein L21 | 1.92 |
| Tiparp | Rattus norvegicus TCDD-inducible poly | 1.91 |
| Tpm1 | Rattus norvegicus tropomyosin 1, alpha | 1.91 |
| LOC365753 | Rattus norvegicus similar to Spindlin | 1.9 |
| Hspa8 | Rattus norvegicus heat shock protein 8 | 1.9 |
| LOC500929 | Rattus norvegicus similar to Tubulin alpha-2 chain | 1.9 |
| Klf5 | Rattus norvegicus Kruppel-like factor 5 | 1.89 |
| Rpl36 | Rattus norvegicus ribosomal protein L36 | 1.89 |
| LOC314556 | Rattus norvegicus similar to ribosomal protein S18 | 1.89 |
| LOC502302 | Rattus norvegicus similar to 40S ribosomal protein S19 | 1.89 |
| LOC294748 | Rattus norvegicus similar to Chain , Human Translation Initiation Factor Eif1, Nmr, 29 Structures | 1.87 |
| LOC289715 | Rattus norvegicus similar to ribosomal protein L37 | 1.87 |
| Wdfy1 | Rattus norvegicus WD repeat and FYVE domain containing 1 | 1.86 |
| P4hb | Rattus norvegicus prolyl 4-hydroxylase, beta polypeptide | 1.86 |
| Rpl26 | Rattus norvegicus ribosomal protein L26 | 1.86 |
| Cmkor1 | Rattus norvegicus chemokine orphan receptor 1 | 1.85 |
| Vgll4 | Rattus norvegicus vestigial like 4 | 1.85 |
| LOC361061 | Rattus norvegicus hypothetical LOC361061 | 1.85 |
| Ppp2r2a | Rattus norvegicus protein phosphatase 2 | 1.84 |
| MGC94937 | Rattus norvegicus similar to RIKEN cDNA 1110020M19 | 1.83 |
| LOC499690 | Rattus norvegicus similar to RIKEN cDNA 2010200O16 | 1.83 |
| St13 | Rattus norvegicus suppression of tumorigenicity 13 | 1.83 |
| Cd63 | Rattus norvegicus CD63 antigen | 1.82 |
| LOC312363 | Rattus norvegicus similar to 60S ribosomal protein L12 | 1.82 |
| LOC303815 | Rattus norvegicus similar to 60S ribosomal protein L12 | 1.82 |
| Trit1 | Rattus norvegicus tRNA isopentenyltransferase 1 | 1.81 |
| LOC501980 | Rattus norvegicus similar to 40S ribosomal protein S19 | 1.81 |
| Ppia | Rattus norvegicus peptidylprolyl isomerase A | 1.81 |
| Mcl1 | Rattus norvegicus myeloid cell leukemia sequence 1 | 1.8 |
| LOC499457 | Rattus norvegicus similar to 60S ribosomal protein L7a | 1.8 |
| Ccng2 | Rattus norvegicus cyclin G2 | 1.79 |
| Rps3a | Rattus norvegicus ribosomal protein S3a | 1.79 |
| Dmd | Rattus norvegicus dystrophin | 1.78 |
| LOC360807 | Rattus norvegicus LOC360807 | 1.78 |
| LOC294700 | Rattus norvegicus similar to ribosomal protein L21 | 1.78 |
| Rpl35 | Rattus norvegicus ribosomal protein L35 | 1.78 |
| LOC310648 | Rattus norvegicus similar to glyceraldehyde-3-phosphate dehydrogenase | 1.77 |
| LOC497816 | Rattus norvegicus hypothetical gene supported by NM_019371 | 1.77 |
| Hif1a | Rattus norvegicus hypoxia inducible factor 1, alpha subunit | 1.77 |
| Ednrb | Rattus norvegicus endothelin receptor type B | 1.77 |
| Clta | Rattus norvegicus clathrin, light polypeptide | 1.77 |
| Rps20 | Rattus norvegicus ribosomal protein S20 | 1.77 |
| Ndfip2 | Rattus norvegicus Nedd4 family interacting protein 2 | 1.76 |
| Fau | Rattus norvegicus Finkel-Biskis-Reilly murine sarcoma virusubiquitously expressed | 1.76 |
| Rpl18 | Rattus norvegicus ribosomal protein L18 | 1.76 |
| LOC302528 | Rattus norvegicus similar to 60S ribosomal protein L37a | 1.76 |
| Cfl1 | Rattus norvegicus cofilin 1 | 1.76 |
| Rpl6 | Rattus norvegicus ribosomal protein L6 | 1.76 |
| LOC363531 | Rattus norvegicus similar to 40S ribosomal protein S19 | 1.75 |
| Hnrpl | Rattus norvegicus heterogeneous nuclear ribonucleoprotein L | 1.75 |
| Rps3 | Rattus norvegicus ribosomal protein S3 | 1.75 |
| LOC309408 | Rattus norvegicus similar to ribosomal protein S12 | 1.75 |
| LOC300870 | Rattus norvegicus similar to hypothetical protein FLJ20037 | 1.74 |
| Rabggtb | Rattus norvegicus RAB geranylgeranyl transferase, b subunit | 1.73 |
| Rpl17 | Rattus norvegicus ribosomal protein L17 | 1.73 |
| Jund | Rattus norvegicus Jun D proto-oncogene | 1.72 |
| LOC307135 | Rattus norvegicus similar to ribosomal protein L34 | 1.71 |
| LOC296582 | Rattus norvegicus similar to ribosomal protein S2 | 1.7 |
| Rps5 | Rattus norvegicus ribosomal protein S5 | 1.7 |
| Rpl7a | Rattus norvegicus ribosomal protein L7a | 1.7 |
| LOC367398 | Rattus norvegicus similar to 60S ribosomal protein L17 | 1.7 |
| Pim3 | Rattus norvegicus serine/threonine-protein kinase pim-3 | 1.69 |
| Cggbp1 | Rattus norvegicus CGG triplet repeat binding protein 1 | 1.68 |
| Slc40a1 | Rattus norvegicus solute carrier family 39 | 1.67 |
| Mdm4 | Rattus norvegicus transformed mouse 3T3 cell double minute 4 | 1.67 |
| LOC500226 | Rattus norvegicus similar to D3Mm3e | 1.67 |
| Cops4 | Rattus norvegicus COP9 signalosome subunit 4 | 1.67 |
| LOC498661 | Rattus norvegicus similar to pyruvate kinase | 1.66 |
| LOC311772 | Rattus norvegicus similar to nidogen 2 | 1.65 |
| Ccnl1 | Rattus norvegicus cyclin L1 | 1.65 |
| Rragc | Rattus norvegicus Ras-related GTP binding C | 1.65 |
| LOC498555 | Rattus norvegicus similar to 60S acidic ribosomal protein P2 | 1.65 |
| Calr | Rattus norvegicus calreticulin | 1.65 |
| Pamci | Rattus norvegicus peptidylglycine alpha-amidating monooxygenase COOH-terminal interactor | 1.64 |
| LOC500559 | Rattus norvegicus similar to 40S ribosomal protein S20 | 1.64 |
| LOC315642 | Rattus norvegicus similar to ribosomal protein L27a | 1.64 |
| Arih1 | Rattus norvegicus ariadne ubiquitin-conjugating enzyme E2 binding protein homolog 1 | 1.63 |
| LOC361613 | Rattus norvegicus similar to Protein phosphatase methylesterase 1 | 1.63 |
| Npm1 | Rattus norvegicus nucleophosmin 1 | 1.63 |
| Lamp1 | Rattus norvegicus lysosomal membrane glycoprotein 1 | 1.62 |
| Tm4sf9 | Rattus norvegicus transmembrane 4 superfamily member 9 | 1.61 |
| Rpl24 | Rattus norvegicus ribosomal protein L24 | 1.61 |
| Atp6v1g1 | Rattus norvegicus ATPase, H+ transporting, V1 subunit G isoform 1 | 1.61 |
| Ywhae | Rattus norvegicus tyrosine 3-monooxygenase/tryptophan 5-monooxygenase activation protein, epsilon polypeptide | 1.61 |
| Lama5 | Rattus norvegicus laminin, alpha 5 | 1.6 |
| LOC499133 | Rattus norvegicus similar to 60S ribosomal protein L27a | 1.6 |
| Pttg1ip | Rattus norvegicus pituitary tumor-transforming 1 interacting protein | 1.6 |
| LOC500856 | Rattus norvegicus hypothetical gene supported by BC087105 | 1.59 |
| Rps2 | Rattus norvegicus ribosomal protein S2 | 1.59 |
| Rps25 | Rattus norvegicus ribosomal protein s25 | 1.59 |
| Bsg | Rattus norvegicus basigin | 1.58 |
| LOC293103 | Rattus norvegicus similar to RIKEN cDNA 0610007P06 | 1.58 |
| Rab24 | Rattus norvegicus RAB24, member RAS oncogene family | 1.58 |
| Hdlbp | Rattus norvegicus high density lipoprotein binding protein | 1.58 |
| Atp5b | Rattus norvegicus ATP synthase, H+ transporting, mitochondrial F1 complex, beta polypeptide | 1.58 |
| Eif3s5 | Rattus norvegicus eukaryotic translation initiation factor 3, subunit 5 | 1.58 |
| Dtr | Rattus norvegicus diphtheria toxin receptor | 1.57 |
| Plekhm2 | Rattus norvegicus pleckstrin homology domain containing, family M | 1.57 |
| Ckb | Rattus norvegicus creatine kinase, brain | 1.57 |
| Actn1 | Rattus norvegicus actinin, alpha 1 | 1.56 |
| LOC364108 | Rattus norvegicus similar to ribosomal protein S17 | 1.56 |
| Prss23 | Rattus norvegicus protease, serine, 23 | 1.55 |
| Uba52 | Rattus norvegicus ubiquitin A-52 residue ribosomal protein fusion product 1 | 1.55 |
| Tln | Rattus norvegicus talin | 1.54 |
| Rps6 | Rattus norvegicus ribosomal protein S6 | 1.54 |
| Gsk3b | Rattus norvegicus glycogen synthase kinase 3 beta | 1.53 |
| RGD1311518 | Rattus norvegicus similar to rab3 GTPase-activating protein, non-catalytic subunit | 1.53 |
| Cd83 | Rattus norvegicus CD83 antigen | 1.53 |
| LOC366693 | Rattus norvegicus similar to S164 | 1.53 |
| Mdm2 | Rattus norvegicus transformed mouse 3T3 cell double minute 2 | 1.53 |
| Ubqln1 | Rattus norvegicus ubiquilin 1 | 1.53 |
| LOC299041 | Rattus norvegicus similar to 60S acidic ribosomal protein P1 | 1.53 |
| LOC503110 | Rattus norvegicus similar to ribosomal protein S19 | 1.53 |
| Slc25a4 | Rattus norvegicus solute carrier family 25 | 1.53 |
| Rpl8 | Rattus norvegicus ribosomal protein L8 | 1.53 |
| Nol8 | Rattus norvegicus nucleolar protein 8 | 1.52 |
| LOC498105 | Rattus norvegicus similar to LRRGT00176 | 1.52 |
| Vapa | Rattus norvegicus vesicle-associated membrane protein, associated protein a | 1.52 |
| LOC299935 | Rattus norvegicus similar to ribosomal protein L31 | 1.52 |
| Junb | Rattus norvegicus Jun-B oncogene | 1.51 |
| LOC362290 | Rattus norvegicus similar to 60S ribosomal protein L7a | 1.51 |
| Rps15 | Rattus norvegicus ribosomal protein S15 | 1.51 |
| LOC499305 | Rattus norvegicus similar to Finkel-Biskis-Reilly murine sarcoma virus | 1.51 |
| LOC367857 | Rattus norvegicus similar to ubiquitin fusion degradation protein 2 | 1.5 |
| Eef2 | Rattus norvegicus eukaryotic translation elongation factor 2 | 1.5 |
| Gnai2 | Rattus norvegicus guanine nucleotide binding protein, alpha inhibiting 2 | 1.5 |
| Peci | Rattus norvegicus peroxisomal delta3, delta2-enoyl-Coenzyme A isomerase | -1.5 |
| Nup107 | Rattus norvegicus nucleoporin 107 | -1.5 |
| Hat1 | Rattus norvegicus histone aminotransferase 1 | -1.5 |
| Pank2 | Rattus norvegicus pantothenate kinase 2 | -1.51 |
| Ubl3 | Rattus norvegicus ubiquitin-like 3 | -1.51 |
| Slc20a2 | Rattus norvegicus solute carrier family 20, member 2 | -1.51 |
| Mapk7 | Rattus norvegicus mitogen-activated protein kinase 7 | -1.51 |
| Rasl11b | Rattus norvegicus RAS-like family 11 member B | -1.51 |
| LOC290372 | Rattus norvegicus similar to expressed sequence AU021034 | -1.51 |
| LOC290833 | Rattus norvegicus similar to RIKEN cDNA 2410018G23 | -1.51 |
| LOC362592 | Rattus norvegicus hypothetical LOC362592 | -1.51 |
| LOC361315 | Rattus norvegicus similar to Ac2-256 | -1.51 |
| Arpc5 | Rattus norvegicus actin related protein 2/3 complex, subunit 5 | -1.51 |
| Hint3 | Rattus norvegicus histidine triad nucleotide binding protein 3 | -1.52 |
| Ythdf2 | Rattus norvegicus YTH domain family 2 | -1.52 |
| Sema6a | Rattus norvegicus sema domain, transmembrane domain | -1.52 |
| Rab3d | Rattus norvegicus RAB3D, member RAS oncogene family | -1.52 |
| Ube4b | Rattus norvegicus ubiquitination factor E4B, UFD2 homolog | -1.52 |
| LOC501550 | Rattus norvegicus similar to Sedlin | -1.52 |
| LOC361712 | Rattus norvegicus similar to HRD1 protein; synoviolin 1 | -1.52 |
| Mrps17 | Rattus norvegicus mitochondrial ribosomal protein S17 | -1.53 |
| LOC315697 | Rattus norvegicus similar to RIKEN cDNA 1190002L16 | -1.53 |
| Ndufa8 | Rattus norvegicus NADH dehydrogenase | -1.53 |
| RGD1307008 | Rattus norvegicus similar to RIKEN cDNA 4833420K19 | -1.53 |
| Cerk | Rattus norvegicus ceramide kinase | -1.53 |
| RGD1310284 | Rattus norvegicus similar to RIKEN cDNA C230075L19 gene | -1.53 |
| Nde1 | Rattus norvegicus nuclear distribution gene E homolog 1 | -1.53 |
| MGC94479 | Rattus norvegicus similar to Protein C3orf4 homolog | -1.53 |
| Ext2 | Rattus norvegicus exostoses | -1.53 |
| Rgs3 | Rattus norvegicus regulator of G-protein signalling 3 | -1.53 |
| MGC94282 | Rattus norvegicus similar to 5930416I19Rik protein | -1.54 |
| LOC502017 | Rattus norvegicus similar to Dolichol-phosphate mannosyltransferase subunit 3 | -1.54 |
| Mrpl49 | Rattus norvegicus mitochondrial ribosomal protein L49 | -1.54 |
| Usp47 | Rattus norvegicus ubiquitin specific protease 47 | -1.54 |
| Snx14 | Rattus norvegicus sorting nexin 14 | -1.54 |
| Ier2 | Rattus norvegicus immediate early response 2 | -1.54 |
| Pgrmc1 | Rattus norvegicus progesterone receptor membrane component 1 | -1.54 |
| Cdkn3 | Rattus norvegicus cyclin-dependent kinase inhibitor 3 | -1.55 |
| Psma5 | Rattus norvegicus proteasome | -1.55 |
| LOC500855 | Rattus norvegicus similar to RIKEN cDNA 3110006P09 | -1.55 |
| RGD1311463 | Rattus norvegicus similar to RIKEN cDNA 2700007P21 | -1.56 |
| Psme2 | Rattus norvegicus protease | -1.56 |
| Eppb9 | Rattus norvegicus endothelial precursor protein B9 | -1.56 |
| LOC502782 | Rattus norvegicus similar to RIKEN cDNA 2610022G08 | -1.56 |
| LOC361502 | Rattus norvegicus similar to B430201G11Rik protein | -1.56 |
| G3bp | Rattus norvegicus Ras-GTPase-activating protein SH3-domain binding protein | -1.56 |
| LOC498433 | Rattus norvegicus similar to proteasome | -1.56 |
| Rcn2 | Rattus norvegicus reticulocalbin 2 | -1.56 |
| Rcn | Rattus norvegicus reticulocalbin | -1.56 |
| Ipo13 | Rattus norvegicus importin 13 | -1.57 |
| Lama2 | Rattus norvegicus laminin, alpha 2 | -1.57 |
| LOC294734 | Rattus norvegicus similar to RIKEN cDNA 1700034P14 | -1.57 |
| Abr | Rattus norvegicus active BCR-related gene | -1.57 |
| Mtvr2 | Rattus norvegicus mammary tumor virus receptor 2 | -1.57 |
| LOC361695 | Rattus norvegicus similar to 2410001H17Rik protein | -1.57 |
| LOC360894 | Rattus norvegicus similar to hypothetical protein FLJ14146 | -1.58 |
| LOC303567 | Rattus norvegicus similar to RIKEN cDNA 2010008E23 gene | -1.58 |
| Zfp297b | Rattus norvegicus zinc finger protein 297B | -1.58 |
| LOC316085 | Rattus norvegicus similar to 106 kDa O-GlcNAc transferase-interacting protein | -1.58 |
| LOC500441 | Rattus norvegicus similar to testes development-related NYD-SP22 isoform 1 | -1.58 |
| Cd2bp2 | Rattus norvegicus CD2 antigen | -1.58 |
| Tlk1 | Rattus norvegicus tousled-like kinase 1 | -1.58 |
| Ahr | Rattus norvegicus aryl hydrocarbon receptor | -1.58 |
| Ube4a | Rattus norvegicus ubiquitin conjugation factor E4 A | -1.58 |
| LOC361980 | Rattus norvegicus similar to RIKEN cDNA 2810403A07 | -1.58 |
| LOC363309 | Rattus norvegicus similar to tubulin-specific chaperone d | -1.58 |
| Arpc1a | Rattus norvegicus actin related protein 2/3 complex, subunit 1A | -1.58 |
| Rnf7 | Rattus norvegicus ring finger protein 7 | -1.59 |
| LOC296469 | Rattus norvegicus similar to chromosome 20 open reading frame 58 | -1.59 |
| Mak10 | Rattus norvegicus corneal wound healing related protein | -1.59 |
| Adprt | Rattus norvegicus ADP-ribosyltransferase 1 | -1.59 |
| RGD1310571 | Rattus norvegicus similar to hypothetical protein | -1.6 |
| LOC362578 | Rattus norvegicus similar to RIKEN cDNA 2410005K17 | -1.6 |
| Pou3f3 | Rattus norvegicus POU domain, class 3, transcription factor 3 | -1.6 |
| Scamp2 | Rattus norvegicus secretory carrier membrane protein 2 | -1.6 |
| Rbm4 | Rattus norvegicus RNA binding motif protein 4 | -1.6 |
| Slc39a13 | Rattus norvegicus solute carrier family 39 | -1.61 |
| Ctsh | Rattus norvegicus cathepsin H | -1.61 |
| RGD1310022 | Rattus norvegicus similar to RIKEN cDNA 2610204K14 | -1.61 |
| Mgst2 | Rattus norvegicus microsomal glutathione S-transferase 2 | -1.61 |
| Msx1 | Rattus norvegicus homeo box, msh-like 1 | -1.61 |
| Snx4 | Rattus norvegicus sorting nexin 4 | -1.61 |
| LOC305633 | Rattus norvegicus similar to Antxr2 protein | -1.61 |
| Thra | Rattus norvegicus thyroid hormone receptor alpha | -1.61 |
| Gtl3 | Rattus norvegicus gene trap locus 3 | -1.62 |
| Map2k4 | Rattus norvegicus mitogen activated protein kinase kinase 4 | -1.62 |
| LOC309081 | Rattus norvegicus similar to Dock1 protein | -1.62 |
| Mocs2 | Rattus norvegicus molybdopterin synthase | -1.62 |
| Lyplal1 | Rattus norvegicus lysophospholipase-like 1 | -1.63 |
| LOC499335 | Rattus norvegicus similar to frataxin | -1.63 |
| LOC302553 | Rattus norvegicus similar to Su | -1.63 |
| LOC499882 | Rattus norvegicus similar to CREBBP/EP300 inhibitory protein 1 | -1.63 |
| Prelp | Rattus norvegicus proline arginine-rich end leucine-rich repeat protein | -1.63 |
| Traf4af1 | Rattus norvegicus TRAF4 associated factor 1 | -1.63 |
| Nedd9 | Rattus norvegicus neural precursor cell expressed, developmentally down-regulated gene 9 | -1.63 |
| LOC288707 | Rattus norvegicus similar to Glycolipid transfer protein | -1.63 |
| Nid2 | Rattus norvegicus nidogen 2 | -1.63 |
| LOC501633 | Rattus norvegicus LOC501633 | -1.64 |
| Exosc4 | Rattus norvegicus exosome component 4 | -1.64 |
| LOC362317 | Rattus norvegicus similar to krev interaction trapped-1A | -1.64 |
| Mkl1 | Rattus norvegicus megakaryoblastic leukemia | -1.64 |
| Cxcr4 | Rattus norvegicus chemokine | -1.64 |
| Zcwcc1 | Rattus norvegicus zinc finger, CW-type with coiled-coil domain 1 | -1.65 |
| Chst2 | Rattus norvegicus carbohydrate sulfotransferase 2 | -1.65 |
| Krt1-19 | Rattus norvegicus keratin complex 1, acidic, gene 19 | -1.65 |
| Scp2 | Rattus norvegicus sterol carrier protein 2 | -1.65 |
| Fjx1 | Rattus norvegicus four jointed box 1 | -1.65 |
| RGD1307935 | Rattus norvegicus similar to Hypothetical protein MGC18716 | -1.65 |
| Adamts1 | Rattus norvegicus a disintegrin-like and metalloprotease | -1.65 |
| Amacr | Rattus norvegicus alpha-methylacyl-CoA racemase | -1.65 |
| Plekhb2 | Rattus norvegicus pleckstrin homology domain containing, family B | -1.65 |
| LOC498736 | Rattus norvegicus similar to tubulin, beta 2 | -1.65 |
| LOC292751 | Rattus norvegicus similar to RIKEN cDNA 2810405O22 | -1.66 |
| Stc1 | Rattus norvegicus stanniocalcin 1 | -1.66 |
| Fh1 | Rattus norvegicus fumarate hydratase 1 | -1.66 |
| Exosc7 | Rattus norvegicus exosome component 7 | -1.66 |
| Polr2b | Rattus norvegicus polymerase | -1.66 |
| Sfxn1 | Rattus norvegicus sideroflexin 1 | -1.66 |
| Map3k4 | Rattus norvegicus mitogen activated protein kinase kinase kinase 4 | -1.66 |
| Acp2 | Rattus norvegicus acid phosphatase 2, lysosomal | -1.66 |
| Dutp | Rattus norvegicus deoxyuridine triphosphatase | -1.66 |
| Col5a1 | Rattus norvegicus collagen, type V, alpha 1 | -1.66 |
| LOC366481 | Rattus norvegicus similar to Zgc:56193 | -1.66 |
| Fgfrl1 | Rattus norvegicus fibroblast growth factor receptor-like 1 | -1.67 |
| LOC293888 | Rattus norvegicus similar to RIKEN cDNA 5033414D02 | -1.67 |
| Fndc3 | Rattus norvegicus fibronectin type III domain containing 3 | -1.67 |
| LOC498398 | Rattus norvegicus similar to selenoprotein SelM | -1.67 |
| Boc | Rattus norvegicus biregional cell adhesion molecule-related/down-regulated by oncogenes | -1.67 |
| Nov | Rattus norvegicus nephroblastoma overexpressed gene | -1.67 |
| LOC288659 | Rattus norvegicus similar to Hypothetical protein MGC25614 | -1.68 |
| LOC501521 | Rattus norvegicus similar to T-complex associated-testis-expressed 1-like | -1.68 |
| Col16a1 | Rattus norvegicus procollagen, type XVI, alpha 1 | -1.68 |
| LOC498107 | Rattus norvegicus LOC498107 | -1.68 |
| Srpr | Rattus norvegicus signal recognition particle receptor | -1.68 |
| LOC362776 | Rattus norvegicus similar to 2900070E19Rik protein | -1.68 |
| Csad | Rattus norvegicus cysteine sulfinic acid decarboxylase | -1.68 |
| Prr3 | Rattus norvegicus proline-rich polypeptide 3 | -1.68 |
| LOC303630 | Rattus norvegicus similar to D11Ertd498e protein | -1.68 |
| LOC498564 | Rattus norvegicus similar to integrin, beta-like 1 | -1.69 |
| RGD1311243 | Rattus norvegicus similar to DKFZP566O084 protein | -1.69 |
| LOC498035 | Rattus norvegicus similar to cylindromatosis | -1.69 |
| Ywhah | Rattus norvegicus tyrosine 3-monooxygenase/tryptophan 5-monooxygenase activation protein, eta polypeptide | -1.69 |
| RGD1308373 | Rattus norvegicus similar to DKFZP566K1924 protein | -1.7 |
| LOC499125 | Rattus norvegicus LOC499125 | -1.7 |
| Dr1 | Rattus norvegicus down-regulator of transcription 1 | -1.7 |
| Ppp1r14b | Rattus norvegicus protein phosphatase 1, regulatory | -1.7 |
| Zfp462 | Rattus norvegicus zinc finger protein 462 | -1.71 |
| Lphn1 | Rattus norvegicus latrophilin 1 | -1.71 |
| RGD1307010 | Rattus norvegicus similar to RIKEN cDNA 2700085E05 | -1.71 |
| Stk16 | Rattus norvegicus serine/threonine kinase 16 | -1.71 |
| Chp | Rattus norvegicus calcium binding protein p22 | -1.71 |
| Pigt | Rattus norvegicus phosphatidylinositol glycan, class T | -1.71 |
| Gstp1 | Rattus norvegicus glutathione-S-transferase, pi 1 | -1.71 |
| LOC299341 | Rattus norvegicus similar to RIKEN cDNA 2810002N01 | -1.72 |
| Tmpo | Rattus norvegicus thymopoietin | -1.72 |
| Cenpb | Rattus norvegicus centromere autoantigen B | -1.72 |
| mrpl11 | Rattus norvegicus mitochondrial ribosomal protein L11 | -1.73 |
| LOC296733 | Rattus norvegicus similar to mKIAA1402 protein | -1.73 |
| MGC94463 | Rattus norvegicus O-linked mannose beta1,2-N-acetylglucosaminyltransferase | -1.73 |
| Srrm2 | Rattus norvegicus serine/arginine repetitive matrix 2 | -1.73 |
| LOC302313 | Rattus norvegicus similar to transmembrane 4 superfamily member 6 | -1.73 |
| Mmp14 | Rattus norvegicus matrix metalloproteinase 14 | -1.73 |
| Cul3 | Rattus norvegicus cullin 3 | -1.74 |
| Pycrl | Rattus norvegicus pyrroline-5-carboxylate reductase-like | -1.74 |
| Gprasp1 | Rattus norvegicus G protein-coupled receptor associated sorting protein 1 | -1.74 |
| Psmb10 | Rattus norvegicus proteasome | -1.74 |
| Calm1 | Rattus norvegicus calmodulin 1 | -1.74 |
| LOC499496 | Rattus norvegicus LOC499496 | -1.75 |
| Gnpat | Rattus norvegicus glyceronephosphate O-acyltransferase | -1.75 |
| Sec5l1 | Rattus norvegicus Rsec5 protein | -1.75 |
| Svil | Rattus norvegicus supervillin | -1.75 |
| Chd4 | Rattus norvegicus chromodomain helicase DNA binding protein 4 | -1.75 |
| Tfpi | Rattus norvegicus tissue factor pathway inhibitor | -1.76 |
| LOC500462 | Rattus norvegicus similar to calcium binding protein P22 | -1.76 |
| Casp2 | Rattus norvegicus caspase 2 | -1.76 |
| Rrm2 | Rattus norvegicus ribonucleotide reductase M2 | -1.76 |
| LOC309848 | Rattus norvegicus similar to Tubulin alpha-2 chain | -1.76 |
| LOC360602 | Rattus norvegicus similar to cisplatin resistance-associated overexpressed protein | -1.76 |
| Ccna2 | Rattus norvegicus cyclin A2 | -1.77 |
| Ifitm1 | Rattus norvegicus interferon induced transmembrane protein 1 | -1.77 |
| Arf2 | Rattus norvegicus ADP-ribosylation factor 2 | -1.78 |
| Fzd1 | Rattus norvegicus frizzled homolog 1 | -1.78 |
| Hnrpa3 | Rattus norvegicus heterogeneous nuclear ribonucleoprotein A3 | -1.78 |
| RGD1308470 | Rattus norvegicus similar to RIKEN cDNA 4933433P14 gene | -1.79 |
| Trp53rk | Rattus norvegicus TP53 regulating kinase | -1.79 |
| D123 | Rattus norvegicus D123 gene product | -1.79 |
| Slc29a3 | Rattus norvegicus solute carrier family 29 | -1.79 |
| Cdc91l1 | Rattus norvegicus CDC91 cell division cycle 91-like 1 | -1.79 |
| Plxnd1 | Rattus norvegicus plexin D1 | -1.79 |
| LOC497712 | Rattus norvegicus hypothetical gene supported by NM_001001511 | -1.79 |
| Mesdc2 | Rattus norvegicus mesoderm development candiate 2 | -1.79 |
| LOC296758 | Rattus norvegicus similar to RIKEN cDNA 2810037C14 | -1.79 |
| Myadm | Rattus norvegicus myeloid-associated differentiation marker | -1.79 |
| Ywhab | Rattus norvegicus tyrosine 3-monooxygenase/tryptophan 5-monooxygenase activation protein, beta polypeptide | -1.79 |
| LOC293844 | Rattus norvegicus similar to UCH37-interacting protein 1 | -1.8 |
| Fbn2 | Rattus norvegicus fibrillin 2 | -1.8 |
| Glrx1 | Rattus norvegicus glutaredoxin 1 | -1.8 |
| LOC316916 | Rattus norvegicus similar to cisplatin resistance related protein CRR9p | -1.8 |
| Lrrk1 | Rattus norvegicus leucine-rich repeat kinase 1 | -1.81 |
| Mtch2 | Rattus norvegicus mitochondrial carrier homolog 2 | -1.81 |
| Kai1 | Rattus norvegicus kangai 1 | -1.81 |
| Cycs | Rattus norvegicus cytochrome c, somatic | -1.81 |
| Dcn | Rattus norvegicus decorin | -1.81 |
| Lactb | Rattus norvegicus lactamase, beta | -1.82 |
| Kdelr3 | Rattus norvegicus KDEL | -1.82 |
| Ckap4 | Rattus norvegicus cytoskeleton-associated protein 4 | -1.82 |
| Cyp1b1 | Rattus norvegicus cytochrome P450, family 1, subfamily b, polypeptide 1 | -1.82 |
| LOC360738 | Rattus norvegicus similar to Ser/Thr-rich protein T10 in DGCR region | -1.83 |
| Rb1cc1 | Rattus norvegicus RB1-inducible coiled-coil 1 | -1.83 |
| Msh6 | Rattus norvegicus mutS homolog 6 | -1.83 |
| Gorasp2 | Rattus norvegicus golgi reassembly stacking protein 2 | -1.83 |
| RGD1306395 | Rattus norvegicus similar to 9530046H09Rik protein | -1.83 |
| Cxcl12 | Rattus norvegicus chemokine | -1.83 |
| Rom1 | Rattus norvegicus rod outer segment membrane protein 1 | -1.84 |
| Agrn | Rattus norvegicus agrin | -1.84 |
| Pabpn1 | Rattus norvegicus poly | -1.84 |
| Lypla1 | Rattus norvegicus lysophospholipase 1 | -1.84 |
| Bcat2 | Rattus norvegicus branched chain aminotransferase 2, mitochondrial | -1.85 |
| LOC308708 | Rattus norvegicus similar to hypothetical protein FLJ12572 | -1.85 |
| Cnot7 | Rattus norvegicus CCR4-NOT transcription complex, subunit 7 | -1.85 |
| Tsn | Rattus norvegicus translin | -1.85 |
| Rarres2 | Rattus norvegicus retinoic acid receptor responder | -1.85 |
| B4galt4 | Rattus norvegicus UDP-Gal:betaGlcNAc beta 1,4-galactosyltransferase, polypeptide 4 | -1.86 |
| Pros1 | Rattus norvegicus protein S | -1.86 |
| Klf7 | Rattus norvegicus Kruppel-like factor 7 | -1.86 |
| Laptm4b | Rattus norvegicus lysosomal-associated protein transmembrane 4B | -1.86 |
| LOC290925 | Rattus norvegicus similar to RIKEN cDNA 2410022L05 | -1.86 |
| LOC310760 | Rattus norvegicus similar to cDNA sequence BC003236 | -1.86 |
| LOC301119 | Rattus norvegicus similar to Btk-PH-domain binding protein | -1.86 |
| c-fos | Rattus norvegicus c-fos oncogene | -1.86 |
| Cirbp | Rattus norvegicus cold inducible RNA binding protein | -1.87 |
| Zmpste24 | Rattus norvegicus zinc metalloproteinase, STE24 homolog | -1.87 |
| Gstt2 | Rattus norvegicus glutathione S-transferase, theta 2 | -1.87 |
| Tec | Rattus norvegicus tec protein tyrosine kinase | -1.88 |
| Lgals3bp | Rattus norvegicus lectin, galactoside-binding, soluble, 3 binding protein | -1.88 |
| Emilin1 | Rattus norvegicus elastin microfibril interfacer 1 | -1.88 |
| LOC298906 | Rattus norvegicus similar to RIKEN cDNA E030024M05 | -1.88 |
| LOC299828 | Rattus norvegicus similar to Ku70-binding protein 3 | -1.88 |
| MGC93733 | Rattus norvegicus similar to RIKEN cDNA 6530411B15 | -1.88 |
| LOC313699 | Rattus norvegicus similar to RIKEN cDNA 2510039O18 | -1.88 |
| Slc33a1 | Rattus norvegicus solute carrier family 33 | -1.89 |
| Wee1 | Rattus norvegicus wee 1 homolog | -1.89 |
| Statip1 | Rattus norvegicus signal transducer and activator of transcription interacting protein 1 | -1.89 |
| Slc16a1 | Rattus norvegicus solute carrier family 16 | -1.89 |
| Eln | Rattus norvegicus elastin | -1.9 |
| LOC498751 | Rattus norvegicus similar to RP23-462P2.7 | -1.9 |
| Ythdf1 | Rattus norvegicus YTH domain family 1 | -1.9 |
| LOC313974 | Rattus norvegicus similar to Tribbles homolog 2 | -1.9 |
| Cdk2ap1 | Rattus norvegicus CDK2 | -1.9 |
| LOC292792 | Rattus norvegicus similar to seven transmembrane domain protein | -1.9 |
| Bbp | Rattus norvegicus beta-amyloid binding protein precursor | -1.9 |
| Calm3 | Rattus norvegicus calmodulin 3 | -1.9 |
| Sfrs5 | Rattus norvegicus splicing factor, arginine/serine-rich 5 | -1.9 |
| LOC498410 | Rattus norvegicus similar to novel protein similar to Tensin Tns | -1.9 |
| RGD1307203 | Rattus norvegicus hypothetical LOC287199 | -1.91 |
| Blcap | Rattus norvegicus bladder cancer associated protein homolog | -1.91 |
| Pdk2 | Rattus norvegicus pyruvate dehydrogenase kinase, isoenzyme 2 | -1.91 |
| Ucp2 | Rattus norvegicus uncoupling protein 2 | -1.91 |
| Evl | Rattus norvegicus Ena-vasodilator stimulated phosphoprotein | -1.91 |
| Mig12 | Rattus norvegicus MID1 interacting G12-like protein | -1.91 |
| Ehd4 | Rattus norvegicus EH-domain containing 4 | -1.92 |
| RGD1305356 | Rattus norvegicus similar to RIKEN cDNA 3110031B13 | -1.92 |
| Atp2a2 | Rattus norvegicus ATPase, Ca++ transporting, cardiac muscle, slow twitch 2 | -1.92 |
| Dab2 | Rattus norvegicus disabled homolog 2 | -1.92 |
| Prkar2b | Rattus norvegicus protein kinase, cAMP dependent regulatory, type II beta | -1.93 |
| MGC94555 | Rattus norvegicus intimal thickness-related receptor | -1.93 |
| Sara1 | Rattus norvegicus SAR1a gene homolog 1 | -1.93 |
| Txndc7 | Rattus norvegicus thioredoxin domain containing 7 | -1.93 |
| LOC360627 | Rattus norvegicus similar to 65kDa FK506-binding protein | -1.93 |
| Tpbg | Rattus norvegicus trophoblast glycoprotein | -1.94 |
| LOC361605 | Rattus norvegicus similar to mKIAA0824 protein | -1.95 |
| Dnajc9 | Rattus norvegicus DnaJ | -1.95 |
| Tsnax | Rattus norvegicus translin-associated factor X | -1.95 |
| Amd1 | Rattus norvegicus S-adenosylmethionine decarboxylase 1 | -1.96 |
| Id1 | Rattus norvegicus Inhibitor of DNA binding 1, helix-loop-helix protein | -1.96 |
| LOC292995 | Rattus norvegicus similar to BBP-like protein 2 | -1.97 |
| Mlycd | Rattus norvegicus malonyl-CoA decarboxylase | -1.98 |
| Sfpq | Rattus norvegicus NonO/p54nrb homolog | -1.98 |
| Cdipt | Rattus norvegicus CDP-diacylglycerol--inositol 3-phosphatidyltransferase | -1.98 |
| LOC302378 | Rattus norvegicus similar to G protein-coupled receptor 23 | -1.99 |
| LOC498095 | Rattus norvegicus similar to RIKEN cDNA 0610012D17 | -1.99 |
| Pon2 | Rattus norvegicus paraoxonase 2 | -1.99 |
| Psmb3 | Rattus norvegicus proteasome | -1.99 |
| RGD1307632 | Rattus norvegicus similar to tumor-related protein | -2 |
| Ormdl2 | Rattus norvegicus ORM1-like 2 | -2 |
| Mospd3 | Rattus norvegicus motile sperm domain containing 3 | -2 |
| Snrpa | Rattus norvegicus small nuclear ribonucleoprotein polypeptide A | -2 |
| Ssbp1 | Rattus norvegicus single-stranded DNA binding protein 1 | -2.01 |
| Lepre1 | Rattus norvegicus leprecan 1 | -2.01 |
| Ndr4 | Rattus norvegicus N-myc downstream regulated 4 | -2.02 |
| Pofut2 | Rattus norvegicus protein O-fucosyltransferase 2 | -2.02 |
| Fxyd6 | Rattus norvegicus FXYD domain-containing ion transport regulator 6 | -2.02 |
| Efnb1 | Rattus norvegicus ephrin B1 | -2.02 |
| Tdg | Rattus norvegicus thymine-DNA glycosylase | -2.02 |
| LOC497841 | Rattus norvegicus hypothetical gene supported by NM_016994 | -2.02 |
| RAMP4 | Rattus norvegicus ribosome associated membrane protein 4 | -2.02 |
| Lsm8 | Rattus norvegicus LSM8 homolog, U6 small nuclear RNA associated | -2.03 |
| RGD1311316 | Rattus norvegicus similar to RIKEN cDNA 5730470L24 | -2.03 |
| Gp1bb | Rattus norvegicus peanut | -2.03 |
| RGD1305133 | Rattus norvegicus similar to Ab2-008 | -2.03 |
| Csrp2 | Rattus norvegicus cysteine and glycine-rich protein 2 | -2.03 |
| LOC360632 | Rattus norvegicus similar to RIKEN cDNA 1300010M03 | -2.04 |
| LOC500039 | Rattus norvegicus similar to Adenylate kinase 2 | -2.04 |
| LOC300149 | Rattus norvegicus similar to hypothetical protein D15Ertd785e | -2.04 |
| Col11a1 | Rattus norvegicus procollagen, type XI, alpha 1 | -2.05 |
| LOC498750 | Rattus norvegicus similar to cDNA sequence BC005537 | -2.05 |
| LOC500015 | Rattus norvegicus similar to mKIAA2005 protein | -2.06 |
| Siat4b | Rattus norvegicus sialyltransferase 4B | -2.06 |
| Ctps | Rattus norvegicus cytidine 5'-triphosphate synthase | -2.06 |
| Col6a3 | Rattus norvegicus procollagen, type VI, alpha 3 | -2.06 |
| Sec24d | Rattus norvegicus SEC24 related gene family, member D | -2.07 |
| Id3 | Rattus norvegicus inhibitor of DNA binding 3 | -2.07 |
| Abcd3 | Rattus norvegicus ATP-binding cassette, sub-family D | -2.07 |
| Lass2 | Rattus norvegicus longevity assurance homolog 2 | -2.07 |
| LOC298500 | Rattus norvegicus similar to hypothetical protein AL133206 | -2.07 |
| LOC317218 | Rattus norvegicus similar to Integral membrane protein 2A | -2.07 |
| LOC499196 | Rattus norvegicus LOC499196 | -2.08 |
| Cntf | Rattus norvegicus ciliary neurotrophic factor | -2.08 |
| LOC361178 | Rattus norvegicus similar to transcription factor | -2.08 |
| Hmgn3 | Rattus norvegicus high mobility group nucleosomal binding domain 3 | -2.09 |
| Abhd8 | Rattus norvegicus abhydrolase domain containing 8 | -2.09 |
| LOC360821 | Rattus norvegicus similar to Putative protein 15E1.2 | -2.09 |
| Leprot | Rattus norvegicus leptin receptor overlapping transcript | -2.09 |
| Gpr37l1 | Rattus norvegicus G protein-coupled receptor 37-like 1 | -2.1 |
| Olig1 | Rattus norvegicus oligodendrocyte transcription factor 1 | -2.1 |
| Thtpa | Rattus norvegicus thiamine triphosphatase | -2.11 |
| LOC501282 | Rattus norvegicus similar to lymphocyte antigen 6 complex, locus E ligand | -2.12 |
| LOC312915 | Rattus norvegicus similar to brefeldin A-inhibited guanine nucleotide-exchange protein 1 | -2.12 |
| Ttc13 | Rattus norvegicus tetratricopeptide repeat domain 13 | -2.12 |
| Pigs | Rattus norvegicus phosphatidylinositol glycan, class S | -2.13 |
| Zfp347 | Rattus norvegicus zinc finger protein 347 | -2.14 |
| LOC499087 | Rattus norvegicus similar to MKIAA1064 protein | -2.14 |
| Ppp1ca | Rattus norvegicus protein phosphatase 1, catalytic subunit, alpha isoform | -2.15 |
| Pphln1 | Rattus norvegicus periphilin 1 | -2.16 |
| Efemp1 | Rattus norvegicus epidermal growth factor-containing fibulin-like extracellular matrix protein 1 | -2.16 |
| Polr2d | Rattus norvegicus polymerase | -2.16 |
| LOC289181 | Rattus norvegicus similar to IQ motif and WD repeats 1 | -2.16 |
| Rbm24 | Rattus norvegicus RNA binding motif protein 24 | -2.16 |
| Atp5j | Rattus norvegicus ATP synthase, H+ transporting, mitochondrial F0 complex, subunit F6 | -2.16 |
| Ppap2b | Rattus norvegicus ER transmembrane protein Dri 42 | -2.16 |
| LOC500629 | Rattus norvegicus similar to alcohol dehydrogenase PAN2 | -2.17 |
| Stard3nl | Rattus norvegicus STARD3 N-terminal like | -2.17 |
| Zic2 | Rattus norvegicus Zic family member 2 | -2.17 |
| Mcmd6 | Rattus norvegicus mini chromosome maintenance deficient 6 | -2.18 |
| LOC305310 | Rattus norvegicus similar to RIKEN cDNA 5033405K12 | -2.18 |
| Lamb1-1 | Rattus norvegicus laminin B1 subunit 1 | -2.18 |
| Atp1b1 | Rattus norvegicus ATPase, Na+/K+ transporting, beta 1 polypeptide | -2.19 |
| AF146738 | Rattus norvegicus testis specific protein | -2.19 |
| Reck | Rattus norvegicus reversion-inducing-cysteine-rich protein with kazal motifs | -2.19 |
| Fbxo11 | Rattus norvegicus F-box only protein 11 | -2.2 |
| Coq3 | Rattus norvegicus coenzyme Q3 homolog, methyltransferase | -2.2 |
| Mknk2 | Rattus norvegicus MAP kinase-interacting serine/threonine kinase 2 | -2.2 |
| Slc6a8 | Rattus norvegicus choline transporter | -2.21 |
| RGD1310553 | Rattus norvegicus similar to expressed sequence AI597479 | -2.22 |
| Api5 | Rattus norvegicus apoptosis inhibitor 5 | -2.22 |
| LOC362264 | Rattus norvegicus similar to dJ862K6.2.2 | -2.22 |
| S100a16 | Rattus norvegicus S100 calcium binding protein A16 | -2.22 |
| Col3a1 | Rattus norvegicus collagen, type III, alpha 1 | -2.22 |
| Serpinb1a | Rattus norvegicus serine | -2.23 |
| Sdfr1 | Rattus norvegicus stromal cell derived factor receptor 1 | -2.23 |
| Ltap | Rattus norvegicus loop tail associated protein | -2.24 |
| Nr2f1 | Rattus norvegicus nuclear receptor subfamily 2, group F, member 1 | -2.24 |
| MGC94969 | Rattus norvegicus transmembrane protein vezatin | -2.24 |
| LOC361467 | Rattus norvegicus similar to hypothetical protein FLJ20481 | -2.24 |
| Lsm7 | Rattus norvegicus LSM7 homolog, U6 small nuclear RNA associated | -2.25 |
| Olfml2b | Rattus norvegicus olfactomedin-like 2B | -2.25 |
| Rhoq | Rattus norvegicus ras homolog gene family, member Q | -2.25 |
| LOC362040 | Rattus norvegicus similar to RIKEN cDNA 2310008M10 | -2.25 |
| RGD1309685 | Rattus norvegicus similar to RIKEN cDNA 2310075G12 | -2.26 |
| LOC302980 | Rattus norvegicus similar to RIKEN cDNA 1110025H10 | -2.27 |
| LOC497673 | Rattus norvegicus hypothetical gene supported by NM_172035 | -2.27 |
| Prss35 | Rattus norvegicus protease, serine, 35 | -2.27 |
| LOC287212 | Rattus norvegicus similar to hypothetical protein FLJ31951 | -2.27 |
| Cfh | Rattus norvegicus complement component factor H | -2.28 |
| Hspa2 | Rattus norvegicus heat shock protein 2 | -2.29 |
| LOC363443 | Rattus norvegicus similar to NDP | -2.29 |
| Alg5 | Rattus norvegicus asparagine-linked glycosylation 5 homolog | -2.29 |
| Tnfrsf11b | Rattus norvegicus tumor necrosis factor receptor superfamily, member 11b | -2.29 |
| LOC498674 | Rattus norvegicus LOC498674 | -2.29 |
| LOC499593 | Rattus norvegicus similar to SOX2 protein | -2.3 |
| LOC289400 | Rattus norvegicus similar to KIAA1078 protein | -2.3 |
| Nrp1 | Rattus norvegicus neuropilin 1 | -2.31 |
| Bak1 | Rattus norvegicus BCL2-antagonist/killer 1 | -2.31 |
| LOC499856 | Rattus norvegicus similar to RIKEN cDNA 1110018M03 | -2.32 |
| LOC310640 | Rattus norvegicus similar to chromosome 1 open reading frame 2 | -2.32 |
| MGC94686 | Rattus norvegicus similar to RIKEN cDNA 2810413N20 | -2.32 |
| Cspg5 | Rattus norvegicus chondroitin sulfate proteoglycan 5 | -2.33 |
| Tia1 | Rattus norvegicus cytotoxic granule-associated RNA binding protein 1 | -2.34 |
| Zhx1 | Rattus norvegicus zinc-fingers and homeoboxes 1 | -2.35 |
| Sc65 | Rattus norvegicus synaptonemal complex protein SC65 | -2.35 |
| Gap43 | Rattus norvegicus growth associated protein 43 | -2.35 |
| RGD1310386 | Rattus norvegicus similar to hypothetical protein MGC10067 | -2.36 |
| Ei24 | Rattus norvegicus etoposide induced 2.4 mRNA | -2.37 |
| MGC105691 | Rattus norvegicus NIPSNAP-related protein | -2.38 |
| Tnmd | Rattus norvegicus tenomodulin | -2.38 |
| LOC306324 | Rattus norvegicus similar to tetraspanin similar to TM4SF9 | -2.39 |
| Np | Rattus norvegicus nucleoside phosphorylase | -2.39 |
| Sart2 | Rattus norvegicus squamous cell carcinoma antigen recognized by T cells 2 | -2.4 |
| Lrrc5 | Rattus norvegicus leucine-rich repeat-containing 5 | -2.4 |
| Hspa14 | Rattus norvegicus heat shock protein hsp70-related protein | -2.4 |
| Sqle | Rattus norvegicus squalene epoxidase | -2.41 |
| Ripk5 | Rattus norvegicus receptor interacting protein kinase 5 | -2.42 |
| RGD1305486 | Rattus norvegicus similar to RIKEN cDNA 2810405J04 | -2.43 |
| Cfl2 | Rattus norvegicus cofilin 2, muscle | -2.43 |
| Fut8 | Rattus norvegicus fucosyltransferase 8 | -2.44 |
| Tm9sf4 | Rattus norvegicus transmembrane 9 superfamily protein member 4 | -2.45 |
| LOC498407 | Rattus norvegicus similar to purine rich element binding protein B | -2.45 |
| Cbfb | Rattus norvegicus core binding factor beta | -2.45 |
| Kpna2 | Rattus norvegicus karyopherin | -2.45 |
| LOC304091 | Rattus norvegicus similar to class II cytokine receptor 4 | -2.46 |
| AY228474 | Rattus norvegicus DNA sequence AY228474 | -2.46 |
| RGD1305625 | Rattus norvegicus similar to RIKEN cDNA 2310075C12 | -2.48 |
| Bmp7 | Rattus norvegicus bone morphogenetic protein 7 | -2.49 |
| Anxa11 | Rattus norvegicus annexin A11 | -2.49 |
| Pcyox1 | Rattus norvegicus chloride ion pump-associated 55 kDa protein | -2.5 |
| Minpp1 | Rattus norvegicus multiple inositol polyphosphate histidine phosphatase 1 | -2.51 |
| Crabp2 | Rattus norvegicus cellular retinoic acid binding protein 2 | -2.51 |
| Asf1a | Rattus norvegicus ASF1 anti-silencing function 1 homolog A | -2.52 |
| Zfp36 | Rattus norvegicus zinc finger protein 36 | -2.52 |
| LOC361213 | Rattus norvegicus similar to Serine palmitoyltransferase 1 | -2.53 |
| Crot | Rattus norvegicus carnitine O-octanoyltransferase | -2.54 |
| LOC499084 | Rattus norvegicus similar to hepatic multiple inositol polyphosphate phosphatase | -2.55 |
| Nfatc4 | Rattus norvegicus nuclear factor of activated T-cells, cytoplasmic, calcineurin-dependent 4 | -2.56 |
| LOC501028 | Rattus norvegicus similar to mitochondrial ribosomal protein L41 | -2.57 |
| LOC303514 | Rattus norvegicus similar to RIKEN cDNA 4121402D02 | -2.58 |
| Gbl | Rattus norvegicus G protein beta subunit-like | -2.58 |
| RGD1305689 | Rattus norvegicus similar to DNA segment, Chr 14, ERATO Doi 449, expressed | -2.58 |
| Sphk1 | Rattus norvegicus sphingosine kinase 1 | -2.59 |
| LOC294942 | Rattus norvegicus hypothetical LOC294942 | -2.59 |
| LOC499380 | Rattus norvegicus similar to empty spiracles-like protein 2 | -2.6 |
| LOC499772 | Rattus norvegicus similar to immediate early response 5-like | -2.6 |
| Scara3 | Rattus norvegicus scavenger receptor class A, member 3 | -2.61 |
| Phc2 | Rattus norvegicus polyhomeotic-like 2 | -2.62 |
| LOC309475 | Rattus norvegicus similar to transmembrane protein TM9SF3 | -2.62 |
| Gusb | Rattus norvegicus glucuronidase, beta | -2.65 |
| Csf1 | Rattus norvegicus colony stimulating factor 1 | -2.66 |
| Prkaca | Rattus norvegicus protein kinase, cAMP-dependent, catalytic, alpha | -2.66 |
| Hsd3b7 | Rattus norvegicus CCA2 protein | -2.67 |
| MGC94167 | Rattus norvegicus similar to RIKEN cDNA C130099A20 | -2.68 |
| Thumpd1 | Rattus norvegicus THUMP domain containing 1 | -2.7 |
| Loxl1 | Rattus norvegicus lysyl oxidase-like 1 | -2.7 |
| LOC500252 | Rattus norvegicus similar to Gene model 461 | -2.71 |
| LOC499775 | Rattus norvegicus LOC499775 | -2.71 |
| LOC306587 | Rattus norvegicus similar to RIKEN cDNA 9130410M22 | -2.72 |
| Mrps12 | Rattus norvegicus mitochondrial ribosomal protein S12 | -2.73 |
| Rbp1 | Rattus norvegicus retinol binding protein 1, cellular | -2.75 |
| Pigq | Rattus norvegicus phosphatidylinositol glycan, class Q | -2.76 |
| B4galt6 | Rattus norvegicus UDP-Gal:betaGlcNAc beta 1,4-galactosyltransferase, polypeptide 6 | -2.81 |
| Colec12 | Rattus norvegicus collectin sub-family member 12 | -2.83 |
| Btg2 | Rattus norvegicus B-cell translocation gene 2, anti-proliferative | -2.83 |
| Cx3cl1 | Rattus norvegicus chemokine | -2.84 |
| Unc50 | Rattus norvegicus unc-50 homolog | -2.84 |
| Cspg2 | Rattus norvegicus chondroitin sulfate proteoglycan 2 | -2.85 |
| LOC360886 | Rattus norvegicus similar to transcription factor ELYS | -2.86 |
| Pfn2 | Rattus norvegicus profilin 2 | -2.86 |
| Aqp1 | Rattus norvegicus aquaporin 1 | -2.87 |
| Rab11a | Rattus norvegicus RAB11a, member RAS oncogene family | -2.95 |
| Ttyh1 | Rattus norvegicus tweety homolog 1 | -2.95 |
| RGD1310861 | Rattus norvegicus similar to RIKEN cDNA 1500011H22 | -2.95 |
| Postn | Rattus norvegicus periostin, osteoblast specific factor | -2.95 |
| Tmem9 | Rattus norvegicus transmembrane protein 9 | -2.99 |
| Cyp26b1 | Rattus norvegicus cytochrome P450, family 26, subfamily b, polypeptide 1 | -3 |
| Hmgcr | Rattus norvegicus 3-hydroxy-3-methylglutaryl-Coenzyme A reductase | -3.07 |
| LOC360546 | Rattus norvegicus similar to m-ephrin-B3 | -3.08 |
| Cpt2 | Rattus norvegicus carnitine palmitoyltransferase 2 | -3.09 |
| Dscr1 | Rattus norvegicus Down syndrome critical region homolog 1 | -3.11 |
| Kazald1 | Rattus norvegicus Kazal-type serine protease inhibitor domain 1 | -3.17 |
| Pdgfra | Rattus norvegicus platelet derived growth factor receptor, alpha polypeptide | -3.2 |
| MGC94018 | Rattus norvegicus glycosyltransferase AD-017 | -3.21 |
| Chdh | Rattus norvegicus choline dehydrogenase | -3.22 |
| Col1a1 | Rattus norvegicus collagen, type 1, alpha 1 | -3.23 |
| LOC306229 | Rattus norvegicus similar to RIKEN cDNA A630054L15; hypothetical protein MGC38041 | -3.26 |
| Idh1 | Rattus norvegicus isocitrate dehydrogenase 1 | -3.26 |
| Tnfrsf1a | Rattus norvegicus tumor necrosis factor receptor superfamily, member 1a | -3.27 |
| MGC95138 | Rattus norvegicus similar to acetyl CoA transferase-like | -3.33 |
| Hn1 | Rattus norvegicus hematological and neurological expressed sequence 1 | -3.4 |
| Alcam | Rattus norvegicus activated leukocyte cell adhesion molecule | -3.45 |
| Sesn1 | Rattus norvegicus sestrin 1 | -3.5 |
| Cd164l1 | Rattus norvegicus CD164 sialomucin-like 1 | -3.52 |
| LOC294789 | Rattus norvegicus similar to Hypothetical protein FLJ25422 | -3.57 |
| Hmgcs1 | Rattus norvegicus 3-hydroxy-3-methylglutaryl-Coenzyme A synthase 1 | -3.63 |
| Igsf11 | Rattus norvegicus immunoglobulin superfamily, member 11 | -3.65 |
| LOC500987 | Rattus norvegicus similar to Histone H2A.x | -3.69 |
| Dnajc10 | Rattus norvegicus DnaJ | -3.71 |
| Ctsk | Rattus norvegicus cathepsin K | -3.82 |
| LOC360698 | Rattus norvegicus similar to RIKEN cDNA B230114J08 | -4.03 |
| Cldn11 | Rattus norvegicus claudin 11 | -4.11 |
| Dspg3 | Rattus norvegicus dermatan sulphate proteoglycan 3 | -4.14 |
| Dhcr7 | Rattus norvegicus 7-dehydrocholesterol reductase | -4.33 |
| Gjb2 | Rattus norvegicus gap junction membrane channel protein beta 2 | -4.43 |
| Atp1a2 | Rattus norvegicus ATPase, Na+/K+ transporting, alpha 2 polypeptide | -4.53 |
| Enpp2 | Rattus norvegicus ectonucleotide pyrophosphatase/phosphodiesterase 2 | -4.53 |
| Ptgds | Rattus norvegicus prostaglandin D2 synthase | -6.25 |
| Txnip | Rattus norvegicus upregulated by 1,25-dihydroxyvitamin D-3 | -6.69 |
