# Supplementary material for: Multiple Oxygen Tension Environments Reveal Diverse Patterns of Transcriptional Regulation in Primary Astrocytes
Source: PLoS One. 2011 Jun 27;6(6):e21638. doi: 10.1371/journal.pone.0021638 (PMC3124552; doi:10.1371/journal.pone.0021638)
Supplement: Table S2 — Significantly regulated (p<0.05) genes in rat primary astrocytes exposed to 4% ambient O2 tension compared to 20% O2 tension. Z ratios were calculated as described in Materials and Methods. (DOC) [file pone.0021638.s008.doc]

**Table S2. Significantly regulated (p<0.05) genes in rat primary astrocytes exposed to 4% ambient O_2_ tension compared to 20% O_2_ tension**. Z ratios were calculated as described in Materials and Methods.

| **Symbol** | **Gene Definition** | **z ratio** |
| --- | --- | --- |
| Ca3 | Rattus norvegicus carbonic anhydrase 3 | 7.28 |
| Gdf10 | Rattus norvegicus growth differentiation factor 10 | 6.63 |
| Rbm3 | Rattus norvegicus RNA binding motif | 6.11 |
| Tnfrsf11b | Rattus norvegicus tumor necrosis factor receptor superfamily, member 11b | 5.37 |
| Snai1 | Rattus norvegicus snail homolog, | 4.97 |
| Lcn2 | Rattus norvegicus lipocalin 2 | 4.34 |
| Gstp2 | Rattus norvegicus glutathione S-transferase, pi 2 | 4.32 |
| S100a4 | Rattus norvegicus S100 calcium-binding protein A4 | 4.13 |
| Dspg3 | Rattus norvegicus dermatan sulphate proteoglycan 3 | 4.12 |
| Grem1 | Rattus norvegicus gremlin 1 homolog, cysteine knot superfamily | 4.07 |
| Gstp1 | Rattus norvegicus glutathione-S-transferase, pi 1 | 4.05 |
| LOC500721 | Rattus norvegicus LOC500721 | 3.88 |
| S100a6 | Rattus norvegicus S100 calcium binding protein A6 | 3.51 |
| Nqo1 | Rattus norvegicus NAD | 3.4 |
| Ptgis | Rattus norvegicus prostaglandin I2 | 3.37 |
| LOC498998 | Rattus norvegicus similar to 60S ribosomal protein L26 | 3.25 |
| LOC500817 | Rattus norvegicus similar to 40S ribosomal protein S20 | 3.18 |
| Rps17 | Rattus norvegicus ribosomal protein S17 | 3.12 |
| Ass | Rattus norvegicus argininosuccinate synthetase | 3.11 |
| LOC299935 | Rattus norvegicus similar to ribosomal protein L31 | 3.09 |
| Penk-rs | Rattus norvegicus preproenkephalin, related sequence | 3.08 |
| LOC498744 | Rattus norvegicus similar to ribosomal protein L37 | 3.02 |
| LOC314434 | Rattus norvegicus similar to 60S ribosomal protein L9 | 2.99 |
| LOC302528 | Rattus norvegicus similar to 60S ribosomal protein L37a | 2.95 |
| Serpine2 | Rattus norvegicus serine | 2.92 |
| LOC292273 | Rattus norvegicus similar to Hnrpa3 protein | 2.89 |
| Serpine1 | Rattus norvegicus serine | 2.86 |
| LOC367822 | Rattus norvegicus similar to ribosomal protein L5 | 2.85 |
| Fau | Rattus norvegicus Finkel-Biskis-Reilly murine sarcoma virusubiquitously expressed | 2.8 |
| Rpl35a | Rattus norvegicus ribosomal protein L35a | 2.77 |
| Ccl2 | Rattus norvegicus chemokine | 2.74 |
| LOC364108 | Rattus norvegicus similar to ribosomal protein S17 | 2.74 |
| S100a11 | Rattus norvegicus S100 calcium binding protein A11 | 2.74 |
| LOC289401 | Rattus norvegicus similar to ribosomal protein L31 | 2.7 |
| Fbln2 | Rattus norvegicus fibulin 2 | 2.68 |
| Mfap4 | Rattus norvegicus microfibrillar-associated protein 4 | 2.67 |
| LOC500155 | Rattus norvegicus similar to protein kinase C inhibitor | 2.67 |
| Rpl19 | Rattus norvegicus ribosomal protein L19 | 2.65 |
| Lum | Rattus norvegicus lumican | 2.64 |
| Vmp1 | Rattus norvegicus vacuole membrane protein 1 | 2.63 |
| LOC498099 | Rattus norvegicus similar to glyceraldehyde-3-phosphate dehydrogenase | 2.59 |
| Pdlim7 | Rattus norvegicus PDZ and LIM domain 7 | 2.58 |
| Cirbp | Rattus norvegicus cold inducible RNA binding protein | 2.57 |
| LOC500559 | Rattus norvegicus similar to 40S ribosomal protein S20 | 2.57 |
| Rps27a | Rattus norvegicus ribosomal protein S27a | 2.57 |
| Mt1a | Rattus norvegicus Metallothionein | 2.54 |
| Cfl1 | Rattus norvegicus cofilin 1 | 2.51 |
| LOC299041 | Rattus norvegicus similar to 60S acidic ribosomal protein P1 | 2.5 |
| Rps8 | Rattus norvegicus ribosomal protein S8 | 2.5 |
| Tnn | Rattus norvegicus tenascin N | 2.49 |
| LOC314556 | Rattus norvegicus similar to ribosomal protein S18 | 2.49 |
| Hes1 | Rattus norvegicus hairy and enhancer of split 1 | 2.48 |
| Pcolce | Rattus norvegicus procollagen C-proteinase enhancer protein | 2.48 |
| Lgals1 | Rattus norvegicus lectin, galactose binding, soluble 1 | 2.48 |
| Sui1-rs1 | Rattus norvegicus suppressor of initiator codon mutations, related sequence 1 | 2.46 |
| LOC363418 | Rattus norvegicus similar to Ac2-210 | 2.46 |
| Rpl10 | Rattus norvegicus ribosomal protein L10 | 2.45 |
| LOC362181 | Rattus norvegicus similar to Ac2-210 | 2.45 |
| Thbs2 | Rattus norvegicus thrombospondin 2 | 2.44 |
| Rps12 | Rattus norvegicus ribosomal protein S12 | 2.44 |
| LOC499592 | Rattus norvegicus similar to Hnrpa3 protein | 2.44 |
| Rpl26 | Rattus norvegicus ribosomal protein L26 | 2.44 |
| LOC501203 | Rattus norvegicus similar to Myosin regulatory light chain 2-A, smooth muscle isoform | 2.44 |
| Thy1 | Rattus norvegicus thymus cell antigen 1, theta | 2.39 |
| Atf4 | Rattus norvegicus activating transcription factor 4 | 2.38 |
| Nbl1 | Rattus norvegicus neuroblastoma, suppression of tumorigenicity 1 | 2.37 |
| LOC500965 | Rattus norvegicus similar to L-lactate dehydrogenase A chain | 2.36 |
| LOC364848 | Rattus norvegicus similar to Glyceraldehyde-3-phosphate dehydrogenase | 2.35 |
| LOC500451 | Rattus norvegicus similar to 40S ribosomal protein S20 | 2.33 |
| Ddit3 | Rattus norvegicus DNA-damage inducible transcript 3 | 2.31 |
| Gmps | Rattus norvegicus guanine monphosphate synthetase | 2.31 |
| Tgfb3 | Rattus norvegicus transforming growth factor, beta 3 | 2.3 |
| LOC500867 | Rattus norvegicus similar to LRRG00116 | 2.3 |
| Myd116 | Rattus norvegicus myeloid differentiation primary response gene 116 | 2.29 |
| Hnrpdl | Rattus norvegicus heterogeneous nuclear ribonucleoprotein D-like | 2.29 |
| LOC295423 | Rattus norvegicus similar to glyceraldehyde-3-phosphate dehydrogenase | 2.29 |
| Hmgn2 | Rattus norvegicus high mobility group protein 17 | 2.29 |
| Cct8 | Rattus norvegicus chaperonin subunit 8 | 2.28 |
| LOC499133 | Rattus norvegicus similar to 60S ribosomal protein L27a | 2.28 |
| LOC315642 | Rattus norvegicus similar to ribosomal protein L27a | 2.28 |
| LOC298785 | Rattus norvegicus similar to ribosomal protein S26 | 2.28 |
| Mk1 | Rattus norvegicus Mk1 protein | 2.27 |
| LOC498048 | Rattus norvegicus similar to ORF4 | 2.27 |
| Nfkbia | Rattus norvegicus nuclear factor of kappa light chain gene enhancer in B-cells inhibitor, alpha | 2.26 |
| LOC500983 | Rattus norvegicus similar to glyceraldehyde-3-phosphate dehydrogenase | 2.26 |
| Gm1012 | Rattus norvegicus gene model 1012, | 2.25 |
| LOC365954 | Rattus norvegicus similar to glyceraldehyde-3-phosphate dehydrogenase | 2.25 |
| Rps27 | Rattus norvegicus ribosomal protein S27 | 2.23 |
| LOC361912 | Rattus norvegicus similar to LRRG00116 | 2.22 |
| LOC502854 | Rattus norvegicus similar to ribosomal protein L31 | 2.22 |
| Mapre1 | Rattus norvegicus microtubule-associated protein, RP/EB family, member 1 | 2.22 |
| Gclc | Rattus norvegicus glutamate-cysteine ligase, catalytic subunit | 2.22 |
| Ppp1r14a | Rattus norvegicus protein phosphatase 1, regulatory | 2.2 |
| Adamts1 | Rattus norvegicus a disintegrin-like and metalloprotease | 2.2 |
| Nedd9 | Rattus norvegicus neural precursor cell expressed, developmentally down-regulated gene 9 | 2.2 |
| Eef1b2 | Rattus norvegicus eukaryotic translation elongation factor 1 beta 2 | 2.2 |
| LOC309408 | Rattus norvegicus similar to ribosomal protein S12 | 2.2 |
| Rps9 | Rattus norvegicus ribosomal protein S9 | 2.19 |
| LOC307135 | Rattus norvegicus similar to ribosomal protein L34 | 2.19 |
| LOC292539 | Rattus norvegicus similar to 60S ribosomal protein L17 | 2.18 |
| Ppic | Rattus norvegicus peptidylprolyl isomerase C | 2.18 |
| LOC500104 | Rattus norvegicus similar to Glyceraldehyde-3-phosphate dehydrogenase | 2.17 |
| LOC289715 | Rattus norvegicus similar to ribosomal protein L37 | 2.17 |
| Mrps18c | Rattus norvegicus mitochondrial ribosomal protein S18C | 2.16 |
| LOC363861 | Rattus norvegicus similar to 60S ribosomal protein L29 | 2.16 |
| Cnn1 | Rattus norvegicus calponin 1 | 2.15 |
| LOC295452 | Rattus norvegicus similar to Glyceraldehyde-3-phosphate dehydrogenase | 2.15 |
| Rpl37a | Rattus norvegicus ribosomal protein L37a | 2.15 |
| Ctgf | Rattus norvegicus connective tissue growth factor | 2.15 |
| LOC302363 | Rattus norvegicus similar to Sh3bgrl protein | 2.14 |
| Boc | Rattus norvegicus biregional cell adhesion molecule-related/down-regulated by oncogenes | 2.14 |
| Gstm2 | Rattus norvegicus glutathione S-transferase, mu 2 | 2.14 |
| LOC363492 | Rattus norvegicus similar to Ac1262 | 2.14 |
| LOC500271 | Rattus norvegicus similar to macrophage migration inhibitory factor | 2.13 |
| LOC498019 | Rattus norvegicus similar to glyceraldehyde-3-phosphate dehydrogenase | 2.13 |
| LOC366193 | Rattus norvegicus similar to 40S ribosomal protein S3a | 2.13 |
| LOC498360 | Rattus norvegicus similar to ribosomal protein S23 | 2.13 |
| Btf3 | Rattus norvegicus basic transcription factor 3 | 2.12 |
| Tle2 | Rattus norvegicus transducin-like enhancer of split 2, homolog of Drosophila E | 2.11 |
| LOC501280 | Rattus norvegicus similar to myosin regulatory light chain-like | 2.1 |
| Lamr1 | Rattus norvegicus laminin receptor 1 | 2.1 |
| Rps25 | Rattus norvegicus ribosomal protein s25 | 2.07 |
| Rgs14 | Rattus norvegicus regulator of G-protein signaling 14 | 2.06 |
| LOC501604 | Rattus norvegicus similar to 60S ribosomal protein L7a | 2.06 |
| Bgn | Rattus norvegicus biglycan | 2.06 |
| LOC499852 | Rattus norvegicus similar to 60S ribosomal protein L7a | 2.06 |
| LOC364343 | Rattus norvegicus similar to RIKEN cDNA A430107P09 gene | 2.05 |
| Ldha | Rattus norvegicus lactate dehydrogenase A | 2.05 |
| Ndr4 | Rattus norvegicus N-myc downstream regulated 4 | 2.04 |
| Rpl13a | Rattus norvegicus ribosomal protein L13A | 2.04 |
| Slc38a4 | Rattus norvegicus amino acid transport system A3 | 2.03 |
| Timp1 | Rattus norvegicus tissue inhibitor of metalloproteinase 1 | 2.02 |
| Rps20 | Rattus norvegicus ribosomal protein S20 | 2.01 |
| Rhoa | Rattus norvegicus ras homolog gene family, member A | 2 |
| LOC298126 | Rattus norvegicus similar to ribosomal protein L31 | 2 |
| LOC500885 | Rattus norvegicus similar to 40S ribosomal protein S19 | 2 |
| LOC293632 | Rattus norvegicus similar to RIKEN cDNA 6330512M04 gene | 1.99 |
| Fbn1 | Rattus norvegicus fibrillin 1 | 1.99 |
| Rpl8 | Rattus norvegicus ribosomal protein L8 | 1.99 |
| Tpm4 | Rattus norvegicus tropomyosin 4 | 1.98 |
| Sirt6 | Rattus norvegicus sirtuin 6 | 1.97 |
| LOC366887 | Rattus norvegicus similar to ribosomal protein L31 | 1.97 |
| LOC498555 | Rattus norvegicus similar to 60S acidic ribosomal protein P2 | 1.97 |
| LOC364828 | Rattus norvegicus similar to 60S ribosomal protein L29 | 1.96 |
| Rpl29 | Rattus norvegicus ribosomal protein L29 | 1.96 |
| Vim | Rattus norvegicus vimentin | 1.96 |
| LOC294781 | Rattus norvegicus similar to 60S ribosomal protein L21 | 1.94 |
| Rps7 | Rattus norvegicus ribosomal protein S7 | 1.94 |
| LOC366656 | Rattus norvegicus similar to ribosomal protein L10a | 1.94 |
| Ninj1 | Rattus norvegicus ninjurin 1 | 1.93 |
| Rpl41 | Rattus norvegicus ribosomal protein L41 | 1.93 |
| LOC302388 | Rattus norvegicus similar to ribosomal protein L19 | 1.92 |
| Rps13 | Rattus norvegicus ribosomal protein S13 | 1.92 |
| LOC499433 | Rattus norvegicus similar to glyceraldehyde-3-phosphate dehydrogenase | 1.92 |
| Cst3 | Rattus norvegicus cystatin C | 1.92 |
| Igf2 | Rattus norvegicus insulin-like growth factor 2 | 1.92 |
| LOC498062 | Rattus norvegicus similar to RIKEN cDNA 1190017O12 | 1.91 |
| LOC310360 | Rattus norvegicus similar to eukaryotic translation elongation factor 1 alpha 1 | 1.91 |
| LOC502302 | Rattus norvegicus similar to 40S ribosomal protein S19 | 1.91 |
| LOC503172 | Rattus norvegicus similar to DAZ associated protein 2 | 1.9 |
| LOC290634 | Rattus norvegicus similar to Glyceraldehyde-3-phosphate dehydrogenase | 1.9 |
| Rab9 | Rattus norvegicus RAB9, member RAS oncogene family | 1.89 |
| Col1a1 | Rattus norvegicus collagen, type 1, alpha 1 | 1.89 |
| Rplp1 | Rattus norvegicus ribosomal protein, large, P1 | 1.89 |
| Fth1 | Rattus norvegicus ferritin, heavy polypeptide 1 | 1.89 |
| LOC366485 | Rattus norvegicus similar to ribosomal protein L36 | 1.88 |
| LOC498828 | Rattus norvegicus similar to ribosomal protein L10 | 1.88 |
| LOC499660 | Rattus norvegicus similar to Cornifin alpha | 1.87 |
| Acta1 | Rattus norvegicus actin, alpha 1, skeletal muscle | 1.87 |
| Fgfr3 | Rattus norvegicus fibroblast growth factor receptor 3 | 1.87 |
| LOC361797 | Rattus norvegicus hypothetical LOC361797 | 1.87 |
| LOC363918 | Rattus norvegicus similar to ribosomal protein L27 | 1.87 |
| Ndufa5 | Rattus norvegicus NADH dehydrogenase | 1.86 |
| Akr1a1 | Rattus norvegicus aldo-keto reductase family 1, member A1 | 1.86 |
| Rps11 | Rattus norvegicus ribosomal protein S11 | 1.86 |
| Ctsl | Rattus norvegicus cathepsin L | 1.86 |
| LOC299622 | Rattus norvegicus similar to glyceraldehyde-3-phosphate dehydrogenase | 1.85 |
| Epdr2 | Rattus norvegicus ependymin related protein 2 | 1.85 |
| Rpl39 | Rattus norvegicus ribosomal protein L39 | 1.84 |
| Actb | Rattus norvegicus actin, beta | 1.84 |
| Dia1 | Rattus norvegicus diaphorase 1 | 1.83 |
| Rpl27 | Rattus norvegicus ribosomal protein L27 | 1.83 |
| Rps18 | Rattus norvegicus ribosomal protein S18 | 1.83 |
| Sdf4 | Rattus norvegicus stromal cell derived factor 4 | 1.82 |
| Sqrdl | Rattus norvegicus sulfide quinone reductase-like | 1.81 |
| Serpinf1 | Rattus norvegicus serine | 1.81 |
| Slc38a2 | Rattus norvegicus solute carrier family 38, member 2 | 1.81 |
| LOC500859 | Rattus norvegicus similar to 60S ribosomal protein L7a | 1.81 |
| LOC499305 | Rattus norvegicus similar to Finkel-Biskis-Reilly murine sarcoma virus | 1.81 |
| Ppia | Rattus norvegicus peptidylprolyl isomerase A | 1.81 |
| MGC72942 | Rattus norvegicus similar to CG6105-PA | 1.8 |
| LOC498881 | Rattus norvegicus similar to glyceraldehyde-3-phosphate dehydrogenase | 1.8 |
| Birc6 | Rattus norvegicus baculoviral IAP repeat-containing 6 | 1.79 |
| Rpl10a | Rattus norvegicus ribosomal protein L10A | 1.79 |
| Sema3f | Rattus norvegicus sema domain, immunoglobulin domain | 1.78 |
| LOC498212 | Rattus norvegicus similar to High mobility group protein 1 | 1.78 |
| Ecm1 | Rattus norvegicus extracellular matrix protein 1 | 1.78 |
| LOC307416 | Rattus norvegicus similar to Rpl7a protein | 1.78 |
| Cspg2 | Rattus norvegicus chondroitin sulfate proteoglycan 2 | 1.78 |
| LOC293860 | Rattus norvegicus similar to Filamin A | 1.78 |
| Ccl7 | Rattus norvegicus chemokine | 1.77 |
| LOC361061 | Rattus norvegicus hypothetical LOC361061 | 1.77 |
| LOC288146 | Rattus norvegicus similar to heterogeneous nuclear ribonucleoprotein A3 | 1.77 |
| Dnm1 | Rattus norvegicus dynamin 1 | 1.76 |
| LOC307731 | Rattus norvegicus similar to L-lactate dehydrogenase A chain | 1.76 |
| LOC502063 | Rattus norvegicus LOC502063 | 1.76 |
| LOC290912 | Rattus norvegicus similar to hypothetical protein FLJ10154 | 1.74 |
| LOC500645 | Rattus norvegicus similar to 60S ribosomal protein L29 | 1.74 |
| Ifitm3 | Rattus norvegicus interferon induced transmembrane protein 3 | 1.74 |
| Actg | Rattus norvegicus actin, gamma, cytoplasmic | 1.74 |
| LOC310512 | Rattus norvegicus similar to expressed sequence C87860 | 1.73 |
| Tceb2 | Rattus norvegicus transcription elongation factor B | 1.73 |
| LOC498523 | Rattus norvegicus similar to 60S ribosomal protein L23a | 1.73 |
| Rpl35 | Rattus norvegicus ribosomal protein L35 | 1.73 |
| LOC500929 | Rattus norvegicus similar to Tubulin alpha-2 chain | 1.73 |
| LOC294748 | Rattus norvegicus similar to Chain , Human Translation Initiation Factor Eif1, Nmr, 29 Structures | 1.72 |
| LOC366999 | Rattus norvegicus similar to 60S ribosomal protein L29 | 1.72 |
| Ppp3ca | Rattus norvegicus protein phosphatase 3, catalytic subunit, alpha isoform | 1.72 |
| LOC298495 | Rattus norvegicus similar to ribosomal protein L35a | 1.72 |
| Colm | Rattus norvegicus collomin | 1.72 |
| Psmb4 | Rattus norvegicus proteasome | 1.72 |
| LOC497813 | Rattus norvegicus similar to ribosomal protein S7 | 1.72 |
| Plcg1 | Rattus norvegicus phospholipase C, gamma 1 | 1.71 |
| Cmkor1 | Rattus norvegicus chemokine orphan receptor 1 | 1.7 |
| Mtch2 | Rattus norvegicus mitochondrial carrier homolog 2 | 1.7 |
| Arpc1b | Rattus norvegicus actin related protein 2/3 complex, subunit 1B | 1.7 |
| LOC367102 | Rattus norvegicus similar to 40S ribosomal protein S9 | 1.7 |
| Actr2 | Rattus norvegicus ARP2 actin-related protein 2 homolog | 1.7 |
| LOC499457 | Rattus norvegicus similar to 60S ribosomal protein L7a | 1.7 |
| LOC305887 | Rattus norvegicus similar to CG11030-PA | 1.68 |
| Commd3 | Rattus norvegicus Unknown | 1.68 |
| Prdx5 | Rattus norvegicus peroxiredoxin 5 | 1.68 |
| Rps14 | Rattus norvegicus ribosomal protein S14 | 1.68 |
| Runx1 | Rattus norvegicus runt related transcription factor 1 | 1.67 |
| MGC72560 | Rattus norvegicus Unknown | 1.67 |
| Igsf4c | Rattus norvegicus immunoglobulin superfamily, member 4C | 1.67 |
| LOC497693 | Rattus norvegicus similar to P11 protein | 1.67 |
| LOC299907 | Rattus norvegicus similar to Ext1 | 1.67 |
| LOC499501 | Rattus norvegicus similar to LRRGT00057 | 1.67 |
| Nat5 | Rattus norvegicus N-acetyltransferase 5 | 1.66 |
| Fdps | Rattus norvegicus farensyl diphosphate synthase | 1.66 |
| Tead3 | Rattus norvegicus TEA domain family member 3 | 1.66 |
| Pgk1 | Rattus norvegicus phosphoglycerate kinase 1 | 1.66 |
| F2r | Rattus norvegicus coagulation factor II | 1.66 |
| Jag1 | Rattus norvegicus jagged 1 | 1.65 |
| LOC306805 | Rattus norvegicus similar to asporin precursor | 1.65 |
| Exosc8 | Rattus norvegicus exosome component 8 | 1.65 |
| LOC298841 | Rattus norvegicus similar to apoptosis related protein APR-3; p18 protein | 1.65 |
| LOC498618 | Rattus norvegicus similar to glyceraldehyde-3-phosphate dehydrogenase | 1.65 |
| Slc3a2 | Rattus norvegicus solute carrier family 3 | 1.65 |
| Rps3 | Rattus norvegicus ribosomal protein S3 | 1.65 |
| Akap8 | Rattus norvegicus A kinase | 1.64 |
| Myl9 | Rattus norvegicus myosin, light polypeptide 9, regulatory | 1.64 |
| LOC499317 | Rattus norvegicus similar to UPF0197 protein C11orf10 homolog | 1.64 |
| MGC94283 | Rattus norvegicus integral type I protein | 1.63 |
| Ssr4 | Rattus norvegicus signal sequence receptor 4 | 1.63 |
| Sat | Rattus norvegicus spermidine/spermine N1-acetyl transferase | 1.63 |
| Tfb2m | Rattus norvegicus transcription factor B2, mitochondrial | 1.62 |
| LOC368016 | Rattus norvegicus similar to transmembrane 4 superfamily member 7; tetraspanin 4; novel antigen 2; tetraspan TM4SF | 1.62 |
| Sfrp2 | Rattus norvegicus secreted frizzled-related protein 2 | 1.62 |
| Stmn2 | Rattus norvegicus stathmin-like 2 | 1.62 |
| LOC498293 | Rattus norvegicus similar to basic transcription factor 3 | 1.62 |
| Ddah1 | Rattus norvegicus dimethylarginine dimethylaminohydrolase 1 | 1.62 |
| LOC501644 | Rattus norvegicus similar to Ferritin light chain 2 | 1.62 |
| Olfml3 | Rattus norvegicus olfactomedin-like 3 | 1.61 |
| Myadm | Rattus norvegicus myeloid-associated differentiation marker | 1.61 |
| LOC298169 | Rattus norvegicus similar to actin alpha 1 skeletal muscle protein | 1.61 |
| LOC294700 | Rattus norvegicus similar to ribosomal protein L21 | 1.61 |
| RGD1310991 | Rattus norvegicus similar to arsenite inducible RNA associated protein | 1.6 |
| LOC287132 | Rattus norvegicus similar to U1 snRNP-specific protein C | 1.6 |
| LOC296402 | Rattus norvegicus similar to Docking protein 5 | 1.6 |
| Agtr1a | Rattus norvegicus angiotensin II receptor, type 1 | 1.6 |
| LOC302497 | Rattus norvegicus similar to ribosomal protein L10a | 1.6 |
| LOC500923 | Rattus norvegicus similar to tumor protein, translationally-controlled 1 | 1.6 |
| LOC499906 | Rattus norvegicus similar to Eukaryotic translation initiation factor 1 | 1.6 |
| Aldoa | Rattus norvegicus aldolase A | 1.6 |
| Rpl18 | Rattus norvegicus ribosomal protein L18 | 1.6 |
| Atf5 | Rattus norvegicus activating transcription factor 5 | 1.59 |
| Arrdc1 | Rattus norvegicus arrestin domain containing 1 | 1.59 |
| LOC293888 | Rattus norvegicus similar to RIKEN cDNA 5033414D02 | 1.59 |
| Tnfrsf12a | Rattus norvegicus tumor necrosis factor receptor superfamily, member 12a | 1.59 |
| LOC300278 | Rattus norvegicus similar to 40S ribosomal protein S9 | 1.58 |
| Ssr2 | Rattus norvegicus signal sequence receptor, beta | 1.58 |
| Tagln | Rattus norvegicus transgelin | 1.58 |
| Prpf39 | Rattus norvegicus PRP39 pre-mRNA processing factor 39 homolog | 1.57 |
| Tcirg1 | Rattus norvegicus T-cell, immune regulator 1, ATPase, H+ transporting, lysosomal V0 protein a isoform 3 | 1.57 |
| LOC311120 | Rattus norvegicus similar to ribosomal protein L15 | 1.57 |
| Col8a1 | Rattus norvegicus procollagen, type VIII, alpha 1 | 1.57 |
| LOC363441 | Rattus norvegicus similar to NADH dehydrogenase | 1.57 |
| Eef1a1 | Rattus norvegicus eukaryotic translation elongation factor 1 alpha 1 | 1.57 |
| LOC314759 | Rattus norvegicus similar to genes associated with retinoid-IFN-induced mortality 19 | 1.56 |
| LOC364139 | Rattus norvegicus similar to ribosomal protein L21 | 1.56 |
| Eef1d | Rattus norvegicus eukaryotic translation elongation factor 1 delta | 1.56 |
| Ubb | Rattus norvegicus polyubiquitin | 1.56 |
| LOC498143 | Rattus norvegicus similar to ribosomal protein L15 | 1.56 |
| Phf3 | Rattus norvegicus PHD finger protein 3 | 1.55 |
| Icam1 | Rattus norvegicus intercellular adhesion molecule 1 | 1.55 |
| Gdi2 | Rattus norvegicus GDP dissociation inhibitor 2 | 1.55 |
| LOC364258 | Rattus norvegicus similar to RIKEN cDNA 1110003E01 | 1.54 |
| Tnpo3 | Rattus norvegicus transportin 3 | 1.54 |
| LOC300802 | Rattus norvegicus similar to APH1B homolog | 1.54 |
| LOC498398 | Rattus norvegicus similar to selenoprotein SelM | 1.54 |
| Fstl3 | Rattus norvegicus follistatin-like 3 | 1.53 |
| Arpp19 | Rattus norvegicus cAMP-regulated phosphoprotein 19 | 1.53 |
| Eif4el3 | Rattus norvegicus eukaryotic translation initiation factor 4E like 3 | 1.53 |
| Lxn | Rattus norvegicus latexin | 1.53 |
| Rps23 | Rattus norvegicus ribosomal protein S23 | 1.53 |
| LOC301299 | Rattus norvegicus similar to ribosomal protein L10a | 1.53 |
| Rps15 | Rattus norvegicus ribosomal protein S15 | 1.53 |
| LOC304035 | Rattus norvegicus similar to 60S ribosomal protein L7a | 1.52 |
| Fn1 | Rattus norvegicus fibronectin 1 | 1.52 |
| Tm4sf1 | Rattus norvegicus transmembrane 4 superfamily member 1 | 1.51 |
| LOC364381 | Rattus norvegicus similar to ribosomal protein S24 | 1.51 |
| Snrp70 | Rattus norvegicus U1 small nuclear ribonucleoprotein polypeptide A | 1.51 |
| RGD1307627 | Rattus norvegicus similar to gp25L2 protein | 1.5 |
| LOC497684 | Rattus norvegicus hypothetical gene supported by NM_017314 | 1.5 |
| Dnase2 | Rattus norvegicus deoxyribonuclease II | -1.5 |
| MGC72974 | Rattus norvegicus Unknown | -1.5 |
| Lyplal1 | Rattus norvegicus lysophospholipase-like 1 | -1.51 |
| Pros1 | Rattus norvegicus protein S | -1.51 |
| LOC499798 | Rattus norvegicus similar to ADP-ribosylation-like factor 6-interacting protein 6 | -1.51 |
| Abcd3 | Rattus norvegicus ATP-binding cassette, sub-family D | -1.51 |
| Igsf11 | Rattus norvegicus immunoglobulin superfamily, member 11 | -1.51 |
| Plekhb2 | Rattus norvegicus pleckstrin homology domain containing, family B | -1.51 |
| Nfia | Rattus norvegicus nuclear factor I/A | -1.51 |
| Pnrc1 | Rattus norvegicus proline rich 2 | -1.51 |
| Capza2 | Rattus norvegicus capping protein | -1.51 |
| Baalc | Rattus norvegicus brain and acute leukemia, cytoplasmic | -1.52 |
| Slc25a29 | Rattus norvegicus solute carrier family 25 | -1.52 |
| RGD735029 | Rattus norvegicus SEL1 domain containing protein RGD735029 | -1.52 |
| Podxl | Rattus norvegicus podocalyxin-like | -1.52 |
| MGC72987 | Rattus norvegicus Unknown | -1.52 |
| Tufm | Rattus norvegicus Tu translation elongation factor, mitochondrial | -1.52 |
| Hnrpa3 | Rattus norvegicus heterogeneous nuclear ribonucleoprotein A3 | -1.52 |
| LOC299050 | Rattus norvegicus similar to 1110008L16Rik protein | -1.53 |
| Donson | Rattus norvegicus downstream neighbor of SON | -1.53 |
| Lsamp | Rattus norvegicus limbic system-associated membrane protein | -1.53 |
| Tyki | Rattus norvegicus thymidylate kinase family LPS-inducible member | -1.53 |
| Nr2f1 | Rattus norvegicus nuclear receptor subfamily 2, group F, member 1 | -1.53 |
| Fchsd2 | Rattus norvegicus FCH and double SH3 domains 2 | -1.53 |
| LOC498931 | Rattus norvegicus similar to short coiled-coil protein | -1.53 |
| Mtpn | Rattus norvegicus myotrophin | -1.54 |
| Ppid | Rattus norvegicus peptidylprolyl isomerase D | -1.54 |
| Sorl1 | Rattus norvegicus sortilin-related receptor, L | -1.54 |
| Setdb1 | Rattus norvegicus SET domain, bifurcated 1 | -1.55 |
| Aif1 | Rattus norvegicus allograft inflammatory factor 1 | -1.56 |
| LOC296126 | Rattus norvegicus similar to U5 snRNP-specific protein, 200 kDa | -1.56 |
| Nt5c3 | Rattus norvegicus 5'-nucleotidase, cytosolic III | -1.56 |
| Ucp2 | Rattus norvegicus uncoupling protein 2 | -1.56 |
| Atp5c1 | Rattus norvegicus ATP synthase, H+ transporting, mitochondrial F1 complex, gamma polypeptide 1 | -1.56 |
| Pc | Rattus norvegicus Pyruvate carboxylase | -1.57 |
| LOC315645 | Rattus norvegicus similar to RIKEN cDNA 2700059L22 | -1.57 |
| RGD1303232 | Rattus norvegicus Phytn_dehydro and Pyr_redox domain containing protein RGD1303232 | -1.57 |
| Eml2 | Rattus norvegicus echinoderm microtubule associated protein like 2 | -1.58 |
| RGD1308373 | Rattus norvegicus similar to DKFZP566K1924 protein | -1.58 |
| Kif20a | Rattus norvegicus kinesin family member 20A | -1.58 |
| Tsn | Rattus norvegicus translin | -1.58 |
| LOC313974 | Rattus norvegicus similar to Tribbles homolog 2 | -1.58 |
| Psmd4 | Rattus norvegicus proteasome | -1.58 |
| LOC314949 | Rattus norvegicus similar to HR21spA | -1.58 |
| LOC311796 | Rattus norvegicus similar to cofactor of BRCA1; negative elongation factor protein B | -1.59 |
| LOC293566 | Rattus norvegicus similar to carboxypeptidase X 2 | -1.59 |
| Mdm2 | Rattus norvegicus transformed mouse 3T3 cell double minute 2 | -1.59 |
| LOC501157 | Rattus norvegicus similar to Ribulose-5-phosphate-3-epimerase | -1.59 |
| Polr2i | Rattus norvegicus polymerase | -1.59 |
| Rbm10 | Rattus norvegicus RNA binding motif protein 10 | -1.59 |
| Phyhd1 | Rattus norvegicus phytanoyl-CoA dioxygenase domain containing 1 | -1.6 |
| LOC500441 | Rattus norvegicus similar to testes development-related NYD-SP22 isoform 1 | -1.6 |
| LOC497766 | Rattus norvegicus hypothetical gene supported by NM_171983 | -1.6 |
| Pik3c3 | Rattus norvegicus phosphoinositide-3-kinase, class 3 | -1.61 |
| LOC306137 | Rattus norvegicus similar to CG10084-PA | -1.61 |
| Bf | Rattus norvegicus B-factor, properdin | -1.61 |
| Gbp2 | Rattus norvegicus guanylate nucleotide binding protein 2 | -1.61 |
| P4ha1 | Rattus norvegicus procollagen-proline, 2-oxoglutarate 4-dioxygenase | -1.61 |
| LOC500987 | Rattus norvegicus similar to Histone H2A.x | -1.61 |
| Smarca5 | Rattus norvegicus SWI/SNF related, matrix associated, actin dependent regulator of chromatin, subfamily a, member 5 | -1.62 |
| LOC315973 | Rattus norvegicus similar to 5730439E10Rik protein | -1.62 |
| LOC362414 | Rattus norvegicus similar to Tada3l protein | -1.62 |
| Lcat | Rattus norvegicus lecithin cholesterol acyltransferase | -1.62 |
| LOC499094 | Rattus norvegicus similar to zinc finger protein 61 | -1.63 |
| Sec24d | Rattus norvegicus SEC24 related gene family, member D | -1.63 |
| Rpia | Rattus norvegicus ribose 5-phosphate isomerase A | -1.63 |
| Urod | Rattus norvegicus uroporphyrinogen decarboxylase | -1.63 |
| Cldn9 | Rattus norvegicus claudin 9 | -1.64 |
| Zfp36l2 | Rattus norvegicus zinc finger protein 36, C3H type-like 2 | -1.64 |
| LOC309081 | Rattus norvegicus similar to Dock1 protein | -1.64 |
| Wdr34 | Rattus norvegicus WD repeat domain 34 | -1.65 |
| Calm3 | Rattus norvegicus calmodulin 3 | -1.65 |
| Aqp1 | Rattus norvegicus aquaporin 1 | -1.65 |
| Anxa5 | Rattus norvegicus annexin A5 | -1.66 |
| Lig1 | Rattus norvegicus ligase I, DNA, ATP-dependent | -1.67 |
| Nubp1 | Rattus norvegicus nucleotide binding protein 1 | -1.67 |
| LOC246187 | Rattus norvegicus liver regeneration-related protein | -1.67 |
| Cpe | Rattus norvegicus carboxypeptidase E | -1.67 |
| Atp6ap2 | Rattus norvegicus ATPase, H+ transporting, lysosomal accessory protein 2 | -1.67 |
| Kpna1 | Rattus norvegicus karyopherin | -1.68 |
| LOC315158 | Rattus norvegicus similar to ubiquitous tetratricopeptide containing protein RoXaN; Rotavirus X associated non-structural protein | -1.68 |
| Gabarapl2 | Rattus norvegicus GABA | -1.69 |
| Cox17 | Rattus norvegicus cytochrome c oxidase, subunit XVII assembly protein homolog | -1.69 |
| Cotl1 | Rattus norvegicus coactosin-like 1 | -1.69 |
| Rab11a | Rattus norvegicus RAB11a, member RAS oncogene family | -1.69 |
| LOC499196 | Rattus norvegicus LOC499196 | -1.69 |
| Xpo1 | Rattus norvegicus exportin 1, CRM1 homolog | -1.69 |
| Lypla1 | Rattus norvegicus lysophospholipase 1 | -1.69 |
| Hexa | Rattus norvegicus hexosaminidase A | -1.69 |
| Mlc1 | Rattus norvegicus megalencephalic leukoencephalopathy with subcortical cysts 1 | -1.69 |
| LOC500865 | Rattus norvegicus similar to RIKEN cDNA 5730410E15 gene | -1.7 |
| Fkbp5 | Rattus norvegicus FK506 binding protein 5 | -1.7 |
| Snrpa1 | Rattus norvegicus small nuclear ribonucleoprotein polypeptide A' | -1.7 |
| Tnmd | Rattus norvegicus tenomodulin | -1.7 |
| Lgi4 | Rattus norvegicus leucine-rich repeat LGI family, member 4 | -1.71 |
| Casp7 | Rattus norvegicus caspase 7 | -1.71 |
| Prtfdc1 | Rattus norvegicus phosphoribosyl transferase domain containing 1 | -1.72 |
| LOC302980 | Rattus norvegicus similar to RIKEN cDNA 1110025H10 | -1.72 |
| LOC360546 | Rattus norvegicus similar to m-ephrin-B3 | -1.72 |
| LOC301563 | Rattus norvegicus similar to RIKEN cDNA 5230400G24 | -1.72 |
| RGD1311049 | Rattus norvegicus similar to RIKEN cDNA 4833417L20 | -1.73 |
| LOC310946 | Rattus norvegicus similar to hypothetical protein FLJ20331 | -1.73 |
| Lr8 | Rattus norvegicus LR8 protein | -1.73 |
| Cfdp1 | Rattus norvegicus craniofacial development protein 1 | -1.73 |
| Adk | Rattus norvegicus adenosine kinase | -1.74 |
| RGD1310686 | Rattus norvegicus similar to chromosome 16 open reading frame 5 | -1.74 |
| LOC289900 | Rattus norvegicus hypothetical LOC289900 | -1.74 |
| Ttyh1 | Rattus norvegicus tweety homolog 1 | -1.74 |
| P2rxl1 | Rattus norvegicus purinergic receptor P2X-like 1, orphan receptor | -1.75 |
| Farslb | Rattus norvegicus phenylalanine-tRNA synthetase-like, beta subunit | -1.75 |
| MGC94969 | Rattus norvegicus transmembrane protein vezatin | -1.75 |
| LOC361519 | Rattus norvegicus similar to hypothetical protein MGC51082 | -1.75 |
| Dnajc10 | Rattus norvegicus DnaJ | -1.75 |
| Kifap3 | Rattus norvegicus kinesin-associated protein 3 | -1.76 |
| LOC368066 | Rattus norvegicus similar to thioether S-methyltransferase | -1.76 |
| Rkhd2 | Rattus norvegicus ring finger and KH domain containing 2 | -1.76 |
| AY228474 | Rattus norvegicus DNA sequence AY228474 | -1.76 |
| Ier2 | Rattus norvegicus immediate early response 2 | -1.76 |
| LOC302378 | Rattus norvegicus similar to G protein-coupled receptor 23 | -1.77 |
| Lpl | Rattus norvegicus lipoprotein lipase | -1.77 |
| Ivns1abp | Rattus norvegicus influenza virus NS1A binding protein | -1.77 |
| LOC304919 | Rattus norvegicus similar to RIKEN cDNA 5830468K18 | -1.78 |
| Ifi44 | Rattus norvegicus interferon-induced protein 44 | -1.78 |
| Flot1 | Rattus norvegicus flotillin 1 | -1.78 |
| Ednrb | Rattus norvegicus endothelin receptor type B | -1.78 |
| LOC362580 | Rattus norvegicus similar to CG2919-PA | -1.79 |
| LOC361988 | Rattus norvegicus similar to expressed sequence C77668 | -1.8 |
| LOC313445 | Rattus norvegicus similar to kelch-like 13 | -1.8 |
| RGD1309676 | Rattus norvegicus similar to RIKEN cDNA 5730469M10 | -1.81 |
| LOC499625 | Rattus norvegicus similar to Selenoprotein T precursor | -1.81 |
| LOC361309 | Rattus norvegicus similar to polyadenylate-binding protein-interacting protein 2 | -1.81 |
| Ptdss2 | Rattus norvegicus phosphatidylserine synthase 2 | -1.81 |
| Myc | Rattus norvegicus myelocytomatosis viral oncogene homolog | -1.82 |
| Bphl | Rattus norvegicus biphenyl hydrolase-like | -1.82 |
| Hes6 | Rattus norvegicus hairy and enhancer of split 6 | -1.82 |
| LOC294917 | Rattus norvegicus similar to Traf2 and NCK interacting kinase, splice variant 4 | -1.83 |
| Ppp4r2 | Rattus norvegicus protein phosphatase 4, regulatory subunit 2 | -1.84 |
| LOC287274 | Rattus norvegicus similar to RIKEN cDNA 0610009B22 | -1.84 |
| LOC304332 | Rattus norvegicus similar to hypothetical protein FLJ20397 | -1.84 |
| LOC498751 | Rattus norvegicus similar to RP23-462P2.7 | -1.85 |
| LOC292486 | Rattus norvegicus similar to Aig1 protein | -1.85 |
| LOC315804 | Rattus norvegicus similar to hypothetical protein FLJ12994 | -1.86 |
| Pdk1 | Rattus norvegicus pyruvate dehydrogenase kinase 1 | -1.86 |
| Nphp1 | Rattus norvegicus nephronophthisis 1 | -1.86 |
| Kpnb3 | Rattus norvegicus karyopherin | -1.86 |
| Capzb | Rattus norvegicus F-actin capping protein beta subunit | -1.86 |
| Gclm | Rattus norvegicus glutamate cysteine ligase, modifier subunit | -1.87 |
| Adss2 | Rattus norvegicus adenylosuccinate synthetase 2, non muscle | -1.87 |
| Nid2 | Rattus norvegicus nidogen 2 | -1.87 |
| Banp | Rattus norvegicus Btg3 associated nuclear protein | -1.88 |
| LOC498564 | Rattus norvegicus similar to integrin, beta-like 1 | -1.88 |
| Krt2-8 | Rattus norvegicus keratin complex 2, basic, gene 8 | -1.88 |
| LOC501341 | Rattus norvegicus similar to glutamate receptor, ionotropic, N-methyl D-aspartate-like 1A | -1.88 |
| Adprt | Rattus norvegicus ADP-ribosyltransferase 1 | -1.88 |
| Pcsk1n | Rattus norvegicus proprotein convertase subtilisin/kexin type 1 inhibitor | -1.89 |
| MGC105961 | Rattus norvegicus similar to mitochondrial ribosomal protein L13 | -1.89 |
| F3 | Rattus norvegicus coagulation factor III | -1.89 |
| LOC360478 | Rattus norvegicus hypothetical LOC360478 | -1.9 |
| Ggcx | Rattus norvegicus gamma-glutamyl carboxylase | -1.91 |
| Phr1 | Rattus norvegicus pam, highwire, rpm 1 | -1.91 |
| Sparcl1 | Rattus norvegicus SPARC-like 1 | -1.91 |
| LOC365214 | Rattus norvegicus similar to zinc finger protein ZFP235 | -1.93 |
| Jun | Rattus norvegicus v-jun sarcoma virus 17 oncogene homolog | -1.94 |
| Pex13 | Rattus norvegicus peroxisomal biogenesis factor 13 | -1.94 |
| Nrep | Rattus norvegicus neuronal regeneration related protein | -1.98 |
| RGD1308463 | Rattus norvegicus similar to IMP4 | -1.99 |
| LOC296758 | Rattus norvegicus similar to RIKEN cDNA 2810037C14 | -2 |
| Anxa3 | Rattus norvegicus annexin A3 | -2 |
| Ppp1r14b | Rattus norvegicus protein phosphatase 1, regulatory | -2.01 |
| LOC499328 | Rattus norvegicus similar to riboflavin kinase | -2.01 |
| Exosc3 | Rattus norvegicus exosome component 3 | -2.02 |
| LOC360941 | Rattus norvegicus similar to ORF7 | -2.03 |
| LOC499593 | Rattus norvegicus similar to SOX2 protein | -2.03 |
| Csda | Rattus norvegicus cold shock domain protein A | -2.03 |
| Fbln1 | Rattus norvegicus fibulin 1 | -2.03 |
| LOC497844 | Rattus norvegicus hypothetical gene supported by NM_138846 | -2.03 |
| Ascl1 | Rattus norvegicus achaete-scute complex homolog-like 1 | -2.04 |
| LOC305035 | Rattus norvegicus similar to D1Ertd396e protein | -2.04 |
| Galm | Rattus norvegicus galactose mutarotase | -2.04 |
| Hnrpm | Rattus norvegicus heterogeneous nuclear ribonucleoprotein M | -2.04 |
| LOC291354 | Rattus norvegicus similar to hypothetical protein MGC26778 | -2.05 |
| RGD1307008 | Rattus norvegicus similar to RIKEN cDNA 4833420K19 | -2.06 |
| LOC500536 | Rattus norvegicus similar to novel protein | -2.07 |
| Znf386 | Rattus norvegicus zinc finger protein 386 | -2.07 |
| Dhx32 | Rattus norvegicus DEAH | -2.08 |
| Vamp4 | Rattus norvegicus vesicle-associated membrane protein 4 | -2.08 |
| LOC171553 | Rattus norvegicus iGb3 synthase | -2.08 |
| Zic2 | Rattus norvegicus Zic family member 2 | -2.08 |
| AF146738 | Rattus norvegicus testis specific protein | -2.09 |
| Psip1 | Rattus norvegicus PC4 and SFRS1 interacting protein 1 | -2.1 |
| Asrgl1 | Rattus norvegicus asparaginase-like sperm autoantigen | -2.1 |
| Pdgfa | Rattus norvegicus platelet derived growth factor, alpha | -2.1 |
| Cds1 | Rattus norvegicus CDP-diacylglycerol synthase 1 | -2.11 |
| Lrp11 | Rattus norvegicus low density lipoprotein receptor-related protein 11 | -2.11 |
| Sdccag3 | Rattus norvegicus serologically defined colon cancer antigen 3 | -2.12 |
| Maob | Rattus norvegicus monoamine oxidase B | -2.12 |
| Tf | Rattus norvegicus Transferrin | -2.12 |
| Slc27a1 | Rattus norvegicus solute carrier family 27 | -2.14 |
| Slc15a4 | Rattus norvegicus peptide/histidine transporter | -2.14 |
| LOC306991 | Rattus norvegicus similar to Vps41 protein | -2.14 |
| RGD1305061 | Rattus norvegicus similar to RIKEN cDNA 2700055K07 | -2.14 |
| Snx25 | Rattus norvegicus sorting nexin 25 | -2.15 |
| Gnpat | Rattus norvegicus glyceronephosphate O-acyltransferase | -2.15 |
| MGC94018 | Rattus norvegicus glycosyltransferase AD-017 | -2.15 |
| LOC317396 | Rattus norvegicus similar to Ubiquilin 2 | -2.15 |
| Rarres1 | Rattus norvegicus retinoic acid receptor responder | -2.16 |
| LOC499513 | Rattus norvegicus LOC499513 | -2.16 |
| LOC308820 | Rattus norvegicus similar to RIKEN cDNA 2310015N07 | -2.16 |
| Yme1l1 | Rattus norvegicus YME1-like 1 | -2.16 |
| Myh14 | Rattus norvegicus myosin, heavy polypeptide 14 | -2.17 |
| Smoc2 | Rattus norvegicus SPARC related modular calcium binding 2 | -2.18 |
| LOC500671 | Rattus norvegicus similar to chromosome 14 open reading frame 135 | -2.18 |
| RGD1311364 | Rattus norvegicus similar to RIKEN cDNA 1810021J13 | -2.18 |
| LOC299209 | Rattus norvegicus similar to 1700019E19Rik protein | -2.18 |
| LOC304289 | Rattus norvegicus similar to KCCR13L | -2.19 |
| Sirt2 | Rattus norvegicus sirtuin | -2.19 |
| LOC294942 | Rattus norvegicus hypothetical LOC294942 | -2.19 |
| Rnpep | Rattus norvegicus arginyl aminopeptidase | -2.19 |
| LOC313842 | Rattus norvegicus similar to 2810036L13Rik protein | -2.21 |
| LOC290851 | Rattus norvegicus similar to RIKEN cDNA 2210415M20 | -2.21 |
| Paqr4 | Rattus norvegicus progestin and adipoQ receptor family member IV | -2.22 |
| Gpm6a | Rattus norvegicus glycoprotein m6a | -2.22 |
| Dcn | Rattus norvegicus decorin | -2.22 |
| Mpp6 | Rattus norvegicus membrane protein, palmitoylated 6 | -2.24 |
| Cte1 | Rattus norvegicus cytosolic acyl-CoA thioesterase 1 | -2.25 |
| Sdccag8 | Rattus norvegicus slinky | -2.26 |
| Serpinb1a | Rattus norvegicus serine | -2.26 |
| LOC303238 | Rattus norvegicus similar to novel protein | -2.27 |
| Klf5 | Rattus norvegicus Kruppel-like factor 5 | -2.27 |
| Sema3b | Rattus norvegicus sema domain, immunoglobulin domain | -2.27 |
| Glrx2 | Rattus norvegicus glutaredoxin 2 | -2.27 |
| Omd | Rattus norvegicus osteomodulin | -2.28 |
| Aqp4 | Rattus norvegicus aquaporin 4 | -2.28 |
| Plekhb1 | Rattus norvegicus evectin-1 | -2.29 |
| Cdo1 | Rattus norvegicus cysteine dioxygenase 1, cytosolic | -2.31 |
| LOC500856 | Rattus norvegicus hypothetical gene supported by BC087105 | -2.31 |
| Gpc3 | Rattus norvegicus glypican 3 | -2.33 |
| LOC360819 | Rattus norvegicus similar to FLJ00052 protein | -2.34 |
| LOC498278 | Rattus norvegicus similar to RIKEN cDNA 1700009P17 | -2.34 |
| Rtn1 | Rattus norvegicus reticulon 1 | -2.34 |
| Aard | Rattus norvegicus alanine and arginine rich domain containing protein | -2.35 |
| Scg3 | Rattus norvegicus secretogranin III | -2.35 |
| Parva | Rattus norvegicus parvin, alpha | -2.36 |
| RGD1311155 | Rattus norvegicus similar to RIKEN cDNA 9230117N10 | -2.37 |
| Alcam | Rattus norvegicus activated leukocyte cell adhesion molecule | -2.37 |
| Stk39 | Rattus norvegicus serine/threonine kinase 39, STE20/SPS1 homolog | -2.38 |
| Loxl2 | Rattus norvegicus lysyl oxidase-like 2 | -2.39 |
| MGC93902 | Rattus norvegicus similar to RIKEN cDNA 2610028I09 | -2.4 |
| Camk2n1 | Rattus norvegicus calcium/calmodulin-dependent protein kinase II inhibitor 1 | -2.4 |
| Rpl15 | Rattus norvegicus ribosomal protein L15 | -2.41 |
| LOC294734 | Rattus norvegicus similar to RIKEN cDNA 1700034P14 | -2.42 |
| Abhd3 | Rattus norvegicus abhydrolase domain containing 3 | -2.42 |
| Gap43 | Rattus norvegicus growth associated protein 43 | -2.44 |
| Trim39 | Rattus norvegicus tripartite motif protein 39 | -2.45 |
| Cspg5 | Rattus norvegicus chondroitin sulfate proteoglycan 5 | -2.45 |
| LOC302863 | Rattus norvegicus similar to mKIAA0267 protein | -2.46 |
| Nrp1 | Rattus norvegicus neuropilin 1 | -2.46 |
| LOC301521 | Rattus norvegicus similar to hypothetical protein DKFZp434O0527 | -2.47 |
| Fxna | Rattus norvegicus putative aminopeptidase Fxna | -2.47 |
| Sil1 | Rattus norvegicus endoplasmic reticulum chaperone SIL1 homolog | -2.49 |
| C4-2 | Rattus norvegicus complement component 4, gene 2 | -2.49 |
| Calm1 | Rattus norvegicus calmodulin 1 | -2.49 |
| Edg2 | Rattus norvegicus endothelial differentiation, lysophosphatidic acid G-protein-coupled receptor, 2 | -2.51 |
| LOC317444 | Rattus norvegicus similar to Hccs protein | -2.53 |
| Igbp1 | Rattus norvegicus immunoglobulin | -2.54 |
| Tfpi | Rattus norvegicus tissue factor pathway inhibitor | -2.54 |
| Tec | Rattus norvegicus tec protein tyrosine kinase | -2.56 |
| Erp29 | Rattus norvegicus endoplasmic retuclum protein 29 | -2.56 |
| Gprasp1 | Rattus norvegicus G protein-coupled receptor associated sorting protein 1 | -2.57 |
| Adm | Rattus norvegicus adrenomedullin | -2.57 |
| LOC292477 | Rattus norvegicus similar to OTTHUMP00000040155 | -2.58 |
| Ppt | Rattus norvegicus palmitoyl-protein thioesterase | -2.6 |
| LOC503409 | Rattus norvegicus similar to Ac1147 | -2.61 |
| Epb4.1l3 | Rattus norvegicus erythrocyte protein band 4.1-like 3 | -2.61 |
| LOC499589 | Rattus norvegicus similar to hypothetical protein MGC27085 | -2.62 |
| RGD1305524 | Rattus norvegicus similar to hypothetical protein FLJ12442 | -2.62 |
| LOC366872 | Rattus norvegicus similar to RIKEN cDNA 4921537D05 | -2.63 |
| LOC365389 | Rattus norvegicus similar to RIKEN cDNA 5730427C23 | -2.63 |
| Tcfap2b | Rattus norvegicus transcription factor AP-2 beta | -2.64 |
| Gmnn | Rattus norvegicus geminin | -2.65 |
| Ltbp4 | Rattus norvegicus latent transforming growth factor beta binding protein 4 | -2.66 |
| Rhpn1 | Rattus norvegicus rhophilin, Rho GTPase binding protein 1 | -2.67 |
| Cyp26b1 | Rattus norvegicus cytochrome P450, family 26, subfamily b, polypeptide 1 | -2.69 |
| Spag8 | Rattus norvegicus sperm associated antigen 8 | -2.77 |
| Pcm1 | Rattus norvegicus pericentriolar material 1 | -2.77 |
| Dnai2 | Rattus norvegicus dynein, axonemal, intermediate polypeptide 2 | -2.82 |
| Cfh | Rattus norvegicus complement component factor H | -2.83 |
| LOC499856 | Rattus norvegicus similar to RIKEN cDNA 1110018M03 | -2.85 |
| Gpr37l1 | Rattus norvegicus G protein-coupled receptor 37-like 1 | -2.85 |
| Dnah1 | Rattus norvegicus dynein, axonemal, heavy polypeptide 1 | -2.88 |
| Zmynd10 | Rattus norvegicus zinc finger, MYND domain-containing 10 | -2.9 |
| RGD1306222 | Rattus norvegicus similar to 1810034B16Rik protein | -2.92 |
| Sesn1 | Rattus norvegicus sestrin 1 | -2.94 |
| Igsf1 | Rattus norvegicus immunoglobulin superfamily, member 1 | -2.95 |
| LOC317575 | Rattus norvegicus similar to Smarca1 protein | -2.99 |
| Strbp | Rattus norvegicus double-stranded RNA-binding protein p74 | -2.99 |
| LOC503278 | Rattus norvegicus similar to testin | -2.99 |
| Egr2 | Rattus norvegicus early growth response 2 | -3.01 |
| Csad | Rattus norvegicus cysteine sulfinic acid decarboxylase | -3.05 |
| LOC499839 | Rattus norvegicus similar to LOC387763 protein | -3.1 |
| Tekt1 | Rattus norvegicus tektin 1 | -3.1 |
| LOC300517 | Rattus norvegicus similar to hypothetical protein FLJ25530 | -3.11 |
| LOC499268 | Rattus norvegicus similar to Gm166 protein | -3.12 |
| RGD1308075 | Rattus norvegicus similar to hypothetical protein MGC27019 | -3.14 |
| LOC313436 | Rattus norvegicus similar to RIKEN cDNA 2810028A01 | -3.14 |
| Sez6 | Rattus norvegicus seizure related 6 homolog | -3.18 |
| Gpr51 | Rattus norvegicus G protein-coupled receptor 51 | -3.19 |
| Dncl2b | Rattus norvegicus dynein, cytoplasmic, light chain 2B | -3.19 |
| LOC498356 | Rattus norvegicus similar to MGC68837 protein | -3.22 |
| LOC362809 | Rattus norvegicus similar to Sid3177p | -3.23 |
| LOC296608 | Rattus norvegicus similar to hypothetical protein MGC29761 | -3.36 |
| Mlf1 | Rattus norvegicus myeloid leukemia factor 1 | -3.36 |
| LOC500416 | Rattus norvegicus LOC500416 | -3.36 |
| Lrpb7 | Rattus norvegicus leucine rich protein, B7 gene | -3.36 |
| Efemp1 | Rattus norvegicus epidermal growth factor-containing fibulin-like extracellular matrix protein 1 | -3.39 |
| LOC500939 | Rattus norvegicus LOC500939 | -3.4 |
| Giot1 | Rattus norvegicus gonadotropin inducible ovarian transcription factor 1 | -3.42 |
| Prelp | Rattus norvegicus proline arginine-rich end leucine-rich repeat protein | -3.42 |
| LOC287346 | Rattus norvegicus similar to novel protein | -3.46 |
| Aurkb | Rattus norvegicus aurora kinase B | -3.46 |
| Khdrbs3 | Rattus norvegicus etoile, Sam68-like protein SLM-2 | -3.55 |
| Dnaja4 | Rattus norvegicus DnaJ | -3.55 |
| Itgb4 | Rattus norvegicus integrin beta 4 | -3.56 |
| Cldn11 | Rattus norvegicus claudin 11 | -3.56 |
| A2m | Rattus norvegicus alpha-2-macroglobulin | -3.64 |
| Fank1 | Rattus norvegicus fibronectin type 3 and ankyrin repeat domains 1 | -3.73 |
| LOC291847 | Rattus norvegicus similar to hypothetical protein 4933409I22 | -3.81 |
| MGC105647 | Rattus norvegicus similar to Nur77 downstream protein 2 | -3.81 |
| Arhgdig | Rattus norvegicus Rho GDP dissociation inhibitor | -3.89 |
| Sostdc1 | Rattus norvegicus uterine sensitization-associated gene 1 protein | -3.9 |
| Olig1 | Rattus norvegicus oligodendrocyte transcription factor 1 | -3.92 |
| LOC293156 | Rattus norvegicus similar to Hypothetical 55.1 kDa protein F09G8.5 in chromosome III | -4.01 |
| Esm1 | Rattus norvegicus endothelial cell-specific molecule 1 | -4.13 |
| Fez1 | Rattus norvegicus fasciculation and elongation protein zeta 1 | -4.18 |
| Atp1a2 | Rattus norvegicus ATPase, Na+/K+ transporting, alpha 2 polypeptide | -4.23 |
| OSP94 | Rattus norvegicus osmotic stress protein 94 kDa | -4.24 |
| LOC294789 | Rattus norvegicus similar to Hypothetical protein FLJ25422 | -4.41 |
| LOC290372 | Rattus norvegicus similar to expressed sequence AU021034 | -4.62 |
| LOC498982 | Rattus norvegicus similar to Myb proto-oncogene protein | -4.62 |
| Calb1 | Rattus norvegicus calbindin 1 | -4.65 |
| LOC360747 | Rattus norvegicus similar to axoneme central apparatus protein | -4.66 |
| Mycl1 | Rattus norvegicus v-myc myelocytomatosis viral oncogene homolog 1, lung carcinoma derived | -4.68 |
| Bmp7 | Rattus norvegicus bone morphogenetic protein 7 | -4.77 |
| LOC310926 | Rattus norvegicus similar to Ac1147 | -5.15 |
| Gjb2 | Rattus norvegicus gap junction membrane channel protein beta 2 | -5.43 |
| Enpp2 | Rattus norvegicus ectonucleotide pyrophosphatase/phosphodiesterase 2 | -5.52 |
| LOC365476 | Rattus norvegicus similar to chromosome 10 open reading frame 79 | -5.54 |
| Ptgs2 | Rattus norvegicus prostaglandin-endoperoxide synthase 2 | -6.05 |
| Ptgds | Rattus norvegicus prostaglandin D2 synthase | -7.17 |
