# Supplementary material for: Multiple Oxygen Tension Environments Reveal Diverse Patterns of Transcriptional Regulation in Primary Astrocytes
Source: PLoS One. 2011 Jun 27;6(6):e21638. doi: 10.1371/journal.pone.0021638 (PMC3124552; doi:10.1371/journal.pone.0021638)
Supplement: Table S3 — Significantly regulated (p<0.05) genes in rat primary astrocytes exposed to 9% ambient O2 tension compared to 20% O2 tension. Z ratios were calculated as described in Materials and Methods. (DOC) [file pone.0021638.s009.doc]

**Table S3. Significantly regulated (p<0.05) genes in rat primary astrocytes exposed to 9% ambient O_2_ tension compared to 20% O_2_ tension**. Z ratios were calculated as described in Materials and Methods.

| **Symbol** | **Gene definition** | **z ratio** |
| --- | --- | --- |
| Eln | Rattus norvegicus elastin | 13.01 |
| Fstl3 | Rattus norvegicus follistatin-like 3 | 5.5 |
| Ca3 | Rattus norvegicus carbonic anhydrase 3 | 5.19 |
| Mfap4 | Rattus norvegicus microfibrillar-associated protein 4 | 4.49 |
| Ptgis | Rattus norvegicus prostaglandin I2 | 4.14 |
| Snai1 | Rattus norvegicus snail homolog, | 4.13 |
| Hes1 | Rattus norvegicus hairy and enhancer of split 1 | 3.94 |
| Eno1 | Rattus norvegicus enolase 1, alpha | 3.89 |
| Thrsp | Rattus norvegicus thyroid hormone responsive protein | 3.73 |
| Tpi1 | Rattus norvegicus triosephosphate isomerase 1 | 3.66 |
| Acta1 | Rattus norvegicus actin, alpha 1, skeletal muscle | 3.52 |
| Cmkor1 | Rattus norvegicus chemokine orphan receptor 1 | 3.49 |
| LOC498731 | Rattus norvegicus similar to Tpi1 protein | 3.4 |
| LOC295423 | Rattus norvegicus similar to glyceraldehyde-3-phosphate dehydrogenase | 3.37 |
| Rhoa | Rattus norvegicus ras homolog gene family, member A | 3.36 |
| LOC500965 | Rattus norvegicus similar to L-lactate dehydrogenase A chain | 3.35 |
| LOC307731 | Rattus norvegicus similar to L-lactate dehydrogenase A chain | 3.29 |
| LOC365954 | Rattus norvegicus similar to glyceraldehyde-3-phosphate dehydrogenase | 3.25 |
| Col14a1 | Rattus norvegicus collagen, type XIV, alpha 1 | 3.25 |
| Pgk1 | Rattus norvegicus phosphoglycerate kinase 1 | 3.18 |
| Abcb9 | Rattus norvegicus ATP-binding cassette, sub-family B | 3.17 |
| LOC364848 | Rattus norvegicus similar to Glyceraldehyde-3-phosphate dehydrogenase | 3.16 |
| LOC497936 | Rattus norvegicus similar to RIKEN cDNA 2600017H02 | 3.07 |
| Wisp2 | Rattus norvegicus WNT1 inducible signaling pathway protein 2 | 3.04 |
| LOC500959 | Rattus norvegicus similar to triosephosphate isomerase | 3.03 |
| Gapd | Rattus norvegicus glyceraldehyde-3-phosphate dehydrogenase | 3.03 |
| LOC311592 | Rattus norvegicus similar to hypothetical protein D630003M21 | 3 |
| LOC501605 | Rattus norvegicus similar to 40S ribosomal protein S2 | 2.98 |
| LOC301438 | Rattus norvegicus similar to 40S ribosomal protein S2 | 2.87 |
| LOC498881 | Rattus norvegicus similar to glyceraldehyde-3-phosphate dehydrogenase | 2.83 |
| LOC364048 | Rattus norvegicus similar to macrophage migration inhibitory factor | 2.77 |
| Fdps | Rattus norvegicus farensyl diphosphate synthase | 2.75 |
| Cerk | Rattus norvegicus ceramide kinase | 2.75 |
| LOC498099 | Rattus norvegicus similar to glyceraldehyde-3-phosphate dehydrogenase | 2.73 |
| MGC95138 | Rattus norvegicus similar to acetyl CoA transferase-like | 2.72 |
| LOC499433 | Rattus norvegicus similar to glyceraldehyde-3-phosphate dehydrogenase | 2.72 |
| Tm4sf1 | Rattus norvegicus transmembrane 4 superfamily member 1 | 2.71 |
| LOC500104 | Rattus norvegicus similar to Glyceraldehyde-3-phosphate dehydrogenase | 2.7 |
| Scd1 | Rattus norvegicus stearoyl-Coenzyme A desaturase 1 | 2.66 |
| LOC497841 | Rattus norvegicus hypothetical gene supported by NM_016994 | 2.65 |
| Ldha | Rattus norvegicus lactate dehydrogenase A | 2.65 |
| LOC500645 | Rattus norvegicus similar to 60S ribosomal protein L29 | 2.64 |
| LOC498068 | Rattus norvegicus similar to ribosomal protein S2 | 2.64 |
| Crabp1 | Rattus norvegicus cellular retinoic acid binding protein I | 2.63 |
| LOC500271 | Rattus norvegicus similar to macrophage migration inhibitory factor | 2.63 |
| Dspg3 | Rattus norvegicus dermatan sulphate proteoglycan 3 | 2.62 |
| LOC498406 | Rattus norvegicus similar to mitochondrial ribosomal protein S24 | 2.61 |
| Serpine2 | Rattus norvegicus serine | 2.61 |
| LOC302388 | Rattus norvegicus similar to ribosomal protein L19 | 2.54 |
| Lum | Rattus norvegicus lumican | 2.51 |
| LOC290634 | Rattus norvegicus similar to Glyceraldehyde-3-phosphate dehydrogenase | 2.49 |
| P4hb | Rattus norvegicus prolyl 4-hydroxylase, beta polypeptide | 2.49 |
| Igfbp3 | Rattus norvegicus insulin-like growth factor binding protein 3 | 2.46 |
| LOC498019 | Rattus norvegicus similar to glyceraldehyde-3-phosphate dehydrogenase | 2.45 |
| Fbl | Rattus norvegicus fibrillarin | 2.44 |
| Hist1h2bp | Rattus norvegicus histone 1, H2bp | 2.44 |
| Foxg1 | Rattus norvegicus forkhead box G1 | 2.43 |
| Scand1 | Rattus norvegicus SCAN domain-containing 1 | 2.43 |
| Stmn2 | Rattus norvegicus stathmin-like 2 | 2.42 |
| LOC310585 | Rattus norvegicus similar to Eno1 protein | 2.42 |
| LOC296582 | Rattus norvegicus similar to ribosomal protein S2 | 2.42 |
| LOC500666 | Rattus norvegicus similar to dapper 1 | 2.4 |
| LOC367339 | Rattus norvegicus similar to 60S ribosomal protein L29 | 2.37 |
| Mapre3 | Rattus norvegicus microtubule-associated protein, RP/EB family, member 3 | 2.36 |
| Tnfrsf11b | Rattus norvegicus tumor necrosis factor receptor superfamily, member 11b | 2.36 |
| Mfge8 | Rattus norvegicus milk fat globule-EGF factor 8 protein | 2.36 |
| LOC292656 | Rattus norvegicus similar to Macrophage migration inhibitory factor | 2.34 |
| Gdf15 | Rattus norvegicus growth differentiation factor 15 | 2.34 |
| Gadd45b | Rattus norvegicus growth arrest and DNA-damage-inducible 45 beta | 2.33 |
| LOC310512 | Rattus norvegicus similar to expressed sequence C87860 | 2.32 |
| Casp8ap2 | Rattus norvegicus caspase 8 associated protein 2 | 2.3 |
| Smfn | Rattus norvegicus small fragment nuclease | 2.3 |
| Lancl2 | Rattus norvegicus LanC | 2.27 |
| LOC366999 | Rattus norvegicus similar to 60S ribosomal protein L29 | 2.26 |
| Flcn | Rattus norvegicus folliculin | 2.26 |
| Ppp1r3c | Rattus norvegicus protein phosphatase 1, regulatory | 2.26 |
| LOC294700 | Rattus norvegicus similar to ribosomal protein L21 | 2.25 |
| LOC500242 | Rattus norvegicus similar to Poly | 2.25 |
| LOC295452 | Rattus norvegicus similar to Glyceraldehyde-3-phosphate dehydrogenase | 2.25 |
| LOC290706 | Rattus norvegicus similar to 2700029M09Rik protein | 2.24 |
| Ubb | Rattus norvegicus polyubiquitin | 2.23 |
| Tax1bp1 | Rattus norvegicus Tax1 | 2.22 |
| Ppap2c | Rattus norvegicus phosphatidic acid phosphatase type 2c | 2.2 |
| LOC502770 | Rattus norvegicus similar to glyceraldehyde-3-phosphate dehydrogenase | 2.2 |
| LOC299907 | Rattus norvegicus similar to Ext1 | 2.19 |
| LOC299127 | Rattus norvegicus similar to RIKEN cDNA 1200003C05 | 2.18 |
| Col8a1 | Rattus norvegicus procollagen, type VIII, alpha 1 | 2.18 |
| RGD1306222 | Rattus norvegicus similar to 1810034B16Rik protein | 2.18 |
| Bsg | Rattus norvegicus basigin | 2.16 |
| Cd63 | Rattus norvegicus CD63 antigen | 2.15 |
| LOC500987 | Rattus norvegicus similar to Histone H2A.x | 2.15 |
| Tram1 | Rattus norvegicus translocation associated membrane protein 1 | 2.15 |
| Txndc5 | Rattus norvegicus thioredoxin domain containing 5 | 2.14 |
| Cct5 | Rattus norvegicus chaperonin subunit 5 | 2.13 |
| Akr1a1 | Rattus norvegicus aldo-keto reductase family 1, member A1 | 2.13 |
| Gadd45gip1 | Rattus norvegicus growth arrest and DNA-damage-inducible, gamma interacting protein 1 | 2.12 |
| 0610031j06rik | Rattus norvegicus kidney predominant protein NCU-G1 | 2.12 |
| Sod3 | Rattus norvegicus superoxide dismutase 3, extracellular | 2.11 |
| Pafah1b2 | Rattus norvegicus platelet-activating factor acetylhydrolase, isoform 1b, alpha2 subunit | 2.1 |
| Ass | Rattus norvegicus argininosuccinate synthetase | 2.08 |
| LOC308503 | Rattus norvegicus similar to Set beta isoform | 2.08 |
| Zfp207 | Rattus norvegicus zinc finger protein 207 | 2.08 |
| Apex1 | Rattus norvegicus apurinic/apyrimidinic endonuclease 1 | 2.07 |
| Ilvbl | Rattus norvegicus ilvB | 2.06 |
| LOC315329 | Rattus norvegicus similar to expressed sequence AW556797 | 2.06 |
| Bgn | Rattus norvegicus biglycan | 2.06 |
| Nfkbia | Rattus norvegicus nuclear factor of kappa light chain gene enhancer in B-cells inhibitor, alpha | 2.06 |
| Rae1 | Rattus norvegicus RAE1 RNA export 1 homolog | 2.05 |
| Nedd9 | Rattus norvegicus neural precursor cell expressed, developmentally down-regulated gene 9 | 2.05 |
| LOC303471 | Rattus norvegicus similar to Stathmin | 2.04 |
| Hmgcs1 | Rattus norvegicus 3-hydroxy-3-methylglutaryl-Coenzyme A synthase 1 | 2.03 |
| Tmepai | Rattus norvegicus transmembrane, prostate androgen induced RNA | 2.02 |
| LOC499125 | Rattus norvegicus LOC499125 | 2.01 |
| Aldoa | Rattus norvegicus aldolase A | 2.01 |
| LOC302500 | Rattus norvegicus similar to malignant T cell amplified sequence 1 | 2 |
| LOC498078 | Rattus norvegicus similar to 60S ribosomal protein L7a | 1.98 |
| Slc25a1 | Rattus norvegicus solute carrier family 25, member 1 | 1.98 |
| Ndn | Rattus norvegicus necdin | 1.98 |
| Ywhag | Rattus norvegicus tyrosine 3-monooxgenase/tryptophan 5-monooxgenase activation protein, gamma polypeptide | 1.97 |
| Fbxo33 | Rattus norvegicus F-box only protein 33 | 1.97 |
| Dab2ip | Rattus norvegicus disabled homolog 2 | 1.97 |
| Commd8 | Rattus norvegicus COMM domain containing 8 | 1.97 |
| Echdc1 | Rattus norvegicus enoyl Coenzyme A hydratase domain containing 1 | 1.97 |
| Cyr61 | Rattus norvegicus cysteine rich protein 61 | 1.97 |
| Exosc5 | Rattus norvegicus exosome component 5 | 1.96 |
| Gng11 | Rattus norvegicus guanine nucleotide binding protein | 1.95 |
| LOC497831 | Rattus norvegicus hypothetical gene supported by NM_175869 | 1.95 |
| LOC310360 | Rattus norvegicus similar to eukaryotic translation elongation factor 1 alpha 1 | 1.94 |
| Lcat | Rattus norvegicus lecithin cholesterol acyltransferase | 1.94 |
| LOC363865 | Rattus norvegicus similar to tumor protein, translationally-controlled 1 | 1.93 |
| LOC501536 | Rattus norvegicus similar to Heterogeneous nuclear ribonucleoprotein A1 | 1.93 |
| Crebl1 | Rattus norvegicus cAMP responsive element binding protein-like 1 | 1.93 |
| LOC500506 | Rattus norvegicus similar to glyceraldehyde-3-phosphate dehydrogenase | 1.93 |
| LOC299622 | Rattus norvegicus similar to glyceraldehyde-3-phosphate dehydrogenase | 1.92 |
| Lamr1 | Rattus norvegicus laminin receptor 1 | 1.92 |
| LOC289930 | Rattus norvegicus similar to tumor protein, translationally-controlled 1 | 1.91 |
| Gdf10 | Rattus norvegicus growth differentiation factor 10 | 1.91 |
| Pfkl | Rattus norvegicus phosphofructokinase, liver, B-type | 1.9 |
| Cx3cl1 | Rattus norvegicus chemokine | 1.89 |
| Plxdc2 | Rattus norvegicus plexin domain containing 2 | 1.89 |
| Rpl24 | Rattus norvegicus ribosomal protein L24 | 1.89 |
| Sfrs10 | Rattus norvegicus splicing factor, arginine/serine-rich 10 | 1.88 |
| LOC502063 | Rattus norvegicus LOC502063 | 1.88 |
| Ddit3 | Rattus norvegicus DNA-damage inducible transcript 3 | 1.87 |
| Znf297 | Rattus norvegicus zinc finger protein 297 | 1.87 |
| Rbm3 | Rattus norvegicus RNA binding motif | 1.87 |
| Loxl1 | Rattus norvegicus lysyl oxidase-like 1 | 1.87 |
| Tex264 | Rattus norvegicus testis expressed gene 264 homolog | 1.87 |
| Ttk | Rattus norvegicus Ttk protein kinase | 1.86 |
| MGC94190 | Rattus norvegicus similar to 0610007L01Rik protein | 1.86 |
| C1s | Rattus norvegicus complement component 1, s subcomponent | 1.86 |
| Hspa8 | Rattus norvegicus heat shock protein 8 | 1.86 |
| Colm | Rattus norvegicus collomin | 1.85 |
| Calr | Rattus norvegicus calreticulin | 1.85 |
| Pam | Rattus norvegicus peptidylglycine alpha-amidating monooxygenase | 1.84 |
| Rpl10a | Rattus norvegicus ribosomal protein L10A | 1.84 |
| Hmgn2 | Rattus norvegicus high mobility group protein 17 | 1.84 |
| Cte1 | Rattus norvegicus cytosolic acyl-CoA thioesterase 1 | 1.83 |
| Ptn | Rattus norvegicus pleiotrophin | 1.83 |
| Plod1 | Rattus norvegicus procollagen-lysine, 2-oxoglutarate 5-dioxygenase 1 | 1.83 |
| Fads1 | Rattus norvegicus fatty acid desaturase 1 | 1.83 |
| LOC294781 | Rattus norvegicus similar to 60S ribosomal protein L21 | 1.82 |
| Psmd5 | Rattus norvegicus proteasome | 1.82 |
| RGD1359127 | Rattus norvegicus similar to RIKEN cDNA 2310011J03 | 1.82 |
| Dpp7 | Rattus norvegicus dipeptidylpeptidase 7 | 1.82 |
| Bnip3 | Rattus norvegicus BCL2/adenovirus E1B 19 kDa-interacting protein 3 | 1.82 |
| Bri3 | Rattus norvegicus brain protein I3 | 1.82 |
| Dnclc1 | Rattus norvegicus dynein, cytoplasmic, light chain 1 | 1.82 |
| Lcn7 | Rattus norvegicus lipocalin 7 | 1.81 |
| Ebp | Rattus norvegicus phenylalkylamine Ca2+ antagonist | 1.81 |
| Asam | Rattus norvegicus adipocyte-specific adhesion molecule | 1.81 |
| LOC300278 | Rattus norvegicus similar to 40S ribosomal protein S9 | 1.81 |
| Ctsd | Rattus norvegicus cathepsin D | 1.81 |
| Eif4a1 | Rattus norvegicus eukaryotic translation initiation factor 4A1 | 1.81 |
| Tnnt2 | Rattus norvegicus troponin T2, cardiac | 1.8 |
| Smndc1 | Rattus norvegicus survival motor neuron domain containing 1 | 1.8 |
| Carhsp1 | Rattus norvegicus calcium regulated heat stable protein 1 | 1.8 |
| Rpp14 | Rattus norvegicus ribonuclease P 14kDa subunit | 1.8 |
| Apeh | Rattus norvegicus N-acylaminoacyl-peptide hydrolase | 1.8 |
| LOC498363 | Rattus norvegicus similar to 60S acidic ribosomal protein P2 | 1.8 |
| Rps2 | Rattus norvegicus ribosomal protein S2 | 1.8 |
| Atf5 | Rattus norvegicus activating transcription factor 5 | 1.79 |
| Slc1a3 | Rattus norvegicus solute carrier family 1 | 1.79 |
| Rabep1 | Rattus norvegicus rabaptin 5 | 1.78 |
| Mtvr2 | Rattus norvegicus mammary tumor virus receptor 2 | 1.78 |
| Lxn | Rattus norvegicus latexin | 1.78 |
| LOC366656 | Rattus norvegicus similar to ribosomal protein L10a | 1.78 |
| Rps10 | Rattus norvegicus ribosomal protein S10 | 1.78 |
| LOC306115 | Rattus norvegicus similar to glyceraldehyde-3-phosphate dehydrogenase | 1.77 |
| Slc37a4 | Rattus norvegicus solute carrier family 37 | 1.77 |
| Chdh | Rattus norvegicus choline dehydrogenase | 1.77 |
| Atf4 | Rattus norvegicus activating transcription factor 4 | 1.77 |
| Ranbp1 | Rattus norvegicus RAN binding protein 1 | 1.77 |
| Gabarap | Rattus norvegicus gamma-aminobutyric acid receptor associated protein | 1.77 |
| Tm4sf9 | Rattus norvegicus transmembrane 4 superfamily member 9 | 1.75 |
| Akr1b4 | Rattus norvegicus aldo-keto reductase family 1, member B4 | 1.75 |
| Pgls | Rattus norvegicus 6-phosphogluconolactonase | 1.74 |
| LOC503418 | Rattus norvegicus LOC503418 | 1.73 |
| MGC109491 | Rattus norvegicus similar to 1110007F12Rik protein | 1.73 |
| LOC362264 | Rattus norvegicus similar to dJ862K6.2.2 | 1.73 |
| Sdfr1 | Rattus norvegicus stromal cell derived factor receptor 1 | 1.72 |
| Ttc13 | Rattus norvegicus tetratricopeptide repeat domain 13 | 1.72 |
| Nme1 | Rattus norvegicus expressed in non-metastatic cells 1 | 1.72 |
| MGC94782 | Rattus norvegicus similar to hypothetical protein MGC33926 | 1.71 |
| LOC366689 | Rattus norvegicus similar to ribosomal protein L21 | 1.71 |
| LOC361571 | Rattus norvegicus similar to RIKEN cDNA 2410004H02 | 1.71 |
| Nde1 | Rattus norvegicus nuclear distribution gene E homolog 1 | 1.71 |
| Taf10 | Rattus norvegicus TAF10 RNA polymerase II, TATA box binding protein | 1.71 |
| LOC499423 | Rattus norvegicus similar to pyruvate kinase | 1.71 |
| MGC94233 | Rattus norvegicus similar to RIKEN cDNA 6720485C15 | 1.71 |
| Ddit4 | Rattus norvegicus DNA-damage-inducible transcript 4 | 1.7 |
| LOC292588 | Rattus norvegicus similar to Ubiquitin-conjugating enzyme E2S | 1.7 |
| C1qbp | Rattus norvegicus complement component 1, q subcomponent binding protein | 1.7 |
| Rpl13 | Rattus norvegicus ribosomal protein L13 | 1.7 |
| Ubtd1 | Rattus norvegicus ubiquitin domain containing 1 | 1.69 |
| Sfrs2 | Rattus norvegicus similar to splicing factor, arginine/serine-rich 2 | 1.69 |
| Hspca | Rattus norvegicus heat shock protein 1, alpha | 1.69 |
| LOC302497 | Rattus norvegicus similar to ribosomal protein L10a | 1.68 |
| Eif3s5 | Rattus norvegicus eukaryotic translation initiation factor 3, subunit 5 | 1.68 |
| Faf1 | Rattus norvegicus Fas-associated factor 1 | 1.67 |
| LOC366485 | Rattus norvegicus similar to ribosomal protein L36 | 1.67 |
| Gpi | Rattus norvegicus glucose phosphate isomerase | 1.67 |
| LOC497882 | Rattus norvegicus similar to ribosomal protein S10 | 1.67 |
| LOC498375 | Rattus norvegicus similar to RIKEN cDNA 4930555G01 | 1.66 |
| Tomm40 | Rattus norvegicus translocase of outer mitochondrial membrane 40 | 1.66 |
| Bambi | Rattus norvegicus BMP and activin membrane-bound inhibitor, homolog | 1.66 |
| Uap1l1 | Rattus norvegicus UDP-N-acteylglucosamine pyrophosphorylase 1-like 1 | 1.66 |
| Wdr34 | Rattus norvegicus WD repeat domain 34 | 1.66 |
| LOC316539 | Rattus norvegicus similar to Eph receptor A4 | 1.66 |
| Lactb | Rattus norvegicus lactamase, beta | 1.66 |
| Map2k3 | Rattus norvegicus mitogen activated protein kinase kinase 3 | 1.66 |
| LOC499845 | Rattus norvegicus similar to ribosomal protein L21 | 1.66 |
| Tcn2 | Rattus norvegicus transcobalamin 2 | 1.65 |
| Phax | Rattus norvegicus phosphorylated adaptor for RNA export | 1.65 |
| Ppp1r14b | Rattus norvegicus protein phosphatase 1, regulatory | 1.65 |
| LOC501140 | Rattus norvegicus similar to BCL2/adenovirus E1B 19 kDa-interacting protein 3 | 1.64 |
| LOC497927 | Rattus norvegicus similar to Phosphoglycerate mutase 1 | 1.64 |
| Mtch2 | Rattus norvegicus mitochondrial carrier homolog 2 | 1.64 |
| Arpc1b | Rattus norvegicus actin related protein 2/3 complex, subunit 1B | 1.64 |
| Prc1 | Rattus norvegicus protein regulator of cytokinesis 1 | 1.64 |
| LOC363531 | Rattus norvegicus similar to 40S ribosomal protein S19 | 1.64 |
| Dnajb11 | Rattus norvegicus DnaJ | 1.63 |
| Aldh1a1 | Rattus norvegicus aldehyde dehydrogenase family 1, member A1 | 1.63 |
| Pdlim7 | Rattus norvegicus PDZ and LIM domain 7 | 1.63 |
| Apoe | Rattus norvegicus apolipoprotein E | 1.63 |
| Bcap29 | Rattus norvegicus B-cell receptor-associated protein BAP29 | 1.62 |
| Pfdn1 | Rattus norvegicus prefoldin 1 | 1.62 |
| LOC298495 | Rattus norvegicus similar to ribosomal protein L35a | 1.62 |
| LOC498618 | Rattus norvegicus similar to glyceraldehyde-3-phosphate dehydrogenase | 1.62 |
| Dirc2 | Rattus norvegicus disrupted in renal carcinoma 2 | 1.61 |
| Mk1 | Rattus norvegicus Mk1 protein | 1.61 |
| LOC501203 | Rattus norvegicus similar to Myosin regulatory light chain 2-A, smooth muscle isoform | 1.61 |
| RGD1304567 | Rattus norvegicus LOC362671 | 1.6 |
| LOC366411 | Rattus norvegicus similar to ribosomal protein S24 | 1.6 |
| LOC499882 | Rattus norvegicus similar to CREBBP/EP300 inhibitory protein 1 | 1.6 |
| LOC363443 | Rattus norvegicus similar to NDP | 1.6 |
| Gdi2 | Rattus norvegicus GDP dissociation inhibitor 2 | 1.6 |
| Rpl21 | Rattus norvegicus ribosomal protein L21 | 1.6 |
| Mig12 | Rattus norvegicus MID1 interacting G12-like protein | 1.59 |
| RGD1307627 | Rattus norvegicus similar to gp25L2 protein | 1.59 |
| LOC300760 | Rattus norvegicus similar to H3 histone, family 3B | 1.58 |
| Mphosph10 | Rattus norvegicus M-phase phosphoprotein 10 | 1.58 |
| Chchd3 | Rattus norvegicus coiled-coil-helix-coiled-coil-helix domain containing 3 | 1.58 |
| Cct6a | Rattus norvegicus chaperonin subunit 6a | 1.58 |
| A2m | Rattus norvegicus alpha-2-macroglobulin | 1.58 |
| Ddx46 | Rattus norvegicus RNA helicase | 1.57 |
| Enpp1 | Rattus norvegicus ectonucleotide pyrophosphatase/phosphodiesterase 1 | 1.57 |
| LOC365416 | Rattus norvegicus similar to ribosomal protein L21 | 1.57 |
| Maob | Rattus norvegicus monoamine oxidase B | 1.57 |
| LOC313722 | Rattus norvegicus similar to SPRY domain-containing SOCS box protein SSB-1 | 1.57 |
| LOC361578 | Rattus norvegicus similar to pM5 protein; DNA segment, Chr 7, ERATO Doi 156, expressed | 1.57 |
| Qdpr | Rattus norvegicus quinoid dihydropteridine reductase | 1.56 |
| Fosl1 | Rattus norvegicus fos-like antigen 1 | 1.56 |
| LOC302671 | Rattus norvegicus similar to Adapter-related protein complex 1 sigma 1B subunit | 1.56 |
| Mdk | Rattus norvegicus midkine | 1.56 |
| LOC500795 | Rattus norvegicus LOC500795 | 1.55 |
| RGD1310571 | Rattus norvegicus similar to hypothetical protein | 1.55 |
| Ruvbl1 | Rattus norvegicus RuvB-like protein 1 | 1.55 |
| Ddx21b | Rattus norvegicus DEAD | 1.55 |
| LOC499512 | Rattus norvegicus similar to ATP synthase, H+ transporting, mitochondrial F0 complex, subunit c | 1.55 |
| Cox6a1 | Rattus norvegicus cytochrome c oxidase, subunit VIa, polypeptide 1 | 1.55 |
| Rpl32 | Rattus norvegicus ribosomal protein L32 | 1.55 |
| Hmmr | Rattus norvegicus hyaluronan mediated motility receptor | 1.54 |
| LOC299823 | Rattus norvegicus similar to ribosomal protein S10 | 1.54 |
| Tp53rk | Rattus norvegicus TP53 regulating kinase | 1.54 |
| Serpine1 | Rattus norvegicus serine | 1.54 |
| Cd164l1 | Rattus norvegicus CD164 sialomucin-like 1 | 1.54 |
| Pbp | Rattus norvegicus phosphatidylethanolamine binding protein | 1.54 |
| Hspb1 | Rattus norvegicus heat shock 27kDa protein 1 | 1.54 |
| LOC362513 | Rattus norvegicus similar to Shb protein | 1.53 |
| LOC501007 | Rattus norvegicus similar to RIKEN cDNA 6030419C18 gene | 1.53 |
| Pcolce | Rattus norvegicus procollagen C-proteinase enhancer protein | 1.53 |
| Mdh1 | Rattus norvegicus malate dehydrogenase 1, NAD | 1.53 |
| LOC497816 | Rattus norvegicus hypothetical gene supported by NM_019371 | 1.52 |
| LOC361178 | Rattus norvegicus similar to transcription factor | 1.52 |
| LOC292780 | Rattus norvegicus similar to hypothetical protein MGC15677 | 1.51 |
| Rara | Rattus norvegicus retinoic acid receptor, alpha | 1.51 |
| Anp32a | Rattus norvegicus acidic | 1.51 |
| Psmc3 | Rattus norvegicus proteasome | 1.51 |
| LOC366258 | Rattus norvegicus similar to 60S ribosomal protein L7a | 1.51 |
| LOC304638 | Rattus norvegicus similar to RIKEN cDNA 3110001N18 | 1.51 |
| Vars2 | Rattus norvegicus valyl-tRNA synthetase 2 | 1.5 |
| Emp3 | Rattus norvegicus epithelial membrane protein 3 | 1.5 |
| RGD1311463 | Rattus norvegicus similar to RIKEN cDNA 2700007P21 | -1.5 |
| RGD1310143 | Rattus norvegicus similar to RIKEN cDNA D030028O16 | -1.5 |
| LOC288515 | Rattus norvegicus similar to FLJ23471 protein | -1.5 |
| Ndrg2 | Rattus norvegicus N-myc downstream regulated gene 2 | -1.5 |
| Dpp3 | Rattus norvegicus dipeptidylpeptidase 3 | -1.51 |
| Wsb1 | Rattus norvegicus WD repeat and SOCS box-containing 1 | -1.51 |
| Gstm2 | Rattus norvegicus glutathione S-transferase, mu 2 | -1.51 |
| RGD1307700 | Rattus norvegicus similar to hypothetical protein BC018453 | -1.52 |
| LOC287452 | Rattus norvegicus similar to RIKEN cDNA 1110020A23 | -1.52 |
| MGC94053 | Rattus norvegicus similar to RECS1 | -1.52 |
| Psmd13 | Rattus norvegicus proteasome | -1.52 |
| Klf4 | Rattus norvegicus Kruppel-like factor 4 | -1.52 |
| Serpinb1a | Rattus norvegicus serine | -1.52 |
| Enh | Rattus norvegicus enigma homolog | -1.53 |
| LOC500042 | Rattus norvegicus similar to RIKEN cDNA 2610101N10 | -1.53 |
| Actn4 | Rattus norvegicus actinin alpha 4 | -1.53 |
| Gusb | Rattus norvegicus glucuronidase, beta | -1.53 |
| LOC306007 | Rattus norvegicus similar to 2610301G19Rik protein | -1.53 |
| LOC499323 | Rattus norvegicus similar to CCTeta, eta subunit of the chaperonin containing TCP-1 | -1.53 |
| Lamb1-1 | Rattus norvegicus laminin B1 subunit 1 | -1.53 |
| Mcm3ap | Rattus norvegicus minichromosome maintenance deficient 3 | -1.54 |
| LOC497834 | Rattus norvegicus hypothetical gene supported by NM_031053 | -1.54 |
| Smad1 | Rattus norvegicus MAD homolog 1 | -1.54 |
| RGD1306020 | Rattus norvegicus similar to aspartyl beta-hydroxylase; calsequestrin-binding protein; 3110001L23Rik | -1.54 |
| Sec23ip | Rattus norvegicus SEC23 interacting protein | -1.54 |
| LOC500974 | Rattus norvegicus similar to CDNA sequence BC024479 | -1.54 |
| LOC360747 | Rattus norvegicus similar to axoneme central apparatus protein | -1.54 |
| Ddx47 | Rattus norvegicus DEAD | -1.54 |
| Cxcl1 | Rattus norvegicus chemokine | -1.54 |
| LOC499615 | Rattus norvegicus similar to RIKEN cDNA 2810489O06 | -1.54 |
| Arpp19 | Rattus norvegicus cAMP-regulated phosphoprotein 19 | -1.55 |
| Flnc | Rattus norvegicus filamin C, gamma | -1.55 |
| LOC299199 | Rattus norvegicus similar to YLP motif containing protein 1 | -1.56 |
| LOC498276 | Rattus norvegicus similar to Fc gamma | -1.56 |
| Cd44 | Rattus norvegicus CD44 antigen | -1.56 |
| Nisch | Rattus norvegicus nischarin | -1.56 |
| Ddost | Rattus norvegicus dolichyl-di-phosphooligosaccharide-protein glycotransferase | -1.56 |
| Irf3 | Rattus norvegicus interferon regulatory factor 3 | -1.56 |
| Snrpd2 | Rattus norvegicus small nuclear ribonucleoprotein D2 | -1.56 |
| Giot1 | Rattus norvegicus gonadotropin inducible ovarian transcription factor 1 | -1.57 |
| LOC364468 | Rattus norvegicus similar to TGF beta-inducible nuclear protein 1 | -1.57 |
| Gnpat | Rattus norvegicus glyceronephosphate O-acyltransferase | -1.57 |
| Tf | Rattus norvegicus Transferrin | -1.57 |
| Sult1a1 | Rattus norvegicus sulfotransferase family 1A, phenol-preferring, member 1 | -1.58 |
| LOC500504 | Rattus norvegicus LOC500504 | -1.58 |
| Ddx23 | Rattus norvegicus DEAD | -1.58 |
| LOC305452 | Rattus norvegicus hypothetical LOC305452 | -1.58 |
| LOC498095 | Rattus norvegicus similar to RIKEN cDNA 0610012D17 | -1.58 |
| Tec | Rattus norvegicus tec protein tyrosine kinase | -1.58 |
| Cox17 | Rattus norvegicus cytochrome c oxidase, subunit XVII assembly protein homolog | -1.59 |
| LOC499856 | Rattus norvegicus similar to RIKEN cDNA 1110018M03 | -1.59 |
| Sphk1 | Rattus norvegicus sphingosine kinase 1 | -1.59 |
| Luc7l | Rattus norvegicus LUC7-like | -1.6 |
| Wbp11 | Rattus norvegicus WW domain binding protein 11 | -1.6 |
| Waspip | Rattus norvegicus Wiskott-Aldrich syndrome protein interacting protein | -1.6 |
| LOC498388 | Rattus norvegicus similar to High mobility group protein 2 | -1.6 |
| Nqo1 | Rattus norvegicus NAD | -1.6 |
| LOC362840 | Rattus norvegicus LOC362840 | -1.61 |
| Abca2 | Rattus norvegicus ATP-binding cassette, sub-family A | -1.61 |
| Myh14 | Rattus norvegicus myosin, heavy polypeptide 14 | -1.61 |
| RGD1309382 | Rattus norvegicus similar to RIKEN cDNA C730048E16 | -1.61 |
| Csad | Rattus norvegicus cysteine sulfinic acid decarboxylase | -1.61 |
| RT1-A1 | Rattus norvegicus RT1 class Ia, locus A1 | -1.62 |
| Lrp16 | Rattus norvegicus LRP16 protein | -1.62 |
| MGC72974 | Rattus norvegicus Unknown | -1.63 |
| LOC296469 | Rattus norvegicus similar to chromosome 20 open reading frame 58 | -1.63 |
| Loc65027 | Rattus norvegicus beta-catenin binding protein | -1.63 |
| Lpl | Rattus norvegicus lipoprotein lipase | -1.63 |
| Lancl1 | Rattus norvegicus lanC | -1.63 |
| LOC315216 | Rattus norvegicus hypothetical LOC315216 | -1.64 |
| Acyp1 | Rattus norvegicus acylphosphatase 1, erythrocyte | -1.64 |
| LOC500829 | Rattus norvegicus similar to ORF2 consensus sequence encoding endonuclease and reverse transcriptase minus RNaseH | -1.64 |
| LOC361117 | Rattus norvegicus similar to LRRGT00149 | -1.65 |
| Ctbs | Rattus norvegicus chitobiase, di-N-acetyl- | -1.65 |
| MGC94954 | Rattus norvegicus similar to RIKEN cDNA 2310042P20 | -1.65 |
| Ifi27l | Rattus norvegicus interferon, alpha-inducible protein 27-like | -1.65 |
| Tnn | Rattus norvegicus tenascin N | -1.65 |
| LOC289437 | Rattus norvegicus similar to Glomulin | -1.66 |
| MGC105797 | Rattus norvegicus similar to ubiquitously-expressed transcript isoform 1 | -1.66 |
| Nup107 | Rattus norvegicus nucleoporin 107 | -1.66 |
| Nr2f1 | Rattus norvegicus nuclear receptor subfamily 2, group F, member 1 | -1.66 |
| Cpd | Rattus norvegicus carboxypeptidase D | -1.66 |
| Edg2 | Rattus norvegicus endothelial differentiation, lysophosphatidic acid G-protein-coupled receptor, 2 | -1.66 |
| Tex27 | Rattus norvegicus testis expressed gene 27 | -1.66 |
| Rda279 | Rattus norvegicus hypothetical protein RDA279 | -1.67 |
| Polr2b | Rattus norvegicus polymerase | -1.67 |
| LOC291750 | Rattus norvegicus similar to TRS85 homolog | -1.67 |
| Snx14 | Rattus norvegicus sorting nexin 14 | -1.67 |
| Vps16 | Rattus norvegicus vacuolar protein sorting 16 | -1.67 |
| Catns | Rattus norvegicus catenin src | -1.67 |
| Ndufb7 | Rattus norvegicus NADH dehydrogenase | -1.67 |
| Pla2g6 | Rattus norvegicus phospholipase A2, group VI | -1.68 |
| Ndr4 | Rattus norvegicus N-myc downstream regulated 4 | -1.69 |
| Abhd3 | Rattus norvegicus abhydrolase domain containing 3 | -1.69 |
| Sod1 | Rattus norvegicus superoxide dismutase 1 | -1.69 |
| Nup54 | Rattus norvegicus nucleoporin 54 | -1.7 |
| Hmox1 | Rattus norvegicus heme oxygenase | -1.7 |
| Dhcr7 | Rattus norvegicus 7-dehydrocholesterol reductase | -1.7 |
| Tctex1 | Rattus norvegicus t-complex testis expressed 1 | -1.7 |
| Cln2 | Rattus norvegicus ceroid-lipofuscinosis, neuronal 2 | -1.7 |
| Txndc1 | Rattus norvegicus thioredoxin domain containing 1 | -1.71 |
| Usp7 | Rattus norvegicus ubiquitin specific protease 7 | -1.71 |
| LOC362490 | Rattus norvegicus similar to RIKEN cDNA 2610319K07 | -1.71 |
| Tmp21 | Rattus norvegicus transmembrane trafficking protein 21 | -1.71 |
| LOC305035 | Rattus norvegicus similar to D1Ertd396e protein | -1.72 |
| Cry1 | Rattus norvegicus cryptochrome 1 | -1.72 |
| Decr1 | Rattus norvegicus 2,4-dienoyl CoA reductase 1, mitochondrial | -1.72 |
| Col6a3 | Rattus norvegicus procollagen, type VI, alpha 3 | -1.72 |
| LOC315804 | Rattus norvegicus similar to hypothetical protein FLJ12994 | -1.73 |
| LOC502614 | Rattus norvegicus similar to RIKEN cDNA 2610205E22 | -1.73 |
| Lrrc5 | Rattus norvegicus leucine-rich repeat-containing 5 | -1.73 |
| Sugt1 | Rattus norvegicus SGT1, suppressor of G2 allele of SKP1 | -1.73 |
| Plekhb2 | Rattus norvegicus pleckstrin homology domain containing, family B | -1.73 |
| Frg1 | Rattus norvegicus FSHD region gene 1 | -1.74 |
| LOC361774 | Rattus norvegicus LOC361774 | -1.74 |
| LOC498228 | Rattus norvegicus similar to hypothetical protein DKFZp761N1114 | -1.74 |
| Slfn3 | Rattus norvegicus schlafen 3 | -1.75 |
| LOC363474 | Rattus norvegicus similar to RIKEN cDNA 0610008C08 | -1.75 |
| RGD1307010 | Rattus norvegicus similar to RIKEN cDNA 2700085E05 | -1.75 |
| Mdm2 | Rattus norvegicus transformed mouse 3T3 cell double minute 2 | -1.75 |
| Fam38a | Rattus norvegicus family with sequence similarity 38, member A | -1.76 |
| Mcm2 | Rattus norvegicus minichromosome maintenance deficient 2 mitotin | -1.76 |
| Pcnxl3 | Rattus norvegicus pecanex-like 3 | -1.76 |
| Ehmt1 | Rattus norvegicus euchromatic histone methyltransferase 1 | -1.76 |
| LOC317612 | Rattus norvegicus similar to HIV TAT specific factor 1 | -1.78 |
| Col18a1 | Rattus norvegicus collagen, type XVIII, alpha 1 | -1.78 |
| Gstm3 | Rattus norvegicus glutathione S-transferase, mu type 3 | -1.79 |
| Vcam1 | Rattus norvegicus vascular cell adhesion molecule 1 | -1.79 |
| Ltbp4 | Rattus norvegicus latent transforming growth factor beta binding protein 4 | -1.79 |
| Dhx30 | Rattus norvegicus DEAH | -1.8 |
| Mark3 | Rattus norvegicus MAP/microtubule affinity-regulating kinase 3 | -1.8 |
| Npr2 | Rattus norvegicus natriuretic peptide receptor 2 | -1.8 |
| B3gnt1 | Rattus norvegicus UDP-GlcNAc:betaGal beta-1,3-N-acetylglucosaminyltransferase 1 | -1.81 |
| Akap12 | Rattus norvegicus A kinase | -1.81 |
| Zfp330 | Rattus norvegicus zinc finger protein 330 | -1.81 |
| LOC309307 | Rattus norvegicus similar to KIAA2026 protein | -1.82 |
| Usp2 | Rattus norvegicus ubiquitin specific protease 2 | -1.82 |
| Olig1 | Rattus norvegicus oligodendrocyte transcription factor 1 | -1.82 |
| Itga7 | Rattus norvegicus integrin alpha 7 | -1.83 |
| MGC105508 | Rattus norvegicus similar to chromosome 6 open reading frame 83; similar to RIKEN cDNA 5630401J11 | -1.83 |
| Itgb1bp1 | Rattus norvegicus integrin beta 1 binding protein 1 | -1.83 |
| Hsd3b7 | Rattus norvegicus CCA2 protein | -1.83 |
| LOC303790 | Rattus norvegicus similar to RIKEN cDNA 4122402O22 | -1.83 |
| Magi3 | Rattus norvegicus membrane-associated guanylate kinase-related | -1.83 |
| Sparcl1 | Rattus norvegicus SPARC-like 1 | -1.83 |
| LOC500671 | Rattus norvegicus similar to chromosome 14 open reading frame 135 | -1.84 |
| Mettl3 | Rattus norvegicus methyltransferase-like 3 | -1.84 |
| Arhe | Rattus norvegicus ras homolog gene family, member E | -1.84 |
| LOC56769 | Rattus norvegicus nuclear protein E3-3 | -1.84 |
| Tep1 | Rattus norvegicus telomerase associated protein 1 | -1.84 |
| Sorl1 | Rattus norvegicus sortilin-related receptor, L | -1.84 |
| LOC316228 | Rattus norvegicus similar to p53-associated parkin-like cytoplasmic protein | -1.85 |
| Rnf7 | Rattus norvegicus ring finger protein 7 | -1.85 |
| LOC500662 | Rattus norvegicus similar to Glutathione S-transferase 8 | -1.85 |
| LOC360733 | Rattus norvegicus similar to 5830458K16Rik protein | -1.86 |
| Slc27a1 | Rattus norvegicus solute carrier family 27 | -1.86 |
| Luc7l2 | Rattus norvegicus LUC7-like 2 | -1.86 |
| Atad1 | Rattus norvegicus ATPase family, AAA domain containing 1 | -1.86 |
| LOC360602 | Rattus norvegicus similar to cisplatin resistance-associated overexpressed protein | -1.86 |
| Prpf39 | Rattus norvegicus PRP39 pre-mRNA processing factor 39 homolog | -1.87 |
| Ifitm3 | Rattus norvegicus interferon induced transmembrane protein 3 | -1.87 |
| LOC315903 | Rattus norvegicus similar to CG9346-PA | -1.89 |
| LOC499094 | Rattus norvegicus similar to zinc finger protein 61 | -1.89 |
| OSP94 | Rattus norvegicus osmotic stress protein 94 kDa | -1.89 |
| Mafg | Rattus norvegicus v-maf musculoaponeurotic fibrosarcoma oncogene family, protein G | -1.89 |
| LOC292477 | Rattus norvegicus similar to OTTHUMP00000040155 | -1.9 |
| Zcchc11 | Rattus norvegicus zinc finger, CCHC domain containing 11 | -1.9 |
| Rnf166 | Rattus norvegicus ring finger protein 166 | -1.9 |
| Qscn6 | Rattus norvegicus quiescin Q6 | -1.9 |
| LOC367903 | Rattus norvegicus similar to armadillo repeat protein ALEX2 | -1.9 |
| RT1-A2 | Rattus norvegicus RT1 class Ia, locus A2 | -1.9 |
| Apoa1bp | Rattus norvegicus apolipoprotein A-I binding protein | -1.9 |
| RGD1308384 | Rattus norvegicus similar to RIKEN cDNA 6330415M09 | -1.9 |
| LOC309953 | Rattus norvegicus similar to hypothetical protein MGC33214 | -1.91 |
| Plxnb2 | Rattus norvegicus plexin B2 | -1.91 |
| Sec5l1 | Rattus norvegicus Rsec5 protein | -1.93 |
| Podxl | Rattus norvegicus podocalyxin-like | -1.93 |
| LOC297514 | Rattus norvegicus similar to mKIAA1757 protein | -1.93 |
| Slc25a25 | Rattus norvegicus solute carrier family 25 | -1.94 |
| Cd14 | Rattus norvegicus CD14 antigen | -1.94 |
| MGC94736 | Rattus norvegicus similar to hypothetical protein MGC35097 | -1.95 |
| Msn | Rattus norvegicus moesin | -1.95 |
| Dpm1 | Rattus norvegicus dolichol-phosphate | -1.95 |
| LOC498378 | Rattus norvegicus similar to LRRGT00176 | -1.96 |
| Lama5 | Rattus norvegicus laminin, alpha 5 | -1.96 |
| LOC306229 | Rattus norvegicus similar to RIKEN cDNA A630054L15; hypothetical protein MGC38041 | -1.96 |
| Cryab | Rattus norvegicus crystallin, alpha B | -1.96 |
| LOC362938 | Rattus norvegicus similar to PDZ-domain protein scribble | -1.97 |
| Cirh1a | Rattus norvegicus cirrhosis, autosomal recessive 1A | -1.97 |
| Taf9 | Rattus norvegicus TAF9 RNA polymerase II, TATA box binding protein | -1.97 |
| LOC499554 | Rattus norvegicus similar to ORF2 consensus sequence encoding endonuclease and reverse transcriptase minus RNaseH | -1.98 |
| Dad1 | Rattus norvegicus defender against cell death 1 | -1.98 |
| Lkap | Rattus norvegicus limkain b1 | -1.99 |
| Nid2 | Rattus norvegicus nidogen 2 | -1.99 |
| LOC363478 | Rattus norvegicus similar to sorting nexin 12 | -2 |
| Ltap | Rattus norvegicus loop tail associated protein | -2 |
| LOC305502 | Rattus norvegicus similar to RIKEN cDNA 1110014L17 | -2 |
| Scara3 | Rattus norvegicus scavenger receptor class A, member 3 | -2 |
| Nrp1 | Rattus norvegicus neuropilin 1 | -2.01 |
| Cbr1 | Rattus norvegicus carbonyl reductase 1 | -2.01 |
| Cherp | Rattus norvegicus calcium homeostasis endoplasmic reticulum protein | -2.02 |
| Hnrph3 | Rattus norvegicus heterogeneous nuclear ribonucleoprotein H3 | -2.02 |
| Ndufb3 | Rattus norvegicus NADH dehydrogenase | -2.02 |
| LOC498644 | Rattus norvegicus similar to Ac1-163 | -2.02 |
| LOC500116 | Rattus norvegicus similar to RIKEN cDNA G430041M01 | -2.02 |
| Mbd1 | Rattus norvegicus methyl-CpG binding domain protein 1 | -2.03 |
| Nfix | Rattus norvegicus nuclear factor I/X | -2.03 |
| LOC498072 | Rattus norvegicus similar to High mobility group protein 2 | -2.03 |
| Ttyh1 | Rattus norvegicus tweety homolog 1 | -2.03 |
| Cldn11 | Rattus norvegicus claudin 11 | -2.04 |
| Dncl2b | Rattus norvegicus dynein, cytoplasmic, light chain 2B | -2.04 |
| LOC307302 | Rattus norvegicus similar to hypothetical protein FLJ36090 | -2.05 |
| Srpr | Rattus norvegicus signal recognition particle receptor | -2.05 |
| Zfp36l2 | Rattus norvegicus zinc finger protein 36, C3H type-like 2 | -2.06 |
| Copz1 | Rattus norvegicus coatomer protein complex, subunit zeta 1 | -2.06 |
| Lpd | Rattus norvegicus lipidosin | -2.06 |
| LOC314859 | Rattus norvegicus similar to transformed mouse 3T3 cell double minute 1 | -2.06 |
| LOC309816 | Rattus norvegicus similar to Laminin alpha-4 chain precursor | -2.07 |
| RGD1305132 | Rattus norvegicus similar to RIKEN cDNA A630065K24 | -2.07 |
| Ywhab | Rattus norvegicus tyrosine 3-monooxygenase/tryptophan 5-monooxygenase activation protein, beta polypeptide | -2.07 |
| LOC300783 | Rattus norvegicus similar to Butyrate-induced transcript 1 | -2.08 |
| Dscr1l1 | Rattus norvegicus Down syndrome critical region gene 1-like 1 | -2.08 |
| Tnk2 | Rattus norvegicus tyrosine kinase, non-receptor, 2 | -2.09 |
| LOC499589 | Rattus norvegicus similar to hypothetical protein MGC27085 | -2.09 |
| Lphn1 | Rattus norvegicus latrophilin 1 | -2.09 |
| LOC306991 | Rattus norvegicus similar to Vps41 protein | -2.09 |
| LOC360760 | Rattus norvegicus similar to OTTHUMP00000042400 | -2.09 |
| LOC498356 | Rattus norvegicus similar to MGC68837 protein | -2.1 |
| 1200013b22rik | Rattus norvegicus SNF1/AMP-activated protein kinase | -2.1 |
| LOC503190 | Rattus norvegicus similar to zinc finger protein 426 | -2.11 |
| Trappc4 | Rattus norvegicus trafficking protein particle complex 4 | -2.11 |
| Fank1 | Rattus norvegicus fibronectin type 3 and ankyrin repeat domains 1 | -2.11 |
| Hspa2 | Rattus norvegicus heat shock protein 2 | -2.11 |
| Rab6ip1 | Rattus norvegicus Rab6 interacting protein 1 | -2.11 |
| Znrd1 | Rattus norvegicus zinc ribbon domain containing, 1 | -2.13 |
| Eif4g3 | Rattus norvegicus eukaryotic translation initiation factor 4 gamma, 3 | -2.13 |
| Polr2i | Rattus norvegicus polymerase | -2.13 |
| Rtn1 | Rattus norvegicus reticulon 1 | -2.13 |
| Ppap2b | Rattus norvegicus ER transmembrane protein Dri 42 | -2.14 |
| Pex13 | Rattus norvegicus peroxisomal biogenesis factor 13 | -2.15 |
| Psmd4 | Rattus norvegicus proteasome | -2.15 |
| Sca2 | Rattus norvegicus spinocerebellar ataxia 2 | -2.15 |
| LOC500002 | Rattus norvegicus similar to A-kinase anchor protein 9 | -2.16 |
| Prelp | Rattus norvegicus proline arginine-rich end leucine-rich repeat protein | -2.16 |
| Lig1 | Rattus norvegicus ligase I, DNA, ATP-dependent | -2.17 |
| Adamts9 | Rattus norvegicus a disintegrin-like and metalloprotease | -2.18 |
| LOC501548 | Rattus norvegicus similar to LRRG00135 | -2.18 |
| LOC498245 | Rattus norvegicus similar to LRRGT00176 | -2.19 |
| Arl6ip2 | Rattus norvegicus ADP-ribosylation factor-like 6 interacting protein 2 | -2.19 |
| LOC313974 | Rattus norvegicus similar to Tribbles homolog 2 | -2.19 |
| Zfp462 | Rattus norvegicus zinc finger protein 462 | -2.2 |
| Trio | Rattus norvegicus triple functional domain | -2.22 |
| Lsm7 | Rattus norvegicus LSM7 homolog, U6 small nuclear RNA associated | -2.22 |
| LOC499564 | Rattus norvegicus similar to LRRGT00057 | -2.22 |
| Helz | Rattus norvegicus helicase with zinc finger domain | -2.23 |
| Slc25a29 | Rattus norvegicus solute carrier family 25 | -2.24 |
| Cyp4f6 | Rattus norvegicus cytochrome P450 4F6 | -2.24 |
| LOC362317 | Rattus norvegicus similar to krev interaction trapped-1A | -2.24 |
| LOC500939 | Rattus norvegicus LOC500939 | -2.24 |
| Atp2a2 | Rattus norvegicus ATPase, Ca++ transporting, cardiac muscle, slow twitch 2 | -2.25 |
| LOC499798 | Rattus norvegicus similar to ADP-ribosylation-like factor 6-interacting protein 6 | -2.26 |
| Aif1 | Rattus norvegicus allograft inflammatory factor 1 | -2.27 |
| Snrpa1 | Rattus norvegicus small nuclear ribonucleoprotein polypeptide A' | -2.28 |
| Prickle1 | Rattus norvegicus prickle-like 1 | -2.28 |
| LOC498674 | Rattus norvegicus LOC498674 | -2.29 |
| Zc3hdc7 | Rattus norvegicus zinc finger CCCH type domain containing 7 | -2.3 |
| Gpr37l1 | Rattus norvegicus G protein-coupled receptor 37-like 1 | -2.3 |
| Mgst2 | Rattus norvegicus microsomal glutathione S-transferase 2 | -2.32 |
| Ctbp2 | Rattus norvegicus C-terminal binding protein 2 | -2.32 |
| Ssx2ip | Rattus norvegicus synovial sarcoma, X breakpoint 2 interacting protein | -2.32 |
| LOC499560 | Rattus norvegicus similar to LRRG00135 | -2.33 |
| LOC293844 | Rattus norvegicus similar to UCH37-interacting protein 1 | -2.33 |
| LOC306734 | Rattus norvegicus similar to RIKEN cDNA 4932432N11 gene | -2.34 |
| Dnajb4 | Rattus norvegicus DnaJ | -2.35 |
| Zcwcc1 | Rattus norvegicus zinc finger, CW-type with coiled-coil domain 1 | -2.35 |
| Crim1 | Rattus norvegicus cysteine-rich motor neuron 1 | -2.36 |
| Lamb2 | Rattus norvegicus laminin, beta 2 | -2.36 |
| Gnao | Rattus norvegicus guanine nucleotide binding protein, alpha o | -2.38 |
| Phr1 | Rattus norvegicus pam, highwire, rpm 1 | -2.38 |
| Chrdl1 | Rattus norvegicus kohjirin | -2.39 |
| Slc1a5 | Rattus norvegicus sodium-dependent neutral amino acid transporter ASCT2 | -2.41 |
| LOC500865 | Rattus norvegicus similar to RIKEN cDNA 5730410E15 gene | -2.41 |
| Srp72 | Rattus norvegicus signal recognition particle 72 | -2.41 |
| LOC501637 | Rattus norvegicus similar to LRRG00135 | -2.41 |
| Pik3c3 | Rattus norvegicus phosphoinositide-3-kinase, class 3 | -2.43 |
| Sirt3 | Rattus norvegicus sirtuin 3 | -2.43 |
| MGC94018 | Rattus norvegicus glycosyltransferase AD-017 | -2.43 |
| Ccnl1 | Rattus norvegicus cyclin L1 | -2.43 |
| LOC497691 | Rattus norvegicus hypothetical gene supported by NM_134416 | -2.44 |
| LOC498931 | Rattus norvegicus similar to short coiled-coil protein | -2.44 |
| Tial1 | Rattus norvegicus Tial1 cytotoxic granule-associated RNA binding protein-like 1 | -2.45 |
| Snrp70 | Rattus norvegicus U1 small nuclear ribonucleoprotein polypeptide A | -2.45 |
| Prss35 | Rattus norvegicus protease, serine, 35 | -2.47 |
| LOC363492 | Rattus norvegicus similar to Ac1262 | -2.47 |
| LOC501562 | Rattus norvegicus similar to ORF2 consensus sequence encoding endonuclease and reverse transcriptase minus RNaseH | -2.5 |
| P2rxl1 | Rattus norvegicus purinergic receptor P2X-like 1, orphan receptor | -2.51 |
| Ech1 | Rattus norvegicus enoyl coenzyme A hydratase 1, peroxisomal | -2.51 |
| LOC363767 | Rattus norvegicus similar to ABI gene family, member 3 | -2.52 |
| LOC500867 | Rattus norvegicus similar to LRRG00116 | -2.52 |
| LOC317575 | Rattus norvegicus similar to Smarca1 protein | -2.53 |
| Camk2g | Rattus norvegicus calcium/calmodulin-dependent protein kinase II gamma | -2.53 |
| Per2 | Rattus norvegicus period homolog 2 | -2.53 |
| Inpp5b | Rattus norvegicus inositol polyphosphate-5-phosphatase B | -2.54 |
| Gstm1 | Rattus norvegicus glutathione S-transferase, mu 1 | -2.54 |
| Mpst | Rattus norvegicus mercaptopyruvate sulfurtransferase | -2.55 |
| Baz2b | Rattus norvegicus bromodomain adjacent to zinc finger domain, 2B | -2.58 |
| Adm | Rattus norvegicus adrenomedullin | -2.58 |
| Suv420h2 | Rattus norvegicus suppressor of variegation 4-20 homolog 2 | -2.59 |
| Txnrd1 | Rattus norvegicus thioredoxin reductase 1 | -2.6 |
| Aox1 | Rattus norvegicus aldehyde oxidase 1 | -2.6 |
| Cotl1 | Rattus norvegicus coactosin-like 1 | -2.61 |
| LOC498279 | Rattus norvegicus similar to NADH dehydrogenase | -2.62 |
| Ahr | Rattus norvegicus aryl hydrocarbon receptor | -2.64 |
| LOC500364 | Rattus norvegicus similar to Sspn protein | -2.65 |
| LOC304919 | Rattus norvegicus similar to RIKEN cDNA 5830468K18 | -2.66 |
| Cd38 | Rattus norvegicus CD38 antigen | -2.66 |
| Ccnl2 | Rattus norvegicus cyclin L2 | -2.66 |
| LOC500916 | Rattus norvegicus similar to LRRGT00176 | -2.67 |
| Pbxip1 | Rattus norvegicus pre-B-cell leukemia transcription factor interacting protein 1 | -2.68 |
| Agrn | Rattus norvegicus agrin | -2.69 |
| Btg2 | Rattus norvegicus B-cell translocation gene 2, anti-proliferative | -2.7 |
| LOC361885 | Rattus norvegicus similar to LRRGT00194 | -2.72 |
| LOC498623 | Rattus norvegicus similar to LRRGT00176 | -2.76 |
| Fbxl20 | Rattus norvegicus F-box and leucine-rich repeat protein 20 | -2.76 |
| Ppt | Rattus norvegicus palmitoyl-protein thioesterase | -2.76 |
| RT1-149 | Rattus norvegicus RT1 class I, T24, gene 4 | -2.77 |
| Dnah1 | Rattus norvegicus dynein, axonemal, heavy polypeptide 1 | -2.78 |
| Pdgfra | Rattus norvegicus platelet derived growth factor receptor, alpha polypeptide | -2.78 |
| Atp1a2 | Rattus norvegicus ATPase, Na+/K+ transporting, alpha 2 polypeptide | -2.79 |
| LOC499600 | Rattus norvegicus similar to 6030410K14Rik protein | -2.8 |
| Fbn2 | Rattus norvegicus fibrillin 2 | -2.81 |
| Trim39 | Rattus norvegicus tripartite motif protein 39 | -2.83 |
| Myh10 | Rattus norvegicus myosin heavy chain 10, non-muscle | -2.85 |
| Plagl1 | Rattus norvegicus pleiomorphic adenoma gene-like 1 | -2.86 |
| Sec24d | Rattus norvegicus SEC24 related gene family, member D | -2.87 |
| LOC501553 | Rattus norvegicus similar to LRRGT00078 | -2.87 |
| LOC498076 | Rattus norvegicus similar to RIKEN cDNA 2410116I05 | -2.89 |
| LOC365476 | Rattus norvegicus similar to chromosome 10 open reading frame 79 | -2.91 |
| LOC361912 | Rattus norvegicus similar to LRRG00116 | -2.93 |
| LOC497766 | Rattus norvegicus hypothetical gene supported by NM_171983 | -2.93 |
| LOC499531 | Rattus norvegicus similar to LRRGT00176 | -2.94 |
| Atp1b2 | Rattus norvegicus ATPase, Na+/K+ transporting, beta 2 polypeptide | -2.96 |
| LOC294789 | Rattus norvegicus similar to Hypothetical protein FLJ25422 | -3.02 |
| Hmgb2 | Rattus norvegicus high mobility group box 2 | -3.04 |
| Ugcg | Rattus norvegicus UDP-glucose ceramide glucosyltransferase | -3.05 |
| Mycl1 | Rattus norvegicus v-myc myelocytomatosis viral oncogene homolog 1, lung carcinoma derived | -3.05 |
| F3 | Rattus norvegicus coagulation factor III | -3.05 |
| LOC498048 | Rattus norvegicus similar to ORF4 | -3.06 |
| LOC361942 | Rattus norvegicus similar to ORF4 | -3.07 |
| Stc1 | Rattus norvegicus stanniocalcin 1 | -3.09 |
| LOC499196 | Rattus norvegicus LOC499196 | -3.12 |
| Esm1 | Rattus norvegicus endothelial cell-specific molecule 1 | -3.17 |
| Msln | Rattus norvegicus mesothelin | -3.19 |
| Lgi4 | Rattus norvegicus leucine-rich repeat LGI family, member 4 | -3.2 |
| isg12(b) | Rattus norvegicus putative ISG12 | -3.21 |
| Gstt2 | Rattus norvegicus glutathione S-transferase, theta 2 | -3.21 |
| Dvl1 | Rattus norvegicus dishevelled, dsh homolog 1 | -3.22 |
| LOC501087 | Rattus norvegicus similar to LRRGT00057 | -3.25 |
| Tcfap2b | Rattus norvegicus transcription factor AP-2 beta | -3.31 |
| Gstp1 | Rattus norvegicus glutathione-S-transferase, pi 1 | -3.35 |
| LOC362587 | Rattus norvegicus similar to microfilament and actin filament cross-linker protein isoform a | -3.37 |
| LOC500960 | Rattus norvegicus similar to Da1-12 | -3.39 |
| Sfrs5 | Rattus norvegicus splicing factor, arginine/serine-rich 5 | -3.39 |
| Zfp36 | Rattus norvegicus zinc finger protein 36 | -3.44 |
| LOC362315 | Rattus norvegicus similar to Retrovirus-related POL polyprotein | -3.55 |
| LOC500398 | Rattus norvegicus similar to LRRGT00082 | -3.7 |
| Tnmd | Rattus norvegicus tenomodulin | -3.78 |
| Enpp2 | Rattus norvegicus ectonucleotide pyrophosphatase/phosphodiesterase 2 | -3.83 |
| Sv2b | Rattus norvegicus synaptic vesicle glycoprotein 2b | -3.84 |
| Cyp26b1 | Rattus norvegicus cytochrome P450, family 26, subfamily b, polypeptide 1 | -3.89 |
| Sostdc1 | Rattus norvegicus uterine sensitization-associated gene 1 protein | -3.9 |
| LOC316085 | Rattus norvegicus similar to 106 kDa O-GlcNAc transferase-interacting protein | -4.06 |
| Per1 | Rattus norvegicus period homolog 1 | -4.09 |
| Hmgn3 | Rattus norvegicus high mobility group nucleosomal binding domain 3 | -4.15 |
| LOC362543 | Rattus norvegicus similar to LRRG00116 | -4.37 |
| Srrm2 | Rattus norvegicus serine/arginine repetitive matrix 2 | -4.43 |
| Ptgs2 | Rattus norvegicus prostaglandin-endoperoxide synthase 2 | -6.44 |
| Txnip | Rattus norvegicus upregulated by 1,25-dihydroxyvitamin D-3 | -11.08 |
