# Supplementary material for: Multiple Oxygen Tension Environments Reveal Diverse Patterns of Transcriptional Regulation in Primary Astrocytes
Source: PLoS One. 2011 Jun 27;6(6):e21638. doi: 10.1371/journal.pone.0021638 (PMC3124552; doi:10.1371/journal.pone.0021638)
Supplement: Table S4 — Venn diagram analysis output for significant gene regulation between 1, 4, 9% O2 tensions versus 20% O2. Official gene symbols are employed to demonstrate the significantly regulated genes populating the Venn diagram intersections, A–G, depicted in Figure 2. (DOC) [file pone.0021638.s010.doc]

**Table S4. Venn diagram analysis output for significant gene regulation between 1, 4, 9% O_2_ tensions versus 20% O_2_.** Official gene symbols are employed to demonstrate the significantly regulated genes populating the Venn diagram intersections, A-G, depicted in Figure 2.

| **A** | **B** | **C** | **D** | **E** | **F** | **G** |
| --- | --- | --- | --- | --- | --- | --- |
| Hk2 | Lcn2 | Thrsp | Pdk1 | Hmox1 | Ca3 | Acta1 |
| Pim1 | LOC500721 | Abcb9 | P4ha1 | LOC501140 | Gdf10 | Ddit3 |
| Vegfa | Grem1 | LOC497936 | LOC501644 | Bnip3 | Rbm3 | LOC500271 |
| Dusp8 | Gstp2 | LOC311592 | Xpo1 | Gadd45b | Snai1 | Atf4 |
| Bhlhb2 | S100a4 | Wisp2 | Mt1a | LOC364048 | Ass | Pgk1 |
| LOC302402 | LOC314434 | Col14a1 | LOC500983 | LOC292656 | Serpine1 | LOC307731 |
| Ptp4a1 | Ccl2 | LOC501605 | LOC298169 | Gdf15 | Nqo1 | Aldoa |
| G0s2 | LOC500817 | Crabp1 | Myd116 | Ppp1r3c | Mfap4 | Ldha |
| Ak3l1 | Thy1 | LOC301438 | Klf5 | LOC500959 | Serpine2 | LOC365954 |
| Nr4a3 | LOC292273 | LOC367339 | LOC294748 | Pfkl | Hes1 | LOC498019 |
| Timp3 | LOC292539 | Scd1 | Fth1 | Uap1l1 | Pcolce | LOC295423 |
| Nr1d1 | Gm1012 | LOC498406 | Hnrpdl | Tpi1 | LOC294781 | LOC500965 |
| Egln1 | S100a6 | LOC500666 | LOC361797 | LOC498731 | Mk1 | LOC500104 |
| Pygl | Tgfb3 | LOC498068 | Ctgf | LOC497927 | LOC361912 | LOC498099 |
| Insig1 | Cct8 | Ppap2c | Vim | Flcn | LOC310512 | LOC299622 |
| LOC291715 | LOC367822 | Lancl2 | Tagln | Hspb1 | Fstl3 | LOC290634 |
| Gadd45g | Thbs2 | Foxg1 | LOC497684 | Cryab | Pdlim7 | Adm |
| Eno2 | Fbln2 | Casp8ap2 | LOC500856 | Luc7l | Atf5 | LOC295452 |
| Slc16a3 | LOC364343 | Mapre3 | LOC499906 | LOC306115 | Tm4sf1 | LOC498618 |
| Hig1 | Tle2 | Igfbp3 | LOC361061 | Gapd | LOC310360 | LOC499433 |
| LOC497803 | Slc38a4 | Fbl | Actg | Eno1 | LOC366999 | Cmkor1 |
| Rragd | LOC293632 | Scand1 | Icam1 | LOC502770 | Prpf39 | LOC364848 |
| Atf3 | Ppp1r14a | Hist1h2bp | Actb | Cyr61 | LOC300278 | LOC498881 |
| LOC499201 | LOC498744 | LOC299127 | Ednrb | Wsb1 | Gstm2 | Ptgis |
| LOC500804 | LOC289401 | Ilvbl | Slc3a2 | LOC497816 | Bgn | Mdm2 |
| Ftl1 | LOC302363 | Gadd45gip1 | Eef1a1 | LOC500506 | Tnn | LOC502063 |
| Rere | Penk-rs | Apex1 | LOC500859 | Gpi | LOC299907 | LOC294700 |
| LOC366205 | Mrps18c | Mfge8 | LOC500885 | Ddit4 | LOC366485 | Lamr1 |
| Pbef1 | Rgs14 | LOC290706 | Rps11 | Tm4sf9 | LOC500645 | Ubb |
| LOC499244 | LOC499660 | Pafah1b2 | LOC366193 | Lama5 | LOC500867 | Prelp |
| Dyrk2 | Birc6 | LOC363865 | LOC314556 | Dpp7 | Fdps | Gnpat |
| Cebpb | Gmps | LOC302500 | Rplp1 | Hspca | Lum | Tec |
| Mif | Ccl7 | LOC501536 | Rps27 | LOC499423 | Arpp19 | Ndr4 |
| Mak3 | LOC501604 | Rae1 | Rps18 | LOC310585 | Nfkbia | Sec24d |
| LOC498038 | Rps17 | LOC308503 | LOC299041 | Vars2 | LOC302388 | Mtch2 |
| Maff | Sqrdl | Ywhag | LOC498143 | Sv2b | LOC302497 | LOC313974 |
| LOC499794 | Sema3f | Gng11 | LOC289715 | Bsg | LOC298495 | Csad |
| LOC499178 | Sirt6 | Fbxo33 | LOC500559 | LOC500242 | Arpc1b | Nedd9 |
| Hspbap1 | Cnn1 | Tax1bp1 | Rpl41 | Ccnl1 | Colm | LOC499196 |
| Nfil3 | LOC502854 | LOC289930 | LOC364108 | Cd63 | LOC498048 | Nrp1 |
| Car9 | LOC498212 | Exosc5 | Rpl19 | Bri3 | Rhoa | Nr2f1 |
| Slc5a3 | LOC500155 | Smfn | LOC499457 | LOC363531 | Stmn2 | Plekhb2 |
| Dctn4 | Mapre1 | LOC315329 | LOC502302 | LOC296582 | Snrp70 | LOC499856 |
| Mgea5 | Jag1 | Dab2ip | Rps3 | Rps2 | LOC363492 | Serpinb1a |
| Slc38a1 | Runx1 | 0610031j06rik | LOC304035 | Rps10 | LOC501203 | Ppp1r14b |
| osr1 | MGC72560 | Sod3 | Rpl35 | Rpl24 | Col8a1 | Gpr37l1 |
| Umpk | LOC290912 | Ttk | Rps27a | Rpl21 | Hmgn2 | Olig1 |
| Cxcl10 | Tfb2m | Cct5 | LOC499133 | LOC499845 | LOC366656 | Tnfrsf11b |
| Gnl3 | LOC498062 | Lcn7 | LOC293860 | P4hb | Lxn | Gstp1 |
| LOC365699 | Ecm1 | Tnnt2 | Rps14 | Hspa8 | Akr1a1 | Nid2 |
| Cpeb4 | LOC503172 | Ebp | Rpl10 | Rpl32 | Rpl10a | Tnmd |
| C3orf6h | Fgfr3 | Commd8 | Rpl29 | Eif3s5 | RGD1307627 | Cyp26b1 |
| Pfkp | Fbn1 | Zfp207 | Rpl26 | Calr | Ifitm3 | Ttyh1 |
| LOC303554 | Vmp1 | Asam | LOC498998 | Rnf7 | Gdi2 | MGC94018 |
| LOC310395 | Igsf4c | Echdc1 | Fau | RGD1310571 | Slc25a29 | LOC294789 |
| Rora | Nat5 | LOC498078 | LOC315642 | RGD1311463 | Lig1 | Dspg3 |
| LOC290704 | LOC306805 | Znf297 | Rpl18 | LOC296469 | MGC72974 | Cldn11 |
| MGC94288 | Dnm1 | Txndc5 | Fn1 | Zcwcc1 | Pik3c3 | LOC500987 |
| LOC288065 | Sui1-rs1 | LOC303471 | LOC298785 | Nup107 | Aif1 | Atp1a2 |
| LOC499171 | RGD1310991 | LOC498375 | LOC307135 | Mgst2 | LOC499094 | Enpp2 |
| Dre1 | Nbl1 | Slc37a4 | Rps15 | Zfp462 | P2rxl1 |  |
| Dmd | Dia1 | Ubtd1 | LOC362181 | LOC316085 | Podxl |  |
| Aatf | MGC94283 | Smndc1 | LOC302528 | LOC362317 | LOC304919 |  |
| LOC310648 | Exosc8 | Ptn | Rps8 | Stc1 | Lgi4 |  |
| MGC94937 | Rpl35a | Plxdc2 | LOC498555 | LOC499882 | LOC500865 |  |
| LOC365753 | LOC305887 | MGC94782 | Rpl37a | Ahr | Wdr34 |  |
| Ccng2 | Phf3 | Psmd5 | Rps25 | Lphn1 | Zfp36l2 |  |
| Slc40a1 | LOC287132 | Slc25a1 | Rps20 | Cerk | LOC499798 |  |
| Trit1 | Rps12 | Pam | LOC309408 | Sec5l1 | Cox17 |  |
| Bhlhb3 | LOC363861 | LOC503418 | LOC499305 | LOC293844 | LOC315804 |  |
| Mdm4 | LOC499592 | Tram1 | Rpl8 | Lactb | Snrpa1 |  |
| LOC300870 | LOC364381 | LOC366689 | Ppia | Polr2b | Cotl1 |  |
| Ndfip2 | LOC296402 | Carhsp1 | Cfl1 | Eln | LOC498931 |  |
| Cggbp1 | Arrdc1 | Rabep1 | LOC299935 | RGD1307010 | Psmd4 |  |
| Tbc1d15 | LOC497693 | Rpp14 | LOC500929 | Fbn2 | Polr2i |  |
| LOC499690 | Rab9 | Faf1 | Lyplal1 | Nde1 | Lcat |  |
| Ugp2 | Sdf4 | MGC109491 | LOC294734 | Snx14 | Lpl |  |
| Copeb | LOC499501 | RGD1359127 | LOC498564 | Srpr | Slc27a1 |  |
| Prss15 | Tcirg1 | Apeh | LOC500441 | LOC498095 | LOC305035 |  |
| Pamci | Ninj1 | Tomm40 | RGD1308373 | LOC499125 | LOC500671 |  |
| Ddr1 | Olfml3 | Ndn | LOC290372 | Agrn | Cte1 |  |
| Tiparp | LOC307416 | Sfrs2 | LOC293888 | Gstt2 | Pex13 |  |
| LOC363377 | Ppic | LOC498363 | Adamts1 | Srrm2 | Sorl1 |  |
| Arih1 | LOC311120 | Bambi | RGD1307008 | Hmgn3 | Trim39 |  |
| Pim3 | Akap8 | LOC361571 | Tfpi | Ltap | Maob |  |
| LOC316842 | Epdr2 | Tmepai | LOC498751 | Hspa2 | Myh14 |  |
| Actn1 | Agtr1a | Qdpr | Gprasp1 | Mtvr2 | LOC306991 |  |
| Gsk3b | LOC298841 | RGD1304567 | Ier2 | Lsm7 | LOC292477 |  |
| Wdfy1 | LOC364258 | Ctsd | Cirbp | Sdfr1 | Phr1 |  |
| Mrpl45 | Tnpo3 | Plod1 | LOC302378 | LOC360602 | LOC497766 |  |
| Hif1a | LOC363418 | Ddx46 | Pros1 | Prss35 | F3 |  |
| RGD1311518 | LOC300802 | Hmmr | LOC309081 | Atp2a2 | LOC499589 |  |
| Dtr | LOC314759 | LOC316539 | Adprt | LOC363443 | Tcfap2b |  |
| Pawr | Rps9 | LOC497831 | Tsn | Lrrc5 | Edg2 |  |
| Nol8 | Plcg1 | Enpp1 | LOC498398 | LOC497841 | LOC317575 |  |
| LOC309161 | Ppp3ca | Dnajb11 | LOC296758 | Gusb | Abhd3 |  |
| LOC498105 | LOC368016 | LOC292780 | Boc | Sphk1 | Ltbp4 |  |
| LOC367857 | Sfrp2 | Tcn2 | Ucp2 | Sfrs5 | Rtn1 |  |
| Arbp | Myl9 | LOC366411 | Efemp1 | LOC361178 | Dnah1 |  |
| Plekhm2 | Serpinf1 | Crebl1 | Cspg5 | LOC498674 | Sparcl1 |  |
| Mcl1 | Eef1b2 | LOC300760 | Cfh | LOC362264 | Ppt |  |
| LOC498661 | Ssr4 | LOC362513 | LOC302980 | Lamb1-1 | LOC498356 |  |
| Eif4ebp1 | LOC499852 | LOC299823 | Myadm | Ttc13 | Giot1 |  |
| Cd83 | Tead3 | Rara | Lypla1 | Ywhab | Tf |  |
| Ndel1 | LOC498828 | LOC501007 | Bmp7 | Mig12 | LOC500939 |  |
| Ppp2r2a | Rpl13a | LOC500795 | Zic2 | Col6a3 | RGD1306222 |  |
| LOC366693 | Lgals1 | Fosl1 | Calm1 | Hsd3b7 | Fank1 |  |
| Vgll4 | Rps7 | Phax | MGC94969 | Scara3 | Dncl2b |  |
| Hspcal3 | LOC366887 | Bcap29 | Abcd3 | Ppap2b | Sostdc1 |  |
| RGD1307599 | Ndufa5 | Mphosph10 | Calm3 | Cx3cl1 | Esm1 |  |
| LOC311772 | S100a11 | Taf10 | AY228474 | Chdh | A2m |  |
| Nfe2l2 | Rps13 | Sfrs10 | LOC499593 | Loxl1 | OSP94 |  |
| LOC361613 | Gclc | Eif4a1 | Gap43 | Pdgfra | Mycl1 |  |
| LOC301068 | Tnfrsf12a | Chchd3 | Hnrpa3 | LOC306229 | LOC360747 |  |
| LOC500226 | LOC500451 | Fads1 | Dcn | Zfp36 | LOC365476 |  |
| Cited2 | Eif4el3 | LOC365416 | AF146738 | Btg2 | Ptgs2 |  |
| LOC360807 | Tceb2 | MGC94190 | Rab11a | MGC95138 |  |  |
| Rnpc2 | Btf3 | Aldh1a1 | Cspg2 | Cd164l1 |  |  |
| LOC293103 | LOC498293 | Tp53rk | LOC294942 | Dhcr7 |  |  |
| Ewsr1 | Sat | Dirc2 | LOC360546 | Hmgcs1 |  |  |
| Id2 | Commd3 | Anp32a | Sesn1 | Txnip |  |  |
| St13 | LOC364828 | Tex264 | Aqp1 |  |  |  |
| Rab24 | MGC72942 | Ranbp1 | Dnajc10 |  |  |  |
| Rabggtb | LOC501280 | Pfdn1 | Igsf11 |  |  |  |
| Rragc | LOC499317 | Psmc3 | Col1a1 |  |  |  |
| Tpm1 | Timp1 | LOC292588 | Alcam |  |  |  |
| Ubqln1 | Ssr2 | Pgls | Gjb2 |  |  |  |
| Cops4 | LOC363441 | Nme1 | Ptgds |  |  |  |
| LOC500914 | LOC500923 | LOC313722 |  |  |  |  |
| Lgals3 | Ddah1 | MGC94233 |  |  |  |  |
| Tln | LOC288146 | C1qbp |  |  |  |  |
| Clta | Prdx5 | LOC302671 |  |  |  |  |
| Cstb | LOC363918 | Rpl13 |  |  |  |  |
| LOC312363 | LOC498360 | Gabarap |  |  |  |  |
| Naca | LOC298126 | Mdk |  |  |  |  |
| Vapa | Psmb4 | Map2k3 |  |  |  |  |
| LOC295439 | Slc38a2 | C1s |  |  |  |  |
| LOC501206 | LOC364139 | Ruvbl1 |  |  |  |  |
| Pabpc1 | Tpm4 | LOC361578 |  |  |  |  |
| Hnrpl | Rpl39 | Prc1 |  |  |  |  |
| Prss23 | F2r | LOC366258 |  |  |  |  |
| Jund | LOC367102 | Dnclc1 |  |  |  |  |
| Rpl36 | Actr2 | Ddx21b |  |  |  |  |
| LOC303815 | LOC498523 | Cct6a |  |  |  |  |
| LOC296870 | Rpl27 | Akr1b4 |  |  |  |  |
| LOC291308 | Rps23 | LOC304638 |  |  |  |  |
| LOC361026 | Eef1d | Slc1a3 |  |  |  |  |
| LOC500660 | LOC301299 | LOC497882 |  |  |  |  |
| LOC293642 | Ctsl | Emp3 |  |  |  |  |
| LOC295472 | Cst3 | LOC499512 |  |  |  |  |
| Hdlbp | LOC497813 | Mdh1 |  |  |  |  |
| LOC310365 | Igf2 | Apoe |  |  |  |  |
| Rpl14 | Baalc | Pbp |  |  |  |  |
| Npm1 | LOC299050 | Cox6a1 |  |  |  |  |
| Rpl27a | Dnase2 | RGD1307700 |  |  |  |  |
| LOC361115 | Eml2 | Mcm3ap |  |  |  |  |
| LOC500669 | Donson | LOC287452 |  |  |  |  |
| LOC499523 | Lsamp | MGC94053 |  |  |  |  |
| LOC500547 | Pc | Enh |  |  |  |  |
| Atp6v1g1 | Gabarapl2 | Sult1a1 |  |  |  |  |
| LOC501980 | Nubp1 | LOC500042 |  |  |  |  |
| Rpl3 | LOC311796 | LOC497834 |  |  |  |  |
| LOC499803 | LOC306137 | LOC500504 |  |  |  |  |
| Rpl4 | Tyki | LOC299199 |  |  |  |  |
| Rps5 | Mtpn | Psmd13 |  |  |  |  |
| LOC503284 | Smarca5 | Smad1 |  |  |  |  |
| Rps3a | Phyhd1 | RGD1310143 |  |  |  |  |
| Junb | RGD735029 | LOC288515 |  |  |  |  |
| Ywhae | LOC293566 | RGD1306020 |  |  |  |  |
| LOC362290 | Kifap3 | LOC498276 |  |  |  |  |
| Tpt1 | Kif20a | Actn4 |  |  |  |  |
| Lamp1 | LOC315645 | LOC315216 |  |  |  |  |
| LOC302445 | LOC296126 | Sec23ip |  |  |  |  |
| LOC503110 | LOC368066 | LOC500974 |  |  |  |  |
| Rps4x | LOC362580 | LOC364468 |  |  |  |  |
| Rpl7a | Prtfdc1 | Ddx23 |  |  |  |  |
| Slc25a4 | Fkbp5 | LOC362840 |  |  |  |  |
| Ckb | Casp7 | Abca2 |  |  |  |  |
| LOC367398 | LOC315973 | LOC305452 |  |  |  |  |
| LOC502629 | LOC362414 | Cd44 |  |  |  |  |
| Rpl9 | Cldn9 | Klf4 |  |  |  |  |
| Pttg1ip | Nt5c3 | Dpp3 |  |  |  |  |
| Rps15a | RGD1311049 | LOC361117 |  |  |  |  |
| Rpl17 | Ppid | Wbp11 |  |  |  |  |
| Uba52 | Bf | Ctbs |  |  |  |  |
| Atp5b | LOC360478 | Pla2g6 |  |  |  |  |
| Rps6 | LOC246187 | LOC289437 |  |  |  |  |
| Eef2 | Setdb1 | Ddx47 |  |  |  |  |
| Rpl6 | Ifi44 | Nup54 |  |  |  |  |
| Gnai2 | Kpna1 | Nisch |  |  |  |  |
| Peci | Banp | Frg1 |  |  |  |  |
| MGC94282 | Farslb | LOC306007 |  |  |  |  |
| Hint3 | MGC72987 | RT1-A1 |  |  |  |  |
| Ythdf2 | LOC501157 | Rda279 |  |  |  |  |
| Sema6a | Fchsd2 | LOC499323 |  |  |  |  |
| LOC360894 | RGD1303232 | Loc65027 |  |  |  |  |
| LOC303567 | LOC315158 | MGC105797 |  |  |  |  |
| Psme2 | Rpia | Lrp16 |  |  |  |  |
| Zfp297b | Gbp2 | LOC361774 |  |  |  |  |
| LOC502017 | Rkhd2 | Slfn3 |  |  |  |  |
| Cdkn3 | Dhx32 | Flnc |  |  |  |  |
| Slc39a13 | Exosc3 | Acyp1 |  |  |  |  |
| Ipo13 | Ppp4r2 | Gstm3 |  |  |  |  |
| Lama2 | RGD1308463 | Fam38a |  |  |  |  |
| Mrpl49 | RGD1309676 | Txndc1 |  |  |  |  |
| LOC288659 | Myc | Lancl1 |  |  |  |  |
| Pank2 | LOC310946 | LOC291750 |  |  |  |  |
| LOC501633 | Rbm10 | LOC498228 |  |  |  |  |
| LOC292751 | Psip1 | RGD1309382 |  |  |  |  |
| Chst2 | LOC499625 | Cry1 |  |  |  |  |
| Mak10 | Nrep | B3gnt1 |  |  |  |  |
| Krt1-19 | Cds1 | Waspip |  |  |  |  |
| Ctsh | Tufm | Cpd |  |  |  |  |
| LOC362578 | Vamp4 | LOC500829 |  |  |  |  |
| Scp2 | Ascl1 | LOC363474 |  |  |  |  |
| Ubl3 | LOC500536 | Usp7 |  |  |  |  |
| RGD1310022 | Sdccag3 | LOC502614 |  |  |  |  |
| Eppb9 | Adk | Itga7 |  |  |  |  |
| RGD1311243 | Smoc2 | Vps16 |  |  |  |  |
| Mrps17 | LOC304289 | Mcm2 |  |  |  |  |
| Slc20a2 | Cpe | LOC360733 |  |  |  |  |
| LOC499335 | Krt2-8 | Pcnxl3 |  |  |  |  |
| LOC499496 | Znf386 | LOC309307 |  |  |  |  |
| mrpl11 | Bphl | LOC316228 |  |  |  |  |
| Cul3 | LOC171553 | Ddost |  |  |  |  |
| Fjx1 | LOC292486 | LOC315903 |  |  |  |  |
| Mapk7 | RGD1310686 | Cxcl1 |  |  |  |  |
| RGD1307935 | RGD1311364 | Usp2 |  |  |  |  |
| LOC302553 | Anxa5 | Vcam1 |  |  |  |  |
| Pou3f3 | LOC360941 | Dhx30 |  |  |  |  |
| LOC502782 | Sdccag8 | Ndrg2 |  |  |  |  |
| Fgfrl1 | Gclm | Tex27 |  |  |  |  |
| Hat1 | LOC361519 | Irf3 |  |  |  |  |
| Exosc4 | Paqr4 | LOC498388 |  |  |  |  |
| LOC315697 | Snx25 | Sugt1 |  |  |  |  |
| Cd2bp2 | LOC299209 | LOC317612 |  |  |  |  |
| RGD1308470 | LOC289900 | Zcchc11 |  |  |  |  |
| Ndufa8 | LOC303238 | LOC500662 |  |  |  |  |
| Arf2 | Nfia | MGC105508 |  |  |  |  |
| Tlk1 | Jun | Snrpd2 |  |  |  |  |
| Rasl11b | LOC287274 | Rnf166 |  |  |  |  |
| Scamp2 | LOC361309 | Ehmt1 |  |  |  |  |
| Trp53rk | Pnrc1 | LOC309953 |  |  |  |  |
| Mkl1 | LOC361988 | Catns |  |  |  |  |
| D123 | LOC294917 | MGC94954 |  |  |  |  |
| LOC290833 | Capza2 | Qscn6 |  |  |  |  |
| Pycrl | Slc15a4 | Mettl3 |  |  |  |  |
| Cxcr4 | LOC501341 | Ifi27l |  |  |  |  |
| Rbm4 | Nphp1 | LOC498378 |  |  |  |  |
| Slc29a3 | Rarres1 | LOC362938 |  |  |  |  |
| Ccna2 | LOC291354 | LOC367903 |  |  |  |  |
| LOC361502 | Ptdss2 | Ndufb7 |  |  |  |  |
| LOC360738 | Hexa | Luc7l2 |  |  |  |  |
| Usp47 | Sirt2 | Mark3 |  |  |  |  |
| Ube4a | Hes6 | Arhe |  |  |  |  |
| Traf4af1 | LOC313445 | Col18a1 |  |  |  |  |
| B4galt4 | LOC365214 | Akap12 |  |  |  |  |
| Fh1 | Sema3b | Slc25a25 |  |  |  |  |
| Rab3d | MGC93902 | Atad1 |  |  |  |  |
| Amacr | Pcsk1n | LOC56769 |  |  |  |  |
| Ube4b | Galm | Tep1 |  |  |  |  |
| Rom1 | Flot1 | Itgb1bp1 |  |  |  |  |
| Cdc91l1 | LOC304332 | Cd14 |  |  |  |  |
| Abr | Lr8 | LOC362490 |  |  |  |  |
| LOC362592 | Lrp11 | LOC499615 |  |  |  |  |
| Exosc7 | Kpnb3 | MGC94736 |  |  |  |  |
| LOC501521 | Parva | LOC363478 |  |  |  |  |
| Plxnd1 | Omd | Npr2 |  |  |  |  |
| Bcat2 | Rpl15 | Msn |  |  |  |  |
| Col16a1 | Mpp6 | LOC499554 |  |  |  |  |
| LOC501550 | Aard | Lkap |  |  |  |  |
| LOC299341 | LOC499513 | Zfp330 |  |  |  |  |
| Slc33a1 | Gpm6a | Cherp |  |  |  |  |
| Stk16 | Cdo1 | Tctex1 |  |  |  |  |
| Sfxn1 | LOC308820 | Cln2 |  |  |  |  |
| RGD1310284 | Stk39 | Cirh1a |  |  |  |  |
| Lrrk1 | Atp5c1 | LOC303790 |  |  |  |  |
| Ythdf1 | Anxa3 | Decr1 |  |  |  |  |
| Fndc3 | Atp6ap2 | LOC503190 |  |  |  |  |
| LOC308708 | Ggcx | Mbd1 |  |  |  |  |
| Prkar2b | Urod | RT1-A2 |  |  |  |  |
| Wee1 | LOC301521 | Magi3 |  |  |  |  |
| Rb1cc1 | Ivns1abp | Apoa1bp |  |  |  |  |
| LOC296733 | LOC301563 | Tnk2 |  |  |  |  |
| RGD1307203 | Loxl2 | LOC309816 |  |  |  |  |
| Cnot7 | RGD1311155 | Hnrph3 |  |  |  |  |
| LOC498035 | LOC503409 | Copz1 |  |  |  |  |
| LOC497712 | Scg3 | LOC300783 |  |  |  |  |
| Gtl3 | Cfdp1 | Trappc4 |  |  |  |  |
| Zmpste24 | LOC302863 | RGD1308384 |  |  |  |  |
| MGC94479 | Plekhb1 | LOC297514 |  |  |  |  |
| Lgals3bp | LOC360819 | Znrd1 |  |  |  |  |
| LOC361980 | Rhpn1 | RGD1305132 |  |  |  |  |
| Svil | LOC314949 | Plxnb2 |  |  |  |  |
| Map2k4 | LOC313842 | Nfix |  |  |  |  |
| RGD1307632 | LOC317444 | Mafg |  |  |  |  |
| Mesdc2 | Asrgl1 | Cbr1 |  |  |  |  |
| LOC500462 | Adss2 | LOC307302 |  |  |  |  |
| MGC94463 | Sil1 | Tmp21 |  |  |  |  |
| Fzd1 | MGC105961 | LOC498245 |  |  |  |  |
| Chp | Csda | Adamts9 |  |  |  |  |
| Msh6 | RGD1305061 | Dscr1l1 |  |  |  |  |
| Ehd4 | LOC366872 | Lpd |  |  |  |  |
| Ext2 | Mlc1 | Sod1 |  |  |  |  |
| Emilin1 | Glrx2 | LOC500002 |  |  |  |  |
| LOC498107 | Aqp4 | LOC498072 |  |  |  |  |
| Msx1 | LOC290851 | Helz |  |  |  |  |
| LOC298906 | Spag8 | Arl6ip2 |  |  |  |  |
| Tmpo | Igbp1 | LOC314859 |  |  |  |  |
| LOC500015 | Hnrpm | Taf9 |  |  |  |  |
| Klf7 | Gmnn | Ndufb3 |  |  |  |  |
| Laptm4b | LOC317396 | Dpm1 |  |  |  |  |
| Map3k4 | Yme1l1 | LOC305502 |  |  |  |  |
| LOC363309 | Fbln1 | Dad1 |  |  |  |  |
| Mocs2 | Capzb | Eif4g3 |  |  |  |  |
| Kdelr3 | LOC498278 | Trio |  |  |  |  |
| Blcap | Dnai2 | LOC498644 |  |  |  |  |
| LOC299828 | LOC499328 | LOC500116 |  |  |  |  |
| Ywhah | Rnpep | LOC360760 |  |  |  |  |
| Glrx1 | RGD1305524 | Cyp4f6 |  |  |  |  |
| LOC361605 | Fxna | LOC499564 |  |  |  |  |
| Lsm8 | LOC365389 | 1200013b22rik |  |  |  |  |
| G3bp | Pdgfa | Rab6ip1 |  |  |  |  |
| Pdk2 | C4-2 | Dnajb4 |  |  |  |  |
| Dnajc9 | LOC497844 | Crim1 |  |  |  |  |
| MGC94555 | Epb4.1l3 | LOC499560 |  |  |  |  |
| LOC362776 | Camk2n1 | Prickle1 |  |  |  |  |
| Cenpb | Igsf1 | Zc3hdc7 |  |  |  |  |
| Statip1 | Erp29 | Slc1a5 |  |  |  |  |
| Zfp347 | Gpr51 | Sirt3 |  |  |  |  |
| Mlycd | Pcm1 | Chrdl1 |  |  |  |  |
| Casp2 | LOC499268 | Ctbp2 |  |  |  |  |
| RGD1311316 | LOC499839 | LOC497691 |  |  |  |  |
| Gp1bb | Sez6 | LOC306734 |  |  |  |  |
| LOC361712 | RGD1308075 | Sca2 |  |  |  |  |
| LOC501282 | LOC362809 | Ssx2ip |  |  |  |  |
| Rgs3 | Strbp | LOC501548 |  |  |  |  |
| Sfpq | Gpc3 | Gnao |  |  |  |  |
| LOC302313 | Zmynd10 | LOC501562 |  |  |  |  |
| Ssbp1 | LOC296608 | Txnrd1 |  |  |  |  |
| Dr1 | LOC313436 | Srp72 |  |  |  |  |
| Acp2 | Mlf1 | Mpst |  |  |  |  |
| Thtpa | Tekt1 | Tial1 |  |  |  |  |
| Snx4 | Egr2 | Camk2g |  |  |  |  |
| LOC360632 | LOC500416 | Per2 |  |  |  |  |
| LOC305633 | LOC287346 | LOC498279 |  |  |  |  |
| Pphln1 | Khdrbs3 | LOC500364 |  |  |  |  |
| Prr3 | LOC291847 | Inpp5b |  |  |  |  |
| Fbxo11 | Itgb4 | Baz2b |  |  |  |  |
| MGC93733 | Dnaja4 | LOC363767 |  |  |  |  |
| Rrm2 | LOC300517 | Cd38 |  |  |  |  |
| Psmb10 | LOC503278 | LOC500916 |  |  |  |  |
| Cdk2ap1 | Arhgdig | RT1-149 |  |  |  |  |
| Arpc1a | Lrpb7 | LOC501637 |  |  |  |  |
| RGD1305356 | Aurkb | LOC498623 |  |  |  |  |
| Thra | LOC293156 | Lamb2 |  |  |  |  |
| Pofut2 | MGC105647 | LOC361885 |  |  |  |  |
| LOC303630 | Fez1 | Fbxl20 |  |  |  |  |
| LOC290925 | LOC498982 | Aox1 |  |  |  |  |
| LOC292995 | Calb1 | Suv420h2 |  |  |  |  |
| LOC310760 | LOC310926 | LOC499600 |  |  |  |  |
| Ormdl2 |  | Plagl1 |  |  |  |  |
| LOC500629 |  | Pbxip1 |  |  |  |  |
| Coq3 |  | Ech1 |  |  |  |  |
| LOC498433 |  | LOC498076 |  |  |  |  |
| Stard3nl |  | Gstm1 |  |  |  |  |
| Dutp |  | LOC501553 |  |  |  |  |
| Kai1 |  | Ccnl2 |  |  |  |  |
| Pabpn1 |  | Ugcg |  |  |  |  |
| Fxyd6 |  | LOC499531 |  |  |  |  |
| Pigt |  | Myh10 |  |  |  |  |
| Polr2d |  | Hmgb2 |  |  |  |  |
| LOC313699 |  | Dvl1 |  |  |  |  |
| Evl |  | isg12(b) |  |  |  |  |
| Efnb1 |  | Msln |  |  |  |  |
| RGD1305133 |  | Atp1b2 |  |  |  |  |
| LOC301119 |  | LOC501087 |  |  |  |  |
| Rcn2 |  | LOC361942 |  |  |  |  |
| LOC292792 |  | LOC500960 |  |  |  |  |
| LOC361695 |  | LOC500398 |  |  |  |  |
| Ckap4 |  | LOC362587 |  |  |  |  |
| Psma5 |  | LOC362315 |  |  |  |  |
| Siat4b |  | Per1 |  |  |  |  |
| Tdg |  | LOC362543 |  |  |  |  |
| LOC309848 |  |  |  |  |  |  |
| Slc6a8 |  |  |  |  |  |  |
| Gorasp2 |  |  |  |  |  |  |
| Tpbg |  |  |  |  |  |  |
| Ctps |  |  |  |  |  |  |
| Abhd8 |  |  |  |  |  |  |
| LOC360821 |  |  |  |  |  |  |
| Tia1 |  |  |  |  |  |  |
| Tsnax |  |  |  |  |  |  |
| LOC316916 |  |  |  |  |  |  |
| Mospd3 |  |  |  |  |  |  |
| Rcn |  |  |  |  |  |  |
| Fut8 |  |  |  |  |  |  |
| LOC361315 |  |  |  |  |  |  |
| LOC289181 |  |  |  |  |  |  |
| Rarres2 |  |  |  |  |  |  |
| Zhx1 |  |  |  |  |  |  |
| MGC105691 |  |  |  |  |  |  |
| Bbp |  |  |  |  |  |  |
| Cntf |  |  |  |  |  |  |
| LOC500039 |  |  |  |  |  |  |
| Nov |  |  |  |  |  |  |
| Ifitm1 |  |  |  |  |  |  |
| LOC310640 |  |  |  |  |  |  |
| Pgrmc1 |  |  |  |  |  |  |
| Sc65 |  |  |  |  |  |  |
| Id3 |  |  |  |  |  |  |
| LOC500855 |  |  |  |  |  |  |
| Amd1 |  |  |  |  |  |  |
| RGD1310553 |  |  |  |  |  |  |
| Slc16a1 |  |  |  |  |  |  |
| Snrpa |  |  |  |  |  |  |
| LOC288707 |  |  |  |  |  |  |
| Col11a1 |  |  |  |  |  |  |
| LOC312915 |  |  |  |  |  |  |
| Col5a1 |  |  |  |  |  |  |
| Api5 |  |  |  |  |  |  |
| LOC361467 |  |  |  |  |  |  |
| LOC304091 |  |  |  |  |  |  |
| LOC499087 |  |  |  |  |  |  |
| LOC497673 |  |  |  |  |  |  |
| Mcmd6 |  |  |  |  |  |  |
| LOC300149 |  |  |  |  |  |  |
| Tm9sf4 |  |  |  |  |  |  |
| RGD1309685 |  |  |  |  |  |  |
| LOC303514 |  |  |  |  |  |  |
| MGC94686 |  |  |  |  |  |  |
| Sart2 |  |  |  |  |  |  |
| Ei24 |  |  |  |  |  |  |
| Bak1 |  |  |  |  |  |  |
| Sqle |  |  |  |  |  |  |
| Olfml2b |  |  |  |  |  |  |
| Lass2 |  |  |  |  |  |  |
| LOC498407 |  |  |  |  |  |  |
| LOC287212 |  |  |  |  |  |  |
| Lepre1 |  |  |  |  |  |  |
| Alg5 |  |  |  |  |  |  |
| Pon2 |  |  |  |  |  |  |
| LOC305310 |  |  |  |  |  |  |
| LOC298500 |  |  |  |  |  |  |
| Hspa14 |  |  |  |  |  |  |
| Arpc5 |  |  |  |  |  |  |
| Sara1 |  |  |  |  |  |  |
| Cycs |  |  |  |  |  |  |
| Cyp1b1 |  |  |  |  |  |  |
| Rhoq |  |  |  |  |  |  |
| Ripk5 |  |  |  |  |  |  |
| RGD1306395 |  |  |  |  |  |  |
| Gbl |  |  |  |  |  |  |
| Pigs |  |  |  |  |  |  |
| RGD1305625 |  |  |  |  |  |  |
| LOC366481 |  |  |  |  |  |  |
| Crot |  |  |  |  |  |  |
| RGD1305486 |  |  |  |  |  |  |
| Atp1b1 |  |  |  |  |  |  |
| Asf1a |  |  |  |  |  |  |
| Ppp1ca |  |  |  |  |  |  |
| LOC498410 |  |  |  |  |  |  |
| LOC361213 |  |  |  |  |  |  |
| LOC306587 |  |  |  |  |  |  |
| Mmp14 |  |  |  |  |  |  |
| RGD1310386 |  |  |  |  |  |  |
| Csrp2 |  |  |  |  |  |  |
| LOC289400 |  |  |  |  |  |  |
| RGD1305689 |  |  |  |  |  |  |
| Cxcl12 |  |  |  |  |  |  |
| Cdipt |  |  |  |  |  |  |
| LOC499084 |  |  |  |  |  |  |
| LOC498750 |  |  |  |  |  |  |
| LOC360886 |  |  |  |  |  |  |
| Pigq |  |  |  |  |  |  |
| Thumpd1 |  |  |  |  |  |  |
| Leprot |  |  |  |  |  |  |
| B4galt6 |  |  |  |  |  |  |
| Chd4 |  |  |  |  |  |  |
| Dab2 |  |  |  |  |  |  |
| LOC501028 |  |  |  |  |  |  |
| Psmb3 |  |  |  |  |  |  |
| LOC306324 |  |  |  |  |  |  |
| Colec12 |  |  |  |  |  |  |
| Id1 |  |  |  |  |  |  |
| Minpp1 |  |  |  |  |  |  |
| Txndc7 |  |  |  |  |  |  |
| Anxa11 |  |  |  |  |  |  |
| Rbm24 |  |  |  |  |  |  |
| Atp5j |  |  |  |  |  |  |
| LOC500252 |  |  |  |  |  |  |
| RAMP4 |  |  |  |  |  |  |
| LOC362040 |  |  |  |  |  |  |
| LOC499380 |  |  |  |  |  |  |
| Cfl2 |  |  |  |  |  |  |
| LOC360627 |  |  |  |  |  |  |
| Pcyox1 |  |  |  |  |  |  |
| Cbfb |  |  |  |  |  |  |
| LOC498736 |  |  |  |  |  |  |
| Reck |  |  |  |  |  |  |
| Crabp2 |  |  |  |  |  |  |
| Phc2 |  |  |  |  |  |  |
| MGC94167 |  |  |  |  |  |  |
| Nfatc4 |  |  |  |  |  |  |
| Unc50 |  |  |  |  |  |  |
| Mrps12 |  |  |  |  |  |  |
| c-fos |  |  |  |  |  |  |
| S100a16 |  |  |  |  |  |  |
| LOC499772 |  |  |  |  |  |  |
| Kazald1 |  |  |  |  |  |  |
| Kpna2 |  |  |  |  |  |  |
| LOC309475 |  |  |  |  |  |  |
| Mknk2 |  |  |  |  |  |  |
| Csf1 |  |  |  |  |  |  |
| Col3a1 |  |  |  |  |  |  |
| Pfn2 |  |  |  |  |  |  |
| Hmgcr |  |  |  |  |  |  |
| Prkaca |  |  |  |  |  |  |
| LOC317218 |  |  |  |  |  |  |
| Tmem9 |  |  |  |  |  |  |
| Cpt2 |  |  |  |  |  |  |
| Rbp1 |  |  |  |  |  |  |
| LOC499775 |  |  |  |  |  |  |
| Np |  |  |  |  |  |  |
| Dscr1 |  |  |  |  |  |  |
| Tnfrsf1a |  |  |  |  |  |  |
| RGD1310861 |  |  |  |  |  |  |
| Hn1 |  |  |  |  |  |  |
| Postn |  |  |  |  |  |  |
| Idh1 |  |  |  |  |  |  |
| LOC360698 |  |  |  |  |  |  |
| Ctsk |  |  |  |  |  |  |
