# Supplementary material for: Multiple Oxygen Tension Environments Reveal Diverse Patterns of Transcriptional Regulation in Primary Astrocytes
Source: PLoS One. 2011 Jun 27;6(6):e21638. doi: 10.1371/journal.pone.0021638 (PMC3124552; doi:10.1371/journal.pone.0021638)
Supplement: Table S5 — Significantly regulated transcripts common between 1% and 4% O2. Official gene symbols are employed to demonstrate the significantly regulated genes populating the Venn diagram intersections D, depicted in Figure 2. Positive z ratios indicate upregulation compared to 20% O2 and negative z ratios indicate downregulation compared to 20% O2. (DOC) [file pone.0021638.s011.doc]

**Table S5. Significantly regulated transcripts common between 1% and 4% O_2_.** Official gene symbols are employed to demonstrate the significantly regulated genes populating the Venn diagram intersections D, depicted in Figure 2. Positive z ratios indicate upregulation compared to 20% O_2_ and negative z ratios indicate downregulation compared to 20% O_2_.

| **Transcripts-common 1-4%** | **1% z ratio** | **4% z ratio** |
| --- | --- | --- |
| LOC501644 | 4.08 | 1.62 |
| Fth1 | 4.34 | 1.89 |
| LOC497684 | 3.63 | 1.5 |
| Vim | 3.98 | 1.96 |
| LOC500983 | 4.08 | 2.26 |
| Tagln | 3.4 | 1.58 |
| LOC289715 | 3.98 | 2.17 |
| Ctgf | 3.91 | 2.15 |
| Actg | 3.49 | 1.74 |
| Actb | 3.39 | 1.84 |
| Mt1a | 4.02 | 2.54 |
| Eef1a1 | 2.88 | 1.57 |
| LOC298169 | 2.81 | 1.61 |
| LOC498143 | 2.47 | 1.56 |
| LOC304035 | 2.4 | 1.52 |
| Fn1 | 2.3 | 1.52 |
| Slc3a2 | 2.39 | 1.65 |
| Rpl41 | 2.6 | 1.93 |
| LOC499906 | 2.25 | 1.6 |
| Rps18 | 2.45 | 1.83 |
| Rps14 | 2.19 | 1.68 |
| Icam1 | 1.99 | 1.55 |
| LOC361797 | 2.26 | 1.87 |
| Rpl29 | 2.34 | 1.96 |
| Rplp1 | 2.24 | 1.89 |
| LOC500859 | 2.01 | 1.81 |
| Rps11 | 2.05 | 1.86 |
| LOC293860 | 1.96 | 1.78 |
| LOC500929 | 1.9 | 1.73 |
| Rpl18 | 1.76 | 1.6 |
| LOC294748 | 1.87 | 1.72 |
| LOC500885 | 2.15 | 2 |
| LOC499457 | 1.8 | 1.7 |
| Rps3 | 1.75 | 1.65 |
| LOC361061 | 1.85 | 1.77 |
| Rpl35 | 1.78 | 1.73 |
| Rps27 | 2.26 | 2.23 |
| Ppia | 1.81 | 1.81 |
|  |  |  |
| LOC299935 | 1.52 | 3.09 |
| LOC302528 | 1.76 | 2.95 |
| LOC364108 | 1.56 | 2.74 |
| LOC498998 | 2.08 | 3.25 |
| Fau | 1.76 | 2.8 |
| LOC299041 | 1.53 | 2.5 |
| LOC500559 | 1.64 | 2.57 |
| Cfl1 | 1.76 | 2.51 |
| LOC499133 | 1.6 | 2.28 |
| LOC315642 | 1.64 | 2.28 |
| LOC314556 | 1.89 | 2.49 |
| Rpl19 | 2.07 | 2.65 |
| Rpl26 | 1.86 | 2.44 |
| Rps8 | 1.95 | 2.5 |
| LOC362181 | 1.94 | 2.45 |
| Rpl10 | 1.96 | 2.45 |
| LOC307135 | 1.71 | 2.19 |
| Rps25 | 1.59 | 2.07 |
| Rps27a | 2.11 | 2.57 |
| Rpl8 | 1.53 | 1.99 |
| LOC309408 | 1.75 | 2.2 |
| LOC498555 | 1.65 | 1.97 |
| LOC499305 | 1.51 | 1.81 |
| Rps20 | 1.77 | 2.01 |
| LOC298785 | 2.08 | 2.28 |
| Hnrpdl | 2.13 | 2.29 |
| Myd116 | 2.14 | 2.29 |
| LOC366193 | 2 | 2.13 |
| Rpl37a | 2.06 | 2.15 |
| LOC502302 | 1.89 | 1.91 |
| Rps15 | 1.51 | 1.53 |
|  |  |  |
| Igsf11 | -3.65 | -1.51 |
| Dnajc10 | -3.71 | -1.75 |
| LOC360546 | -3.08 | -1.72 |
| Rab11a | -2.95 | -1.69 |
| Aqp1 | -2.87 | -1.65 |
| Alcam | -3.45 | -2.37 |
| AY228474 | -2.46 | -1.76 |
| Sesn1 | -3.5 | -2.94 |
| Abcd3 | -2.07 | -1.51 |
| LOC302980 | -2.27 | -1.72 |
| MGC94969 | -2.24 | -1.75 |
| LOC294942 | -2.59 | -2.19 |
| Pros1 | -1.86 | -1.51 |
| Ucp2 | -1.91 | -1.56 |
| Tsn | -1.85 | -1.58 |
| LOC499593 | -2.3 | -2.03 |
| Hnrpa3 | -1.78 | -1.52 |
| Calm3 | -1.9 | -1.65 |
| LOC302378 | -1.99 | -1.77 |
| Lypla1 | -1.84 | -1.69 |
| Lyplal1 | -1.63 | -1.51 |
| RGD1308373 | -1.7 | -1.58 |
| AF146738 | -2.19 | -2.09 |
| Zic2 | -2.17 | -2.08 |
| LOC498751 | -1.9 | -1.85 |
|  |  |  |
| LOC290372 | -1.51 | -4.62 |
| Bmp7 | -2.49 | -4.77 |
| Efemp1 | -2.16 | -3.39 |
| Gjb2 | -4.43 | -5.43 |
| Ptgds | -6.25 | -7.17 |
| LOC294734 | -1.57 | -2.42 |
| Gprasp1 | -1.74 | -2.57 |
| Tfpi | -1.76 | -2.54 |
| Calm1 | -1.74 | -2.49 |
| Cfh | -2.28 | -2.83 |
| RGD1307008 | -1.53 | -2.06 |
| Dcn | -1.81 | -2.22 |
| Adprt | -1.59 | -1.88 |
| Ier2 | -1.54 | -1.76 |
| LOC296758 | -1.79 | -2 |
| LOC498564 | -1.69 | -1.88 |
| Cspg5 | -2.33 | -2.45 |
| Gap43 | -2.35 | -2.44 |
| LOC500441 | -1.58 | -1.6 |
| LOC309081 | -1.62 | -1.64 |
|  |  |  |
| Pdk1 | 3.97 | -1.86 |
| P4ha1 | 4.11 | -1.61 |
| Xpo1 | 2.95 | -1.69 |
| Klf5 | 1.89 | -2.27 |
| LOC500856 | 1.59 | -2.31 |
| Ednrb | 1.77 | -1.78 |
| LOC498398 | -1.67 | 1.54 |
| LOC293888 | -1.67 | 1.59 |
| Myadm | -1.79 | 1.61 |
| Boc | -1.67 | 2.14 |
| Adamts1 | -1.65 | 2.2 |
| Cirbp | -1.87 | 2.57 |
| Cspg2 | -2.85 | 1.78 |
| Col1a1 | -3.23 | 1.89 |
