# Supplementary material for: Multiple Oxygen Tension Environments Reveal Diverse Patterns of Transcriptional Regulation in Primary Astrocytes
Source: PLoS One. 2011 Jun 27;6(6):e21638. doi: 10.1371/journal.pone.0021638 (PMC3124552; doi:10.1371/journal.pone.0021638)
Supplement: Table S6 — Significantly regulated transcripts common between 4% and 9% O2. Official gene symbols are employed to demonstrate the significantly regulated genes populating the Venn diagram intersections F, depicted in Figure 2. Positive z ratios indicate upregulation compared to 20% O2 and negative z ratios indicate downregulation compared to 20% O2. (DOC) [file pone.0021638.s012.doc]

**Table S6. Significantly regulated transcripts common between 4% and 9% O_2_.** Official gene symbols are employed to demonstrate the significantly regulated genes populating the Venn diagram intersections F, depicted in Figure 2. Positive z ratios indicate upregulation compared to 20% O_2_ and negative z ratios indicate downregulation compared to 20% O_2_.

| **Transcripts-common 4-9%** | **4% z ratio** | **9% z ratio** |
| --- | --- | --- |
| Gdf10 | 6.63 | 1.91 |
| Rbm3 | 6.11 | 1.87 |
| Ca3 | 7.28 | 5.19 |
| Serpine1 | 2.86 | 1.54 |
| Ass | 3.11 | 2.08 |
| Pdlim7 | 2.58 | 1.63 |
| Pcolce | 2.48 | 1.53 |
| Snai1 | 4.97 | 4.13 |
| LOC501203 | 2.44 | 1.61 |
| Mk1 | 2.27 | 1.61 |
| Hmgn2 | 2.29 | 1.84 |
| Serpine2 | 2.92 | 2.61 |
| LOC366485 | 1.88 | 1.67 |
| Nfkbia | 2.26 | 2.06 |
| LOC366656 | 1.94 | 1.78 |
| Lum | 2.64 | 2.51 |
| LOC294781 | 1.94 | 1.82 |
| LOC298495 | 1.72 | 1.62 |
| Arpc1b | 1.7 | 1.64 |
| Bgn | 2.06 | 2.06 |
| Fstl3 | 1.53 | 5.5 |
| Mfap4 | 2.67 | 4.49 |
| Hes1 | 2.48 | 3.94 |
| Rhoa | 2 | 3.36 |
| Tm4sf1 | 1.51 | 2.71 |
| Fdps | 1.66 | 2.75 |
| LOC500645 | 1.74 | 2.64 |
| Stmn2 | 1.62 | 2.42 |
| LOC302388 | 1.92 | 2.54 |
| Col8a1 | 1.57 | 2.18 |
| LOC310512 | 1.73 | 2.32 |
| LOC366999 | 1.72 | 2.26 |
| LOC299907 | 1.67 | 2.19 |
| Akr1a1 | 1.86 | 2.13 |
| Lxn | 1.53 | 1.78 |
| LOC300278 | 1.58 | 1.81 |
| Atf5 | 1.59 | 1.79 |
| Colm | 1.72 | 1.85 |
| RGD1307627 | 1.5 | 1.59 |
| LOC302497 | 1.6 | 1.68 |
| Rpl10a | 1.79 | 1.84 |
| Gdi2 | 1.55 | 1.6 |
| LOC310360 | 1.91 | 1.94 |
|  |  |  |
| LOC360747 | -4.66 | -1.54 |
| LOC365476 | -5.54 | -2.91 |
| OSP94 | -4.24 | -1.89 |
| Giot1 | -3.42 | -1.57 |
| Mycl1 | -4.68 | -3.05 |
| Fank1 | -3.73 | -2.11 |
| LOC500939 | -3.4 | -2.24 |
| Dncl2b | -3.19 | -2.04 |
| LOC498356 | -3.22 | -2.1 |
| Esm1 | -4.13 | -3.17 |
| Ltbp4 | -2.66 | -1.79 |
| Edg2 | -2.51 | -1.66 |
| Abhd3 | -2.42 | -1.69 |
| LOC292477 | -2.58 | -1.9 |
| Myh14 | -2.17 | -1.61 |
| Tf | -2.12 | -1.57 |
| LOC499589 | -2.62 | -2.09 |
| LOC317575 | -2.99 | -2.53 |
| LOC500671 | -2.18 | -1.84 |
| LOC305035 | -2.04 | -1.72 |
| Slc27a1 | -2.14 | -1.86 |
| Rtn1 | -2.34 | -2.13 |
| Lpl | -1.77 | -1.63 |
| LOC315804 | -1.86 | -1.73 |
| Dnah1 | -2.88 | -2.78 |
| Cox17 | -1.69 | -1.59 |
| Sparcl1 | -1.91 | -1.83 |
| LOC306991 | -2.14 | -2.09 |
|  |  |  |
| Lgi4 | -1.71 | -3.2 |
| LOC497766 | -1.6 | -2.93 |
| F3 | -1.89 | -3.05 |
| Cotl1 | -1.69 | -2.61 |
| LOC498931 | -1.53 | -2.44 |
| LOC304919 | -1.78 | -2.66 |
| Pik3c3 | -1.61 | -2.43 |
| P2rxl1 | -1.75 | -2.51 |
| LOC499798 | -1.51 | -2.26 |
| Slc25a29 | -1.52 | -2.24 |
| LOC500865 | -1.7 | -2.41 |
| Aif1 | -1.56 | -2.27 |
| Tcfap2b | -2.64 | -3.31 |
| Snrpa1 | -1.7 | -2.28 |
| Psmd4 | -1.58 | -2.15 |
| Polr2i | -1.59 | -2.13 |
| Lig1 | -1.67 | -2.17 |
| Phr1 | -1.91 | -2.38 |
| Zfp36l2 | -1.64 | -2.06 |
| Podxl | -1.52 | -1.93 |
| Ptgs2 | -6.05 | -6.44 |
| Trim39 | -2.45 | -2.83 |
| Sorl1 | -1.54 | -1.84 |
| LOC499094 | -1.63 | -1.89 |
| Pex13 | -1.94 | -2.15 |
| Ppt | -2.6 | -2.76 |
| MGC72974 | -1.5 | -1.63 |
| Sostdc1 | -3.9 | -3.9 |
|  |  |  |
| LOC498048 | 2.27 | -3.06 |
| LOC361912 | 2.22 | -2.93 |
| Nqo1 | 3.4 | -1.6 |
| LOC500867 | 2.3 | -2.52 |
| LOC363492 | 2.14 | -2.47 |
| Tnn | 2.49 | -1.65 |
| Snrp70 | 1.51 | -2.45 |
| Gstm2 | 2.14 | -1.51 |
| Ifitm3 | 1.74 | -1.87 |
| Prpf39 | 1.57 | -1.87 |
| Arpp19 | 1.53 | -1.55 |
| A2m | -3.64 | 1.58 |
| RGD1306222 | -2.92 | 2.18 |
| Cte1 | -2.25 | 1.83 |
| Maob | -2.12 | 1.57 |
| Lcat | -1.62 | 1.94 |
| Wdr34 | -1.65 | 1.66 |
