# Supplementary material for: Multiple Oxygen Tension Environments Reveal Diverse Patterns of Transcriptional Regulation in Primary Astrocytes
Source: PLoS One. 2011 Jun 27;6(6):e21638. doi: 10.1371/journal.pone.0021638 (PMC3124552; doi:10.1371/journal.pone.0021638)
Supplement: Table S7 — Significantly regulated transcripts common between 1% and 9% O2. Official gene symbols are employed to demonstrate the significantly regulated genes populating the Venn diagram intersections E, depicted in Figure 2. Positive z ratios indicate upregulation compared to 20% O2 and negative z ratios indicate downregulation compared to 20% O2. (DOC) [file pone.0021638.s013.doc]

**Table S7. Significantly regulated transcripts common between 1% and 9% O_2_.** Official gene symbols are employed to demonstrate the significantly regulated genes populating the Venn diagram intersections E, depicted in Figure 2. Positive z ratios indicate upregulation compared to 20% O_2_ and negative z ratios indicate downregulation compared to 20% O_2_.

| **Transcripts-common 1-9%** | **1% z ratio** | **9% z ratio** |
| --- | --- | --- |
| Bnip3 | 5 | 1.82 |
| LOC501140 | 4.67 | 1.64 |
| Hspb1 | 4.47 | 1.54 |
| LOC500506 | 3.77 | 1.93 |
| Uap1l1 | 3.34 | 1.66 |
| Gpi | 3.32 | 1.67 |
| Ppp1r3c | 3.83 | 2.26 |
| LOC497927 | 3.17 | 1.64 |
| Gdf15 | 3.78 | 2.34 |
| Pfkl | 3.24 | 1.9 |
| LOC500959 | 4.29 | 3.03 |
| LOC292656 | 3.53 | 2.34 |
| Gadd45b | 3.5 | 2.33 |
| LOC498731 | 4.53 | 3.4 |
| LOC364048 | 3.87 | 2.77 |
| Cyr61 | 2.99 | 1.97 |
| Tpi1 | 4.62 | 3.66 |
| Hspca | 2.57 | 1.69 |
| Gapd | 3.81 | 3.03 |
| LOC502770 | 2.96 | 2.2 |
| Rps10 | 2.52 | 1.78 |
| LOC306115 | 2.46 | 1.77 |
| Flcn | 2.9 | 2.26 |
| Rpl21 | 2.24 | 1.6 |
| Rpl32 | 2.13 | 1.55 |
| Vars2 | 2.03 | 1.5 |
| LOC499845 | 2.17 | 1.66 |
| LOC499423 | 2.09 | 1.71 |
| Bri3 | 2.17 | 1.82 |
| Dpp7 | 2.16 | 1.82 |
| Ddit4 | 2.02 | 1.7 |
| LOC497816 | 1.77 | 1.52 |
| LOC363531 | 1.75 | 1.64 |
| Hspa8 | 1.9 | 1.86 |
|  |  |  |
| Eno1 | 2.09 | 3.89 |
| P4hb | 1.86 | 2.49 |
| LOC296582 | 1.7 | 2.42 |
| LOC310585 | 2.07 | 2.42 |
| Bsg | 1.58 | 2.16 |
| Cd63 | 1.82 | 2.15 |
| Rpl24 | 1.61 | 1.89 |
| Calr | 1.65 | 1.85 |
| Rps2 | 1.59 | 1.8 |
| Tm4sf9 | 1.61 | 1.75 |
| Eif3s5 | 1.58 | 1.68 |
|  |  |  |
| Dhcr7 | -4.33 | -1.7 |
| LOC306229 | -3.26 | -1.96 |
| Gusb | -2.65 | -1.53 |
| Sphk1 | -2.59 | -1.59 |
| Hsd3b7 | -2.67 | -1.83 |
| Lrrc5 | -2.4 | -1.73 |
| Lamb1-1 | -2.18 | -1.53 |
| Scara3 | -2.61 | -2 |
| Pdgfra | -3.2 | -2.78 |
| LOC498095 | -1.99 | -1.58 |
| Col6a3 | -2.06 | -1.72 |
| Ltap | -2.24 | -2 |
| Hspa2 | -2.29 | -2.11 |
| Btg2 | -2.83 | -2.7 |
| RGD1311463 | -1.56 | -1.5 |
| Lsm7 | -2.25 | -2.22 |
| Ppap2b | -2.16 | -2.14 |
|  |  |  |
| Txnip | -6.69 | -11.08 |
| Srrm2 | -1.73 | -4.43 |
| LOC316085 | -1.58 | -4.06 |
| Hmgn3 | -2.09 | -4.15 |
| Sfrs5 | -1.9 | -3.39 |
| Stc1 | -1.66 | -3.09 |
| Gstt2 | -1.87 | -3.21 |
| Ahr | -1.58 | -2.64 |
| Fbn2 | -1.8 | -2.81 |
| Zfp36 | -2.52 | -3.44 |
| Agrn | -1.84 | -2.69 |
| Mgst2 | -1.61 | -2.32 |
| Zcwcc1 | -1.65 | -2.35 |
| LOC362317 | -1.64 | -2.24 |
| LOC293844 | -1.8 | -2.33 |
| Zfp462 | -1.71 | -2.2 |
| Lphn1 | -1.71 | -2.09 |
| Srpr | -1.68 | -2.05 |
| Atp2a2 | -1.92 | -2.25 |
| Ywhab | -1.79 | -2.07 |
| Rnf7 | -1.59 | -1.85 |
| Prss35 | -2.27 | -2.47 |
| Sec5l1 | -1.75 | -1.93 |
| Nup107 | -1.5 | -1.66 |
| Snx14 | -1.54 | -1.67 |
| LOC360602 | -1.76 | -1.86 |
| RGD1307010 | -1.71 | -1.75 |
| LOC296469 | -1.59 | -1.63 |
| Polr2b | -1.66 | -1.67 |
| LOC498674 | -2.29 | -2.29 |
| LOC500242 | 2.25 | 2.25 |
|  |  |  |
| Hmox1 | 5.9 | -1.7 |
| Sv2b | 2.92 | -3.84 |
| Cryab | 3.38 | -1.96 |
| Ccnl1 | 1.65 | -2.43 |
| Wsb1 | 2.51 | -1.51 |
| Luc7l | 2.39 | -1.6 |
| Lama5 | 1.6 | -1.96 |
| Eln | -1.9 | 13.01 |
| MGC95138 | -3.33 | 2.72 |
| Hmgcs1 | -3.63 | 2.03 |
| Cd164l1 | -3.52 | 1.54 |
| Chdh | -3.22 | 1.77 |
| Cx3cl1 | -2.84 | 1.89 |
| LOC497841 | -2.02 | 2.65 |
| Loxl1 | -2.7 | 1.87 |
| Cerk | -1.53 | 2.75 |
| Sdfr1 | -2.23 | 1.72 |
| LOC362264 | -2.22 | 1.73 |
| LOC363443 | -2.29 | 1.6 |
| Ttc13 | -2.12 | 1.72 |
| LOC499125 | -1.7 | 2.01 |
| LOC361178 | -2.08 | 1.52 |
| Mig12 | -1.91 | 1.59 |
| Lactb | -1.82 | 1.66 |
| Mtvr2 | -1.57 | 1.78 |
| Nde1 | -1.53 | 1.71 |
| LOC499882 | -1.63 | 1.6 |
| RGD1310571 | -1.6 | 1.55 |
