# Supplementary material for: Multiple Oxygen Tension Environments Reveal Diverse Patterns of Transcriptional Regulation in Primary Astrocytes
Source: PLoS One. 2011 Jun 27;6(6):e21638. doi: 10.1371/journal.pone.0021638 (PMC3124552; doi:10.1371/journal.pone.0021638)
Supplement: Table S8 — Significantly populated PAGE gene collections created with transcripts responding to 1% O2 tension compared to control 20% O2 condition. Significantly-regulated genes were used to populate the specified MSigDB collections. ‘# genes in collection’ describes the total gene count of the specific MSigDB collection and the ‘# exp genes in collection’ describes the number of genes from the input experimental set that were able to significantly populate the specific MSigDB collection. The Z score is calculated based upon the cumulative z ratios of the respective genes from the experimental dataset that populated the specific MSigDB collection. (DOC) [file pone.0021638.s014.doc]

**Table S8. Significantly populated PAGE gene collections created with transcripts responding to 1% O_2_ tension compared to control 20% O_2_ condition.** Significantly-regulated genes were used to populate the specified MSigDB collections. ‘# genes in collection’ describes the total gene count of the specific MSigDB collection and the ‘# exp genes in collection’ describes the number of genes from the input experimental set that were able to significantly populate the specific MSigDB collection. The Z score is calculated based upon the cumulative z ratios of the respective genes from the experimental dataset that populated the specific MSigDB collection

| **MSigDB collection** | **# genes in collection** | **# exp genes in collection** | **Z Score** |
| --- | --- | --- | --- |
| MENSE_HYPOXIA_UP | 109 | 16 | 15.94246 |
| RIBOSOMAL_PROTEINS | 203 | 49 | 13.43949 |
| HYPOXIA_REVIEW | 81 | 26 | 11.97456 |
| GLYCOLYSIS_AND_GLUCONEOGENESIS | 44 | 12 | 11.9605 |
| GLYCOLYSIS | 56 | 11 | 11.81789 |
| GLUCONEOGENESIS | 56 | 11 | 11.81789 |
| HYPOXIA_REG_UP | 38 | 12 | 11.61698 |
| HIF1_TARGETS | 36 | 15 | 11.47513 |
| ROME_INSULIN_2F_UP | 235 | 29 | 9.531574 |
| FRUCTOSE_AND_MANNOSE_METABOLISM | 25 | 4 | 9.458731 |
| GLYCOLYSISPATHWAY | 10 | 6 | 9.41061 |
| UVB_NHEK1_UP | 177 | 22 | 9.293641 |
| LVAD_HEARTFAILURE_UP | 90 | 18 | 8.933959 |
| GENOTOXINS_ALL_24HRS_REG | 28 | 6 | 7.793947 |
| HYPERTROPHY_MODEL | 20 | 5 | 7.358599 |
| CARBON_FIXATION | 23 | 4 | 7.178731 |
| HIPPOCAMPUS_DEVELOPMENT_POSTNATAL | 45 | 8 | 6.977349 |
| MTA3PATHWAY | 16 | 4 | 6.828731 |
| BRCA_PROGNOSIS_NEG | 95 | 5 | 6.812998 |
| P53_SIGNALING | 101 | 12 | 6.784554 |
| NING_COPD_UP | 180 | 30 | 6.652267 |
| ET743_SARCOMA_UP | 71 | 10 | 6.627709 |
| MENSSEN_MYC_UP | 35 | 10 | 6.621384 |
| INSULIN_SIGNALING | 103 | 12 | 6.602689 |
| TSA_PANC50_UP | 39 | 9 | 6.520596 |
| GILDEA_BLADDER_UP | 30 | 3 | 6.518629 |
| CMV_HCMV_TIMECOURSE_8HRS_UP | 20 | 3 | 6.489761 |
| HYPOXIA_FIBRO_UP | 20 | 9 | 6.450596 |
| HDACI_COLON_CUR_UP | 108 | 18 | 6.327092 |
| PENTOSE_PHOSPHATE_PATHWAY | 27 | 3 | 6.299236 |
| HDACI_COLON_CUR24HRS_UP | 37 | 9 | 6.167263 |
| ZUCCHI_EPITHELIAL_UP | 50 | 13 | 6.154191 |
| ELONGINA_KO_DN | 184 | 17 | 6.128078 |
| EGF_HDMEC_UP | 43 | 9 | 6.027263 |
| STRESS_ARSENIC_SPECIFIC_UP | 149 | 8 | 5.980329 |
| AGED_MOUSE_CORTEX_UP | 31 | 11 | 5.826864 |
| WALKER_MM_SNP_DIFF | 46 | 7 | 5.778343 |
| ET743_SARCOMA_6HRS_UP | 30 | 6 | 5.764953 |
| JISON_SICKLECELL_DIFF | 385 | 52 | 5.754599 |
| MYC_TARGETS | 42 | 12 | 5.652948 |
| TUMOR_SUPRESSOR | 26 | 3 | 5.54868 |
| CORDERO_KRAS_KD_VS_CONTROL_UP | 84 | 20 | 5.511306 |
| ZUCCHI_EPITHELIAL_DN | 50 | 10 | 5.508262 |
| FLECHNER_KIDNEY_TRANSPLANT_REJECTION_PBL_DN | 51 | 4 | 5.458731 |
| O6BG_RESIST_MEDULLOBLASTOMA_UP | 25 | 5 | 5.448997 |
| MARCINIAK_CHOP_DIFF | 26 | 3 | 5.43321 |
| P38MAPKPATHWAY | 40 | 4 | 5.428731 |
| HYPOXIA_RCC_UP | 104 | 7 | 5.321006 |
| POMEROY_DESMOPLASIC_VS_CLASSIC_MD_UP | 49 | 11 | 5.278113 |
| UVB_NHEK1_C2 | 22 | 5 | 5.207502 |
| FLOTHO_CASP8AP2_MRD_DIFF | 90 | 14 | 5.189176 |
| HDACI_COLON_CUR2HRS_UP | 29 | 7 | 5.150922 |
| VERNELL_PRB_CLSTR2 | 23 | 4 | 5.028731 |
| HUMAN_CD34_ENRICHED_TRANSCRIPTION_FACTORS | 202 | 19 | 5.022012 |
| CALCINEURIN_NF_AT_SIGNALING | 100 | 11 | 4.877103 |
| ET743_SARCOMA_24HRS_UP | 10 | 3 | 4.826993 |
| ET743_SARCOMA_72HRS_UP | 67 | 10 | 4.818886 |
| UVB_NHEK1_C1 | 52 | 6 | 4.77291 |
| ZELLER_MYC_UP | 27 | 9 | 4.70393 |
| HIPPOCAMPUS_DEVELOPMENT_PRENATAL | 35 | 8 | 4.689859 |
| CMV_HCMV_6HRS_DN | 56 | 7 | 4.648229 |
| KNUDSEN_PMNS_DN | 233 | 17 | 4.641335 |
| BREAST_CANCER_ESTROGEN_SIGNALING | 101 | 17 | 4.597678 |
| FERNANDEZ_MYC_TARGETS | 186 | 39 | 4.588913 |
| BASSO_GERMINAL_CENTER_CD40_UP | 108 | 7 | 4.542399 |
| PTDINSPATHWAY | 23 | 4 | 4.523731 |
| NGUYEN_KERATO_DN | 86 | 8 | 4.283272 |
| DRUG_RESISTANCE_AND_METABOLISM | 100 | 12 | 4.25576 |
| CIS_RESIST_LUNG_DN | 11 | 6 | 3.980908 |
| MA_ATRA_EMP_UP | 42 | 8 | 3.954468 |
| DNA_REPLICATION_REACTOME | 51 | 3 | 3.781989 |
| UVB_NHEK2_UP | 69 | 10 | 3.689953 |
| METASTASIS_ADENOCARC_UP | 14 | 3 | 3.608784 |
| CROONQUIST_IL6_STROMA_UP | 40 | 5 | 3.5707 |
| BREASTCA_THREE_CLASSES | 42 | 5 | 3.499146 |
| IFN_BETA_GLIOMA_DN | 44 | 7 | 3.344252 |
| SANA_IFNG_ENDOTHELIAL_DN | 90 | 12 | 3.282925 |
| TRANSLATION_FACTORS | 56 | 5 | 3.078765 |
| SIG_CHEMOTAXIS | 45 | 4 | 3.033731 |
| XU_ATRA_PLUSNSC_DN | 15 | 3 | 2.915963 |
| AGED_MOUSE_HYPOTH_UP | 44 | 5 | 2.881991 |
| ST_INTEGRIN_SIGNALING_PATHWAY | 82 | 5 | 2.349807 |
| UVB_NHEK3_C2 | 43 | 6 | 2.082553 |
| IFN_BETA_UP | 65 | 4 | 1.883731 |
| SIG_BCR_SIGNALING_PATHWAY | 46 | 3 | 1.853639 |
| IL6_FIBRO_UP | 47 | 3 | -1.14858 |
| LOTEM_LEUKEMIA_UP | 26 | 4 | -1.56627 |
| HDACI_COLON_TSA_DN | 64 | 5 | -1.82717 |
| CHEMICALPATHWAY | 22 | 3 | -1.87027 |
| ROSS_AML1_ETO | 87 | 4 | -1.93627 |
| CORTEX_ENRICHMENT_LATE_UP | 20 | 3 | -1.99151 |
| CIS_XPC_UP | 151 | 14 | -2.13913 |
| VERNELL_PRB_CLSTR1 | 69 | 4 | -2.19627 |
| UVC_LOW_C2_DN | 20 | 3 | -2.31483 |
| SANSOM_APC_4_DN | 88 | 4 | -2.35627 |
| CASPASEPATHWAY | 23 | 4 | -2.39127 |
| D4GDIPATHWAY | 13 | 3 | -2.41298 |
| GALE_FLT3ANDAPL_UP | 61 | 6 | -2.46533 |
| HDACPATHWAY | 30 | 5 | -2.52929 |
| BECKER_TAMOXIFEN_RESISTANT_DN | 53 | 4 | -2.53627 |
| GUO_HEX_UP | 88 | 10 | -2.53657 |
| AT1RPATHWAY | 34 | 3 | -2.55154 |
| ST_GA12_PATHWAY | 23 | 4 | -2.57127 |
| CHESLER_BRAIN_HIGHEST_VARIANCE_GENES | 50 | 3 | -2.6266 |
| LDLPATHWAY | 6 | 3 | -2.70743 |
| H2O2_CSBDIFF_C1 | 33 | 3 | -2.7998 |
| NI2_MOUSE_DN | 46 | 4 | -2.85627 |
| CCR5PATHWAY | 18 | 7 | -2.89972 |
| DNMT1_KO_DN | 16 | 3 | -2.93259 |
| GH_GHRHR_KO_24HRS_DN | 172 | 9 | -2.94274 |
| HDACI_COLON_SUL24HRS_DN | 128 | 4 | -2.95627 |
| POD1_KO_MOST_UP | 32 | 9 | -2.96274 |
| STAEGE_EFTS_UP | 33 | 4 | -3.01127 |
| MMS_MOUSE_LYMPH_HIGH_4HRS_UP | 36 | 6 | -3.31857 |
| ALCALAY_AML_NPMC_DN | 193 | 13 | -3.32564 |
| EDG1PATHWAY | 26 | 3 | -3.39448 |
| TNFR1PATHWAY | 29 | 4 | -3.59127 |
| CMV_HCMV_TIMECOURSE_20HRS_UP | 86 | 5 | -3.63391 |
| FATTY_ACID_METABOLISM | 94 | 6 | -3.64109 |
| SERUM_FIBROBLAST_CELLCYCLE | 138 | 9 | -3.79607 |
| PRMT5_KD_UP | 187 | 29 | -3.93319 |
| VEGF_HUVEC_UP | 15 | 4 | -4.12627 |
| CPR_LOW_LIVER_UP | 17 | 3 | -4.27205 |
| ASTON_DEPRESSION_DN | 160 | 8 | -4.29393 |
| GH_AUTOCRINE_UP | 220 | 18 | -4.35493 |
| CALCIUM_REGULATION_IN_CARDIAC_CELLS | 144 | 16 | -4.48004 |
| HASLINGER_B_CLL_11Q23 | 23 | 5 | -4.76983 |
| BIOSYNTHESIS_OF_STEROIDS | 16 | 4 | -5.12627 |
| CHOLESTEROL_BIOSYNTHESIS | 18 | 4 | -5.12627 |
| HSC_LTHSC_FETAL | 274 | 14 | -5.27945 |
| HSC_LTHSC_SHARED | 274 | 14 | -5.27945 |
| HSC_LTHSC_ADULT | 370 | 16 | -5.51504 |
| GAY_YY1_DN | 281 | 20 | -5.80767 |
| REOVIRUS_HEK293_DN | 231 | 22 | -5.92036 |
