# Supplementary material for: Multiple Oxygen Tension Environments Reveal Diverse Patterns of Transcriptional Regulation in Primary Astrocytes
Source: PLoS One. 2011 Jun 27;6(6):e21638. doi: 10.1371/journal.pone.0021638 (PMC3124552; doi:10.1371/journal.pone.0021638)
Supplement: Table S9 — Significantly populated PAGE gene collections created with transcripts responding to 4% O2 tension compared to control 20% O2 condition. Significantly-regulated genes were used to populate the specified MSigDB collections. ‘# genes in collection’ describes the total gene count of the specific MSigDB collection and the ‘# exp genes in collection’ describes the number of genes from the input experimental set that were able to significantly populate the specific MSigDB collection. The Z score is calculated based upon the cumulative z ratios of the respective genes from the experimental dataset that populated the specific MSigDB collection. (DOC) [file pone.0021638.s015.doc]

**Table S9. Significantly populated PAGE gene collections created with transcripts responding to 4% O_2_ tension compared to control 20% O_2_ condition.** Significantly-regulated genes were used to populate the specified MSigDB collections. ‘# genes in collection’ describes the total gene count of the specific MSigDB collection and the ‘# exp genes in collection’ describes the number of genes from the input experimental set that were able to significantly populate the specific MSigDB collection. The Z score is calculated based upon the cumulative z ratios of the respective genes from the experimental dataset that populated the specific MSigDB collection

| **MSigDB collection** | **# genes in collection** | **# exp genes in collection** | **Z Score** |
| --- | --- | --- | --- |
| RIBOSOMAL_PROTEINS | 203 | 49 | 12.08692 |
| FLOTHO_CASP8AP2_MRD_DIFF | 90 | 14 | 5.481408 |
| POMEROY_DESMOPLASIC_VS_CLASSIC_MD_UP | 49 | 11 | 5.470601 |
| JISON_SICKLECELL_DIFF | 385 | 52 | 5.400567 |
| AGEING_KIDNEY_UP | 411 | 49 | 5.202632 |
| IFN_BETA_GLIOMA_DN | 44 | 7 | 4.902114 |
| FERNANDEZ_MYC_TARGETS | 186 | 39 | 4.85397 |
| CORDERO_KRAS_KD_VS_CONTROL_UP | 84 | 20 | 4.747348 |
| EGF_HDMEC_UP | 43 | 9 | 4.632284 |
| HIPPOCAMPUS_DEVELOPMENT_PRENATAL | 35 | 8 | 4.429428 |
| NADLER_OBESITY_UP | 59 | 13 | 4.393506 |
| CROONQUIST_IL6_STROMA_UP | 40 | 5 | 4.34365 |
| CHEN_LUNG_SURVIVAL | 29 | 4 | 4.332079 |
| TNFALPHA_4HRS_UP | 40 | 6 | 4.299359 |
| NAKAJIMA_MCS_UP | 101 | 9 | 4.252284 |
| TGFBETA_EARLY_UP | 47 | 12 | 4.177843 |
| UVB_NHEK1_C1 | 52 | 6 | 4.172802 |
| ZHAN_MM_CD138_MS_VS_REST | 47 | 3 | 4.131298 |
| ZHAN_TONSIL_BONEMARROW | 50 | 5 | 4.026128 |
| ZHAN_MMPC_LATEVS | 47 | 6 | 3.911523 |
| PASSERINI_EM | 42 | 8 | 3.895562 |
| TGFBETA_ALL_UP | 80 | 13 | 3.894276 |
| HDACI_COLON_CUR2HRS_UP | 29 | 7 | 3.847593 |
| IL6_FIBRO_UP | 47 | 3 | 3.790661 |
| VEGF_MMMEC_6HRS_UP | 50 | 8 | 3.77889 |
| HIF1_TARGETS | 36 | 15 | 3.702079 |
| BRCA1_SW480_UP | 25 | 4 | 3.687079 |
| BYSTRYKH_HSC_BRAIN_TRANS_GLOCUS | 218 | 11 | 3.685654 |
| CIS_RESIST_LUNG_DN | 11 | 6 | 3.637997 |
| PGC | 425 | 42 | 3.619748 |
| HYPOXIA_RCC_UP | 104 | 7 | 3.609476 |
| LIZUKA_L0_SM_L1 | 21 | 4 | 3.602079 |
| DRUG_RESISTANCE_AND_METABOLISM | 100 | 12 | 3.583172 |
| TUMOR_SUPRESSOR | 26 | 3 | 3.582815 |
| UVB_NHEK2_UP | 69 | 10 | 3.571917 |
| RHOPATHWAY | 31 | 4 | 3.542079 |
| BRCA2_BRCA1_UP | 49 | 3 | 3.501986 |
| SIG_CHEMOTAXIS | 45 | 4 | 3.492079 |
| NING_COPD_UP | 180 | 30 | 3.47734 |
| DNA_REPLICATION_REACTOME | 51 | 3 | 3.403837 |
| HTERT_UP | 69 | 5 | 3.337419 |
| GNATENKO_PLATELET_UP | 48 | 6 | 3.33181 |
| GNATENKO_PLATELET | 48 | 6 | 3.33181 |
| ZUCCHI_EPITHELIAL_UP | 50 | 13 | 3.264691 |
| METASTASIS_ADENOCARC_DN | 34 | 4 | 3.257079 |
| TPA_RESIST_EARLY_UP | 30 | 4 | 3.242079 |
| FSH_OVARY_MCV152_DN | 46 | 4 | 3.237079 |
| ACTINYPATHWAY | 19 | 4 | 3.222079 |
| SALMONELLAPATHWAY | 12 | 4 | 3.222079 |
| VERNELL_PRB_CLSTR2 | 23 | 4 | 3.212079 |
| SERUM_FIBROBLAST_CORE_DN | 197 | 20 | 3.191045 |
| CHESLER_BRAIN_ONLY_SUBSET | 26 | 4 | 3.187079 |
| CALRES_MOUSE_UP | 30 | 5 | 3.185367 |
| GLYCOLYSIS | 56 | 11 | 3.148964 |
| GLUCONEOGENESIS | 56 | 11 | 3.148964 |
| ROME_INSULIN_2F_UP | 235 | 29 | 3.128528 |
| INSULIN_SIGNALING | 103 | 12 | 3.115518 |
| SIG_REGULATION_OF_THE_ACTIN_CYTOSKELETON_BY_RHO_GTPASES | 35 | 5 | 3.02437 |
| MARCINIAK_CHOP_DIFF | 26 | 3 | 3.017012 |
| FLECHNER_KIDNEY_TRANSPLANT_REJECTION_PBL_DN | 51 | 4 | 2.982079 |
| CHANG_SERUM_RESPONSE_DN | 194 | 20 | 2.962966 |
| HDACI_COLON_CUR_UP | 108 | 18 | 2.921225 |
| ALZHEIMERS_INCIPIENT_DN | 144 | 7 | 2.895123 |
| TGFBETA_C2_UP | 18 | 6 | 2.882737 |
| DFOSB_BRAIN_2WKS_UP | 39 | 4 | 2.847079 |
| P53_SIGNALING | 101 | 12 | 2.835503 |
| ZELLER_MYC_UP | 27 | 9 | 2.828951 |
| MA_ATRA_EMP_UP | 42 | 8 | 2.785405 |
| ST_INTEGRIN_SIGNALING_PATHWAY | 82 | 5 | 2.77393 |
| METASTASIS_ADENOCARC_UP | 14 | 3 | 2.762978 |
| HALMOS_CEBP_DN | 46 | 5 | 2.720264 |
| GLYCOLYSISPATHWAY | 10 | 6 | 2.662283 |
| TPA_RESIST_MIDDLE_UP | 49 | 5 | 2.644238 |
| MYOD_NIH3T3_UP | 82 | 6 | 2.621458 |
| RADIATION_SENSITIVITY | 27 | 3 | 2.61864 |
| YAGI_AML_PROG_ASSOC | 130 | 9 | 2.492284 |
| PENG_LEUCINE_DN | 180 | 22 | 2.417241 |
| MTA3PATHWAY | 16 | 4 | 2.372079 |
| MATRIX_METALLOPROTEINASES | 32 | 4 | 2.362079 |
| YAO_P4_KO_VS_WT_DN | 23 | 4 | 2.332079 |
| MYC_TARGETS | 42 | 12 | 2.313001 |
| TRANSLATION_FACTORS | 56 | 5 | 2.214913 |
| GILDEA_BLADDER_UP | 30 | 3 | 2.15676 |
| PROTEASOMEPATHWAY | 21 | 4 | 2.132079 |
| GLYCOLYSIS_AND_GLUCONEOGENESIS | 44 | 12 | 2.131136 |
| CARBON_FIXATION | 23 | 4 | 2.092079 |
| NADLER_OBESITY_DN | 39 | 8 | 2.081833 |
| DER_IFNG_UP | 64 | 7 | 1.825483 |
| HIPPOCAMPUS_DEVELOPMENT_POSTNATAL | 45 | 8 | 1.710602 |
| DAVIES_N | 24 | 4 | 1.542079 |
| ZHAN_MM_MOLECULAR_CLASSI_DN | 50 | 3 | -0.75886 |
| ET743_SARCOMA_24HRS_UP | 10 | 3 | -0.90897 |
| SMITH_HTERT_UP | 117 | 9 | -1.45438 |
| CMV_HCMV_TIMECOURSE_4HRS_DN | 35 | 3 | -1.51519 |
| DAC_FIBRO_DN | 11 | 3 | -1.79809 |
| FATTY_ACID_METABOLISM | 94 | 6 | -1.87336 |
| UVC_HIGH_D7_DN | 32 | 4 | -1.87792 |
| INSULIN_ADIP_INSENS_DN | 17 | 3 | -1.91933 |
| ET743_HELA_UP | 56 | 9 | -1.93438 |
| VERNELL_PRB_CLSTR1 | 69 | 4 | -2.02792 |
| EDG1PATHWAY | 26 | 3 | -2.12141 |
| ASTON_DEPRESSION_UP | 46 | 6 | -2.15505 |
| CHEMICALPATHWAY | 22 | 3 | -2.16759 |
| HDACI_COLON_SUL24HRS_DN | 128 | 4 | -2.27792 |
| D4GDIPATHWAY | 13 | 3 | -2.30038 |
| TENEDINI_MEGAKARYOCYTIC_GENES | 55 | 8 | -2.30223 |
| HESS_HOXAANMEIS1_UP | 75 | 6 | -2.31018 |
| HESS_HOXAANMEIS1_DN | 75 | 6 | -2.31018 |
| ASTIER_FN_DIFF | 63 | 3 | -2.34657 |
| ASTIER_BCELL | 62 | 3 | -2.34657 |
| NI2_MOUSE_DN | 46 | 4 | -2.64292 |
| HDACI_COLON_TSA_DN | 64 | 5 | -2.73127 |
| UVC_HIGH_D6_DN | 31 | 5 | -2.7581 |
| ROSS_CBF | 83 | 8 | -2.76892 |
| ROSS_CBF_LEUKEMIA | 75 | 8 | -2.76892 |
| ADIP_VS_FIBRO_UP | 35 | 7 | -2.7819 |
| GCRPATHWAY | 19 | 3 | -2.94702 |
| ROSS_CBF_MYH | 57 | 4 | -2.99292 |
| GALE_FLT3ANDAPL_UP | 61 | 6 | -3.18383 |
| BECKER_TAMOXIFEN_RESISTANT_DN | 53 | 4 | -3.18792 |
| LOTEM_LEUKEMIA_UP | 26 | 4 | -3.24792 |
| AT1RPATHWAY | 34 | 3 | -3.25301 |
| ROSS_AML1_ETO | 87 | 4 | -3.34292 |
| LEE_MYC_E2F1_DN | 66 | 3 | -3.39158 |
| HSC_LTHSC_ADULT | 370 | 16 | -3.72334 |
| OLDAGE_DN | 47 | 5 | -4.3949 |
| ASTON_OLIGODENDROGLIA_MYELINATION_SUBSET | 17 | 3 | -5.60283 |
| HG_PROGERIA_DN | 25 | 4 | -6.76292 |
