# Supplementary material for: Multiple Oxygen Tension Environments Reveal Diverse Patterns of Transcriptional Regulation in Primary Astrocytes
Source: PLoS One. 2011 Jun 27;6(6):e21638. doi: 10.1371/journal.pone.0021638 (PMC3124552; doi:10.1371/journal.pone.0021638)
Supplement: Table S10 — Significantly populated PAGE gene collections created with transcripts responding to 9% O2 tension compared to control 20% O2 condition. Significantly-regulated genes were used to populate the specified MSigDB collections. ‘# genes in collection’ describes the total gene count of the specific MSigDB collection and the ‘# exp genes in collection’ describes the number of genes from the input experimental set that were able to significantly populate the specific MSigDB collection. The Z score is calculated based upon the cumulative z ratios of the respective genes from the experimental dataset that populated the specific MSigDB collection. (DOC) [file pone.0021638.s016.doc]

**Table S10. Significantly populated PAGE gene collections created with transcripts responding to 9% O_2_ tension compared to control 20% O_2_ condition.** Significantly-regulated genes were used to populate the specified MSigDB collections. ‘# genes in collection’ describes the total gene count of the specific MSigDB collection and the ‘# exp genes in collection’ describes the number of genes from the input experimental set that were able to significantly populate the specific MSigDB collection. The Z score is calculated based upon the cumulative z ratios of the respective genes from the experimental dataset that populated the specific MSigDB collection

| **MSigDB collection** | **# genes in collection** | **# exp genes in collection** | **Z Score** |
| --- | --- | --- | --- |
| GLYCOLYSISPATHWAY | 10 | 6 | 7.368747 |
| PENG_LEUCINE_DN | 180 | 22 | 7.284812 |
| GLYCOLYSIS | 56 | 11 | 7.012972 |
| GLUCONEOGENESIS | 56 | 11 | 7.012972 |
| HIF1_TARGETS | 36 | 15 | 6.609679 |
| GLYCOLYSIS_AND_GLUCONEOGENESIS | 44 | 12 | 6.4517 |
| CARBON_FIXATION | 23 | 4 | 5.42989 |
| PGC | 425 | 42 | 5.42958 |
| CORDERO_KRAS_KD_VS_CONTROL_UP | 84 | 20 | 5.361846 |
| ZUCCHI_EPITHELIAL_UP | 50 | 13 | 5.299963 |
| MENSSEN_MYC_UP | 35 | 10 | 5.227072 |
| PENG_RAPAMYCIN_DN | 229 | 25 | 5.181726 |
| CAMPTOTHECIN_PROBCELL_DN | 31 | 5 | 5.165195 |
| LEI_MYB_REGULATED_GENES | 339 | 47 | 4.788372 |
| METHOTREXATE_PROBCELL_DN | 14 | 3 | 4.757271 |
| CANCERDRUGS_PROBCELL_DN | 15 | 3 | 4.757271 |
| RIBOSOMAL_PROTEINS | 203 | 49 | 4.679617 |
| NADLER_OBESITY_DN | 39 | 8 | 4.610181 |
| ADIPOGENESIS_HMSC_CLASS8_DN | 32 | 8 | 4.574826 |
| SHIPP_FL_VS_DLBCL_DN | 40 | 11 | 4.492337 |
| MTA3PATHWAY | 16 | 4 | 4.43489 |
| HDACI_COLON_CUR24HRS_UP | 37 | 9 | 3.979836 |
| MYOD_NIH3T3_UP | 82 | 6 | 3.959874 |
| HALMOS_CEBP_DN | 46 | 5 | 3.953246 |
| HSP27PATHWAY | 16 | 3 | 3.902793 |
| P53_SIGNALING | 101 | 12 | 3.882491 |
| ROME_INSULIN_2F_UP | 235 | 29 | 3.865882 |
| RADIATION_SENSITIVITY | 27 | 3 | 3.862378 |
| FRUCTOSE_AND_MANNOSE_METABOLISM | 25 | 4 | 3.83989 |
| ARGININE_AND_PROLINE_METABOLISM | 47 | 5 | 3.787777 |
| STEMCELL_NEURAL_UP | 1838 | 144 | 3.780176 |
| FERNANDEZ_MYC_TARGETS | 186 | 39 | 3.773878 |
| TARTE_MATURE_PC | 425 | 57 | 3.728145 |
| SERUM_FIBROBLAST_CORE_DN | 197 | 20 | 3.680323 |
| GLYCOGEN | 21 | 4 | 3.67989 |
| ZELLER_MYC_UP | 27 | 9 | 3.673169 |
| CROONQUIST_IL6_STROMA_UP | 40 | 5 | 3.64914 |
| LEE_MYC_DN | 67 | 5 | 3.622308 |
| BYSTRYKH_HSC_BRAIN_TRANS_GLOCUS | 218 | 11 | 3.563682 |
| CHANG_SERUM_RESPONSE_DN | 194 | 20 | 3.508146 |
| DER_IFNG_UP | 64 | 7 | 3.480908 |
| HDACI_COLON_CUR_UP | 108 | 18 | 3.464591 |
| METASTASIS_ADENOCARC_UP | 14 | 3 | 3.394725 |
| BLEO_MOUSE_LYMPH_HIGH_24HRS_DN | 34 | 5 | 3.336091 |
| PASSERINI_EM | 42 | 8 | 3.31264 |
| LIZUKA_L0_SM_L1 | 21 | 4 | 3.24989 |
| TUMOR_SUPRESSOR | 26 | 3 | 3.227293 |
| METASTASIS_ADENOCARC_DN | 34 | 4 | 3.21989 |
| TPA_SENS_MIDDLE_UP | 67 | 13 | 3.219837 |
| AGED_MOUSE_HYPOTH_UP | 44 | 5 | 3.192983 |
| MYC_TARGETS | 42 | 12 | 3.166576 |
| NADLER_OBESITY_UP | 59 | 13 | 3.111671 |
| UVB_NHEK1_UP | 177 | 22 | 3.110342 |
| HYPOXIA_RCC_UP | 104 | 7 | 3.106723 |
| INSULIN_SIGNALING | 103 | 12 | 3.010692 |
| BASSO_GERMINAL_CENTER_CD40_UP | 108 | 7 | 2.978215 |
| CALCINEURIN_NF_AT_SIGNALING | 100 | 11 | 2.942569 |
| IFN_GAMMA_UP | 39 | 4 | 2.92989 |
| BREASTCA_THREE_CLASSES | 42 | 5 | 2.848628 |
| REN_E2F1_TARGETS | 50 | 6 | 2.759624 |
| AGED_MOUSE_CORTEX_UP | 31 | 11 | 2.743572 |
| ET743_SARCOMA_24HRS_DN | 110 | 7 | 2.728759 |
| DER_IFNA_UP | 67 | 4 | 2.66489 |
| PENTOSE_PHOSPHATE_PATHWAY | 27 | 3 | 2.621075 |
| POMEROY_DESMOPLASIC_VS_CLASSIC_MD_UP | 49 | 11 | 2.592816 |
| HOFMANN_MDS_CD34_LOW_AND_HIGH_RISK | 47 | 3 | 2.592208 |
| BCRABL_HL60_CDNA_UP | 14 | 4 | 2.58989 |
| AKTPATHWAY | 19 | 3 | 2.586434 |
| VERNELL_PRB_CLSTR2 | 23 | 4 | 2.50489 |
| P38MAPKPATHWAY | 40 | 4 | 2.48489 |
| HTERT_UP | 69 | 5 | 2.481913 |
| LIZUKA_G2_GR_G3 | 28 | 3 | 2.465191 |
| TPA_RESIST_EARLY_UP | 30 | 4 | 2.45989 |
| MRNA_SPLICING | 58 | 3 | 2.436323 |
| TPA_RESIST_MIDDLE_UP | 49 | 5 | 2.428247 |
| SIG_CHEMOTAXIS | 45 | 4 | 2.39489 |
| ZHAN_MMPC_LATEVS | 47 | 6 | 2.355458 |
| HIPPOCAMPUS_DEVELOPMENT_PRENATAL | 35 | 8 | 2.290871 |
| PTDINSPATHWAY | 23 | 4 | 2.28989 |
| HDACI_COLON_CUR2HRS_UP | 29 | 7 | 2.275201 |
| ZHAN_TONSIL_BONEMARROW | 50 | 5 | 2.253834 |
| GENOTOXINS_24HRS_DISCR | 40 | 6 | 2.237066 |
| TSA_CD4_UP | 28 | 9 | 2.199836 |
| GNATENKO_PLATELET_UP | 48 | 6 | 2.167664 |
| GNATENKO_PLATELET | 48 | 6 | 2.167664 |
| FLECHNER_KIDNEY_TRANSPLANT_REJECTION_PBL_DN | 51 | 4 | 2.15989 |
| CHESLER_BRAIN_ONLY_SUBSET | 26 | 4 | 2.08489 |
| P21_ANY_DN | 36 | 3 | 2.078366 |
| EIF4PATHWAY | 24 | 4 | 2.06489 |
| TRANSLATION_FACTORS | 56 | 5 | 2.007867 |
| UVB_NHEK1_C2 | 22 | 5 | 1.998922 |
| SIG_REGULATION_OF_THE_ACTIN_CYTOSKELETON_BY_RHO_GTPASES | 35 | 5 | 1.963145 |
| RHOPATHWAY | 31 | 4 | 1.81989 |
| GCRPATHWAY | 19 | 3 | 1.812785 |
| MARCINIAK_CHOP_DIFF | 26 | 3 | 1.795464 |
| PLATELET_EXPRESSED | 34 | 3 | 1.726182 |
| INOS_ALL_DN | 77 | 10 | 1.666347 |
| WALLACE_JAK2_DIFF | 31 | 3 | 1.570298 |
| HDACPATHWAY | 30 | 5 | 1.158161 |
| CIS_RESIST_LUNG_DN | 11 | 6 | 1.151126 |
| HDACI_COLON_SUL16HRS_DN | 72 | 3 | 1.039136 |
| TNFR1PATHWAY | 29 | 4 | -0.96511 |
| UVC_XPCS_8HR_UP | 59 | 3 | -0.97004 |
| ADIP_VS_FIBRO_UP | 35 | 7 | -1.84461 |
| CMV_HCMV_TIMECOURSE_18HRS_UP | 74 | 3 | -1.98041 |
| BCRABL_HL60_CDNA_DN | 28 | 3 | -2.18825 |
| EDG1PATHWAY | 26 | 3 | -2.47115 |
| ROSS_CBF_MYH | 57 | 4 | -2.61011 |
| GALE_FLT3ANDAPL_UP | 61 | 6 | -2.61292 |
| HDACI_COLON_SUL24HRS_DN | 128 | 4 | -2.75011 |
| HDACI_COLON_TSA_DN | 64 | 5 | -3.09931 |
| VEGF_HUVEC_UP | 15 | 4 | -3.15011 |
| UVC_HIGH_D6_DN | 31 | 5 | -3.29161 |
| LINDSTEDT_DEND_DN | 66 | 7 | -3.31867 |
| ET743_HELA_UP | 56 | 9 | -3.4235 |
| UVB_NHEK3_C2 | 43 | 6 | -3.61313 |
| ASTON_DEPRESSION_UP | 46 | 6 | -3.77643 |
| ASTON_OLIGODENDROGLIA_MYELINATION_SUBSET | 17 | 3 | -3.86834 |
| CREB_BRAIN_8WKS_DN | 49 | 6 | -4.02138 |
| SMITH_HTERT_UP | 117 | 9 | -4.4835 |
| HSC_LTHSC_SHARED | 274 | 14 | -5.71158 |
| HSC_LTHSC_FETAL | 274 | 14 | -5.71158 |
| HSC_LTHSC_ADULT | 370 | 16 | -5.97272 |
| OLDAGE_DN | 47 | 5 | -6.76646 |
| HG_PROGERIA_DN | 25 | 4 | -7.94011 |
