# Supplementary material for: Multiple Oxygen Tension Environments Reveal Diverse Patterns of Transcriptional Regulation in Primary Astrocytes
Source: PLoS One. 2011 Jun 27;6(6):e21638. doi: 10.1371/journal.pone.0021638 (PMC3124552; doi:10.1371/journal.pone.0021638)
Supplement: Table S11 — Venn diagram analysis output for significant PAGE gene collection population between 1, 4, 9% O2 tensions versus 20% O2. PAGE gene collections populated by significantly regulated genes with a positive Z score are denoted in normal text while PAGE gene collections populated by significantly regulated genes with a negative Z score are denoted in italics. The PAGE collections are organized into the specific Venn diagram subsets, A–G, depicted in Figure 4. (DOC) [file pone.0021638.s017.doc]

**Table S11. Venn diagram analysis output for significant PAGE gene collection population between 1, 4, 9% O_2_ tensions versus 20% O_2_.** PAGE gene collections populated by significantly regulated genes with a positive Z score are denoted in normal text while PAGE gene collections populated by significantly regulated genes with a negative Z score are denoted in italics. The PAGE collections are organized into the specific Venn diagram subsets, A-G, depicted in Figure 4.

| **A** | **D** |
| --- | --- |
| MENSE_HYPOXIA_UP | HIPPOCAMPUS_DEVELOPMENT_POSTNATAL |
| HYPOXIA_REVIEW | NING_COPD_UP |
| HYPOXIA_REG_UP | GILDEA_BLADDER_UP |
| LVAD_HEARTFAILURE_UP | EGF_HDMEC_UP |
| GENOTOXINS_ALL_24HRS_REG | JISON_SICKLECELL_DIFF |
| HYPERTROPHY_MODEL | FLOTHO_CASP8AP2_MRD_DIFF |
| BRCA_PROGNOSIS_NEG | UVB_NHEK1_C1 |
| ET743_SARCOMA_UP | DRUG_RESISTANCE_AND_METABOLISM |
| TSA_PANC50_UP | MA_ATRA_EMP_UP |
| CMV_HCMV_TIMECOURSE_8HRS_UP | DNA_REPLICATION_REACTOME |
| HYPOXIA_FIBRO_UP | UVB_NHEK2_UP |
| ELONGINA_KO_DN | IFN_BETA_GLIOMA_DN |
| STRESS_ARSENIC_SPECIFIC_UP | ST_INTEGRIN_SIGNALING_PATHWAY |
| WALKER_MM_SNP_DIFF | *LOTEM_LEUKEMIA_UP* |
| ET743_SARCOMA_6HRS_UP | *CHEMICALPATHWAY* |
| ZUCCHI_EPITHELIAL_DN | *ROSS_AML1_ETO* |
| O6BG_RESIST_MEDULLOBLASTOMA_UP | *VERNELL_PRB_CLSTR1* |
| HUMAN_CD34_ENRICHED_TRANSCRIPTION_FACTORS | *D4GDIPATHWAY* |
| ET743_SARCOMA_24HRS_UP | *BECKER_TAMOXIFEN_RESISTANT_DN* |
| ET743_SARCOMA_72HRS_UP | *AT1RPATHWAY* |
| CMV_HCMV_6HRS_DN | *NI2_MOUSE_DN* |
| KNUDSEN_PMNS_DN | *FATTY_ACID_METABOLISM* |
| BREAST_CANCER_ESTROGEN_SIGNALING |  |
| NGUYEN_KERATO_DN | **E** |
| SANA_IFNG_ENDOTHELIAL_DN | FRUCTOSE_AND_MANNOSE_METABOLISM |
| XU_ATRA_PLUSNSC_DN | UVB_NHEK1_UP |
| UVB_NHEK3_C2 | MENSSEN_MYC_UP |
| IFN_BETA_UP | PENTOSE_PHOSPHATE_PATHWAY |
| SIG_BCR_SIGNALING_PATHWAY | HDACI_COLON_CUR24HRS_UP |
| *IL6_FIBRO_UP* | AGED_MOUSE_CORTEX_UP |
| *CORTEX_ENRICHMENT_LATE_UP* | P38MAPKPATHWAY |
| *CIS_XPC_UP* | UVB_NHEK1_C2 |
| *UVC_LOW_C2_DN* | CALCINEURIN_NF_AT_SIGNALING |
| *SANSOM_APC_4_DN* | BASSO_GERMINAL_CENTER_CD40_UP |
| *CASPASEPATHWAY* | PTDINSPATHWAY |
| *HDACPATHWAY* | BREASTCA_THREE_CLASSES |
| *GUO_HEX_UP* | AGED_MOUSE_HYPOTH_UP |
| *ST_GA12_PATHWAY* | *TNFR1PATHWAY* |
| *CHESLER_BRAIN_HIGHEST_VARIANCE_GENES* | *VEGF_HUVEC_UP* |
| *LDLPATHWAY* | *HSC_LTHSC_FETAL* |
| *H2O2_CSBDIFF_C1* | *HSC_LTHSC_SHARED* |
| *CCR5PATHWAY* |  |
| *DNMT1_KO_DN* | **F** |
| *GH_GHRHR_KO_24HRS_DN* | NADLER_OBESITY_UP |
| *POD1_KO_MOST_UP* | ZHAN_TONSIL_BONEMARROW |
| *STAEGE_EFTS_UP* | ZHAN_MMPC_LATEVS |
| *MMS_MOUSE_LYMPH_HIGH_4HRS_UP* | PASSERINI_EM |
| *ALCALAY_AML_NPMC_DN* | BYSTRYKH_HSC_BRAIN_TRANS_GLOCUS |
| *CMV_HCMV_TIMECOURSE_20HRS_UP* | PGC |
| *SERUM_FIBROBLAST_CELLCYCLE* | LIZUKA_L0_SM_L1 |
| *PRMT5_KD_UP* | RHOPATHWAY |
| *CPR_LOW_LIVER_UP* | HTERT_UP |
| *ASTON_DEPRESSION_DN* | GNATENKO_PLATELET_UP |
| *GH_AUTOCRINE_UP* | GNATENKO_PLATELET |
| *CALCIUM_REGULATION_IN_CARDIAC_CELLS* | METASTASIS_ADENOCARC_DN |
| *HASLINGER_B_CLL_11Q23* | TPA_RESIST_EARLY_UP |
| *BIOSYNTHESIS_OF_STEROIDS* | SERUM_FIBROBLAST_CORE_DN |
| *CHOLESTEROL_BIOSYNTHESIS* | CHESLER_BRAIN_ONLY_SUBSET |
| *GAY_YY1_DN* | SIG_REGULATION_OF_THE_ACTIN_CYTOSKELETON_BY_RHO_GTPASES |
| *REOVIRUS_HEK293_DN* | CHANG_SERUM_RESPONSE_DN |
|  | HALMOS_CEBP_DN |
| **B** | TPA_RESIST_MIDDLE_UP |
| AGEING_KIDNEY_UP | MYOD_NIH3T3_UP |
| CHEN_LUNG_SURVIVAL | RADIATION_SENSITIVITY |
| TNFALPHA_4HRS_UP | PENG_LEUCINE_DN |
| NAKAJIMA_MCS_UP | NADLER_OBESITY_DN |
| TGFBETA_EARLY_UP | DER_IFNG_UP |
| ZHAN_MM_CD138_MS_VS_REST | *SMITH_HTERT_UP* |
| TGFBETA_ALL_UP | *ET743_HELA_UP* |
| IL6_FIBRO_UP | *ASTON_DEPRESSION_UP* |
| VEGF_MMMEC_6HRS_UP | *UVC_HIGH_D6_DN* |
| BRCA1_SW480_UP | *ADIP_VS_FIBRO_UP* |
| BRCA2_BRCA1_UP | *ROSS_CBF_MYH* |
| FSH_OVARY_MCV152_DN | *OLDAGE_DN* |
| ACTINYPATHWAY | *ASTON_OLIGODENDROGLIA_MYELINATION_SUBSET* |
| SALMONELLAPATHWAY | *HG_PROGERIA_DN* |
| CALRES_MOUSE_UP |  |
| ALZHEIMERS_INCIPIENT_DN | **G** |
| TGFBETA_C2_UP | RIBOSOMAL_PROTEINS |
| DFOSB_BRAIN_2WKS_UP | GLYCOLYSIS_AND_GLUCONEOGENESIS |
| YAGI_AML_PROG_ASSOC | GLYCOLYSIS |
| MATRIX_METALLOPROTEINASES | GLUCONEOGENESIS |
| YAO_P4_KO_VS_WT_DN | HIF1_TARGETS |
| PROTEASOMEPATHWAY | ROME_INSULIN_2F_UP |
| DAVIES_N | GLYCOLYSISPATHWAY |
| *ZHAN_MM_MOLECULAR_CLASSI_DN* | CARBON_FIXATION |
| *ET743_SARCOMA_24HRS_UP* | MTA3PATHWAY |
| *CMV_HCMV_TIMECOURSE_4HRS_DN* | P53_SIGNALING |
| *DAC_FIBRO_DN* | INSULIN_SIGNALING |
| *UVC_HIGH_D7_DN* | HDACI_COLON_CUR_UP |
| *INSULIN_ADIP_INSENS_DN* | ZUCCHI_EPITHELIAL_UP |
| *TENEDINI_MEGAKARYOCYTIC_GENES* | MYC_TARGETS |
| *HESS_HOXAANMEIS1_UP* | TUMOR_SUPRESSOR |
| *HESS_HOXAANMEIS1_DN* | CORDERO_KRAS_KD_VS_CONTROL_UP |
| *ASTIER_FN_DIFF* | FLECHNER_KIDNEY_TRANSPLANT_REJECTION_PBL_DN |
| *ASTIER_BCELL* | MARCINIAK_CHOP_DIFF |
| *ROSS_CBF* | HYPOXIA_RCC_UP |
| *ROSS_CBF_LEUKEMIA* | POMEROY_DESMOPLASIC_VS_CLASSIC_MD_UP |
| *GCRPATHWAY* | HDACI_COLON_CUR2HRS_UP |
| *LEE_MYC_E2F1_DN* | VERNELL_PRB_CLSTR2 |
|  | ZELLER_MYC_UP |
| **C** | HIPPOCAMPUS_DEVELOPMENT_PRENATAL |
| PENG_RAPAMYCIN_DN | FERNANDEZ_MYC_TARGETS |
| CAMPTOTHECIN_PROBCELL_DN | CIS_RESIST_LUNG_DN |
| LEI_MYB_REGULATED_GENES | METASTASIS_ADENOCARC_UP |
| METHOTREXATE_PROBCELL_DN | CROONQUIST_IL6_STROMA_UP |
| CANCERDRUGS_PROBCELL_DN | TRANSLATION_FACTORS |
| ADIPOGENESIS_HMSC_CLASS8_DN | SIG_CHEMOTAXIS |
| SHIPP_FL_VS_DLBCL_DN | *HDACI_COLON_TSA_DN* |
| HSP27PATHWAY | *GALE_FLT3ANDAPL_UP* |
| ARGININE_AND_PROLINE_METABOLISM | *HDACI_COLON_SUL24HRS_DN* |
| STEMCELL_NEURAL_UP | *EDG1PATHWAY* |
| TARTE_MATURE_PC | *HSC_LTHSC_ADULT* |
| GLYCOGEN |  |
| LEE_MYC_DN |  |
| BLEO_MOUSE_LYMPH_HIGH_24HRS_DN |  |
| TPA_SENS_MIDDLE_UP |  |
| IFN_GAMMA_UP |  |
| REN_E2F1_TARGETS |  |
| ET743_SARCOMA_24HRS_DN |  |
| DER_IFNA_UP |  |
| HOFMANN_MDS_CD34_LOW_AND_HIGH_RISK |  |
| BCRABL_HL60_CDNA_UP |  |
| AKTPATHWAY |  |
| LIZUKA_G2_GR_G3 |  |
| MRNA_SPLICING |  |
| GENOTOXINS_24HRS_DISCR |  |
| TSA_CD4_UP |  |
| P21_ANY_DN |  |
| EIF4PATHWAY |  |
| GCRPATHWAY |  |
| PLATELET_EXPRESSED |  |
| INOS_ALL_DN |  |
| WALLACE_JAK2_DIFF |  |
| HDACPATHWAY |  |
| HDACI_COLON_SUL16HRS_DN |  |
| *UVC_XPCS_8HR_UP* |  |
| *CMV_HCMV_TIMECOURSE_18HRS_UP* |  |
| *BCRABL_HL60_CDNA_DN* |  |
| *LINDSTEDT_DEND_DN* |  |
| *UVB_NHEK3_C2* |  |
| *CREB_BRAIN_8WKS_DN* |  |
