# Supplementary material for: Multiple Oxygen Tension Environments Reveal Diverse Patterns of Transcriptional Regulation in Primary Astrocytes
Source: PLoS One. 2011 Jun 27;6(6):e21638. doi: 10.1371/journal.pone.0021638 (PMC3124552; doi:10.1371/journal.pone.0021638)
Supplement: Table S12 — K-means clusters for 1, 4, 9% O2 tension versus 20% control O2 tension. The respective z ratios (z-r) for the specific oxygen tension versus 20% O2 for each transcript are displayed alongside the k-means generated cluster number. (DOC) [file pone.0021638.s018.doc]

**Table S12. *K-means* clusters for 1, 4, 9% O_2_ tension versus 20% control O_2_ tension.** The respective z ratios (z-r) for the specific oxygen tension versus 20% O_2_ for each transcript are displayed alongside the *k-means* generated cluster number.

| **Gene Symbol** | **Gene Definition** | **Cluster**  **number** | **1 vs. 20% (z-r)** | **4 vs. 20% (z-r)** | **9 vs. 20% (z-r)** |
| --- | --- | --- | --- | --- | --- |
| Txnip | Rattus norvegicus upregulated by 1,25-dihydroxyvitamin D-3 | ***1*** | -6.69 | -0.44 | -11.08 |
|  |  |  |  |  |  |
| Ptgds | Rattus norvegicus prostaglandin D2 synthase | ***3*** | -6.25 | -7.17 | 1.16 |
|  |  |  |  |  |  |
| Gjb2 | Rattus norvegicus gap junction membrane channel protein beta 2 | ***32*** | -4.43 | -5.43 | 0.17 |
| Bmp7 | Rattus norvegicus bone morphogenetic protein 7 | ***32*** | -2.49 | -4.77 | 0.68 |
|  |  |  |  |  |  |
| Atp1a2 | Rattus norvegicus ATPase, Na+/K+ transporting, alpha 2 polypeptide | ***8*** | -4.53 | -4.23 | -2.79 |
| LOC294789 | Rattus norvegicus similar to Hypothetical protein FLJ25422 | ***8*** | -3.57 | -4.41 | -3.02 |
| Cldn11 | Rattus norvegicus claudin 11 | ***8*** | -4.11 | -3.56 | -2.04 |
| Enpp2 | Rattus norvegicus ectonucleotide pyrophosphatase/phosphodiesterase 2 | ***8*** | -4.53 | -5.52 | -3.83 |
|  |  |  |  |  |  |
| Mycl1 | Rattus norvegicus v-myc myelocytomatosis viral oncogene homolog 1, lung carcinoma derived | ***27*** | -1.26 | -4.68 | -3.05 |
| Esm1 | Rattus norvegicus endothelial cell-specific molecule 1 | ***27*** | -0.95 | -4.13 | -3.17 |
| Sostdc1 | Rattus norvegicus uterine sensitization-associated gene 1 protein | ***27*** | -0.56 | -3.9 | -3.9 |
| LOC365476 | Rattus norvegicus similar to chromosome 10 open reading frame 79 | ***27*** | -0.94 | -5.54 | -2.91 |
| LOC360747 | Rattus norvegicus similar to axoneme central apparatus protein | ***27*** | -0.24 | -4.66 | -1.54 |
| OSP94 | Rattus norvegicus osmotic stress protein 94 kDa | ***27*** | -0.15 | -4.24 | -1.89 |
|  |  |  |  |  |  |
| Ptgs2 | Rattus norvegicus prostaglandin-endoperoxide synthase 2 | ***15*** | 0.15 | -6.05 | -6.44 |
|  |  |  |  |  |  |
| Dhcr7 | Rattus norvegicus 7-dehydrocholesterol reductase | ***4*** | -4.33 | -1.1 | -1.7 |
| MGC94018 | Rattus norvegicus glycosyltransferase AD-017 | ***4*** | -3.21 | -2.15 | -2.43 |
| Ttyh1 | Rattus norvegicus tweety homolog 1 | ***4*** | -2.95 | -1.74 | -2.03 |
| Podxl | Rattus norvegicus podocalyxin-like | ***4*** | -2.75 | -1.52 | -1.93 |
| Pdgfra | Rattus norvegicus platelet derived growth factor receptor, alpha polypeptide | ***4*** | -3.2 | -1.15 | -2.78 |
| Btg2 | Rattus norvegicus B-cell translocation gene 2, anti-proliferative | ***4*** | -2.83 | -1.12 | -2.7 |
| Tnfrsf1a | Rattus norvegicus tumor necrosis factor receptor superfamily, member 1a | ***4*** | -3.27 | -0.58 | -1.13 |
| Kazald1 | Rattus norvegicus Kazal-type serine protease inhibitor domain 1 | ***4*** | -3.17 | -0.66 | -0.92 |
| Unc50 | Rattus norvegicus unc-50 homolog | ***4*** | -2.84 | -0.6 | -1.03 |
| LOC306229 | Rattus norvegicus similar to RIKEN cDNA A630054L15; hypothetical protein MGC38041 | ***4*** | -3.26 | -0.78 | -1.96 |
| Hsd3b7 | Rattus norvegicus CCA2 protein | ***4*** | -2.67 | -0.7 | -1.83 |
| Aqp1 | Rattus norvegicus aquaporin 1 | ***4*** | -2.87 | -1.65 | -1.43 |
| Rbp1 | Rattus norvegicus retinol binding protein 1, cellular | ***4*** | -2.75 | -1.41 | -1.23 |
| Gusb | Rattus norvegicus glucuronidase, beta | ***4*** | -2.65 | -0.81 | -1.53 |
| Sphk1 | Rattus norvegicus sphingosine kinase 1 | ***4*** | -2.59 | -0.87 | -1.59 |
| LOC361213 | Rattus norvegicus similar to Serine palmitoyltransferase 1 | ***4*** | -2.53 | -0.78 | -1.33 |
| Lrrc5 | Rattus norvegicus leucine-rich repeat-containing 5 | ***4*** | -2.4 | -1.1 | -1.73 |
| Lamb1-1 | Rattus norvegicus laminin B1 subunit 1 | ***4*** | -2.18 | -1.13 | -1.53 |
| LOC300783 | Rattus norvegicus similar to Butyrate-induced transcript 1 | ***4*** | -2.62 | -1.16 | -2.08 |
| Hspa2 | Rattus norvegicus heat shock protein 2 | ***4*** | -2.29 | -0.8 | -2.11 |
| Lsm7 | Rattus norvegicus LSM7 homolog, U6 small nuclear RNA associated | ***4*** | -2.25 | -0.88 | -2.22 |
| Ppap2b | Rattus norvegicus ER transmembrane protein Dri 42 | ***4*** | -2.16 | -0.89 | -2.14 |
| Ltap | Rattus norvegicus loop tail associated protein | ***4*** | -2.24 | -1.11 | -2 |
| Slc1a5 | Rattus norvegicus sodium-dependent neutral amino acid transporter ASCT2 | ***4*** | -2.13 | -0.64 | -2.41 |
| Col6a3 | Rattus norvegicus procollagen, type VI, alpha 3 | ***4*** | -2.06 | -0.74 | -1.72 |
| LOC498095 | Rattus norvegicus similar to RIKEN cDNA 0610012D17 | ***4*** | -1.99 | -0.55 | -1.58 |
| LOC367903 | Rattus norvegicus similar to armadillo repeat protein ALEX2 | ***4*** | -1.73 | -0.65 | -1.9 |
| Polr2b | Rattus norvegicus polymerase | ***4*** | -1.66 | -0.78 | -1.67 |
| LOC309307 | Rattus norvegicus similar to KIAA2026 protein | ***4*** | -1.49 | -0.79 | -1.82 |
| Pou3f3 | Rattus norvegicus POU domain, class 3, transcription factor 3 | ***4*** | -1.6 | -0.7 | -1.49 |
| RGD1311463 | Rattus norvegicus similar to RIKEN cDNA 2700007P21 | ***4*** | -1.56 | -0.68 | -1.5 |
| LOC500042 | Rattus norvegicus similar to RIKEN cDNA 2610101N10 | ***4*** | -1.44 | -0.8 | -1.53 |
| Zfp297b | Rattus norvegicus zinc finger protein 297B | ***4*** | -1.58 | -0.84 | -1.27 |
| Waspip | Rattus norvegicus Wiskott-Aldrich syndrome protein interacting protein | ***4*** | -1.4 | -0.92 | -1.6 |
| RGD1309382 | Rattus norvegicus similar to RIKEN cDNA C730048E16 | ***4*** | -1.27 | -0.87 | -1.61 |
| LOC293844 | Rattus norvegicus similar to UCH37-interacting protein 1 | ***4*** | -1.8 | -0.8 | -2.33 |
| LOC362317 | Rattus norvegicus similar to krev interaction trapped-1A | ***4*** | -1.64 | -0.73 | -2.24 |
| Sec5l1 | Rattus norvegicus Rsec5 protein | ***4*** | -1.75 | -0.93 | -1.93 |
| LOC503190 | Rattus norvegicus similar to zinc finger protein 426 | ***4*** | -1.59 | -0.88 | -2.11 |
| Cbr1 | Rattus norvegicus carbonyl reductase 1 | ***4*** | -1.26 | -0.84 | -2.01 |
| LOC309816 | Rattus norvegicus similar to Laminin alpha-4 chain precursor | ***4*** | -1.17 | -0.67 | -2.07 |
| Nr2f1 | Rattus norvegicus nuclear receptor subfamily 2, group F, member 1 | ***4*** | -2.24 | -1.53 | -1.66 |
| MGC72974 | Rattus norvegicus Unknown | ***4*** | -2.17 | -1.5 | -1.63 |
| LOC315804 | Rattus norvegicus similar to hypothetical protein FLJ12994 | ***4*** | -2.15 | -1.86 | -1.73 |
| LOC313974 | Rattus norvegicus similar to Tribbles homolog 2 | ***4*** | -1.9 | -1.58 | -2.19 |
| Ywhab | Rattus norvegicus tyrosine 3-monooxygenase/tryptophan 5-monooxygenase activation protein, beta polypeptide | ***4*** | -1.79 | -1.49 | -2.07 |
| Nid2 | Rattus norvegicus nidogen 2 | ***4*** | -1.63 | -1.87 | -1.99 |
| Zfp462 | Rattus norvegicus zinc finger protein 462 | ***4*** | -1.71 | -1.14 | -2.2 |
| Adamts9 | Rattus norvegicus a disintegrin-like and metalloprotease | ***4*** | -1.58 | -1.26 | -2.18 |
| Slc25a29 | Rattus norvegicus solute carrier family 25 | ***4*** | -1.48 | -1.52 | -2.24 |
| Aif1 | Rattus norvegicus allograft inflammatory factor 1 | ***4*** | -1.41 | -1.56 | -2.27 |
| Dpm1 | Rattus norvegicus dolichol-phosphate | ***4*** | -1.43 | -1.36 | -1.95 |
| Lkap | Rattus norvegicus limkain b1 | ***4*** | -1.27 | -1.33 | -1.99 |
| RGD1307010 | Rattus norvegicus similar to RIKEN cDNA 2700085E05 | ***4*** | -1.71 | -1.21 | -1.75 |
| Rnf7 | Rattus norvegicus ring finger protein 7 | ***4*** | -1.59 | -1.37 | -1.85 |
| Plekhb2 | Rattus norvegicus pleckstrin homology domain containing, family B | ***4*** | -1.65 | -1.51 | -1.73 |
| Nup107 | Rattus norvegicus nucleoporin 107 | ***4*** | -1.5 | -1.46 | -1.66 |
| Gap43 | Rattus norvegicus growth associated protein 43 | ***4*** | -2.35 | -2.44 | -1.33 |
| Serpinb1a | Rattus norvegicus serine | ***4*** | -2.23 | -2.26 | -1.52 |
| Cspg5 | Rattus norvegicus chondroitin sulfate proteoglycan 5 | ***4*** | -2.33 | -2.45 | -1 |
| LOC302863 | Rattus norvegicus similar to mKIAA0267 protein | ***4*** | -2.2 | -2.46 | -1.03 |
| LOC499593 | Rattus norvegicus similar to SOX2 protein | ***4*** | -2.3 | -2.03 | -1.11 |
| AF146738 | Rattus norvegicus testis specific protein | ***4*** | -2.19 | -2.09 | -1.21 |
| Tec | Rattus norvegicus tec protein tyrosine kinase | ***4*** | -1.88 | -2.56 | -1.58 |
| Gprasp1 | Rattus norvegicus G protein-coupled receptor associated sorting protein 1 | ***4*** | -1.74 | -2.57 | -1.43 |
| Gnpat | Rattus norvegicus glyceronephosphate O-acyltransferase | ***4*** | -1.75 | -2.15 | -1.57 |
| LOC294734 | Rattus norvegicus similar to RIKEN cDNA 1700034P14 | ***4*** | -1.57 | -2.42 | -1.43 |
| Thtpa | Rattus norvegicus thiamine triphosphatase | ***4*** | -2.11 | -1.38 | -1.38 |
| Abcd3 | Rattus norvegicus ATP-binding cassette, sub-family D | ***4*** | -2.07 | -1.51 | -1.19 |
| Ythdf1 | Rattus norvegicus YTH domain family 1 | ***4*** | -1.9 | -1.18 | -1.37 |
| LOC501521 | Rattus norvegicus similar to T-complex associated-testis-expressed 1-like | ***4*** | -1.68 | -1.07 | -1.34 |
| Wee1 | Rattus norvegicus wee 1 homolog | ***4*** | -1.89 | -1.2 | -1.19 |
| Klf7 | Rattus norvegicus Kruppel-like factor 7 | ***4*** | -1.86 | -1.33 | -1.19 |
| Abr | Rattus norvegicus active BCR-related gene | ***4*** | -1.57 | -1.29 | -1.4 |
| Sema6a | Rattus norvegicus sema domain, transmembrane domain | ***4*** | -1.52 | -1.39 | -1.19 |
| LOC302378 | Rattus norvegicus similar to G protein-coupled receptor 23 | ***4*** | -1.99 | -1.77 | -1 |
| LOC498751 | Rattus norvegicus similar to RP23-462P2.7 | ***4*** | -1.9 | -1.85 | -1.1 |
| LOC296758 | Rattus norvegicus similar to RIKEN cDNA 2810037C14 | ***4*** | -1.79 | -2 | -1.1 |
| LOC498564 | Rattus norvegicus similar to integrin, beta-like 1 | ***4*** | -1.69 | -1.88 | -1.23 |
| Tsn | Rattus norvegicus translin | ***4*** | -1.85 | -1.58 | -1.23 |
| Donson | Rattus norvegicus downstream neighbor of SON | ***4*** | -1.84 | -1.53 | -1.35 |
| Scp2 | Rattus norvegicus sterol carrier protein 2 | ***4*** | -1.65 | -1.62 | -1.36 |
| Lyplal1 | Rattus norvegicus lysophospholipase-like 1 | ***4*** | -1.63 | -1.51 | -1.3 |
| Stk16 | Rattus norvegicus serine/threonine kinase 16 | ***4*** | -1.71 | -1.67 | -1.19 |
| RGD1308373 | Rattus norvegicus similar to DKFZP566K1924 protein | ***4*** | -1.7 | -1.58 | -1.08 |
| LOC309081 | Rattus norvegicus similar to Dock1 protein | ***4*** | -1.62 | -1.64 | -1.08 |
| LOC499625 | Rattus norvegicus similar to Selenoprotein T precursor | ***4*** | -1.46 | -1.81 | -1.29 |
| Bphl | Rattus norvegicus biphenyl hydrolase-like | ***4*** | -1.34 | -1.82 | -1.16 |
| LOC299050 | Rattus norvegicus similar to 1110008L16Rik protein | ***4*** | -1.29 | -1.53 | -1.34 |
| Setdb1 | Rattus norvegicus SET domain, bifurcated 1 | ***4*** | -1.12 | -1.55 | -1.27 |
| RGD1306020 | Rattus norvegicus similar to aspartyl beta-hydroxylase; calsequestrin-binding protein; 3110001L23Rik | ***4*** | -1.23 | -1.42 | -1.54 |
| Dnase2 | Rattus norvegicus deoxyribonuclease II | ***4*** | -1.11 | -1.5 | -0.99 |
| Baalc | Rattus norvegicus brain and acute leukemia, cytoplasmic | ***4*** | -1.01 | -1.52 | -0.93 |
| Rkhd2 | Rattus norvegicus ring finger and KH domain containing 2 | ***4*** | -1.04 | -1.76 | -1.13 |
| RGD1311049 | Rattus norvegicus similar to RIKEN cDNA 4833417L20 | ***4*** | -1.01 | -1.73 | -0.99 |
| LOC294917 | Rattus norvegicus similar to Traf2 and NCK interacting kinase, splice variant 4 | ***4*** | -0.92 | -1.83 | -1.31 |
| Lr8 | Rattus norvegicus LR8 protein | ***4*** | -0.81 | -1.73 | -1.24 |
| Slc27a1 | Rattus norvegicus solute carrier family 27 | ***4*** | -1.52 | -2.14 | -1.86 |
| LOC500671 | Rattus norvegicus similar to chromosome 14 open reading frame 135 | ***4*** | -1.49 | -2.18 | -1.84 |
| LOC360733 | Rattus norvegicus similar to 5830458K16Rik protein | ***4*** | -1.3 | -2.32 | -1.86 |
| Rtn1 | Rattus norvegicus reticulon 1 | ***4*** | -1.41 | -2.34 | -2.13 |
| Edg2 | Rattus norvegicus endothelial differentiation, lysophosphatidic acid G-protein-coupled receptor, 2 | ***4*** | -1.29 | -2.51 | -1.66 |
| Sult1a1 | Rattus norvegicus sulfotransferase family 1A, phenol-preferring, member 1 | ***4*** | -1.09 | -2.42 | -1.58 |
| Ltbp4 | Rattus norvegicus latent transforming growth factor beta binding protein 4 | ***4*** | -1.05 | -2.66 | -1.79 |
| LOC499589 | Rattus norvegicus similar to hypothetical protein MGC27085 | ***4*** | -1.03 | -2.62 | -2.09 |
| Lpl | Rattus norvegicus lipoprotein lipase | ***4*** | -1.21 | -1.77 | -1.63 |
| LOC305035 | Rattus norvegicus similar to D1Ertd396e protein | ***4*** | -1 | -2.04 | -1.72 |
| Abhd3 | Rattus norvegicus abhydrolase domain containing 3 | ***4*** | -0.71 | -2.42 | -1.69 |
| Myh14 | Rattus norvegicus myosin, heavy polypeptide 14 | ***4*** | -0.59 | -2.17 | -1.61 |
| Vamp4 | Rattus norvegicus vesicle-associated membrane protein 4 | ***4*** | -0.49 | -2.08 | -1.43 |
| Tcfap2b | Rattus norvegicus transcription factor AP-2 beta | ***4*** | -3.02 | -2.64 | -3.31 |
| Cyp26b1 | Rattus norvegicus cytochrome P450, family 26, subfamily b, polypeptide 1 | ***4*** | -3 | -2.69 | -3.89 |
| Tnmd | Rattus norvegicus tenomodulin | ***4*** | -2.38 | -1.7 | -3.78 |
| Zfp36 | Rattus norvegicus zinc finger protein 36 | ***4*** | -2.52 | -0.28 | -3.44 |
| LOC316085 | Rattus norvegicus similar to 106 kDa O-GlcNAc transferase-interacting protein | ***4*** | -1.58 | -0.68 | -4.06 |
| Sfrs5 | Rattus norvegicus splicing factor, arginine/serine-rich 5 | ***4*** | -1.9 | -1.14 | -3.39 |
| Gstt2 | Rattus norvegicus glutathione S-transferase, theta 2 | ***4*** | -1.87 | -0.61 | -3.21 |
| Agrn | Rattus norvegicus agrin | ***4*** | -1.84 | -1.1 | -2.69 |
| Fbn2 | Rattus norvegicus fibrillin 2 | ***4*** | -1.8 | -1.25 | -2.81 |
|  |  |  |  |  |  |
| LOC499196 | Rattus norvegicus LOC499196 | ***29*** | -2.08 | -1.69 | -3.12 |
| Lgi4 | Rattus norvegicus leucine-rich repeat LGI family, member 4 | ***29*** | -2.04 | -1.71 | -3.2 |
| Sec24d | Rattus norvegicus SEC24 related gene family, member D | ***29*** | -2.07 | -1.63 | -2.87 |
| Cd38 | Rattus norvegicus CD38 antigen | ***29*** | -1.59 | -1.54 | -2.66 |
| Cotl1 | Rattus norvegicus coactosin-like 1 | ***29*** | -1.46 | -1.69 | -2.61 |
| P2rxl1 | Rattus norvegicus purinergic receptor P2X-like 1, orphan receptor | ***29*** | -1.44 | -1.75 | -2.51 |
| LOC304919 | Rattus norvegicus similar to RIKEN cDNA 5830468K18 | ***29*** | -1.31 | -1.78 | -2.66 |
| Trim39 | Rattus norvegicus tripartite motif protein 39 | ***29*** | -1.34 | -2.45 | -2.83 |
| Ppt | Rattus norvegicus palmitoyl-protein thioesterase | ***29*** | -1.31 | -2.6 | -2.76 |
| Chrdl1 | Rattus norvegicus kohjirin | ***29*** | -1.1 | -1.27 | -2.39 |
| LOC499798 | Rattus norvegicus similar to ADP-ribosylation-like factor 6-interacting protein 6 | ***29*** | -1.06 | -1.51 | -2.26 |
| LOC500865 | Rattus norvegicus similar to RIKEN cDNA 5730410E15 gene | ***29*** | -1.05 | -1.7 | -2.41 |
| LOC498931 | Rattus norvegicus similar to short coiled-coil protein | ***29*** | -1.02 | -1.53 | -2.44 |
| LOC307302 | Rattus norvegicus similar to hypothetical protein FLJ36090 | ***29*** | -0.96 | -1.43 | -2.05 |
| Lpd | Rattus norvegicus lipidosin | ***29*** | -0.89 | -1.46 | -2.06 |
| Polr2i | Rattus norvegicus polymerase | ***29*** | -0.85 | -1.59 | -2.13 |
| Lig1 | Rattus norvegicus ligase I, DNA, ATP-dependent | ***29*** | -0.81 | -1.67 | -2.17 |
| LOC500364 | Rattus norvegicus similar to Sspn protein | ***29*** | -0.79 | -1.09 | -2.65 |
| Sirt3 | Rattus norvegicus sirtuin 3 | ***29*** | -0.56 | -1.19 | -2.43 |
| Pex13 | Rattus norvegicus peroxisomal biogenesis factor 13 | ***29*** | -0.81 | -1.94 | -2.15 |
| Mbd1 | Rattus norvegicus methyl-CpG binding domain protein 1 | ***29*** | -0.77 | -1.98 | -2.03 |
| LOC306991 | Rattus norvegicus similar to Vps41 protein | ***29*** | -0.73 | -2.14 | -2.09 |
| Snrpa1 | Rattus norvegicus small nuclear ribonucleoprotein polypeptide A' | ***29*** | -0.47 | -1.7 | -2.28 |
| Psmd4 | Rattus norvegicus proteasome | ***29*** | -0.45 | -1.58 | -2.15 |
| Pik3c3 | Rattus norvegicus phosphoinositide-3-kinase, class 3 | ***29*** | -0.35 | -1.61 | -2.43 |
| LOC497766 | Rattus norvegicus hypothetical gene supported by NM_171983 | ***29*** | -0.41 | -1.6 | -2.93 |
| Hmgb2 | Rattus norvegicus high mobility group box 2 | ***29*** | 0.06 | -1.72 | -3.04 |
| F3 | Rattus norvegicus coagulation factor III | ***29*** | 0.23 | -1.89 | -3.05 |
| LOC499531 | Rattus norvegicus similar to LRRGT00176 | ***29*** | 0.23 | -1.39 | -2.94 |
| Phr1 | Rattus norvegicus pam, highwire, rpm 1 | ***29*** | 0.24 | -1.91 | -2.38 |
| Dscr1l1 | Rattus norvegicus Down syndrome critical region gene 1-like 1 | ***29*** | -1.11 | -1.18 | -2.08 |
| LOC500116 | Rattus norvegicus similar to RIKEN cDNA G430041M01 | ***29*** | -0.97 | -1.3 | -2.02 |
| LOC500002 | Rattus norvegicus similar to A-kinase anchor protein 9 | ***29*** | -0.94 | -1.28 | -2.16 |
| LOC297514 | Rattus norvegicus similar to mKIAA1757 protein | ***29*** | -0.98 | -1.09 | -1.93 |
| Arhe | Rattus norvegicus ras homolog gene family, member E | ***29*** | -0.78 | -1.13 | -1.84 |
| Slc25a25 | Rattus norvegicus solute carrier family 25 | ***29*** | -0.73 | -1.11 | -1.94 |
| Trappc4 | Rattus norvegicus trafficking protein particle complex 4 | ***29*** | -0.81 | -0.99 | -2.11 |
| LOC497834 | Rattus norvegicus hypothetical gene supported by NM_031053 | ***29*** | -1.04 | -1.13 | -1.54 |
| Ddx23 | Rattus norvegicus DEAD | ***29*** | -0.87 | -1.05 | -1.58 |
| LOC288515 | Rattus norvegicus similar to FLJ23471 protein | ***29*** | -0.65 | -0.96 | -1.5 |
| LOC306007 | Rattus norvegicus similar to 2610301G19Rik protein | ***29*** | -0.55 | -1.14 | -1.53 |
| Vcam1 | Rattus norvegicus vascular cell adhesion molecule 1 | ***29*** | -0.86 | -0.74 | -1.79 |
| Zcchc11 | Rattus norvegicus zinc finger, CCHC domain containing 11 | ***29*** | -0.84 | -0.9 | -1.9 |
| Atad1 | Rattus norvegicus ATPase family, AAA domain containing 1 | ***29*** | -0.78 | -0.81 | -1.86 |
| LOC362938 | Rattus norvegicus similar to PDZ-domain protein scribble | ***29*** | -0.81 | -0.78 | -1.97 |
| Pla2g6 | Rattus norvegicus phospholipase A2, group VI | ***29*** | -0.8 | -0.98 | -1.68 |
| LOC291750 | Rattus norvegicus similar to TRS85 homolog | ***29*** | -0.72 | -0.92 | -1.67 |
| Lancl1 | Rattus norvegicus lanC | ***29*** | -0.8 | -0.85 | -1.63 |
| LOC317612 | Rattus norvegicus similar to HIV TAT specific factor 1 | ***29*** | -0.59 | -1.01 | -1.78 |
| Col18a1 | Rattus norvegicus collagen, type XVIII, alpha 1 | ***29*** | -0.59 | -0.87 | -1.78 |
| Plxnb2 | Rattus norvegicus plexin B2 | ***29*** | -0.51 | -0.89 | -1.91 |
| Ctbp2 | Rattus norvegicus C-terminal binding protein 2 | ***29*** | -0.72 | -0.96 | -2.32 |
| Cyp4f6 | Rattus norvegicus cytochrome P450 4F6 | ***29*** | -0.62 | -0.94 | -2.24 |
| Gnao | Rattus norvegicus guanine nucleotide binding protein, alpha o | ***29*** | -0.59 | -0.86 | -2.38 |
| Sca2 | Rattus norvegicus spinocerebellar ataxia 2 | ***29*** | -0.47 | -0.78 | -2.15 |
| Trio | Rattus norvegicus triple functional domain | ***29*** | -0.25 | -1 | -2.22 |
| Helz | Rattus norvegicus helicase with zinc finger domain | ***29*** | -0.03 | -1.06 | -2.23 |
| Inpp5b | Rattus norvegicus inositol polyphosphate-5-phosphatase B | ***29*** | -0.09 | -0.79 | -2.54 |
| Irf3 | Rattus norvegicus interferon regulatory factor 3 | ***29*** | -0.41 | -0.73 | -1.56 |
| Wbp11 | Rattus norvegicus WW domain binding protein 11 | ***29*** | -0.38 | -0.95 | -1.6 |
| Vps16 | Rattus norvegicus vacuolar protein sorting 16 | ***29*** | -0.32 | -0.95 | -1.67 |
| Sec23ip | Rattus norvegicus SEC23 interacting protein | ***29*** | -0.28 | -0.96 | -1.54 |
| Dhx30 | Rattus norvegicus DEAH | ***29*** | -0.31 | -0.94 | -1.8 |
| Gstm3 | Rattus norvegicus glutathione S-transferase, mu type 3 | ***29*** | -0.3 | -0.92 | -1.79 |
| Sugt1 | Rattus norvegicus SGT1, suppressor of G2 allele of SKP1 | ***29*** | -0.23 | -0.91 | -1.73 |
| Cry1 | Rattus norvegicus cryptochrome 1 | ***29*** | -0.13 | -0.96 | -1.72 |
| Taf9 | Rattus norvegicus TAF9 RNA polymerase II, TATA box binding protein | ***29*** | -0.17 | -0.71 | -1.97 |
| Cherp | Rattus norvegicus calcium homeostasis endoplasmic reticulum protein | ***29*** | -0.03 | -0.71 | -2.02 |
| LOC363478 | Rattus norvegicus similar to sorting nexin 12 | ***29*** | 0.03 | -0.77 | -2 |
| Usp2 | Rattus norvegicus ubiquitin specific protease 2 | ***29*** | 0.04 | -0.73 | -1.82 |
| Luc7l2 | Rattus norvegicus LUC7-like 2 | ***29*** | 0.09 | -0.64 | -1.86 |
| Msln | Rattus norvegicus mesothelin | ***29*** | -0.72 | -0.92 | -3.19 |
| Dvl1 | Rattus norvegicus dishevelled, dsh homolog 1 | ***29*** | -0.39 | -0.92 | -3.22 |
| LOC363767 | Rattus norvegicus similar to ABI gene family, member 3 | ***29*** | -0.62 | 0.03 | -2.52 |
| LOC498279 | Rattus norvegicus similar to NADH dehydrogenase | ***29*** | -0.29 | -0.08 | -2.62 |
| Plagl1 | Rattus norvegicus pleiomorphic adenoma gene-like 1 | ***29*** | -0.6 | -0.21 | -2.86 |
| Myh10 | Rattus norvegicus myosin heavy chain 10, non-muscle | ***29*** | -0.48 | -0.14 | -2.85 |
| Ech1 | Rattus norvegicus enoyl coenzyme A hydratase 1, peroxisomal | ***29*** | -0.47 | -0.48 | -2.51 |
| Suv420h2 | Rattus norvegicus suppressor of variegation 4-20 homolog 2 | ***29*** | -0.32 | -0.36 | -2.59 |
| Ccnl2 | Rattus norvegicus cyclin L2 | ***29*** | -0.32 | -0.5 | -2.66 |
| Pbxip1 | Rattus norvegicus pre-B-cell leukemia transcription factor interacting protein 1 | ***29*** | -0.21 | -0.6 | -2.68 |
| Ugcg | Rattus norvegicus UDP-glucose ceramide glucosyltransferase | ***29*** | -0.03 | -0.83 | -3.05 |
| LOC361885 | Rattus norvegicus similar to LRRGT00194 | ***29*** | 0.45 | -0.52 | -2.72 |
|  |  |  |  |  |  |
| LOC360698 | Rattus norvegicus similar to RIKEN cDNA B230114J08 | ***16*** | -4.03 | -0.61 | -0.28 |
| Ctsk | Rattus norvegicus cathepsin K | ***16*** | -3.82 | -0.86 | 0.21 |
| Dnajc10 | Rattus norvegicus DnaJ | ***16*** | -3.71 | -1.75 | -0.73 |
| Cldn9 | Rattus norvegicus claudin 9 | ***16*** | -3.27 | -1.64 | -0.94 |
| Igsf11 | Rattus norvegicus immunoglobulin superfamily, member 11 | ***16*** | -3.65 | -1.51 | -0.2 |
| LOC360546 | Rattus norvegicus similar to m-ephrin-B3 | ***16*** | -3.08 | -1.72 | -0.64 |
| Rab11a | Rattus norvegicus RAB11a, member RAS oncogene family | ***16*** | -2.95 | -1.69 | -0.44 |
| Ppp4r2 | Rattus norvegicus protein phosphatase 4, regulatory subunit 2 | ***16*** | -3.04 | -1.84 | -0.13 |
| Hmgcr | Rattus norvegicus 3-hydroxy-3-methylglutaryl-Coenzyme A reductase | ***16*** | -3.07 | -1.25 | 0.14 |
| RGD1310861 | Rattus norvegicus similar to RIKEN cDNA 1500011H22 | ***16*** | -2.95 | -0.92 | 0.35 |
| LOC306587 | Rattus norvegicus similar to RIKEN cDNA 9130410M22 | ***16*** | -2.72 | -1.42 | 0.45 |
| RGD1305689 | Rattus norvegicus similar to DNA segment, Chr 14, ERATO Doi 449, expressed | ***16*** | -2.58 | -1.22 | 0.24 |
| Postn | Rattus norvegicus periostin, osteoblast specific factor | ***16*** | -2.95 | -1.13 | -0.22 |
| Pigq | Rattus norvegicus phosphatidylinositol glycan, class Q | ***16*** | -2.76 | -1.01 | -0.1 |
| LOC499772 | Rattus norvegicus similar to immediate early response 5-like | ***16*** | -2.6 | -0.81 | 0.49 |
| RGD1309685 | Rattus norvegicus similar to RIKEN cDNA 2310075G12 | ***16*** | -2.26 | -0.77 | 0.5 |
| LOC361467 | Rattus norvegicus similar to hypothetical protein FLJ20481 | ***16*** | -2.24 | -1.2 | 0.47 |
| LOC305310 | Rattus norvegicus similar to RIKEN cDNA 5033405K12 | ***16*** | -2.18 | -1.1 | 0.21 |
| LOC300149 | Rattus norvegicus similar to hypothetical protein D15Ertd785e | ***16*** | -2.04 | -0.45 | 0.55 |
| RGD1307632 | Rattus norvegicus similar to tumor-related protein | ***16*** | -2 | -0.41 | 0.62 |
| Pdk2 | Rattus norvegicus pyruvate dehydrogenase kinase, isoenzyme 2 | ***16*** | -1.91 | -0.43 | 0.7 |
| Id1 | Rattus norvegicus Inhibitor of DNA binding 1, helix-loop-helix protein | ***16*** | -1.96 | -0.34 | 0.42 |
| LOC301119 | Rattus norvegicus similar to Btk-PH-domain binding protein | ***16*** | -1.86 | -0.4 | 0.52 |
| Gorasp2 | Rattus norvegicus golgi reassembly stacking protein 2 | ***16*** | -1.83 | -0.38 | 0.51 |
| LOC313699 | Rattus norvegicus similar to RIKEN cDNA 2510039O18 | ***16*** | -1.88 | -0.28 | 0.56 |
| Rb1cc1 | Rattus norvegicus RB1-inducible coiled-coil 1 | ***16*** | -1.83 | -0.19 | 0.49 |
| Traf4af1 | Rattus norvegicus TRAF4 associated factor 1 | ***16*** | -1.63 | -0.56 | 0.7 |
| Tlk1 | Rattus norvegicus tousled-like kinase 1 | ***16*** | -1.58 | -0.41 | 0.4 |
| RGD1311316 | Rattus norvegicus similar to RIKEN cDNA 5730470L24 | ***16*** | -2.03 | -0.7 | 0.81 |
| Pofut2 | Rattus norvegicus protein O-fucosyltransferase 2 | ***16*** | -2.02 | -0.78 | 0.63 |
| Csrp2 | Rattus norvegicus cysteine and glycine-rich protein 2 | ***16*** | -2.03 | -0.84 | 0.41 |
| Sfpq | Rattus norvegicus NonO/p54nrb homolog | ***16*** | -1.98 | -0.89 | 0.29 |
| Snrpa | Rattus norvegicus small nuclear ribonucleoprotein polypeptide A | ***16*** | -2 | -0.73 | 0.2 |
| LOC292995 | Rattus norvegicus similar to BBP-like protein 2 | ***16*** | -1.97 | -1.02 | 0.37 |
| Cnot7 | Rattus norvegicus CCR4-NOT transcription complex, subunit 7 | ***16*** | -1.85 | -1.08 | 0.48 |
| Prkar2b | Rattus norvegicus protein kinase, cAMP dependent regulatory, type II beta | ***16*** | -1.93 | -1.01 | 0.6 |
| Rom1 | Rattus norvegicus rod outer segment membrane protein 1 | ***16*** | -1.84 | -1 | 0.63 |
| LOC310760 | Rattus norvegicus similar to cDNA sequence BC003236 | ***16*** | -1.86 | -0.8 | 0.38 |
| RGD1308470 | Rattus norvegicus similar to RIKEN cDNA 4933433P14 gene | ***16*** | -1.79 | -0.9 | 0.5 |
| Gtl3 | Rattus norvegicus gene trap locus 3 | ***16*** | -1.62 | -1.05 | 0.44 |
| Lama2 | Rattus norvegicus laminin, alpha 2 | ***16*** | -1.57 | -1.03 | 0.43 |
| Pgrmc1 | Rattus norvegicus progesterone receptor membrane component 1 | ***16*** | -1.54 | -0.89 | 0.43 |
| LOC360894 | Rattus norvegicus similar to hypothetical protein FLJ14146 | ***16*** | -1.58 | -0.98 | 0.22 |
| Nrep | Rattus norvegicus neuronal regeneration related protein | ***16*** | -2.98 | -1.98 | 0.31 |
| LOC294942 | Rattus norvegicus hypothetical LOC294942 | ***16*** | -2.59 | -2.19 | 0.09 |
| Cfh | Rattus norvegicus complement component factor H | ***16*** | -2.28 | -2.83 | -0.07 |
| Tfpi | Rattus norvegicus tissue factor pathway inhibitor | ***16*** | -1.76 | -2.54 | 0.22 |
| Calm1 | Rattus norvegicus calmodulin 1 | ***16*** | -1.74 | -2.49 | 0.11 |
| LOC317218 | Rattus norvegicus similar to Integral membrane protein 2A | ***16*** | -2.07 | -1.33 | 0.09 |
| LOC498750 | Rattus norvegicus similar to cDNA sequence BC005537 | ***16*** | -2.05 | -1.4 | -0.11 |
| Cul3 | Rattus norvegicus cullin 3 | ***16*** | -1.74 | -1.33 | 0.06 |
| LOC316916 | Rattus norvegicus similar to cisplatin resistance related protein CRR9p | ***16*** | -1.8 | -1.11 | 0.05 |
| Map3k4 | Rattus norvegicus mitogen activated protein kinase kinase kinase 4 | ***16*** | -1.66 | -1.06 | 0.1 |
| LOC299341 | Rattus norvegicus similar to RIKEN cDNA 2810002N01 | ***16*** | -1.72 | -1.02 | -0.12 |
| Sfxn1 | Rattus norvegicus sideroflexin 1 | ***16*** | -1.66 | -1.25 | -0.15 |
| LOC501550 | Rattus norvegicus similar to Sedlin | ***16*** | -1.52 | -1.22 | -0.23 |
| Ucp2 | Rattus norvegicus uncoupling protein 2 | ***16*** | -1.91 | -1.56 | 0.27 |
| G3bp | Rattus norvegicus Ras-GTPase-activating protein SH3-domain binding protein | ***16*** | -1.56 | -1.46 | 0.43 |
| Pcsk1n | Rattus norvegicus proprotein convertase subtilisin/kexin type 1 inhibitor | ***16*** | -1.28 | -1.89 | 0.33 |
| RGD1310686 | Rattus norvegicus similar to chromosome 16 open reading frame 5 | ***16*** | -1.28 | -1.74 | 0.27 |
| LOC501157 | Rattus norvegicus similar to Ribulose-5-phosphate-3-epimerase | ***16*** | -1.19 | -1.59 | 0.41 |
| Bf | Rattus norvegicus B-factor, properdin | ***16*** | -1.19 | -1.61 | 0.45 |
| Smoc2 | Rattus norvegicus SPARC related modular calcium binding 2 | ***16*** | -1.61 | -2.18 | 0.03 |
| RGD1307008 | Rattus norvegicus similar to RIKEN cDNA 4833420K19 | ***16*** | -1.53 | -2.06 | -0.09 |
| LOC499513 | Rattus norvegicus LOC499513 | ***16*** | -1.43 | -2.16 | -0.15 |
| Dhx32 | Rattus norvegicus DEAH | ***16*** | -1.12 | -2.08 | 0.06 |
| Fkbp5 | Rattus norvegicus FK506 binding protein 5 | ***16*** | -1.45 | -1.7 | 0.03 |
| Gbp2 | Rattus norvegicus guanylate nucleotide binding protein 2 | ***16*** | -1.39 | -1.61 | 0.01 |
| LOC361988 | Rattus norvegicus similar to expressed sequence C77668 | ***16*** | -1.23 | -1.8 | 0.08 |
| Prtfdc1 | Rattus norvegicus phosphoribosyl transferase domain containing 1 | ***16*** | -1.29 | -1.72 | -0.16 |
| Ifi44 | Rattus norvegicus interferon-induced protein 44 | ***16*** | -1.14 | -1.78 | -0.21 |
| Ptdss2 | Rattus norvegicus phosphatidylserine synthase 2 | ***16*** | -1.13 | -1.81 | -0.15 |
| LOC246187 | Rattus norvegicus liver regeneration-related protein | ***16*** | -1.1 | -1.67 | -0.32 |
| LOC315973 | Rattus norvegicus similar to 5730439E10Rik protein | ***16*** | -1.02 | -1.62 | -0.08 |
| LOC360886 | Rattus norvegicus similar to transcription factor ELYS | ***16*** | -2.86 | -1.44 | -0.65 |
| B4galt6 | Rattus norvegicus UDP-Gal:betaGlcNAc beta 1,4-galactosyltransferase, polypeptide 6 | ***16*** | -2.81 | -1.28 | -0.51 |
| Asf1a | Rattus norvegicus ASF1 anti-silencing function 1 homolog A | ***16*** | -2.52 | -1.15 | -0.79 |
| LOC306324 | Rattus norvegicus similar to tetraspanin similar to TM4SF9 | ***16*** | -2.39 | -1.4 | -0.69 |
| LOC304091 | Rattus norvegicus similar to class II cytokine receptor 4 | ***16*** | -2.46 | -0.97 | -1.05 |
| Cbfb | Rattus norvegicus core binding factor beta | ***16*** | -2.45 | -0.84 | -0.99 |
| Reck | Rattus norvegicus reversion-inducing-cysteine-rich protein with kazal motifs | ***16*** | -2.19 | -1.07 | -0.93 |
| Zfp347 | Rattus norvegicus zinc finger protein 347 | ***16*** | -2.14 | -0.93 | -0.85 |
| LOC310640 | Rattus norvegicus similar to chromosome 1 open reading frame 2 | ***16*** | -2.32 | -1.17 | -0.98 |
| Mcmd6 | Rattus norvegicus mini chromosome maintenance deficient 6 | ***16*** | -2.18 | -1.27 | -1.01 |
| Api5 | Rattus norvegicus apoptosis inhibitor 5 | ***16*** | -2.22 | -1.12 | -1.18 |
| LOC500629 | Rattus norvegicus similar to alcohol dehydrogenase PAN2 | ***16*** | -2.17 | -1.37 | -0.42 |
| Rbm24 | Rattus norvegicus RNA binding motif protein 24 | ***16*** | -2.16 | -0.99 | -0.66 |
| Leprot | Rattus norvegicus leptin receptor overlapping transcript | ***16*** | -2.09 | -0.98 | -0.46 |
| LOC500015 | Rattus norvegicus similar to mKIAA2005 protein | ***16*** | -2.06 | -1.28 | -0.61 |
| Fxyd6 | Rattus norvegicus FXYD domain-containing ion transport regulator 6 | ***16*** | -2.02 | -1.29 | -0.8 |
| LOC299828 | Rattus norvegicus similar to Ku70-binding protein 3 | ***16*** | -1.88 | -1.26 | -0.82 |
| Psmb10 | Rattus norvegicus proteasome | ***16*** | -1.74 | -1.23 | -0.79 |
| Zmpste24 | Rattus norvegicus zinc metalloproteinase, STE24 homolog | ***16*** | -1.87 | -1.12 | -0.76 |
| B4galt4 | Rattus norvegicus UDP-Gal:betaGlcNAc beta 1,4-galactosyltransferase, polypeptide 4 | ***16*** | -1.86 | -1.05 | -0.75 |
| AY228474 | Rattus norvegicus DNA sequence AY228474 | ***16*** | -2.46 | -1.76 | -0.73 |
| LOC368066 | Rattus norvegicus similar to thioether S-methyltransferase | ***16*** | -2.3 | -1.76 | -0.61 |
| MGC94969 | Rattus norvegicus transmembrane protein vezatin | ***16*** | -2.24 | -1.75 | -0.62 |
| LOC171553 | Rattus norvegicus iGb3 synthase | ***16*** | -2.32 | -2.08 | -0.51 |
| Zic2 | Rattus norvegicus Zic family member 2 | ***16*** | -2.17 | -2.08 | -0.81 |
| Paqr4 | Rattus norvegicus progestin and adipoQ receptor family member IV | ***16*** | -2.09 | -2.22 | -0.4 |
| Dcn | Rattus norvegicus decorin | ***16*** | -1.81 | -2.22 | -0.35 |
| Lypla1 | Rattus norvegicus lysophospholipase 1 | ***16*** | -1.84 | -1.69 | -0.24 |
| Adprt | Rattus norvegicus ADP-ribosyltransferase 1 | ***16*** | -1.59 | -1.88 | -0.26 |
| Lrp11 | Rattus norvegicus low density lipoprotein receptor-related protein 11 | ***16*** | -1.49 | -2.11 | -0.71 |
| LOC287274 | Rattus norvegicus similar to RIKEN cDNA 0610009B22 | ***16*** | -1.44 | -1.84 | -0.56 |
| Jun | Rattus norvegicus v-jun sarcoma virus 17 oncogene homolog | ***16*** | -1.43 | -1.94 | -0.42 |
| Pros1 | Rattus norvegicus protein S | ***16*** | -1.86 | -1.51 | -0.48 |
| LOC288707 | Rattus norvegicus similar to Glycolipid transfer protein | ***16*** | -1.63 | -1.46 | -0.4 |
| Trp53rk | Rattus norvegicus TP53 regulating kinase | ***16*** | -1.79 | -1.3 | -0.66 |
| LOC292751 | Rattus norvegicus similar to RIKEN cDNA 2810405O22 | ***16*** | -1.66 | -1.33 | -0.67 |
| LOC302553 | Rattus norvegicus similar to Su | ***16*** | -1.63 | -1.26 | -0.65 |
| Hnrpa3 | Rattus norvegicus heterogeneous nuclear ribonucleoprotein A3 | ***16*** | -1.78 | -1.52 | -0.73 |
| LOC502782 | Rattus norvegicus similar to RIKEN cDNA 2610022G08 | ***16*** | -1.56 | -1.4 | -0.79 |
| Ier2 | Rattus norvegicus immediate early response 2 | ***16*** | -1.54 | -1.76 | -0.74 |
| LOC315158 | Rattus norvegicus similar to ubiquitous tetratricopeptide containing protein RoXaN; Rotavirus X associated non-structural protein | ***16*** | -1.32 | -1.68 | -0.85 |
| Urod | Rattus norvegicus uroporphyrinogen decarboxylase | ***16*** | -1.44 | -1.63 | -0.41 |
| LOC293566 | Rattus norvegicus similar to carboxypeptidase X 2 | ***16*** | -1.35 | -1.59 | -0.54 |
| Nt5c3 | Rattus norvegicus 5'-nucleotidase, cytosolic III | ***16*** | -1.24 | -1.56 | -0.44 |
|  |  |  |  |  |  |
| Dscr1 | Rattus norvegicus Down syndrome critical region homolog 1 | ***22*** | -3.11 | 0.4 | -1.39 |
| LOC499775 | Rattus norvegicus LOC499775 | ***22*** | -2.71 | 0.64 | -1.43 |
| LOC309475 | Rattus norvegicus similar to transmembrane protein TM9SF3 | ***22*** | -2.62 | 0.74 | -0.74 |
| LOC498407 | Rattus norvegicus similar to purine rich element binding protein B | ***22*** | -2.45 | 0.67 | -0.64 |
| Col3a1 | Rattus norvegicus collagen, type III, alpha 1 | ***22*** | -2.22 | 0.77 | -0.44 |
| Pphln1 | Rattus norvegicus periphilin 1 | ***22*** | -2.16 | 0.67 | -1.29 |
| Plxnd1 | Rattus norvegicus plexin D1 | ***22*** | -1.79 | 0.63 | -1.13 |
| LOC288659 | Rattus norvegicus similar to Hypothetical protein MGC25614 | ***22*** | -1.68 | 0.56 | -1.23 |
| LOC303514 | Rattus norvegicus similar to RIKEN cDNA 4121402D02 | ***22*** | -2.58 | -0.4 | -1.27 |
| RGD1310386 | Rattus norvegicus similar to hypothetical protein MGC10067 | ***22*** | -2.36 | -0.24 | -1.14 |
| RGD1305625 | Rattus norvegicus similar to RIKEN cDNA 2310075C12 | ***22*** | -2.48 | -0.53 | -0.86 |
| Ei24 | Rattus norvegicus etoposide induced 2.4 mRNA | ***22*** | -2.37 | -0.5 | -0.93 |
| LOC289400 | Rattus norvegicus similar to KIAA1078 protein | ***22*** | -2.3 | -0.64 | -1.22 |
| LOC312915 | Rattus norvegicus similar to brefeldin A-inhibited guanine nucleotide-exchange protein 1 | ***22*** | -2.12 | -0.73 | -1.38 |
| Siat4b | Rattus norvegicus sialyltransferase 4B | ***22*** | -2.06 | -0.48 | -1.16 |
| Tia1 | Rattus norvegicus cytotoxic granule-associated RNA binding protein 1 | ***22*** | -2.34 | 0.09 | -1.13 |
| LOC501282 | Rattus norvegicus similar to lymphocyte antigen 6 complex, locus E ligand | ***22*** | -2.12 | -0.03 | -1.23 |
| MGC94555 | Rattus norvegicus intimal thickness-related receptor | ***22*** | -1.93 | 0.02 | -1.05 |
| LOC360738 | Rattus norvegicus similar to Ser/Thr-rich protein T10 in DGCR region | ***22*** | -1.83 | 0.09 | -0.86 |
| Rrm2 | Rattus norvegicus ribonucleotide reductase M2 | ***22*** | -1.76 | -0.05 | -1.02 |
| Nov | Rattus norvegicus nephroblastoma overexpressed gene | ***22*** | -1.67 | -0.02 | -0.98 |
| LOC498410 | Rattus norvegicus similar to novel protein similar to Tensin Tns | ***22*** | -1.9 | -0.34 | -1.36 |
| Statip1 | Rattus norvegicus signal transducer and activator of transcription interacting protein 1 | ***22*** | -1.89 | -0.39 | -1.45 |
| Slc33a1 | Rattus norvegicus solute carrier family 33 | ***22*** | -1.89 | -0.06 | -1.47 |
| Slc29a3 | Rattus norvegicus solute carrier family 29 | ***22*** | -1.79 | -0.05 | -1.35 |
| Ifitm1 | Rattus norvegicus interferon induced transmembrane protein 1 | ***22*** | -1.77 | -0.07 | -1.29 |
| Col11a1 | Rattus norvegicus procollagen, type XI, alpha 1 | ***22*** | -2.05 | -0.78 | -0.39 |
| RGD1305133 | Rattus norvegicus similar to Ab2-008 | ***22*** | -2.03 | -0.84 | -0.53 |
| Bbp | Rattus norvegicus beta-amyloid binding protein precursor | ***22*** | -1.9 | -0.78 | -0.56 |
| Emilin1 | Rattus norvegicus elastin microfibril interfacer 1 | ***22*** | -1.88 | -0.7 | -0.27 |
| LOC308708 | Rattus norvegicus similar to hypothetical protein FLJ12572 | ***22*** | -1.85 | -0.8 | -0.32 |
| Ccna2 | Rattus norvegicus cyclin A2 | ***22*** | -1.77 | -0.72 | -0.39 |
| Exosc7 | Rattus norvegicus exosome component 7 | ***22*** | -1.66 | -0.91 | -0.94 |
| RGD1310022 | Rattus norvegicus similar to RIKEN cDNA 2610204K14 | ***22*** | -1.61 | -1.13 | -0.85 |
| Mak10 | Rattus norvegicus corneal wound healing related protein | ***22*** | -1.59 | -1.09 | -0.84 |
| Tsnax | Rattus norvegicus translin-associated factor X | ***22*** | -1.95 | -0.49 | -0.71 |
| LOC292792 | Rattus norvegicus similar to seven transmembrane domain protein | ***22*** | -1.9 | -0.3 | -0.89 |
| Slc16a1 | Rattus norvegicus solute carrier family 16 | ***22*** | -1.89 | -0.35 | -0.75 |
| Rarres2 | Rattus norvegicus retinoic acid receptor responder | ***22*** | -1.85 | -0.69 | -0.78 |
| LOC302313 | Rattus norvegicus similar to transmembrane 4 superfamily member 6 | ***22*** | -1.73 | -0.69 | -0.75 |
| Dutp | Rattus norvegicus deoxyuridine triphosphatase | ***22*** | -1.66 | -0.65 | -0.75 |
| LOC303630 | Rattus norvegicus similar to D11Ertd498e protein | ***22*** | -1.68 | -0.54 | -0.65 |
| Cdkn3 | Rattus norvegicus cyclin-dependent kinase inhibitor 3 | ***22*** | -1.55 | -0.56 | -0.62 |
| Ube4b | Rattus norvegicus ubiquitination factor E4B, UFD2 homolog | ***22*** | -1.52 | -0.51 | -0.58 |
| Cxcr4 | Rattus norvegicus chemokine | ***22*** | -1.64 | -0.38 | -0.64 |
| Ythdf2 | Rattus norvegicus YTH domain family 2 | ***22*** | -1.52 | -0.43 | -0.56 |
| Msh6 | Rattus norvegicus mutS homolog 6 | ***22*** | -1.83 | -0.58 | -1.07 |
| Lrrk1 | Rattus norvegicus leucine-rich repeat kinase 1 | ***22*** | -1.81 | -0.59 | -1.1 |
| Pycrl | Rattus norvegicus pyrroline-5-carboxylate reductase-like | ***22*** | -1.74 | -0.54 | -0.96 |
| Ubl3 | Rattus norvegicus ubiquitin-like 3 | ***22*** | -1.51 | -0.68 | -1.02 |
| LOC498035 | Rattus norvegicus similar to cylindromatosis | ***22*** | -1.69 | -0.46 | -1.27 |
| Fh1 | Rattus norvegicus fumarate hydratase 1 | ***22*** | -1.66 | -0.51 | -1.26 |
| Eppb9 | Rattus norvegicus endothelial precursor protein B9 | ***22*** | -1.56 | -0.5 | -1.24 |
| Cpt2 | Rattus norvegicus carnitine palmitoyltransferase 2 | ***22*** | -3.09 | -0.25 | 0.3 |
| Phc2 | Rattus norvegicus polyhomeotic-like 2 | ***22*** | -2.62 | -0.28 | 0.29 |
| Tmem9 | Rattus norvegicus transmembrane protein 9 | ***22*** | -2.99 | 0.02 | -0.45 |
| Pfn2 | Rattus norvegicus profilin 2 | ***22*** | -2.86 | -0.37 | -0.52 |
| MGC94167 | Rattus norvegicus similar to RIKEN cDNA C130099A20 | ***22*** | -2.68 | 0.4 | 0.37 |
| Csf1 | Rattus norvegicus colony stimulating factor 1 | ***22*** | -2.66 | 0.27 | -0.3 |
| LOC501028 | Rattus norvegicus similar to mitochondrial ribosomal protein L41 | ***22*** | -2.57 | 0 | -0.23 |
| LOC499380 | Rattus norvegicus similar to empty spiracles-like protein 2 | ***22*** | -2.6 | 0.19 | -0.04 |
| Nfatc4 | Rattus norvegicus nuclear factor of activated T-cells, cytoplasmic, calcineurin-dependent 4 | ***22*** | -2.56 | 0.32 | 0.06 |
| Thumpd1 | Rattus norvegicus THUMP domain containing 1 | ***22*** | -2.7 | -0.74 | -0.52 |
| Prkaca | Rattus norvegicus protein kinase, cAMP-dependent, catalytic, alpha | ***22*** | -2.66 | -0.61 | -0.64 |
| Ripk5 | Rattus norvegicus receptor interacting protein kinase 5 | ***22*** | -2.42 | -0.52 | -0.55 |
| LOC287212 | Rattus norvegicus similar to hypothetical protein FLJ31951 | ***22*** | -2.27 | -0.71 | -0.54 |
| Gbl | Rattus norvegicus G protein beta subunit-like | ***22*** | -2.58 | -0.62 | -0.33 |
| Crot | Rattus norvegicus carnitine O-octanoyltransferase | ***22*** | -2.54 | -0.88 | -0.32 |
| Crabp2 | Rattus norvegicus cellular retinoic acid binding protein 2 | ***22*** | -2.51 | -0.66 | 0.05 |
| RGD1310553 | Rattus norvegicus similar to expressed sequence AI597479 | ***22*** | -2.22 | -0.92 | -0.17 |
| Mknk2 | Rattus norvegicus MAP kinase-interacting serine/threonine kinase 2 | ***22*** | -2.2 | -0.78 | -0.2 |
| Anxa11 | Rattus norvegicus annexin A11 | ***22*** | -2.49 | -0.04 | -0.5 |
| Sart2 | Rattus norvegicus squamous cell carcinoma antigen recognized by T cells 2 | ***22*** | -2.4 | -0.22 | -0.65 |
| Zhx1 | Rattus norvegicus zinc-fingers and homeoboxes 1 | ***22*** | -2.35 | -0.21 | -0.62 |
| Tm9sf4 | Rattus norvegicus transmembrane 9 superfamily protein member 4 | ***22*** | -2.45 | -0.28 | -0.46 |
| MGC94686 | Rattus norvegicus similar to RIKEN cDNA 2810413N20 | ***22*** | -2.32 | -0.32 | -0.33 |
| S100a16 | Rattus norvegicus S100 calcium binding protein A16 | ***22*** | -2.22 | -0.47 | -0.45 |
| Fbxo11 | Rattus norvegicus F-box only protein 11 | ***22*** | -2.2 | -0.47 | -0.38 |
| Ssbp1 | Rattus norvegicus single-stranded DNA binding protein 1 | ***22*** | -2.01 | -0.48 | -0.49 |
| Ctps | Rattus norvegicus cytidine 5'-triphosphate synthase | ***22*** | -2.06 | -0.22 | -0.27 |
| Lsm8 | Rattus norvegicus LSM8 homolog, U6 small nuclear RNA associated | ***22*** | -2.03 | -0.31 | -0.22 |
| Pon2 | Rattus norvegicus paraoxonase 2 | ***22*** | -1.99 | -0.35 | -0.33 |
| Cdipt | Rattus norvegicus CDP-diacylglycerol--inositol 3-phosphatidyltransferase | ***22*** | -1.98 | -0.2 | -0.51 |
| Fut8 | Rattus norvegicus fucosyltransferase 8 | ***22*** | -2.44 | -0.08 | -0.03 |
| Olfml2b | Rattus norvegicus olfactomedin-like 2B | ***22*** | -2.25 | -0.11 | 0.04 |
| Cfl2 | Rattus norvegicus cofilin 2, muscle | ***22*** | -2.43 | -0.31 | -0.09 |
| RGD1305486 | Rattus norvegicus similar to RIKEN cDNA 2810405J04 | ***22*** | -2.43 | -0.33 | -0.18 |
| Alg5 | Rattus norvegicus asparagine-linked glycosylation 5 homolog | ***22*** | -2.29 | -0.36 | 0.33 |
| Rhoq | Rattus norvegicus ras homolog gene family, member Q | ***22*** | -2.25 | -0.32 | 0.23 |
| LOC497673 | Rattus norvegicus hypothetical gene supported by NM_172035 | ***22*** | -2.27 | -0.21 | 0.34 |
| LOC360821 | Rattus norvegicus similar to Putative protein 15E1.2 | ***22*** | -2.09 | -0.37 | 0.24 |
| LOC360632 | Rattus norvegicus similar to RIKEN cDNA 1300010M03 | ***22*** | -2.04 | -0.43 | 0.21 |
| Lepre1 | Rattus norvegicus leprecan 1 | ***22*** | -2.01 | -0.33 | 0.15 |
| RGD1305356 | Rattus norvegicus similar to RIKEN cDNA 3110031B13 | ***22*** | -1.92 | -0.63 | -0.02 |
| Blcap | Rattus norvegicus bladder cancer associated protein homolog | ***22*** | -1.91 | -0.51 | -0.08 |
| LOC497712 | Rattus norvegicus hypothetical gene supported by NM_001001511 | ***22*** | -1.79 | -0.64 | 0.24 |
| LOC498107 | Rattus norvegicus LOC498107 | ***22*** | -1.68 | -0.68 | 0.14 |
| Lgals3bp | Rattus norvegicus lectin, galactoside-binding, soluble, 3 binding protein | ***22*** | -1.88 | -0.84 | -0.09 |
| Cyp1b1 | Rattus norvegicus cytochrome P450, family 1, subfamily b, polypeptide 1 | ***22*** | -1.82 | -0.75 | -0.04 |
| D123 | Rattus norvegicus D123 gene product | ***22*** | -1.79 | -0.68 | 0.02 |
| LOC501633 | Rattus norvegicus LOC501633 | ***22*** | -1.64 | -0.75 | -0.21 |
| Rcn2 | Rattus norvegicus reticulocalbin 2 | ***22*** | -1.56 | -0.75 | -0.08 |
| RGD1311243 | Rattus norvegicus similar to DKFZP566O084 protein | ***22*** | -1.69 | -0.52 | -0.21 |
| Prr3 | Rattus norvegicus proline-rich polypeptide 3 | ***22*** | -1.68 | -0.47 | -0.14 |
| MGC94282 | Rattus norvegicus similar to 5930416I19Rik protein | ***22*** | -1.54 | -0.59 | -0.11 |
| Hint3 | Rattus norvegicus histidine triad nucleotide binding protein 3 | ***22*** | -1.52 | -0.51 | -0.09 |
| Fndc3 | Rattus norvegicus fibronectin type III domain containing 3 | ***22*** | -1.67 | -0.41 | 0.02 |
| Sc65 | Rattus norvegicus synaptonemal complex protein SC65 | ***22*** | -2.35 | 0.29 | -0.33 |
| Atp1b1 | Rattus norvegicus ATPase, Na+/K+ transporting, beta 1 polypeptide | ***22*** | -2.19 | 0.2 | -0.51 |
| Slc6a8 | Rattus norvegicus choline transporter | ***22*** | -2.21 | 0.02 | -0.31 |
| LOC289181 | Rattus norvegicus similar to IQ motif and WD repeats 1 | ***22*** | -2.16 | 0.06 | -0.39 |
| Coq3 | Rattus norvegicus coenzyme Q3 homolog, methyltransferase | ***22*** | -2.2 | -0.13 | -0.44 |
| LOC500039 | Rattus norvegicus similar to Adenylate kinase 2 | ***22*** | -2.04 | -0.08 | -0.08 |
| Mospd3 | Rattus norvegicus motile sperm domain containing 3 | ***22*** | -2 | -0.01 | -0.35 |
| Laptm4b | Rattus norvegicus lysosomal-associated protein transmembrane 4B | ***22*** | -1.86 | -0.04 | -0.28 |
| Kai1 | Rattus norvegicus kangai 1 | ***22*** | -1.81 | 0 | -0.29 |
| Psmb3 | Rattus norvegicus proteasome | ***22*** | -1.99 | 0.25 | -0.09 |
| Lass2 | Rattus norvegicus longevity assurance homolog 2 | ***22*** | -2.07 | 0.41 | -0.57 |
| Dab2 | Rattus norvegicus disabled homolog 2 | ***22*** | -1.92 | 0.45 | -0.7 |
| LOC361605 | Rattus norvegicus similar to mKIAA0824 protein | ***22*** | -1.95 | 0.13 | -0.66 |
| Dnajc9 | Rattus norvegicus DnaJ | ***22*** | -1.95 | -0.08 | -0.63 |
| Ehd4 | Rattus norvegicus EH-domain containing 4 | ***22*** | -1.92 | -0.06 | -0.69 |
| LOC500462 | Rattus norvegicus similar to calcium binding protein P22 | ***22*** | -1.76 | 0.04 | -0.69 |
| Chst2 | Rattus norvegicus carbohydrate sulfotransferase 2 | ***22*** | -1.65 | -0.03 | -0.61 |
| Ctsh | Rattus norvegicus cathepsin H | ***22*** | -1.61 | 0.34 | -0.58 |
| LOC361502 | Rattus norvegicus similar to B430201G11Rik protein | ***22*** | -1.56 | 0.22 | -0.52 |
| Ext2 | Rattus norvegicus exostoses | ***22*** | -1.53 | 0.25 | -0.56 |
| Scamp2 | Rattus norvegicus secretory carrier membrane protein 2 | ***22*** | -1.6 | 0.01 | -0.36 |
| LOC361315 | Rattus norvegicus similar to Ac2-256 | ***22*** | -1.51 | 0.04 | -0.56 |
| mrpl11 | Rattus norvegicus mitochondrial ribosomal protein L11 | ***22*** | -1.73 | -0.19 | -0.1 |
| Chp | Rattus norvegicus calcium binding protein p22 | ***22*** | -1.71 | -0.07 | -0.17 |
| Cd2bp2 | Rattus norvegicus CD2 antigen | ***22*** | -1.58 | -0.15 | -0.22 |
| LOC499335 | Rattus norvegicus similar to frataxin | ***22*** | -1.63 | -0.36 | -0.38 |
| Snx4 | Rattus norvegicus sorting nexin 4 | ***22*** | -1.61 | -0.43 | -0.28 |
| LOC290833 | Rattus norvegicus similar to RIKEN cDNA 2410018G23 | ***22*** | -1.51 | 0.26 | -0.28 |
| Hat1 | Rattus norvegicus histone aminotransferase 1 | ***22*** | -1.5 | 0.04 | -0.09 |
| LOC362040 | Rattus norvegicus similar to RIKEN cDNA 2310008M10 | ***22*** | -2.25 | 0.09 | 0.37 |
| Pigs | Rattus norvegicus phosphatidylinositol glycan, class S | ***22*** | -2.13 | -0.04 | 0.51 |
| RGD1307203 | Rattus norvegicus hypothetical LOC287199 | ***22*** | -1.91 | 0.35 | 0.49 |
| LOC290925 | Rattus norvegicus similar to RIKEN cDNA 2410022L05 | ***22*** | -1.86 | 0.24 | 0.32 |
| Evl | Rattus norvegicus Ena-vasodilator stimulated phosphoprotein | ***22*** | -1.91 | 0.01 | 0.24 |
| Bcat2 | Rattus norvegicus branched chain aminotransferase 2, mitochondrial | ***22*** | -1.85 | -0.13 | 0.35 |
| Ywhah | Rattus norvegicus tyrosine 3-monooxygenase/tryptophan 5-monooxygenase activation protein, eta polypeptide | ***22*** | -1.69 | -0.01 | 0.37 |
| Amacr | Rattus norvegicus alpha-methylacyl-CoA racemase | ***22*** | -1.65 | -0.09 | 0.18 |
| Mocs2 | Rattus norvegicus molybdopterin synthase | ***22*** | -1.62 | -0.05 | 0.09 |
| RGD1310284 | Rattus norvegicus similar to RIKEN cDNA C230075L19 gene | ***22*** | -1.53 | -0.02 | 0.03 |
| Fjx1 | Rattus norvegicus four jointed box 1 | ***22*** | -1.65 | 0.08 | 0.59 |
| Map2k4 | Rattus norvegicus mitogen activated protein kinase kinase 4 | ***22*** | -1.62 | 0.08 | 0.63 |
| Mrps17 | Rattus norvegicus mitochondrial ribosomal protein S17 | ***22*** | -1.53 | -0.05 | 0.65 |
| LOC361712 | Rattus norvegicus similar to HRD1 protein; synoviolin 1 | ***22*** | -1.52 | -0.16 | 0.45 |
| Peci | Rattus norvegicus peroxisomal delta3, delta2-enoyl-Coenzyme A isomerase | ***22*** | -1.5 | -0.1 | 0.45 |
| LOC499087 | Rattus norvegicus similar to MKIAA1064 protein | ***22*** | -2.14 | 0.58 | -0.07 |
| Cntf | Rattus norvegicus ciliary neurotrophic factor | ***22*** | -2.08 | 0.64 | -0.2 |
| Tpbg | Rattus norvegicus trophoblast glycoprotein | ***22*** | -1.94 | 0.75 | 0.04 |
| Sara1 | Rattus norvegicus SAR1a gene homolog 1 | ***22*** | -1.93 | 0.71 | 0.06 |
| Mlycd | Rattus norvegicus malonyl-CoA decarboxylase | ***22*** | -1.98 | 0.93 | -0.17 |
| LOC360627 | Rattus norvegicus similar to 65kDa FK506-binding protein | ***22*** | -1.93 | 0.83 | -0.06 |
| c-fos | Rattus norvegicus c-fos oncogene | ***22*** | -1.86 | 0.81 | -0.13 |
| Kdelr3 | Rattus norvegicus KDEL | ***22*** | -1.82 | 0.91 | -0.07 |
| Arf2 | Rattus norvegicus ADP-ribosylation factor 2 | ***22*** | -1.78 | 0.53 | -0.26 |
| Krt1-19 | Rattus norvegicus keratin complex 1, acidic, gene 19 | ***22*** | -1.65 | 0.53 | -0.42 |
| Exosc4 | Rattus norvegicus exosome component 4 | ***22*** | -1.64 | 0.43 | -0.37 |
| Ndufa8 | Rattus norvegicus NADH dehydrogenase | ***22*** | -1.53 | 0.86 | -0.37 |
| Fgfrl1 | Rattus norvegicus fibroblast growth factor receptor-like 1 | ***22*** | -1.67 | 0.34 | 0.09 |
| Slc39a13 | Rattus norvegicus solute carrier family 39 | ***22*** | -1.61 | 0.22 | 0.24 |
| Mkl1 | Rattus norvegicus megakaryoblastic leukemia | ***22*** | -1.64 | 0.56 | 0.37 |
| Rbm4 | Rattus norvegicus RNA binding motif protein 4 | ***22*** | -1.6 | 0.56 | 0.17 |
| Arpc1a | Rattus norvegicus actin related protein 2/3 complex, subunit 1A | ***22*** | -1.58 | 0.58 | 0.04 |
| Rab3d | Rattus norvegicus RAB3D, member RAS oncogene family | ***22*** | -1.52 | 0.56 | 0.03 |
| LOC303567 | Rattus norvegicus similar to RIKEN cDNA 2010008E23 gene | ***22*** | -1.58 | 0.71 | 0.01 |
| Ipo13 | Rattus norvegicus importin 13 | ***22*** | -1.57 | 0.43 | -0.01 |
| Pank2 | Rattus norvegicus pantothenate kinase 2 | ***22*** | -1.51 | 0.37 | -0.07 |
| Rcn | Rattus norvegicus reticulocalbin | ***22*** | -1.56 | 0.51 | -0.24 |
| Psma5 | Rattus norvegicus proteasome | ***22*** | -1.55 | 0.46 | -0.15 |
| Stard3nl | Rattus norvegicus STARD3 N-terminal like | ***22*** | -2.17 | 1.15 | -0.3 |
| Atp5j | Rattus norvegicus ATP synthase, H+ transporting, mitochondrial F0 complex, subunit F6 | ***22*** | -2.16 | 1.3 | -0.53 |
| LOC366481 | Rattus norvegicus similar to Zgc:56193 | ***22*** | -1.66 | 1.39 | -0.26 |
| LOC362578 | Rattus norvegicus similar to RIKEN cDNA 2410005K17 | ***22*** | -1.6 | 1.25 | -0.49 |
| Fzd1 | Rattus norvegicus frizzled homolog 1 | ***22*** | -1.78 | 1.01 | -0.73 |
| RGD1307935 | Rattus norvegicus similar to Hypothetical protein MGC18716 | ***22*** | -1.65 | 0.89 | -0.84 |
| Casp2 | Rattus norvegicus caspase 2 | ***22*** | -1.76 | 0.79 | -0.6 |
| LOC296733 | Rattus norvegicus similar to mKIAA1402 protein | ***22*** | -1.73 | 0.69 | -0.64 |
| Psme2 | Rattus norvegicus protease | ***22*** | -1.56 | 0.83 | -0.64 |
| Pabpn1 | Rattus norvegicus poly | ***22*** | -1.84 | 1.32 | -1.33 |
| Svil | Rattus norvegicus supervillin | ***22*** | -1.75 | 1.05 | -1.12 |
| LOC499496 | Rattus norvegicus LOC499496 | ***22*** | -1.75 | 0.85 | -1.13 |
| LOC498433 | Rattus norvegicus similar to proteasome | ***22*** | -1.56 | 1.44 | -0.81 |
| LOC298841 | Rattus norvegicus similar to apoptosis related protein APR-3; p18 protein | ***22*** | -0.98 | 1.65 | -0.44 |
| LOC290912 | Rattus norvegicus similar to hypothetical protein FLJ10154 | ***22*** | -0.96 | 1.74 | -0.51 |
| Ssr2 | Rattus norvegicus signal sequence receptor, beta | ***22*** | -0.8 | 1.58 | -0.57 |
| Exosc8 | Rattus norvegicus exosome component 8 | ***22*** | -0.55 | 1.65 | -0.54 |
| LOC293632 | Rattus norvegicus similar to RIKEN cDNA 6330512M04 gene | ***22*** | -0.74 | 1.99 | -0.63 |
| Ndufa5 | Rattus norvegicus NADH dehydrogenase | ***22*** | -0.61 | 1.86 | -0.93 |
| Dnm1 | Rattus norvegicus dynamin 1 | ***22*** | -0.59 | 1.76 | -0.78 |
| Tcirg1 | Rattus norvegicus T-cell, immune regulator 1, ATPase, H+ transporting, lysosomal V0 protein a isoform 3 | ***22*** | -0.98 | 1.57 | -1.4 |
| Arrdc1 | Rattus norvegicus arrestin domain containing 1 | ***22*** | -0.69 | 1.59 | -1.23 |
| Ndr4 | Rattus norvegicus N-myc downstream regulated 4 | ***22*** | -2.02 | 2.04 | -1.69 |
|  |  |  |  |  |  |
| Scara3 | Rattus norvegicus scavenger receptor class A, member 3 | ***10*** | -2.61 | 0.19 | -2 |
| Cd14 | Rattus norvegicus CD14 antigen | ***10*** | -2.43 | -0.14 | -1.94 |
| LOC498674 | Rattus norvegicus LOC498674 | ***10*** | -2.29 | 0.25 | -2.29 |
| Prss35 | Rattus norvegicus protease, serine, 35 | ***10*** | -2.27 | -0.14 | -2.47 |
| Atp2a2 | Rattus norvegicus ATPase, Ca++ transporting, cardiac muscle, slow twitch 2 | ***10*** | -1.92 | -0.08 | -2.25 |
| Lphn1 | Rattus norvegicus latrophilin 1 | ***10*** | -1.71 | -0.09 | -2.09 |
| LOC360602 | Rattus norvegicus similar to cisplatin resistance-associated overexpressed protein | ***10*** | -1.76 | -0.18 | -1.86 |
| Srpr | Rattus norvegicus signal recognition particle receptor | ***10*** | -1.68 | 0.36 | -2.05 |
| Mgst2 | Rattus norvegicus microsomal glutathione S-transferase 2 | ***10*** | -1.61 | 0.66 | -2.32 |
| Stc1 | Rattus norvegicus stanniocalcin 1 | ***10*** | -1.66 | -0.01 | -3.09 |
| Atp1b2 | Rattus norvegicus ATPase, Na+/K+ transporting, beta 2 polypeptide | ***10*** | -1.37 | -0.01 | -2.96 |
| LOC499600 | Rattus norvegicus similar to 6030410K14Rik protein | ***10*** | -1.44 | -0.36 | -2.8 |
| Ahr | Rattus norvegicus aryl hydrocarbon receptor | ***10*** | -1.58 | 0.21 | -2.64 |
| Txnrd1 | Rattus norvegicus thioredoxin reductase 1 | ***10*** | -1.09 | 0.12 | -2.6 |
| Zcwcc1 | Rattus norvegicus zinc finger, CW-type with coiled-coil domain 1 | ***10*** | -1.65 | -0.45 | -2.35 |
| LOC306734 | Rattus norvegicus similar to RIKEN cDNA 4932432N11 gene | ***10*** | -1.36 | -0.3 | -2.34 |
| Ssx2ip | Rattus norvegicus synovial sarcoma, X breakpoint 2 interacting protein | ***10*** | -1.32 | -0.7 | -2.32 |
| Prickle1 | Rattus norvegicus prickle-like 1 | ***10*** | -1.17 | -0.72 | -2.28 |
| Baz2b | Rattus norvegicus bromodomain adjacent to zinc finger domain, 2B | ***10*** | -1.21 | -0.71 | -2.58 |
| Per2 | Rattus norvegicus period homolog 2 | ***10*** | -1.08 | -0.31 | -2.53 |
| Mpst | Rattus norvegicus mercaptopyruvate sulfurtransferase | ***10*** | -1.05 | -0.57 | -2.55 |
| Aox1 | Rattus norvegicus aldehyde oxidase 1 | ***10*** | -0.85 | -0.52 | -2.6 |
| LOC296469 | Rattus norvegicus similar to chromosome 20 open reading frame 58 | ***10*** | -1.59 | -0.36 | -1.63 |
| Snx14 | Rattus norvegicus sorting nexin 14 | ***10*** | -1.54 | -0.14 | -1.67 |
| Abca2 | Rattus norvegicus ATP-binding cassette, sub-family A | ***10*** | -1.37 | -0.18 | -1.61 |
| RGD1307700 | Rattus norvegicus similar to hypothetical protein BC018453 | ***10*** | -1.34 | -0.23 | -1.52 |
| Dpp3 | Rattus norvegicus dipeptidylpeptidase 3 | ***10*** | -1.33 | -0.31 | -1.51 |
| LOC303790 | Rattus norvegicus similar to RIKEN cDNA 4122402O22 | ***10*** | -1.42 | -0.37 | -1.83 |
| LOC500662 | Rattus norvegicus similar to Glutathione S-transferase 8 | ***10*** | -1.15 | -0.31 | -1.85 |
| Rnf166 | Rattus norvegicus ring finger protein 166 | ***10*** | -1.4 | -0.08 | -1.9 |
| Apoa1bp | Rattus norvegicus apolipoprotein A-I binding protein | ***10*** | -1.38 | 0.22 | -1.9 |
| Copz1 | Rattus norvegicus coatomer protein complex, subunit zeta 1 | ***10*** | -1.33 | 0.06 | -2.06 |
| Dad1 | Rattus norvegicus defender against cell death 1 | ***10*** | -1.24 | 0 | -1.98 |
| LOC360760 | Rattus norvegicus similar to OTTHUMP00000042400 | ***10*** | -1.09 | 0.04 | -2.09 |
| LOC502017 | Rattus norvegicus similar to Dolichol-phosphate mannosyltransferase subunit 3 | ***10*** | -1.54 | 0.27 | -1.48 |
| LOC299199 | Rattus norvegicus similar to YLP motif containing protein 1 | ***10*** | -1.39 | 0.25 | -1.56 |
| Rda279 | Rattus norvegicus hypothetical protein RDA279 | ***10*** | -1.45 | 0.14 | -1.67 |
| Loc65027 | Rattus norvegicus beta-catenin binding protein | ***10*** | -1.28 | -0.02 | -1.63 |
| Pcnxl3 | Rattus norvegicus pecanex-like 3 | ***10*** | -1.2 | 0 | -1.76 |
| LOC362840 | Rattus norvegicus LOC362840 | ***10*** | -1.15 | 0.14 | -1.61 |
| Ifi27l | Rattus norvegicus interferon, alpha-inducible protein 27-like | ***10*** | -1.11 | 0.05 | -1.65 |
| Itgb1bp1 | Rattus norvegicus integrin beta 1 binding protein 1 | ***10*** | -1.14 | 0.34 | -1.83 |
| Tep1 | Rattus norvegicus telomerase associated protein 1 | ***10*** | -0.97 | 0.33 | -1.84 |
| Qscn6 | Rattus norvegicus quiescin Q6 | ***10*** | -0.96 | 0.3 | -1.9 |
| MGC94053 | Rattus norvegicus similar to RECS1 | ***10*** | -1.01 | 0.42 | -1.52 |
| Usp7 | Rattus norvegicus ubiquitin specific protease 7 | ***10*** | -1.18 | -0.54 | -1.71 |
| Decr1 | Rattus norvegicus 2,4-dienoyl CoA reductase 1, mitochondrial | ***10*** | -1.1 | -0.57 | -1.72 |
| Txndc1 | Rattus norvegicus thioredoxin domain containing 1 | ***10*** | -1.03 | -0.47 | -1.71 |
| LOC56769 | Rattus norvegicus nuclear protein E3-3 | ***10*** | -0.92 | -0.53 | -1.84 |
| Mcm2 | Rattus norvegicus minichromosome maintenance deficient 2 mitotin | ***10*** | -0.9 | -0.36 | -1.76 |
| Akap12 | Rattus norvegicus A kinase | ***10*** | -0.88 | -0.37 | -1.81 |
| Ehmt1 | Rattus norvegicus euchromatic histone methyltransferase 1 | ***10*** | -0.82 | -0.44 | -1.76 |
| LOC315216 | Rattus norvegicus hypothetical LOC315216 | ***10*** | -0.88 | -0.59 | -1.64 |
| Ndufb7 | Rattus norvegicus NADH dehydrogenase | ***10*** | -0.83 | -0.6 | -1.67 |
| Mark3 | Rattus norvegicus MAP/microtubule affinity-regulating kinase 3 | ***10*** | -0.9 | -0.26 | -1.8 |
| Cpd | Rattus norvegicus carboxypeptidase D | ***10*** | -0.87 | -0.4 | -1.66 |
| MGC105797 | Rattus norvegicus similar to ubiquitously-expressed transcript isoform 1 | ***10*** | -0.74 | -0.33 | -1.66 |
| Ddost | Rattus norvegicus dolichyl-di-phosphooligosaccharide-protein glycotransferase | ***10*** | -0.87 | -0.26 | -1.56 |
| LOC499615 | Rattus norvegicus similar to RIKEN cDNA 2810489O06 | ***10*** | -0.72 | -0.26 | -1.54 |
| Ctbs | Rattus norvegicus chitobiase, di-N-acetyl- | ***10*** | -0.82 | 0.32 | -1.65 |
| LOC289437 | Rattus norvegicus similar to Glomulin | ***10*** | -0.61 | 0.32 | -1.66 |
| Klf4 | Rattus norvegicus Kruppel-like factor 4 | ***10*** | -0.41 | 0.39 | -1.52 |
| Acyp1 | Rattus norvegicus acylphosphatase 1, erythrocyte | ***10*** | -0.52 | -0.13 | -1.64 |
| LOC363474 | Rattus norvegicus similar to RIKEN cDNA 0610008C08 | ***10*** | -0.29 | -0.2 | -1.75 |
| Itga7 | Rattus norvegicus integrin alpha 7 | ***10*** | -0.4 | 0.04 | -1.83 |
| Psmd13 | Rattus norvegicus proteasome | ***10*** | -0.48 | 0.14 | -1.52 |
| Mcm3ap | Rattus norvegicus minichromosome maintenance deficient 3 | ***10*** | -0.41 | 0 | -1.54 |
| Cd44 | Rattus norvegicus CD44 antigen | ***10*** | -0.04 | 0.45 | -1.56 |
| Tctex1 | Rattus norvegicus t-complex testis expressed 1 | ***10*** | 0.02 | 0.31 | -1.7 |
| Cirh1a | Rattus norvegicus cirrhosis, autosomal recessive 1A | ***10*** | -0.02 | 0.08 | -1.97 |
| LOC498388 | Rattus norvegicus similar to High mobility group protein 2 | ***10*** | -0.01 | 0.03 | -1.6 |
| LOC500504 | Rattus norvegicus LOC500504 | ***10*** | 0 | 0.09 | -1.58 |
| LOC309953 | Rattus norvegicus similar to hypothetical protein MGC33214 | ***10*** | -0.74 | -0.03 | -1.91 |
| Rab6ip1 | Rattus norvegicus Rab6 interacting protein 1 | ***10*** | -0.57 | -0.04 | -2.11 |
| MGC94736 | Rattus norvegicus similar to hypothetical protein MGC35097 | ***10*** | -0.55 | -0.13 | -1.95 |
| 1200013b22rik | Rattus norvegicus SNF1/AMP-activated protein kinase | ***10*** | -0.54 | -0.24 | -2.1 |
| RGD1305132 | Rattus norvegicus similar to RIKEN cDNA A630065K24 | ***10*** | -0.32 | -0.27 | -2.07 |
| Msn | Rattus norvegicus moesin | ***10*** | -0.17 | -0.27 | -1.95 |
| Arl6ip2 | Rattus norvegicus ADP-ribosylation factor-like 6 interacting protein 2 | ***10*** | -0.17 | -0.12 | -2.19 |
| Tnk2 | Rattus norvegicus tyrosine kinase, non-receptor, 2 | ***10*** | 0.02 | -0.23 | -2.09 |
| LOC305452 | Rattus norvegicus hypothetical LOC305452 | ***10*** | -0.51 | -0.37 | -1.58 |
| Zfp330 | Rattus norvegicus zinc finger protein 330 | ***10*** | -0.49 | -0.52 | -1.81 |
| LOC362490 | Rattus norvegicus similar to RIKEN cDNA 2610319K07 | ***10*** | -0.3 | -0.56 | -1.71 |
| Nisch | Rattus norvegicus nischarin | ***10*** | -0.25 | -0.29 | -1.56 |
| Snrpd2 | Rattus norvegicus small nuclear ribonucleoprotein D2 | ***10*** | -0.04 | -0.18 | -1.56 |
| RT1-A1 | Rattus norvegicus RT1 class Ia, locus A1 | ***10*** | -0.03 | -0.56 | -1.62 |
| Fam38a | Rattus norvegicus family with sequence similarity 38, member A | ***10*** | -0.12 | -0.35 | -1.76 |
| Mettl3 | Rattus norvegicus methyltransferase-like 3 | ***10*** | 0.02 | -0.38 | -1.84 |
| MGC105508 | Rattus norvegicus similar to chromosome 6 open reading frame 83; similar to RIKEN cDNA 5630401J11 | ***10*** | 0.06 | -0.18 | -1.83 |
| Eif4g3 | Rattus norvegicus eukaryotic translation initiation factor 4 gamma, 3 | ***10*** | -0.86 | 0.73 | -2.13 |
| Catns | Rattus norvegicus catenin src | ***10*** | -0.76 | 0.92 | -1.67 |
| RGD1308384 | Rattus norvegicus similar to RIKEN cDNA 6330415M09 | ***10*** | -0.43 | 0.58 | -1.9 |
| Ndufb3 | Rattus norvegicus NADH dehydrogenase | ***10*** | -0.24 | 0.64 | -2.02 |
| Frg1 | Rattus norvegicus FSHD region gene 1 | ***10*** | -0.29 | 0.85 | -1.74 |
| Npr2 | Rattus norvegicus natriuretic peptide receptor 2 | ***10*** | -0.18 | 0.65 | -1.8 |
| Hmgn3 | Rattus norvegicus high mobility group nucleosomal binding domain 3 | ***10*** | -2.09 | 1.31 | -4.15 |
| Srrm2 | Rattus norvegicus serine/arginine repetitive matrix 2 | ***10*** | -1.73 | 0.34 | -4.43 |
|  |  |  |  |  |  |
| LOC362587 | Rattus norvegicus similar to microfilament and actin filament cross-linker protein isoform a | ***35*** | -0.68 | 0.15 | -3.37 |
| isg12(b) | Rattus norvegicus putative ISG12 | ***35*** | -0.43 | 0.12 | -3.21 |
| LOC500398 | Rattus norvegicus similar to LRRGT00082 | ***35*** | 0.22 | 0.48 | -3.7 |
| Fbxl20 | Rattus norvegicus F-box and leucine-rich repeat protein 20 | ***35*** | -0.36 | 0.61 | -2.76 |
| Srp72 | Rattus norvegicus signal recognition particle 72 | ***35*** | -0.1 | 0.43 | -2.41 |
| LOC499560 | Rattus norvegicus similar to LRRG00135 | ***35*** | -0.07 | 0.76 | -2.33 |
| LOC501562 | Rattus norvegicus similar to ORF2 consensus sequence encoding endonuclease and reverse transcriptase minus RNaseH | ***35*** | 0.25 | 1.2 | -2.5 |
| LOC501553 | Rattus norvegicus similar to LRRGT00078 | ***35*** | 0.25 | 1.07 | -2.87 |
| LOC501087 | Rattus norvegicus similar to LRRGT00057 | ***35*** | 0.32 | 1.17 | -3.25 |
| Camk2g | Rattus norvegicus calcium/calmodulin-dependent protein kinase II gamma | ***35*** | 0.14 | 0.48 | -2.53 |
| LOC500916 | Rattus norvegicus similar to LRRGT00176 | ***35*** | 0.3 | 0.42 | -2.67 |
| Zc3hdc7 | Rattus norvegicus zinc finger CCCH type domain containing 7 | ***35*** | 0.4 | 0.35 | -2.3 |
| Tial1 | Rattus norvegicus Tial1 cytotoxic granule-associated RNA binding protein-like 1 | ***35*** | 0.59 | 0.37 | -2.45 |
| RT1-149 | Rattus norvegicus RT1 class I, T24, gene 4 | ***35*** | 0.73 | 0.17 | -2.77 |
| LOC498076 | Rattus norvegicus similar to RIKEN cDNA 2410116I05 | ***35*** | 0.77 | -0.02 | -2.89 |
| LOC498623 | Rattus norvegicus similar to LRRGT00176 | ***35*** | 0.94 | 0.51 | -2.76 |
| Gstm1 | Rattus norvegicus glutathione S-transferase, mu 1 | ***35*** | 1 | 0.21 | -2.54 |
| LOC501637 | Rattus norvegicus similar to LRRG00135 | ***35*** | 1.08 | 0.21 | -2.41 |
| LOC501548 | Rattus norvegicus similar to LRRG00135 | ***35*** | 1.14 | 0.28 | -2.18 |
| LOC498245 | Rattus norvegicus similar to LRRGT00176 | ***35*** | 1.07 | -0.28 | -2.19 |
| Crim1 | Rattus norvegicus cysteine-rich motor neuron 1 | ***35*** | 1.26 | -0.15 | -2.36 |
| LOC362543 | Rattus norvegicus similar to LRRG00116 | ***35*** | 0.8 | 1.06 | -4.37 |
| LOC362315 | Rattus norvegicus similar to Retrovirus-related POL polyprotein | ***35*** | 1 | 1.09 | -3.55 |
| LOC500960 | Rattus norvegicus similar to Da1-12 | ***35*** | 1.07 | 0.67 | -3.39 |
| LOC361942 | Rattus norvegicus similar to ORF4 | ***35*** | 1.2 | 1.32 | -3.07 |
| Per1 | Rattus norvegicus period homolog 1 | ***35*** | 0.95 | -0.88 | -4.09 |
|  |  |  |  |  |  |
| Gstp1 | Rattus norvegicus glutathione-S-transferase, pi 1 | ***18*** | -1.71 | 4.05 | -3.35 |
|  |  |  |  |  |  |
| Sesn1 | Rattus norvegicus sestrin 1 | ***7*** | -3.5 | -2.94 | -1.44 |
| Alcam | Rattus norvegicus activated leukocyte cell adhesion molecule | ***7*** | -3.45 | -2.37 | -1.11 |
| LOC498356 | Rattus norvegicus similar to MGC68837 protein | ***7*** | -2.82 | -3.22 | -2.1 |
| Nrp1 | Rattus norvegicus neuropilin 1 | ***7*** | -2.31 | -2.46 | -2.01 |
| LOC317575 | Rattus norvegicus similar to Smarca1 protein | ***7*** | -2.12 | -2.99 | -2.53 |
| Gpr37l1 | Rattus norvegicus G protein-coupled receptor 37-like 1 | ***7*** | -2.1 | -2.85 | -2.3 |
| Gpr51 | Rattus norvegicus G protein-coupled receptor 51 | ***7*** | -2.54 | -3.19 | -1.38 |
| LOC499856 | Rattus norvegicus similar to RIKEN cDNA 1110018M03 | ***7*** | -2.32 | -2.85 | -1.59 |
| Efemp1 | Rattus norvegicus epidermal growth factor-containing fibulin-like extracellular matrix protein 1 | ***7*** | -2.16 | -3.39 | -1.3 |
| Giot1 | Rattus norvegicus gonadotropin inducible ovarian transcription factor 1 | ***7*** | -1.99 | -3.42 | -1.57 |
| Olig1 | Rattus norvegicus oligodendrocyte transcription factor 1 | ***7*** | -2.1 | -3.92 | -1.82 |
| LOC290372 | Rattus norvegicus similar to expressed sequence AU021034 | ***7*** | -1.51 | -4.62 | -1.45 |
| Csad | Rattus norvegicus cysteine sulfinic acid decarboxylase | ***7*** | -1.68 | -3.05 | -1.61 |
| Egr2 | Rattus norvegicus early growth response 2 | ***7*** | -1.1 | -3.01 | -1.32 |
| Prelp | Rattus norvegicus proline arginine-rich end leucine-rich repeat protein | ***7*** | -1.63 | -3.42 | -2.16 |
| LOC500939 | Rattus norvegicus LOC500939 | ***7*** | -1.23 | -3.4 | -2.24 |
| Dncl2b | Rattus norvegicus dynein, cytoplasmic, light chain 2B | ***7*** | -0.67 | -3.19 | -2.04 |
|  |  |  |  |  |  |
| RGD1311364 | Rattus norvegicus similar to RIKEN cDNA 1810021J13 | ***36*** | -1.49 | -2.18 | -0.97 |
| Znf386 | Rattus norvegicus zinc finger protein 386 | ***36*** | -1.35 | -2.07 | -1.17 |
| Cdo1 | Rattus norvegicus cysteine dioxygenase 1, cytosolic | ***36*** | -1.3 | -2.31 | -1.19 |
| Gpc3 | Rattus norvegicus glypican 3 | ***36*** | -1.18 | -2.33 | -1.18 |
| Psip1 | Rattus norvegicus PC4 and SFRS1 interacting protein 1 | ***36*** | -1.2 | -2.1 | -1.29 |
| LOC304289 | Rattus norvegicus similar to KCCR13L | ***36*** | -1.14 | -2.19 | -1.3 |
| Stk39 | Rattus norvegicus serine/threonine kinase 39, STE20/SPS1 homolog | ***36*** | -1.32 | -2.38 | -0.85 |
| Fxna | Rattus norvegicus putative aminopeptidase Fxna | ***36*** | -1.3 | -2.47 | -0.67 |
| Rarres1 | Rattus norvegicus retinoic acid receptor responder | ***36*** | -1.22 | -2.16 | -0.84 |
| LOC500536 | Rattus norvegicus similar to novel protein | ***36*** | -1.1 | -2.07 | -0.79 |
| LOC366872 | Rattus norvegicus similar to RIKEN cDNA 4921537D05 | ***36*** | -0.96 | -2.63 | -0.9 |
| C4-2 | Rattus norvegicus complement component 4, gene 2 | ***36*** | -0.83 | -2.49 | -0.74 |
| LOC365214 | Rattus norvegicus similar to zinc finger protein ZFP235 | ***36*** | -0.88 | -1.93 | -0.68 |
| Anxa3 | Rattus norvegicus annexin A3 | ***36*** | -0.86 | -2 | -0.76 |
| LOC291354 | Rattus norvegicus similar to hypothetical protein MGC26778 | ***36*** | -0.7 | -2.05 | -0.97 |
| MGC105961 | Rattus norvegicus similar to mitochondrial ribosomal protein L13 | ***36*** | -0.69 | -1.89 | -0.84 |
| Rnpep | Rattus norvegicus arginyl aminopeptidase | ***36*** | -0.42 | -2.19 | -0.72 |
| Gpm6a | Rattus norvegicus glycoprotein m6a | ***36*** | -0.35 | -2.22 | -0.66 |
| Sirt2 | Rattus norvegicus sirtuin | ***36*** | -0.33 | -2.19 | -0.58 |
| Rpl15 | Rattus norvegicus ribosomal protein L15 | ***36*** | -0.81 | -2.41 | -0.96 |
| Aqp4 | Rattus norvegicus aquaporin 4 | ***36*** | -0.74 | -2.28 | -1.02 |
| Sil1 | Rattus norvegicus endoplasmic reticulum chaperone SIL1 homolog | ***36*** | -0.63 | -2.49 | -1.21 |
| Plekhb1 | Rattus norvegicus evectin-1 | ***36*** | -0.47 | -2.29 | -1.19 |
| LOC303238 | Rattus norvegicus similar to novel protein | ***36*** | -0.41 | -2.27 | -0.98 |
| Scg3 | Rattus norvegicus secretogranin III | ***36*** | -1.28 | -2.35 | -0.26 |
| Sdccag3 | Rattus norvegicus serologically defined colon cancer antigen 3 | ***36*** | -1.13 | -2.12 | -0.51 |
| Asrgl1 | Rattus norvegicus asparaginase-like sperm autoantigen | ***36*** | -1.04 | -2.1 | -0.44 |
| Gmnn | Rattus norvegicus geminin | ***36*** | -1.08 | -2.65 | -0.45 |
| LOC365389 | Rattus norvegicus similar to RIKEN cDNA 5730427C23 | ***36*** | -0.95 | -2.63 | -0.51 |
| Epb4.1l3 | Rattus norvegicus erythrocyte protein band 4.1-like 3 | ***36*** | -1.03 | -2.61 | -0.31 |
| Pdgfa | Rattus norvegicus platelet derived growth factor, alpha | ***36*** | -0.74 | -2.1 | -0.27 |
| Glrx2 | Rattus norvegicus glutaredoxin 2 | ***36*** | -0.6 | -2.27 | -0.35 |
| LOC317396 | Rattus norvegicus similar to Ubiquilin 2 | ***36*** | -0.4 | -2.15 | -0.34 |
| LOC360819 | Rattus norvegicus similar to FLJ00052 protein | ***36*** | -0.6 | -2.34 | 0.04 |
| Mpp6 | Rattus norvegicus membrane protein, palmitoylated 6 | ***36*** | -0.54 | -2.24 | -0.1 |
| Pc | Rattus norvegicus Pyruvate carboxylase | ***36*** | -0.99 | -1.57 | -0.7 |
| LOC311796 | Rattus norvegicus similar to cofactor of BRCA1; negative elongation factor protein B | ***36*** | -0.78 | -1.59 | -0.82 |
| Gabarapl2 | Rattus norvegicus GABA | ***36*** | -0.74 | -1.69 | -0.7 |
| LOC314949 | Rattus norvegicus similar to HR21spA | ***36*** | -0.67 | -1.58 | -0.43 |
| LOC362414 | Rattus norvegicus similar to Tada3l protein | ***36*** | -0.6 | -1.62 | -0.5 |
| Mtpn | Rattus norvegicus myotrophin | ***36*** | -0.42 | -1.54 | -0.6 |
| LOC313445 | Rattus norvegicus similar to kelch-like 13 | ***36*** | -0.92 | -1.8 | -0.49 |
| LOC501341 | Rattus norvegicus similar to glutamate receptor, ionotropic, N-methyl D-aspartate-like 1A | ***36*** | -0.88 | -1.88 | -0.4 |
| Nphp1 | Rattus norvegicus nephronophthisis 1 | ***36*** | -0.86 | -1.86 | -0.38 |
| Gclm | Rattus norvegicus glutamate cysteine ligase, modifier subunit | ***36*** | -0.67 | -1.87 | -0.23 |
| LOC360478 | Rattus norvegicus hypothetical LOC360478 | ***36*** | -0.57 | -1.9 | -0.25 |
| Adk | Rattus norvegicus adenosine kinase | ***36*** | -0.55 | -1.74 | -0.34 |
| LOC289900 | Rattus norvegicus hypothetical LOC289900 | ***36*** | -0.86 | -1.74 | -0.15 |
| LOC310946 | Rattus norvegicus similar to hypothetical protein FLJ20331 | ***36*** | -0.81 | -1.73 | -0.26 |
| Ppid | Rattus norvegicus peptidylprolyl isomerase D | ***36*** | -0.69 | -1.54 | -0.08 |
| RGD1308463 | Rattus norvegicus similar to IMP4 | ***36*** | -0.17 | -1.99 | -0.36 |
| LOC313842 | Rattus norvegicus similar to 2810036L13Rik protein | ***36*** | -0.12 | -2.21 | -0.19 |
| Cds1 | Rattus norvegicus CDP-diacylglycerol synthase 1 | ***36*** | -0.02 | -2.11 | -0.01 |
| LOC497844 | Rattus norvegicus hypothetical gene supported by NM_138846 | ***36*** | 0.07 | -2.03 | -0.18 |
| Yme1l1 | Rattus norvegicus YME1-like 1 | ***36*** | 0.17 | -2.16 | -0.51 |
| LOC290851 | Rattus norvegicus similar to RIKEN cDNA 2210415M20 | ***36*** | 0.36 | -2.21 | -0.55 |
| LOC361309 | Rattus norvegicus similar to polyadenylate-binding protein-interacting protein 2 | ***36*** | 0.3 | -1.81 | -0.59 |
| Capzb | Rattus norvegicus F-actin capping protein beta subunit | ***36*** | 0.4 | -1.86 | -0.4 |
| Rhpn1 | Rattus norvegicus rhophilin, Rho GTPase binding protein 1 | ***36*** | -0.16 | -2.67 | -0.52 |
| LOC317444 | Rattus norvegicus similar to Hccs protein | ***36*** | -0.06 | -2.53 | -0.31 |
| LOC503409 | Rattus norvegicus similar to Ac1147 | ***36*** | 0.28 | -2.61 | -0.68 |
| Igbp1 | Rattus norvegicus immunoglobulin | ***36*** | 0.31 | -2.54 | -0.46 |
| Parva | Rattus norvegicus parvin, alpha | ***36*** | 0.24 | -2.36 | -0.38 |
| LOC498278 | Rattus norvegicus similar to RIKEN cDNA 1700009P17 | ***36*** | 0.38 | -2.34 | -0.26 |
| Loxl2 | Rattus norvegicus lysyl oxidase-like 2 | ***36*** | 0.6 | -2.39 | -0.21 |
| LOC498228 | Rattus norvegicus similar to hypothetical protein DKFZp761N1114 | ***36*** | -0.93 | -1.27 | -1.74 |
| LOC316228 | Rattus norvegicus similar to p53-associated parkin-like cytoplasmic protein | ***36*** | -0.92 | -1.46 | -1.85 |
| Cln2 | Rattus norvegicus ceroid-lipofuscinosis, neuronal 2 | ***36*** | -0.78 | -1.39 | -1.7 |
| MGC94954 | Rattus norvegicus similar to RIKEN cDNA 2310042P20 | ***36*** | -0.6 | -1.34 | -1.65 |
| LOC287452 | Rattus norvegicus similar to RIKEN cDNA 1110020A23 | ***36*** | -0.77 | -1.44 | -1.52 |
| RT1-A2 | Rattus norvegicus RT1 class Ia, locus A2 | ***36*** | -0.61 | -1.36 | -1.9 |
| LOC499094 | Rattus norvegicus similar to zinc finger protein 61 | ***36*** | -0.49 | -1.63 | -1.89 |
| Lrp16 | Rattus norvegicus LRP16 protein | ***36*** | -0.28 | -1.34 | -1.62 |
| LOC362580 | Rattus norvegicus similar to CG2919-PA | ***36*** | -0.57 | -1.79 | -1.49 |
| Cpe | Rattus norvegicus carboxypeptidase E | ***36*** | -0.54 | -1.67 | -1.27 |
| LOC296126 | Rattus norvegicus similar to U5 snRNP-specific protein, 200 kDa | ***36*** | -0.47 | -1.56 | -1.31 |
| Krt2-8 | Rattus norvegicus keratin complex 2, basic, gene 8 | ***36*** | -0.33 | -1.88 | -1.28 |
| Sparcl1 | Rattus norvegicus SPARC-like 1 | ***36*** | -0.28 | -1.91 | -1.83 |
| LOC502614 | Rattus norvegicus similar to RIKEN cDNA 2610205E22 | ***36*** | -0.14 | -1.87 | -1.73 |
| Cox17 | Rattus norvegicus cytochrome c oxidase, subunit XVII assembly protein homolog | ***36*** | -0.09 | -1.69 | -1.59 |
| Kifap3 | Rattus norvegicus kinesin-associated protein 3 | ***36*** | 0.12 | -1.76 | -1.47 |
| Ndrg2 | Rattus norvegicus N-myc downstream regulated gene 2 | ***36*** | 0.24 | -1.28 | -1.5 |
| LOC499323 | Rattus norvegicus similar to CCTeta, eta subunit of the chaperonin containing TCP-1 | ***36*** | 0.29 | -1.32 | -1.53 |
| B3gnt1 | Rattus norvegicus UDP-GlcNAc:betaGal beta-1,3-N-acetylglucosaminyltransferase 1 | ***36*** | 0 | -1.37 | -1.81 |
| Sorl1 | Rattus norvegicus sortilin-related receptor, L | ***36*** | 0.14 | -1.54 | -1.84 |
| LOC314859 | Rattus norvegicus similar to transformed mouse 3T3 cell double minute 1 | ***36*** | 0.21 | -1.37 | -2.06 |
| LOC498072 | Rattus norvegicus similar to High mobility group protein 2 | ***36*** | 0.26 | -1.56 | -2.03 |
| Eml2 | Rattus norvegicus echinoderm microtubule associated protein like 2 | ***36*** | -0.53 | -1.58 | -0.97 |
| RGD1309676 | Rattus norvegicus similar to RIKEN cDNA 5730469M10 | ***36*** | -0.31 | -1.81 | -0.98 |
| LOC306137 | Rattus norvegicus similar to CG10084-PA | ***36*** | -0.04 | -1.61 | -1.01 |
| Hexa | Rattus norvegicus hexosaminidase A | ***36*** | 0 | -1.69 | -1 |
| Capza2 | Rattus norvegicus capping protein | ***36*** | -0.02 | -1.51 | -1.29 |
| LOC308820 | Rattus norvegicus similar to RIKEN cDNA 2310015N07 | ***36*** | -0.23 | -2.16 | -1.15 |
| LOC299209 | Rattus norvegicus similar to 1700019E19Rik protein | ***36*** | -0.1 | -2.18 | -1.29 |
| Sema3b | Rattus norvegicus sema domain, immunoglobulin domain | ***36*** | 0.16 | -2.27 | -1.17 |
| Tf | Rattus norvegicus Transferrin | ***36*** | 0.17 | -2.12 | -1.57 |
| LOC499328 | Rattus norvegicus similar to riboflavin kinase | ***36*** | 0.49 | -2.01 | -1.31 |
| LOC301521 | Rattus norvegicus similar to hypothetical protein DKFZp434O0527 | ***36*** | 0.4 | -2.47 | -1.08 |
| Sdccag8 | Rattus norvegicus slinky | ***36*** | 0.55 | -2.26 | -1.01 |
| LOC292477 | Rattus norvegicus similar to OTTHUMP00000040155 | ***36*** | 0.06 | -2.58 | -1.9 |
| Pcm1 | Rattus norvegicus pericentriolar material 1 | ***36*** | 0.62 | -2.77 | -1.49 |
| Dnah1 | Rattus norvegicus dynein, axonemal, heavy polypeptide 1 | ***36*** | 0.31 | -2.88 | -2.78 |
| Fank1 | Rattus norvegicus fibronectin type 3 and ankyrin repeat domains 1 | ***36*** | 0.47 | -3.73 | -2.11 |
|  |  |  |  |  |  |
| Aard | Rattus norvegicus alanine and arginine rich domain containing protein | ***25*** | -1.34 | -2.35 | 0.8 |
| RGD1311155 | Rattus norvegicus similar to RIKEN cDNA 9230117N10 | ***25*** | -1.31 | -2.37 | 0.67 |
| Exosc3 | Rattus norvegicus exosome component 3 | ***25*** | -0.95 | -2.02 | 0.45 |
| Snx25 | Rattus norvegicus sorting nexin 25 | ***25*** | -0.78 | -2.15 | 0.44 |
| Hnrpm | Rattus norvegicus heterogeneous nuclear ribonucleoprotein M | ***25*** | -0.76 | -2.04 | 0.19 |
| Omd | Rattus norvegicus osteomodulin | ***25*** | -0.63 | -2.28 | 0.36 |
| Camk2n1 | Rattus norvegicus calcium/calmodulin-dependent protein kinase II inhibitor 1 | ***25*** | -0.59 | -2.4 | 0.46 |
| Smarca5 | Rattus norvegicus SWI/SNF related, matrix associated, actin dependent regulator of chromatin, subfamily a, member 5 | ***25*** | -1.14 | -1.62 | 0.8 |
| Kif20a | Rattus norvegicus kinesin family member 20A | ***25*** | -0.76 | -1.58 | 0.87 |
| Ggcx | Rattus norvegicus gamma-glutamyl carboxylase | ***25*** | -0.67 | -1.91 | 1.21 |
| Ascl1 | Rattus norvegicus achaete-scute complex homolog-like 1 | ***25*** | -0.54 | -2.04 | 0.96 |
| RGD1305524 | Rattus norvegicus similar to hypothetical protein FLJ12442 | ***25*** | -0.59 | -2.62 | 0.75 |
| Erp29 | Rattus norvegicus endoplasmic retuclum protein 29 | ***25*** | -0.46 | -2.56 | 1.25 |
| RGD1308075 | Rattus norvegicus similar to hypothetical protein MGC27019 | ***25*** | -0.5 | -3.14 | 1.24 |
| Adss2 | Rattus norvegicus adenylosuccinate synthetase 2, non muscle | ***25*** | -0.89 | -1.87 | 0.25 |
| Kpna1 | Rattus norvegicus karyopherin | ***25*** | -0.89 | -1.68 | 0.27 |
| LOC301563 | Rattus norvegicus similar to RIKEN cDNA 5230400G24 | ***25*** | -0.82 | -1.72 | 0.19 |
| Nubp1 | Rattus norvegicus nucleotide binding protein 1 | ***25*** | -0.71 | -1.67 | 0.33 |
| LOC361519 | Rattus norvegicus similar to hypothetical protein MGC51082 | ***25*** | -0.63 | -1.75 | 0.22 |
| Fchsd2 | Rattus norvegicus FCH and double SH3 domains 2 | ***25*** | -0.85 | -1.53 | 0.2 |
| Nfia | Rattus norvegicus nuclear factor I/A | ***25*** | -0.73 | -1.51 | 0.08 |
| Farslb | Rattus norvegicus phenylalanine-tRNA synthetase-like, beta subunit | ***25*** | -0.72 | -1.75 | 0.07 |
| Casp7 | Rattus norvegicus caspase 7 | ***25*** | -0.49 | -1.71 | 0.12 |
| LOC315645 | Rattus norvegicus similar to RIKEN cDNA 2700059L22 | ***25*** | -0.79 | -1.57 | 0.45 |
| RGD735029 | Rattus norvegicus SEL1 domain containing protein RGD735029 | ***25*** | -0.65 | -1.52 | 0.56 |
| Rbm10 | Rattus norvegicus RNA binding motif protein 10 | ***25*** | -0.47 | -1.59 | 0.37 |
| Mlc1 | Rattus norvegicus megalencephalic leukoencephalopathy with subcortical cysts 1 | ***25*** | -0.29 | -1.69 | 0.48 |
| LOC292486 | Rattus norvegicus similar to Aig1 protein | ***25*** | -0.59 | -1.85 | 0.78 |
| Hes6 | Rattus norvegicus hairy and enhancer of split 6 | ***25*** | -0.57 | -1.82 | 0.6 |
| Tyki | Rattus norvegicus thymidylate kinase family LPS-inducible member | ***25*** | -0.48 | -1.53 | -0.01 |
| Tufm | Rattus norvegicus Tu translation elongation factor, mitochondrial | ***25*** | -0.43 | -1.52 | 0.13 |
| RGD1303232 | Rattus norvegicus Phytn_dehydro and Pyr_redox domain containing protein RGD1303232 | ***25*** | -0.29 | -1.57 | 0.03 |
| Rpia | Rattus norvegicus ribose 5-phosphate isomerase A | ***25*** | -0.38 | -1.63 | -0.17 |
| Anxa5 | Rattus norvegicus annexin A5 | ***25*** | 0.06 | -1.66 | 0.18 |
| Cfdp1 | Rattus norvegicus craniofacial development protein 1 | ***25*** | 0.17 | -1.73 | 0.06 |
| Phyhd1 | Rattus norvegicus phytanoyl-CoA dioxygenase domain containing 1 | ***25*** | 0.1 | -1.6 | -0.2 |
| Fbln1 | Rattus norvegicus fibulin 1 | ***25*** | -0.01 | -2.03 | 0.38 |
| Slc15a4 | Rattus norvegicus peptide/histidine transporter | ***25*** | 0.03 | -2.14 | 0.3 |
| LOC304332 | Rattus norvegicus similar to hypothetical protein FLJ20397 | ***25*** | 0.21 | -1.84 | 0.59 |
| Kpnb3 | Rattus norvegicus karyopherin | ***25*** | 0.47 | -1.86 | 0.45 |
| RGD1305061 | Rattus norvegicus similar to RIKEN cDNA 2700055K07 | ***25*** | 0.26 | -2.14 | 0.71 |
| MGC93902 | Rattus norvegicus similar to RIKEN cDNA 2610028I09 | ***25*** | 0.6 | -2.4 | 0.21 |
| Galm | Rattus norvegicus galactose mutarotase | ***25*** | 0.67 | -2.04 | 0.35 |
| Csda | Rattus norvegicus cold shock domain protein A | ***25*** | 0.87 | -2.03 | 0.64 |
| Ivns1abp | Rattus norvegicus influenza virus NS1A binding protein | ***25*** | 0.02 | -1.77 | 0.95 |
| Atp5c1 | Rattus norvegicus ATP synthase, H+ transporting, mitochondrial F1 complex, gamma polypeptide 1 | ***25*** | 0.15 | -1.56 | 0.91 |
| MGC72987 | Rattus norvegicus Unknown | ***25*** | 0.49 | -1.52 | 1.11 |
|  |  |  |  |  |  |
| Calb1 | Rattus norvegicus calbindin 1 | ***34*** | -1.3 | -4.65 | -0.25 |
| LOC293156 | Rattus norvegicus similar to Hypothetical 55.1 kDa protein F09G8.5 in chromosome III | ***34*** | -0.98 | -4.01 | -0.41 |
| Fez1 | Rattus norvegicus fasciculation and elongation protein zeta 1 | ***34*** | -0.25 | -4.18 | -0.69 |
| LOC498982 | Rattus norvegicus similar to Myb proto-oncogene protein | ***34*** | 0.1 | -4.62 | -0.32 |
| LOC310926 | Rattus norvegicus similar to Ac1147 | ***34*** | 0.33 | -5.15 | -0.65 |
| LOC296608 | Rattus norvegicus similar to hypothetical protein MGC29761 | ***34*** | -1.28 | -3.36 | -0.98 |
| Itgb4 | Rattus norvegicus integrin beta 4 | ***34*** | -1.22 | -3.56 | -0.62 |
| Arhgdig | Rattus norvegicus Rho GDP dissociation inhibitor | ***34*** | -0.59 | -3.89 | -1.39 |
| Dnaja4 | Rattus norvegicus DnaJ | ***34*** | -0.54 | -3.55 | -1.26 |
| Igsf1 | Rattus norvegicus immunoglobulin superfamily, member 1 | ***34*** | -0.8 | -2.95 | -1.06 |
| Dnai2 | Rattus norvegicus dynein, axonemal, intermediate polypeptide 2 | ***34*** | -0.77 | -2.82 | -0.83 |
| Strbp | Rattus norvegicus double-stranded RNA-binding protein p74 | ***34*** | -0.23 | -2.99 | -0.87 |
| Tekt1 | Rattus norvegicus tektin 1 | ***34*** | -0.56 | -3.1 | -1.46 |
| Spag8 | Rattus norvegicus sperm associated antigen 8 | ***34*** | -0.35 | -2.77 | -1.34 |
| Khdrbs3 | Rattus norvegicus etoile, Sam68-like protein SLM-2 | ***34*** | -1.17 | -3.55 | 0.23 |
| Sez6 | Rattus norvegicus seizure related 6 homolog | ***34*** | -0.96 | -3.18 | 0.21 |
| LOC362809 | Rattus norvegicus similar to Sid3177p | ***34*** | -0.82 | -3.23 | 0.35 |
| LOC313436 | Rattus norvegicus similar to RIKEN cDNA 2810028A01 | ***34*** | -0.99 | -3.14 | -0.52 |
| LOC500416 | Rattus norvegicus LOC500416 | ***34*** | -0.67 | -3.36 | -0.13 |
| LOC287346 | Rattus norvegicus similar to novel protein | ***34*** | -0.53 | -3.46 | -0.56 |
| Aurkb | Rattus norvegicus aurora kinase B | ***34*** | -0.44 | -3.46 | -0.53 |
| Mlf1 | Rattus norvegicus myeloid leukemia factor 1 | ***34*** | -0.22 | -3.36 | -0.76 |
| LOC499268 | Rattus norvegicus similar to Gm166 protein | ***34*** | 0.09 | -3.12 | 0.37 |
| LOC300517 | Rattus norvegicus similar to hypothetical protein FLJ25530 | ***34*** | 0.5 | -3.11 | 0.31 |
| LOC291847 | Rattus norvegicus similar to hypothetical protein 4933409I22 | ***34*** | 0.36 | -3.81 | 0.75 |
| Lrpb7 | Rattus norvegicus leucine rich protein, B7 gene | ***34*** | 0.72 | -3.36 | 0.74 |
| MGC105647 | Rattus norvegicus similar to Nur77 downstream protein 2 | ***34*** | 1.33 | -3.81 | 0.53 |
| Zmynd10 | Rattus norvegicus zinc finger, MYND domain-containing 10 | ***34*** | 0.44 | -2.9 | 0 |
| LOC503278 | Rattus norvegicus similar to testin | ***34*** | 0.69 | -2.99 | -0.03 |
| LOC499839 | Rattus norvegicus similar to LOC387763 protein | ***34*** | 1.07 | -3.1 | -0.28 |
|  |  |  |  |  |  |
| Nup54 | Rattus norvegicus nucleoporin 54 | ***13*** | 0.15 | -0.89 | -1.7 |
| Tmp21 | Rattus norvegicus transmembrane trafficking protein 21 | ***13*** | 0.19 | -0.84 | -1.71 |
| RGD1310143 | Rattus norvegicus similar to RIKEN cDNA D030028O16 | ***13*** | 0.18 | -0.92 | -1.5 |
| Tex27 | Rattus norvegicus testis expressed gene 27 | ***13*** | 0.31 | -0.7 | -1.66 |
| LOC364468 | Rattus norvegicus similar to TGF beta-inducible nuclear protein 1 | ***13*** | 0.45 | -0.41 | -1.57 |
| Magi3 | Rattus norvegicus membrane-associated guanylate kinase-related | ***13*** | 0.66 | -0.8 | -1.83 |
| LOC498276 | Rattus norvegicus similar to Fc gamma | ***13*** | 0.8 | -0.9 | -1.56 |
| LOC361774 | Rattus norvegicus LOC361774 | ***13*** | 0.87 | -1.14 | -1.74 |
| Nfix | Rattus norvegicus nuclear factor I/X | ***13*** | 0.31 | -0.89 | -2.03 |
| LOC305502 | Rattus norvegicus similar to RIKEN cDNA 1110014L17 | ***13*** | 0.33 | -1 | -2 |
| Znrd1 | Rattus norvegicus zinc ribbon domain containing, 1 | ***13*** | 0.31 | -0.73 | -2.13 |
| LOC498378 | Rattus norvegicus similar to LRRGT00176 | ***13*** | 0.4 | -0.79 | -1.96 |
| Hnrph3 | Rattus norvegicus heterogeneous nuclear ribonucleoprotein H3 | ***13*** | 0.48 | -1.19 | -2.02 |
| Lamb2 | Rattus norvegicus laminin, beta 2 | ***13*** | 0.39 | -0.98 | -2.36 |
| LOC497691 | Rattus norvegicus hypothetical gene supported by NM_134416 | ***13*** | 0.64 | -0.94 | -2.44 |
| Dnajb4 | Rattus norvegicus DnaJ | ***13*** | 0.93 | -1.2 | -2.35 |
| Zfp36l2 | Rattus norvegicus zinc finger protein 36, C3H type-like 2 | ***13*** | 0.88 | -1.64 | -2.06 |
| LOC315903 | Rattus norvegicus similar to CG9346-PA | ***13*** | 1.28 | -2.16 | -1.89 |
| Mdm2 | Rattus norvegicus transformed mouse 3T3 cell double minute 2 | ***13*** | 1.53 | -1.59 | -1.75 |
| Mdm4 | Rattus norvegicus transformed mouse 3T3 cell double minute 4 | ***13*** | 1.67 | -0.99 | -2.16 |
| Dmd | Rattus norvegicus dystrophin | ***13*** | 1.78 | -0.91 | -2.77 |
| Atp6ap2 | Rattus norvegicus ATPase, H+ transporting, lysosomal accessory protein 2 | ***13*** | 0.81 | -1.67 | -0.98 |
| Flot1 | Rattus norvegicus flotillin 1 | ***13*** | 0.93 | -1.78 | -1.05 |
| Pnrc1 | Rattus norvegicus proline rich 2 | ***13*** | 1.35 | -1.51 | -0.87 |
| LOC360941 | Rattus norvegicus similar to ORF7 | ***13*** | 1.45 | -2.03 | -1.09 |
| Rab24 | Rattus norvegicus RAB24, member RAS oncogene family | ***13*** | 1.58 | -0.99 | -1.47 |
| LOC500226 | Rattus norvegicus similar to D3Mm3e | ***13*** | 1.67 | -1.14 | -1 |
| Pim3 | Rattus norvegicus serine/threonine-protein kinase pim-3 | ***13*** | 1.69 | -1.22 | -1.08 |
| Wdfy1 | Rattus norvegicus WD repeat and FYVE domain containing 1 | ***13*** | 1.86 | -1.33 | -1.08 |
| LOC303554 | Rattus norvegicus similar to Nbr1 | ***13*** | 2 | -1.55 | -0.81 |
| Dre1 | Rattus norvegicus Dre1 protein | ***13*** | 2.11 | -1.75 | -1.37 |
|  |  |  |  |  |  |
| Cxcl10 | Rattus norvegicus chemokine | ***39*** | 2.21 | -1.83 | -3.34 |
| Adm | Rattus norvegicus adrenomedullin | ***39*** | 2.84 | -2.57 | -2.58 |
| LOC497803 | Rattus norvegicus hypothetical gene supported by NM_178095 | ***39*** | 3.78 | -1.43 | -3.35 |
|  |  |  |  |  |  |
| Myc | Rattus norvegicus myelocytomatosis viral oncogene homolog | ***17*** | 0.61 | -1.82 | -0.28 |
| Lsamp | Rattus norvegicus limbic system-associated membrane protein | ***17*** | 0.93 | -1.53 | -0.2 |
| Banp | Rattus norvegicus Btg3 associated nuclear protein | ***17*** | 0.95 | -1.88 | 0.14 |
| LOC367857 | Rattus norvegicus similar to ubiquitin fusion degradation protein 2 | ***17*** | 1.5 | -1.24 | -0.11 |
| Mcl1 | Rattus norvegicus myeloid cell leukemia sequence 1 | ***17*** | 1.8 | -1.22 | -0.07 |
| Vapa | Rattus norvegicus vesicle-associated membrane protein, associated protein a | ***17*** | 1.52 | -1.07 | -0.51 |
| Jund | Rattus norvegicus Jun D proto-oncogene | ***17*** | 1.72 | -1.19 | 0.93 |
| LOC301068 | Rattus norvegicus similar to translation factor sui1 homolog | ***17*** | 2.04 | -1.03 | 0.36 |
| Mrpl45 | Rattus norvegicus mitochondrial ribosomal protein L45 | ***17*** | 2.22 | -1.01 | 0.2 |
| Ugp2 | Rattus norvegicus UDP-glucose pyrophosphorylase 2 | ***17*** | 2.3 | -1.47 | 0.59 |
| LOC365699 | Rattus norvegicus similar to hypothetical protein FLJ30596 | ***17*** | 2.19 | -2.31 | 0.58 |
| Junb | Rattus norvegicus Jun-B oncogene | ***17*** | 1.51 | -0.94 | 1.5 |
| LOC497816 | Rattus norvegicus hypothetical gene supported by NM_019371 | ***17*** | 1.77 | -1.02 | 1.52 |
| Ddit4 | Rattus norvegicus DNA-damage-inducible transcript 4 | ***17*** | 2.02 | -0.87 | 1.7 |
| Hnrpl | Rattus norvegicus heterogeneous nuclear ribonucleoprotein L | ***17*** | 1.75 | -0.37 | 1.27 |
| Vars2 | Rattus norvegicus valyl-tRNA synthetase 2 | ***17*** | 2.03 | -0.43 | 1.5 |
| Umpk | Rattus norvegicus uridine monophosphate kinase | ***17*** | 2.15 | -0.7 | 1.8 |
| Bri3 | Rattus norvegicus brain protein I3 | ***17*** | 2.17 | -0.53 | 1.82 |
| Dpp7 | Rattus norvegicus dipeptidylpeptidase 7 | ***17*** | 2.16 | -0.35 | 1.82 |
| Nol8 | Rattus norvegicus nucleolar protein 8 | ***17*** | 1.52 | -0.33 | 0.89 |
| Ubqln1 | Rattus norvegicus ubiquilin 1 | ***17*** | 1.53 | -0.06 | 1.03 |
| Ccng2 | Rattus norvegicus cyclin G2 | ***17*** | 1.79 | -0.57 | 0.89 |
| St13 | Rattus norvegicus suppression of tumorigenicity 13 | ***17*** | 1.83 | -0.53 | 0.96 |
| Tbc1d15 | Rattus norvegicus TBC1 domain family, member 15 | ***17*** | 2.07 | -0.51 | 0.68 |
| C3orf6h | Rattus norvegicus putative C3orf6 protein homolog | ***17*** | 2.2 | -0.43 | 0.69 |
| Hspcal3 | Rattus norvegicus heat shock 90kDa protein 1, alpha-like 3 | ***17*** | 2.39 | -0.71 | 0.84 |
| Hspca | Rattus norvegicus heat shock protein 1, alpha | ***17*** | 2.57 | -1.41 | 1.69 |
| Uap1l1 | Rattus norvegicus UDP-N-acteylglucosamine pyrophosphorylase 1-like 1 | ***17*** | 3.34 | -1.35 | 1.66 |
| Rragd | Rattus norvegicus Ras-related GTP binding D | ***17*** | 3.66 | -1.29 | 1.02 |
| Plekhm2 | Rattus norvegicus pleckstrin homology domain containing, family M | ***17*** | 1.57 | -0.14 | 0.37 |
| Ckb | Rattus norvegicus creatine kinase, brain | ***17*** | 1.57 | -0.03 | 0.5 |
| Rragc | Rattus norvegicus Ras-related GTP binding C | ***17*** | 1.65 | -0.08 | 0.59 |
| Clta | Rattus norvegicus clathrin, light polypeptide | ***17*** | 1.77 | 0.04 | 0.26 |
| LOC499690 | Rattus norvegicus similar to RIKEN cDNA 2010200O16 | ***17*** | 1.83 | -0.08 | 0.44 |
| Eif4ebp1 | Rattus norvegicus eukaryotic translation initiation factor 4E binding protein 1 | ***17*** | 2.06 | -0.18 | 0.22 |
| LOC499171 | Rattus norvegicus similar to BTEB3 protein | ***17*** | 2.27 | -0.28 | 0.4 |
| LOC499523 | Rattus norvegicus similar to 60S ribosomal protein L23a | ***17*** | 2.3 | -0.14 | 0.36 |
| Rpl3 | Rattus norvegicus ribosomal protein L3 | ***17*** | 2.28 | 0.02 | 0.5 |
| Prss15 | Rattus norvegicus protease, serine, 15 | ***17*** | 2.41 | 0.18 | 0.31 |
| LOC293103 | Rattus norvegicus similar to RIKEN cDNA 0610007P06 | ***17*** | 1.58 | -0.29 | 0.19 |
| LOC300870 | Rattus norvegicus similar to hypothetical protein FLJ20037 | ***17*** | 1.74 | -0.14 | 0.11 |
| Lamp1 | Rattus norvegicus lysosomal membrane glycoprotein 1 | ***17*** | 1.62 | -0.46 | 0.11 |
| Pamci | Rattus norvegicus peptidylglycine alpha-amidating monooxygenase COOH-terminal interactor | ***17*** | 1.64 | -0.43 | 0.04 |
| Arih1 | Rattus norvegicus ariadne ubiquitin-conjugating enzyme E2 binding protein homolog 1 | ***17*** | 1.63 | -0.58 | 0.08 |
| Trit1 | Rattus norvegicus tRNA isopentenyltransferase 1 | ***17*** | 1.81 | -0.5 | -0.19 |
| RGD1307599 | Rattus norvegicus similar to Mitogen-inducible gene 6 protein homolog | ***17*** | 2.26 | -0.6 | -0.12 |
| Id2 | Rattus norvegicus Inhibitor of DNA binding 2, dominant negative helix-loop-helix protein | ***17*** | 2.36 | -0.44 | -0.03 |
| LOC310395 | Rattus norvegicus similar to carbon catabolite repression 4 protein homolog | ***17*** | 2.53 | -0.74 | -0.1 |
| MGC94288 | Rattus norvegicus similar to 4632419K20Rik protein | ***17*** | 2.56 | -0.91 | -0.01 |
| Bhlhb3 | Rattus norvegicus basic helix-loop-helix domain containing, class B3 | ***17*** | 2.38 | -0.4 | 0.48 |
| Pfkp | Rattus norvegicus phosphofructokinase, platelet | ***17*** | 2.59 | -0.35 | 0.5 |
| Pawr | Rattus norvegicus PRKC, apoptosis, WT1, regulator | ***17*** | 2.55 | -0.33 | 0.15 |
| Maff | Rattus norvegicus v-maf musculoaponeurotic fibrosarcoma oncogene family, protein F | ***17*** | 2.68 | -0.65 | 0.3 |
| Mak3 | Rattus norvegicus Mak3 homolog | ***17*** | 3.33 | -0.43 | 0.6 |
| Rere | Rattus norvegicus arginine-glutamic acid dipeptide | ***17*** | 3.46 | -0.32 | -0.11 |
|  |  |  |  |  |  |
| LOC500974 | Rattus norvegicus similar to CDNA sequence BC024479 | ***31*** | 1.13 | 0.02 | -1.54 |
| LOC361117 | Rattus norvegicus similar to LRRGT00149 | ***31*** | 1.33 | -0.13 | -1.65 |
| LOC498105 | Rattus norvegicus similar to LRRGT00176 | ***31*** | 1.52 | 0.18 | -1.4 |
| Cxcl1 | Rattus norvegicus chemokine | ***31*** | 1.28 | -0.43 | -1.54 |
| Mafg | Rattus norvegicus v-maf musculoaponeurotic fibrosarcoma oncogene family, protein G | ***31*** | 1.39 | -0.31 | -1.89 |
| Dtr | Rattus norvegicus diphtheria toxin receptor | ***31*** | 1.57 | 0.01 | -0.53 |
| Ppp2r2a | Rattus norvegicus protein phosphatase 2 | ***31*** | 1.84 | 0.18 | -0.52 |
| Ewsr1 | Rattus norvegicus Ewing sarcoma breakpoint region 1 | ***31*** | 1.92 | 0.05 | -0.36 |
| Nfe2l2 | Rattus norvegicus nuclear factor, erythroid derived 2, like 2 | ***31*** | 2 | 0.12 | -0.42 |
| Slc40a1 | Rattus norvegicus solute carrier family 39 | ***31*** | 1.67 | 0 | -1.09 |
| LOC360807 | Rattus norvegicus LOC360807 | ***31*** | 1.78 | -0.06 | -0.85 |
| Hif1a | Rattus norvegicus hypoxia inducible factor 1, alpha subunit | ***31*** | 1.77 | -0.66 | -0.77 |
| Tpm1 | Rattus norvegicus tropomyosin 1, alpha | ***31*** | 1.91 | -0.62 | -0.56 |
| Tiparp | Rattus norvegicus TCDD-inducible poly | ***31*** | 1.91 | -0.27 | -0.63 |
| Ddr1 | Rattus norvegicus discoidin domain receptor family, member 1 | ***31*** | 2.22 | -0.92 | -0.9 |
| Slc38a1 | Rattus norvegicus solute carrier family 38, member 1 | ***31*** | 2.52 | -0.77 | -1.04 |
| Eef2 | Rattus norvegicus eukaryotic translation elongation factor 2 | ***31*** | 1.5 | 0.59 | 0.01 |
| LOC311772 | Rattus norvegicus similar to nidogen 2 | ***31*** | 1.65 | 0.56 | 0.2 |
| Cops4 | Rattus norvegicus COP9 signalosome subunit 4 | ***31*** | 1.67 | 0.77 | 0.13 |
| RGD1311518 | Rattus norvegicus similar to rab3 GTPase-activating protein, non-catalytic subunit | ***31*** | 1.53 | 0.62 | -0.22 |
| Cggbp1 | Rattus norvegicus CGG triplet repeat binding protein 1 | ***31*** | 1.68 | 0.64 | -0.28 |
| Ywhae | Rattus norvegicus tyrosine 3-monooxygenase/tryptophan 5-monooxygenase activation protein, epsilon polypeptide | ***31*** | 1.61 | 0.5 | -0.17 |
| Atp6v1g1 | Rattus norvegicus ATPase, H+ transporting, V1 subunit G isoform 1 | ***31*** | 1.61 | 0.51 | -0.44 |
| Vgll4 | Rattus norvegicus vestigial like 4 | ***31*** | 1.85 | 0.36 | 0 |
| LOC365753 | Rattus norvegicus similar to Spindlin | ***31*** | 1.9 | 0.23 | -0.05 |
| LOC366693 | Rattus norvegicus similar to S164 | ***31*** | 1.53 | 0.74 | -0.7 |
| Actn1 | Rattus norvegicus actinin, alpha 1 | ***31*** | 1.56 | 0.75 | -0.59 |
| Tln | Rattus norvegicus talin | ***31*** | 1.54 | 0.92 | -0.5 |
| Rps6 | Rattus norvegicus ribosomal protein S6 | ***31*** | 1.54 | 0.85 | -0.87 |
| Rpl4 | Rattus norvegicus ribosomal protein L4 | ***31*** | 1.97 | 0.92 | -1.01 |
| Rora | Rattus norvegicus RAR-related orphan receptor alpha | ***31*** | 2.14 | 0.65 | -1 |
| Rps15a | Rattus norvegicus ribosomal protein S15a | ***31*** | 1.97 | 1 | -0.45 |
| Naca | Rattus norvegicus nascent-polypeptide-associated complex alpha polypeptide | ***31*** | 2.07 | 1.01 | -0.53 |
| LOC361115 | Rattus norvegicus similar to tumor protein, translationally-controlled 1 | ***31*** | 1.99 | 0.79 | -0.61 |
| Rnpc2 | Rattus norvegicus RNA-binding region | ***31*** | 2.25 | 0.86 | -0.3 |
| Mgea5 | Rattus norvegicus meningioma expressed antigen 5 | ***31*** | 2.43 | 1.27 | -0.5 |
| Hspbap1 | Rattus norvegicus Hspb associated protein 1 | ***31*** | 2.38 | 0.02 | -0.24 |
| Copeb | Rattus norvegicus core promoter element binding protein | ***31*** | 2.85 | 0.03 | -0.55 |
| LOC498038 | Rattus norvegicus similar to colon carcinoma related protein | ***31*** | 2.98 | 0.3 | -0.46 |
| Cpeb4 | Rattus norvegicus cytoplasmic polyadenylation element binding protein 4 | ***31*** | 3.29 | 0.74 | -0.47 |
| Slc5a3 | Rattus norvegicus solute carrier family 5 | ***31*** | 3.33 | 0.6 | -1.01 |
| LOC499201 | Rattus norvegicus hypothetical gene supported by BC061525 | ***31*** | 3.34 | 0.36 | -0.82 |
| LOC499794 | Rattus norvegicus similar to ribosomal protein L10 | ***31*** | 2.76 | -0.2 | -1.77 |
| Dyrk2 | Rattus norvegicus dual-specificity tyrosine- | ***31*** | 3.18 | -1.24 | -1.13 |
| Nfil3 | Rattus norvegicus nuclear factor, interleukin 3 regulated | ***31*** | 3.25 | -1.3 | -1.33 |
| Atf3 | Rattus norvegicus activating transcription factor 3 | ***31*** | 3.84 | -0.64 | -0.94 |
| Eno2 | Rattus norvegicus enolase 2, gamma | ***31*** | 4.44 | -0.49 | -0.41 |
| Nr1d1 | Rattus norvegicus nuclear receptor subfamily 1, group D, member 1 | ***31*** | 4.5 | -0.45 | -0.27 |
| Pbef1 | Rattus norvegicus pre-B-cell colony enhancing factor 1 | ***31*** | 3.89 | -1.48 | -0.24 |
|  |  |  |  |  |  |
| Dspg3 | Rattus norvegicus dermatan sulphate proteoglycan 3 | ***2*** | -4.14 | 4.12 | 2.62 |
| Tnfrsf11b | Rattus norvegicus tumor necrosis factor receptor superfamily, member 11b | ***2*** | -2.29 | 5.37 | 2.36 |
|  |  |  |  |  |  |
| Snai1 | Rattus norvegicus snail homolog, | ***21*** | -0.8 | 4.97 | 4.13 |
| Ca3 | Rattus norvegicus carbonic anhydrase 3 | ***21*** | -0.47 | 7.28 | 5.19 |
|  |  |  |  |  |  |
| Gdf10 | Rattus norvegicus growth differentiation factor 10 | ***38*** | -0.52 | 6.63 | 1.91 |
| Rbm3 | Rattus norvegicus RNA binding motif | ***38*** | 0.59 | 6.11 | 1.87 |
|  |  |  |  |  |  |
| LOC500987 | Rattus norvegicus similar to Histone H2A.x | ***6*** | -3.69 | -1.61 | 2.15 |
| Hn1 | Rattus norvegicus hematological and neurological expressed sequence 1 | ***6*** | -3.4 | -0.68 | 1.06 |
| Colec12 | Rattus norvegicus collectin sub-family member 12 | ***6*** | -2.83 | -1.25 | 0.99 |
| LOC500252 | Rattus norvegicus similar to Gene model 461 | ***6*** | -2.71 | -0.89 | 1.48 |
| Kpna2 | Rattus norvegicus karyopherin | ***6*** | -2.45 | -1.43 | 1.49 |
| LOC302980 | Rattus norvegicus similar to RIKEN cDNA 1110025H10 | ***6*** | -2.27 | -1.72 | 0.78 |
| Calm3 | Rattus norvegicus calmodulin 3 | ***6*** | -1.9 | -1.65 | 0.98 |
| Cxcl12 | Rattus norvegicus chemokine | ***6*** | -1.83 | -1.12 | 0.86 |
| Tmpo | Rattus norvegicus thymopoietin | ***6*** | -1.72 | -1.35 | 0.73 |
| LOC315697 | Rattus norvegicus similar to RIKEN cDNA 1190002L16 | ***6*** | -1.53 | -1.32 | 0.98 |
| LOC362776 | Rattus norvegicus similar to 2900070E19Rik protein | ***6*** | -1.68 | -1.12 | 1.28 |
| Msx1 | Rattus norvegicus homeo box, msh-like 1 | ***6*** | -1.61 | -1.28 | 1.17 |
| Usp47 | Rattus norvegicus ubiquitin specific protease 47 | ***6*** | -1.54 | -1.02 | 1.12 |
| Ppp1r14b | Rattus norvegicus protein phosphatase 1, regulatory | ***6*** | -1.7 | -2.01 | 1.65 |
| LOC500441 | Rattus norvegicus similar to testes development-related NYD-SP22 isoform 1 | ***6*** | -1.58 | -1.6 | 1.4 |
| Abcb9 | Rattus norvegicus ATP-binding cassette, sub-family B | ***6*** | -2.26 | -0.44 | 3.17 |
| Thrsp | Rattus norvegicus thyroid hormone responsive protein | ***6*** | -1.06 | -0.44 | 3.73 |
| LOC311592 | Rattus norvegicus similar to hypothetical protein D630003M21 | ***6*** | -1.38 | -2.15 | 3 |
|  |  |  |  |  |  |
| Idh1 | Rattus norvegicus isocitrate dehydrogenase 1 | ***23*** | -3.26 | 1.27 | 1.21 |
| Ppp1ca | Rattus norvegicus protein phosphatase 1, catalytic subunit, alpha isoform | ***23*** | -2.15 | 0.68 | 0.74 |
| Tdg | Rattus norvegicus thymine-DNA glycosylase | ***23*** | -2.02 | 0.55 | 1.14 |
| Cdk2ap1 | Rattus norvegicus CDK2 | ***23*** | -1.9 | 0.52 | 1.05 |
| Chd4 | Rattus norvegicus chromodomain helicase DNA binding protein 4 | ***23*** | -1.75 | 0.33 | 0.73 |
| LOC305633 | Rattus norvegicus similar to Antxr2 protein | ***23*** | -1.61 | 0.35 | 0.67 |
| Ube4a | Rattus norvegicus ubiquitin conjugation factor E4 A | ***23*** | -1.58 | 0.37 | 0.66 |
| LOC362592 | Rattus norvegicus hypothetical LOC362592 | ***23*** | -1.51 | 0.37 | 0.73 |
| LOC498736 | Rattus norvegicus similar to tubulin, beta 2 | ***23*** | -1.65 | 0.74 | 0.77 |
| Mrpl49 | Rattus norvegicus mitochondrial ribosomal protein L49 | ***23*** | -1.54 | 0.74 | 0.76 |
| Mapk7 | Rattus norvegicus mitogen-activated protein kinase 7 | ***23*** | -1.51 | 0.7 | 0.57 |
| Id3 | Rattus norvegicus inhibitor of DNA binding 3 | ***23*** | -2.07 | 0.99 | 1.1 |
| LOC298500 | Rattus norvegicus similar to hypothetical protein AL133206 | ***23*** | -2.07 | 1.3 | 1.12 |
| Gp1bb | Rattus norvegicus peanut | ***23*** | -2.03 | 1.04 | 0.87 |
| Mesdc2 | Rattus norvegicus mesoderm development candiate 2 | ***23*** | -1.79 | 1.05 | 0.82 |
| Cdc91l1 | Rattus norvegicus CDC91 cell division cycle 91-like 1 | ***23*** | -1.79 | 0.98 | 1.28 |
| Cenpb | Rattus norvegicus centromere autoantigen B | ***23*** | -1.72 | 1.04 | 1.33 |
| Rgs3 | Rattus norvegicus regulator of G-protein signalling 3 | ***23*** | -1.53 | 0.91 | 1.2 |
| LOC298906 | Rattus norvegicus similar to RIKEN cDNA E030024M05 | ***23*** | -1.88 | 1.19 | 0.46 |
| Mmp14 | Rattus norvegicus matrix metalloproteinase 14 | ***23*** | -1.73 | 1.23 | 0.59 |
| MGC93733 | Rattus norvegicus similar to RIKEN cDNA 6530411B15 | ***23*** | -1.88 | 1.38 | 0.72 |
| Ckap4 | Rattus norvegicus cytoskeleton-associated protein 4 | ***23*** | -1.82 | 0.84 | 0.44 |
| Thra | Rattus norvegicus thyroid hormone receptor alpha | ***23*** | -1.61 | 0.99 | 0.57 |
| LOC361980 | Rattus norvegicus similar to RIKEN cDNA 2810403A07 | ***23*** | -1.58 | 0.82 | 0.34 |
| Rasl11b | Rattus norvegicus RAS-like family 11 member B | ***23*** | -1.51 | 1.02 | 0.37 |
| Col5a1 | Rattus norvegicus collagen, type V, alpha 1 | ***23*** | -1.66 | 1.18 | 0.17 |
| Mrps12 | Rattus norvegicus mitochondrial ribosomal protein S12 | ***23*** | -2.73 | -0.41 | 0.82 |
| Hspa14 | Rattus norvegicus heat shock protein hsp70-related protein | ***23*** | -2.4 | -0.42 | 1.24 |
| Pcyox1 | Rattus norvegicus chloride ion pump-associated 55 kDa protein | ***23*** | -2.5 | -0.15 | 0.82 |
| Bak1 | Rattus norvegicus BCL2-antagonist/killer 1 | ***23*** | -2.31 | -0.25 | 0.85 |
| Polr2d | Rattus norvegicus polymerase | ***23*** | -2.16 | -0.17 | 0.9 |
| Abhd8 | Rattus norvegicus abhydrolase domain containing 8 | ***23*** | -2.09 | -0.37 | 1.06 |
| Ormdl2 | Rattus norvegicus ORM1-like 2 | ***23*** | -2 | -0.07 | 1.02 |
| Efnb1 | Rattus norvegicus ephrin B1 | ***23*** | -2.02 | -0.16 | 1.37 |
| RGD1306395 | Rattus norvegicus similar to 9530046H09Rik protein | ***23*** | -1.83 | -0.2 | 1.42 |
| Dr1 | Rattus norvegicus down-regulator of transcription 1 | ***23*** | -1.7 | -0.25 | 1.29 |
| Txndc7 | Rattus norvegicus thioredoxin domain containing 7 | ***23*** | -1.93 | -0.74 | 1.02 |
| Cycs | Rattus norvegicus cytochrome c, somatic | ***23*** | -1.81 | -0.61 | 1.34 |
| Glrx1 | Rattus norvegicus glutaredoxin 1 | ***23*** | -1.8 | -0.38 | 0.92 |
| LOC500855 | Rattus norvegicus similar to RIKEN cDNA 3110006P09 | ***23*** | -1.55 | -0.27 | 0.91 |
| MGC94479 | Rattus norvegicus similar to Protein C3orf4 homolog | ***23*** | -1.53 | -0.41 | 1.07 |
| LOC499084 | Rattus norvegicus similar to hepatic multiple inositol polyphosphate phosphatase | ***23*** | -2.55 | 0.31 | 0.73 |
| Sqle | Rattus norvegicus squalene epoxidase | ***23*** | -2.41 | 0.45 | 0.91 |
| Minpp1 | Rattus norvegicus multiple inositol polyphosphate histidine phosphatase 1 | ***23*** | -2.51 | 0.25 | 1.26 |
| Np | Rattus norvegicus nucleoside phosphorylase | ***23*** | -2.39 | 0.65 | 1.1 |
| MGC105691 | Rattus norvegicus NIPSNAP-related protein | ***23*** | -2.38 | 0.5 | 1.09 |
| RAMP4 | Rattus norvegicus ribosome associated membrane protein 4 | ***23*** | -2.02 | 0.11 | 1.39 |
| Amd1 | Rattus norvegicus S-adenosylmethionine decarboxylase 1 | ***23*** | -1.96 | 0.19 | 1.17 |
| Pigt | Rattus norvegicus phosphatidylinositol glycan, class T | ***23*** | -1.71 | 0.06 | 1.49 |
| Acp2 | Rattus norvegicus acid phosphatase 2, lysosomal | ***23*** | -1.66 | 0.17 | 1.32 |
| LOC361695 | Rattus norvegicus similar to 2410001H17Rik protein | ***23*** | -1.57 | 0.16 | 1.24 |
| Slc20a2 | Rattus norvegicus solute carrier family 20, member 2 | ***23*** | -1.51 | 0.13 | 1.32 |
| LOC309848 | Rattus norvegicus similar to Tubulin alpha-2 chain | ***23*** | -1.76 | 0.17 | 0.99 |
| Col16a1 | Rattus norvegicus procollagen, type XVI, alpha 1 | ***23*** | -1.68 | 0.18 | 0.86 |
| MGC94463 | Rattus norvegicus O-linked mannose beta1,2-N-acetylglucosaminyltransferase | ***23*** | -1.73 | -0.03 | 1.15 |
| LOC363309 | Rattus norvegicus similar to tubulin-specific chaperone d | ***23*** | -1.58 | 0 | 1.02 |
| Arpc5 | Rattus norvegicus actin related protein 2/3 complex, subunit 5 | ***23*** | -1.51 | 0.47 | 1.14 |
|  |  |  |  |  |  |
| Mtch2 | Rattus norvegicus mitochondrial carrier homolog 2 | ***33*** | -1.81 | 1.7 | 1.64 |
| Nedd9 | Rattus norvegicus neural precursor cell expressed, developmentally down-regulated gene 9 | ***33*** | -1.63 | 2.2 | 2.05 |
| Psmd5 | Rattus norvegicus proteasome | ***33*** | -1.13 | 1.44 | 1.82 |
| Sod3 | Rattus norvegicus superoxide dismutase 3, extracellular | ***33*** | -0.98 | 1.28 | 2.11 |
| Eif4a1 | Rattus norvegicus eukaryotic translation initiation factor 4A1 | ***33*** | -1 | 1.01 | 1.81 |
| Carhsp1 | Rattus norvegicus calcium regulated heat stable protein 1 | ***33*** | -0.92 | 1.08 | 1.8 |
| Sfrs10 | Rattus norvegicus splicing factor, arginine/serine-rich 10 | ***33*** | -0.83 | 1.08 | 1.88 |
| Pam | Rattus norvegicus peptidylglycine alpha-amidating monooxygenase | ***33*** | -0.65 | 1.22 | 1.84 |
| LOC299907 | Rattus norvegicus similar to Ext1 | ***33*** | -0.95 | 1.67 | 2.19 |
| Col8a1 | Rattus norvegicus procollagen, type VIII, alpha 1 | ***33*** | -0.66 | 1.57 | 2.18 |
| Stmn2 | Rattus norvegicus stathmin-like 2 | ***33*** | -0.58 | 1.62 | 2.42 |
| Colm | Rattus norvegicus collomin | ***33*** | -0.49 | 1.72 | 1.85 |
| Fbn1 | Rattus norvegicus fibrillin 1 | ***33*** | -1.38 | 1.99 | 1.25 |
| LOC300760 | Rattus norvegicus similar to H3 histone, family 3B | ***33*** | -1.26 | 1.65 | 1.58 |
| Tfb2m | Rattus norvegicus transcription factor B2, mitochondrial | ***33*** | -1.09 | 1.62 | 1.32 |
| Agtr1a | Rattus norvegicus angiotensin II receptor, type 1 | ***33*** | -0.95 | 1.6 | 1.02 |
| Dia1 | Rattus norvegicus diaphorase 1 | ***33*** | -0.91 | 1.83 | 1.29 |
| Sdf4 | Rattus norvegicus stromal cell derived factor 4 | ***33*** | -0.91 | 1.82 | 1.09 |
| Epdr2 | Rattus norvegicus ependymin related protein 2 | ***33*** | -0.59 | 1.85 | 1.35 |
| Eif4el3 | Rattus norvegicus eukaryotic translation initiation factor 4E like 3 | ***33*** | -0.45 | 1.53 | 1.32 |
| Hmgn2 | Rattus norvegicus high mobility group protein 17 | ***33*** | -0.92 | 2.29 | 1.84 |
| Pcolce | Rattus norvegicus procollagen C-proteinase enhancer protein | ***33*** | -0.91 | 2.48 | 1.53 |
| Cct8 | Rattus norvegicus chaperonin subunit 8 | ***33*** | -0.55 | 2.28 | 1.44 |
| Mk1 | Rattus norvegicus Mk1 protein | ***33*** | -0.32 | 2.27 | 1.61 |
| Tm4sf1 | Rattus norvegicus transmembrane 4 superfamily member 1 | ***33*** | -1.5 | 1.51 | 2.71 |
| Fdps | Rattus norvegicus farensyl diphosphate synthase | ***33*** | -1.31 | 1.66 | 2.75 |
| Wisp2 | Rattus norvegicus WNT1 inducible signaling pathway protein 2 | ***33*** | -1.47 | 1.32 | 3.04 |
| Col14a1 | Rattus norvegicus collagen, type XIV, alpha 1 | ***33*** | -0.87 | 1.44 | 3.25 |
| Lum | Rattus norvegicus lumican | ***33*** | -1.21 | 2.64 | 2.51 |
| Serpine2 | Rattus norvegicus serine | ***33*** | -1.07 | 2.92 | 2.61 |
|  |  |  |  |  |  |
| Tnnt2 | Rattus norvegicus troponin T2, cardiac | ***28*** | -1.73 | 0.96 | 1.8 |
| Commd8 | Rattus norvegicus COMM domain containing 8 | ***28*** | -1.44 | 0.43 | 1.97 |
| LOC316539 | Rattus norvegicus similar to Eph receptor A4 | ***28*** | -1.38 | 0.63 | 1.66 |
| LOC503418 | Rattus norvegicus LOC503418 | ***28*** | -1.37 | 0.46 | 1.73 |
| Asam | Rattus norvegicus adipocyte-specific adhesion molecule | ***28*** | -1.24 | 0.79 | 1.81 |
| Apeh | Rattus norvegicus N-acylaminoacyl-peptide hydrolase | ***28*** | -1.14 | 0.82 | 1.8 |
| Map2k3 | Rattus norvegicus mitogen activated protein kinase kinase 3 | ***28*** | -1.04 | 0.96 | 1.66 |
| Bambi | Rattus norvegicus BMP and activin membrane-bound inhibitor, homolog | ***28*** | -1.02 | 0.58 | 1.66 |
| Ubtd1 | Rattus norvegicus ubiquitin domain containing 1 | ***28*** | -0.82 | 0.76 | 1.69 |
| Zfp207 | Rattus norvegicus zinc finger protein 207 | ***28*** | -1.17 | 0.68 | 2.08 |
| Tram1 | Rattus norvegicus translocation associated membrane protein 1 | ***28*** | -0.99 | 0.72 | 2.15 |
| Dnajb11 | Rattus norvegicus DnaJ | ***28*** | -1.34 | 0.09 | 1.63 |
| Fbxo33 | Rattus norvegicus F-box only protein 33 | ***28*** | -1.15 | -0.03 | 1.97 |
| RGD1359127 | Rattus norvegicus similar to RIKEN cDNA 2310011J03 | ***28*** | -1.12 | 0.18 | 1.82 |
| Txndc5 | Rattus norvegicus thioredoxin domain containing 5 | ***28*** | -1.12 | 0.16 | 2.14 |
| LOC299127 | Rattus norvegicus similar to RIKEN cDNA 1200003C05 | ***28*** | -1.07 | 0.47 | 2.18 |
| Ywhag | Rattus norvegicus tyrosine 3-monooxgenase/tryptophan 5-monooxgenase activation protein, gamma polypeptide | ***28*** | -1.06 | 0.38 | 1.97 |
| Dirc2 | Rattus norvegicus disrupted in renal carcinoma 2 | ***28*** | -1.03 | 0.21 | 1.61 |
| LOC501007 | Rattus norvegicus similar to RIKEN cDNA 6030419C18 gene | ***28*** | -0.86 | 0.24 | 1.53 |
| Tcn2 | Rattus norvegicus transcobalamin 2 | ***28*** | -0.85 | 0.33 | 1.65 |
| LOC292780 | Rattus norvegicus similar to hypothetical protein MGC15677 | ***28*** | -0.52 | 0.14 | 1.51 |
| Ptn | Rattus norvegicus pleiotrophin | ***28*** | -0.84 | 0.44 | 1.83 |
| Ranbp1 | Rattus norvegicus RAN binding protein 1 | ***28*** | -0.77 | 0.34 | 1.77 |
| Fads1 | Rattus norvegicus fatty acid desaturase 1 | ***28*** | -0.7 | 0.39 | 1.83 |
| Ttk | Rattus norvegicus Ttk protein kinase | ***28*** | -0.6 | 0.37 | 1.86 |
| Ndn | Rattus norvegicus necdin | ***28*** | -0.71 | 0.14 | 1.98 |
| Echdc1 | Rattus norvegicus enoyl Coenzyme A hydratase domain containing 1 | ***28*** | -0.56 | 0.11 | 1.97 |
| Crebl1 | Rattus norvegicus cAMP responsive element binding protein-like 1 | ***28*** | -0.53 | 0.14 | 1.93 |
| Foxg1 | Rattus norvegicus forkhead box G1 | ***28*** | -0.98 | 0.14 | 2.43 |
| Scd1 | Rattus norvegicus stearoyl-Coenzyme A desaturase 1 | ***28*** | -0.96 | 0.16 | 2.66 |
| Igfbp3 | Rattus norvegicus insulin-like growth factor binding protein 3 | ***28*** | -0.44 | 0.84 | 2.46 |
| LOC498406 | Rattus norvegicus similar to mitochondrial ribosomal protein S24 | ***28*** | -0.4 | 0.23 | 2.61 |
| LOC497936 | Rattus norvegicus similar to RIKEN cDNA 2600017H02 | ***28*** | -0.25 | 0.21 | 3.07 |
| Znf297 | Rattus norvegicus zinc finger protein 297 | ***28*** | -0.53 | 0.74 | 1.87 |
| LOC302500 | Rattus norvegicus similar to malignant T cell amplified sequence 1 | ***28*** | -0.41 | 0.66 | 2 |
| Dab2ip | Rattus norvegicus disabled homolog 2 | ***28*** | -0.34 | 0.79 | 1.97 |
| Ddx46 | Rattus norvegicus RNA helicase | ***28*** | -0.52 | 0.7 | 1.57 |
| LOC313722 | Rattus norvegicus similar to SPRY domain-containing SOCS box protein SSB-1 | ***28*** | -0.26 | 0.65 | 1.57 |
| Nme1 | Rattus norvegicus expressed in non-metastatic cells 1 | ***28*** | -0.39 | 0.4 | 1.72 |
| Plxdc2 | Rattus norvegicus plexin domain containing 2 | ***28*** | -0.09 | 0.48 | 1.89 |
| Mphosph10 | Rattus norvegicus M-phase phosphoprotein 10 | ***28*** | -0.08 | 0.44 | 1.58 |
| LOC500795 | Rattus norvegicus LOC500795 | ***28*** | 0.12 | 0.45 | 1.55 |
| Mdk | Rattus norvegicus midkine | ***28*** | -0.18 | 0.95 | 1.56 |
| Akr1b4 | Rattus norvegicus aldo-keto reductase family 1, member B4 | ***28*** | -0.04 | 0.94 | 1.75 |
| Sfrs2 | Rattus norvegicus similar to splicing factor, arginine/serine-rich 2 | ***28*** | 0.09 | 0.89 | 1.69 |
| Enpp1 | Rattus norvegicus ectonucleotide pyrophosphatase/phosphodiesterase 1 | ***28*** | 0.15 | 0.87 | 1.57 |
| Cct6a | Rattus norvegicus chaperonin subunit 6a | ***28*** | 0.17 | 0.63 | 1.58 |
| Pafah1b2 | Rattus norvegicus platelet-activating factor acetylhydrolase, isoform 1b, alpha2 subunit | ***28*** | -0.28 | 0.17 | 2.1 |
| LOC315329 | Rattus norvegicus similar to expressed sequence AW556797 | ***28*** | -0.24 | 0.23 | 2.06 |
| LOC303471 | Rattus norvegicus similar to Stathmin | ***28*** | -0.16 | 0.3 | 2.04 |
| Smfn | Rattus norvegicus small fragment nuclease | ***28*** | 0 | 0.34 | 2.3 |
| Exosc5 | Rattus norvegicus exosome component 5 | ***28*** | 0.12 | 0.11 | 1.96 |
| Ilvbl | Rattus norvegicus ilvB | ***28*** | 0.16 | 0.29 | 2.06 |
| LOC308503 | Rattus norvegicus similar to Set beta isoform | ***28*** | 0.09 | 0.48 | 2.08 |
| Ppap2c | Rattus norvegicus phosphatidic acid phosphatase type 2c | ***28*** | 0.16 | 0.56 | 2.2 |
| Rae1 | Rattus norvegicus RAE1 RNA export 1 homolog | ***28*** | 0.23 | 0.63 | 2.05 |
| Tax1bp1 | Rattus norvegicus Tax1 | ***28*** | 0.4 | 0.6 | 2.22 |
| LOC501536 | Rattus norvegicus similar to Heterogeneous nuclear ribonucleoprotein A1 | ***28*** | 0.35 | 0.84 | 1.93 |
| Tmepai | Rattus norvegicus transmembrane, prostate androgen induced RNA | ***28*** | 0.42 | 0.84 | 2.02 |
| Apex1 | Rattus norvegicus apurinic/apyrimidinic endonuclease 1 | ***28*** | 0.43 | 0.72 | 2.07 |
| C1s | Rattus norvegicus complement component 1, s subcomponent | ***28*** | 0.55 | 0.42 | 1.86 |
| MGC94190 | Rattus norvegicus similar to 0610007L01Rik protein | ***28*** | 0.55 | 0.22 | 1.86 |
| LOC498363 | Rattus norvegicus similar to 60S acidic ribosomal protein P2 | ***28*** | 0.57 | 0.77 | 1.8 |
| LOC361571 | Rattus norvegicus similar to RIKEN cDNA 2410004H02 | ***28*** | 0.7 | 0.58 | 1.71 |
| LOC299823 | Rattus norvegicus similar to ribosomal protein S10 | ***28*** | 0.62 | 0.8 | 1.54 |
| Tp53rk | Rattus norvegicus TP53 regulating kinase | ***28*** | -1.42 | -0.55 | 1.54 |
| Slc25a1 | Rattus norvegicus solute carrier family 25, member 1 | ***28*** | -0.97 | -0.44 | 1.98 |
| Gng11 | Rattus norvegicus guanine nucleotide binding protein | ***28*** | -0.69 | -0.7 | 1.95 |
| Phax | Rattus norvegicus phosphorylated adaptor for RNA export | ***28*** | -0.52 | -0.44 | 1.65 |
| Lancl2 | Rattus norvegicus LanC | ***28*** | -1.3 | -0.41 | 2.27 |
| LOC290706 | Rattus norvegicus similar to 2700029M09Rik protein | ***28*** | -1.18 | -0.4 | 2.24 |
| Casp8ap2 | Rattus norvegicus caspase 8 associated protein 2 | ***28*** | -1.28 | -0.93 | 2.3 |
| Bcap29 | Rattus norvegicus B-cell receptor-associated protein BAP29 | ***28*** | -0.89 | -0.99 | 1.62 |
| Ebp | Rattus norvegicus phenylalkylamine Ca2+ antagonist | ***28*** | -0.72 | -1.44 | 1.81 |
| Gadd45gip1 | Rattus norvegicus growth arrest and DNA-damage-inducible, gamma interacting protein 1 | ***28*** | -0.3 | -1.21 | 2.12 |
| LOC292588 | Rattus norvegicus similar to Ubiquitin-conjugating enzyme E2S | ***28*** | -0.42 | -0.82 | 1.7 |
| Ddx21b | Rattus norvegicus DEAD | ***28*** | -0.28 | -0.93 | 1.55 |
| Pgls | Rattus norvegicus 6-phosphogluconolactonase | ***28*** | -0.36 | -1.21 | 1.74 |
| Taf10 | Rattus norvegicus TAF10 RNA polymerase II, TATA box binding protein | ***28*** | -0.23 | -1.17 | 1.71 |
| Smndc1 | Rattus norvegicus survival motor neuron domain containing 1 | ***28*** | -0.06 | -1.02 | 1.8 |
| LOC361578 | Rattus norvegicus similar to pM5 protein; DNA segment, Chr 7, ERATO Doi 156, expressed | ***28*** | -0.01 | -0.76 | 1.57 |
| Ruvbl1 | Rattus norvegicus RuvB-like protein 1 | ***28*** | 0.03 | -0.96 | 1.55 |
| Lcn7 | Rattus norvegicus lipocalin 7 | ***28*** | -0.23 | -0.14 | 1.81 |
| Rabep1 | Rattus norvegicus rabaptin 5 | ***28*** | -0.17 | -0.02 | 1.78 |
| Mdh1 | Rattus norvegicus malate dehydrogenase 1, NAD | ***28*** | -0.15 | -0.28 | 1.53 |
| Qdpr | Rattus norvegicus quinoid dihydropteridine reductase | ***28*** | -0.06 | -0.15 | 1.56 |
| Aldh1a1 | Rattus norvegicus aldehyde dehydrogenase family 1, member A1 | ***28*** | -0.13 | -0.33 | 1.63 |
| Tomm40 | Rattus norvegicus translocase of outer mitochondrial membrane 40 | ***28*** | -0.08 | -0.33 | 1.66 |
| LOC302671 | Rattus norvegicus similar to Adapter-related protein complex 1 sigma 1B subunit | ***28*** | 0 | -0.31 | 1.56 |
| Hmmr | Rattus norvegicus hyaluronan mediated motility receptor | ***28*** | 0.14 | 0.09 | 1.54 |
| LOC362513 | Rattus norvegicus similar to Shb protein | ***28*** | 0.23 | -0.17 | 1.53 |
| Faf1 | Rattus norvegicus Fas-associated factor 1 | ***28*** | 0.4 | 0.06 | 1.67 |
| Fosl1 | Rattus norvegicus fos-like antigen 1 | ***28*** | 0.48 | 0.07 | 1.56 |
| Plod1 | Rattus norvegicus procollagen-lysine, 2-oxoglutarate 5-dioxygenase 1 | ***28*** | 0.35 | -0.19 | 1.83 |
| Cct5 | Rattus norvegicus chaperonin subunit 5 | ***28*** | 0.5 | -0.26 | 2.13 |
| MGC109491 | Rattus norvegicus similar to 1110007F12Rik protein | ***28*** | 0.55 | -0.41 | 1.73 |
| Prc1 | Rattus norvegicus protein regulator of cytokinesis 1 | ***28*** | 0.63 | -0.49 | 1.64 |
| Rpp14 | Rattus norvegicus ribonuclease P 14kDa subunit | ***28*** | 0.64 | -0.38 | 1.8 |
| Dnclc1 | Rattus norvegicus dynein, cytoplasmic, light chain 1 | ***28*** | 0.66 | -0.33 | 1.82 |
| Anp32a | Rattus norvegicus acidic | ***28*** | 0.7 | -0.13 | 1.51 |
| Rara | Rattus norvegicus retinoic acid receptor, alpha | ***28*** | 0.76 | 0 | 1.51 |
| Chchd3 | Rattus norvegicus coiled-coil-helix-coiled-coil-helix domain containing 3 | ***28*** | 0.99 | -0.22 | 1.58 |
| RGD1304567 | Rattus norvegicus LOC362671 | ***28*** | 1.04 | -0.25 | 1.6 |
| LOC498375 | Rattus norvegicus similar to RIKEN cDNA 4930555G01 | ***28*** | 0.41 | -1.2 | 1.66 |
| LOC366411 | Rattus norvegicus similar to ribosomal protein S24 | ***28*** | 0.56 | -1.19 | 1.6 |
| Pfdn1 | Rattus norvegicus prefoldin 1 | ***28*** | 0.62 | -0.84 | 1.62 |
| LOC304638 | Rattus norvegicus similar to RIKEN cDNA 3110001N18 | ***28*** | 0.8 | -0.97 | 1.51 |
| C1qbp | Rattus norvegicus complement component 1, q subcomponent binding protein | ***28*** | 0.79 | -0.98 | 1.7 |
| MGC94233 | Rattus norvegicus similar to RIKEN cDNA 6720485C15 | ***28*** | 0.89 | -0.87 | 1.71 |
| Hist1h2bp | Rattus norvegicus histone 1, H2bp | ***28*** | 0.65 | -0.52 | 2.44 |
| Scand1 | Rattus norvegicus SCAN domain-containing 1 | ***28*** | 1.01 | -0.58 | 2.43 |
|  |  |  |  |  |  |
| Ccl2 | Rattus norvegicus chemokine | ***11*** | -1.47 | 2.74 | 0.34 |
| Ppic | Rattus norvegicus peptidylprolyl isomerase C | ***11*** | -1.33 | 2.18 | 0.48 |
| Penk-rs | Rattus norvegicus preproenkephalin, related sequence | ***11*** | -0.58 | 3.08 | 0.8 |
| LOC500155 | Rattus norvegicus similar to protein kinase C inhibitor | ***11*** | 0.04 | 2.67 | 0.58 |
| LOC499592 | Rattus norvegicus similar to Hnrpa3 protein | ***11*** | 0.08 | 2.44 | 0.56 |
| Thy1 | Rattus norvegicus thymus cell antigen 1, theta | ***11*** | -1.05 | 2.39 | -0.12 |
| Vmp1 | Rattus norvegicus vacuole membrane protein 1 | ***11*** | -0.75 | 2.63 | -0.12 |
| Tle2 | Rattus norvegicus transducin-like enhancer of split 2, homolog of Drosophila E | ***11*** | -0.74 | 2.11 | -0.02 |
| Timp1 | Rattus norvegicus tissue inhibitor of metalloproteinase 1 | ***11*** | -0.7 | 2.02 | -0.11 |
| Mapre1 | Rattus norvegicus microtubule-associated protein, RP/EB family, member 1 | ***11*** | -0.45 | 2.22 | 0.02 |
| Mrps18c | Rattus norvegicus mitochondrial ribosomal protein S18C | ***11*** | -0.42 | 2.16 | 0.01 |
| Thbs2 | Rattus norvegicus thrombospondin 2 | ***11*** | -0.41 | 2.44 | -0.79 |
| Igsf4c | Rattus norvegicus immunoglobulin superfamily, member 4C | ***11*** | -1.41 | 1.67 | 0.19 |
| Ccl7 | Rattus norvegicus chemokine | ***11*** | -1.27 | 1.77 | 0.07 |
| Olfml3 | Rattus norvegicus olfactomedin-like 3 | ***11*** | -1.39 | 1.61 | -0.04 |
| Sfrp2 | Rattus norvegicus secreted frizzled-related protein 2 | ***11*** | -1.18 | 1.62 | -0.3 |
| LOC499317 | Rattus norvegicus similar to UPF0197 protein C11orf10 homolog | ***11*** | -1.15 | 1.64 | 0.17 |
| MGC94283 | Rattus norvegicus integral type I protein | ***11*** | -1.01 | 1.63 | 0.21 |
| LOC306805 | Rattus norvegicus similar to asporin precursor | ***11*** | -1 | 1.65 | 0.28 |
| LOC497693 | Rattus norvegicus similar to P11 protein | ***11*** | -0.89 | 1.67 | -0.08 |
| Nat5 | Rattus norvegicus N-acetyltransferase 5 | ***11*** | -0.88 | 1.66 | -0.05 |
| LOC314759 | Rattus norvegicus similar to genes associated with retinoid-IFN-induced mortality 19 | ***11*** | -0.76 | 1.56 | -0.02 |
| Slc38a2 | Rattus norvegicus solute carrier family 38, member 2 | ***11*** | -0.84 | 1.81 | 0.05 |
| LOC296402 | Rattus norvegicus similar to Docking protein 5 | ***11*** | -1.19 | 1.6 | 0.63 |
| LOC499501 | Rattus norvegicus similar to LRRGT00057 | ***11*** | -1.13 | 1.67 | 0.67 |
| Akap8 | Rattus norvegicus A kinase | ***11*** | -1.11 | 1.64 | 0.79 |
| LOC364258 | Rattus norvegicus similar to RIKEN cDNA 1110003E01 | ***11*** | -0.79 | 1.54 | 0.49 |
| Ssr4 | Rattus norvegicus signal sequence receptor 4 | ***11*** | -0.54 | 1.63 | 0.67 |
| LOC368016 | Rattus norvegicus similar to transmembrane 4 superfamily member 7; tetraspanin 4; novel antigen 2; tetraspan TM4SF | ***11*** | -0.46 | 1.62 | 0.61 |
| LOC503172 | Rattus norvegicus similar to DAZ associated protein 2 | ***11*** | -0.5 | 1.9 | 0.37 |
| Birc6 | Rattus norvegicus baculoviral IAP repeat-containing 6 | ***11*** | -0.42 | 1.79 | 0.31 |
| Tceb2 | Rattus norvegicus transcription elongation factor B | ***11*** | -0.47 | 1.73 | 0.17 |
| Ninj1 | Rattus norvegicus ninjurin 1 | ***11*** | -0.6 | 1.93 | 0.81 |
| Sirt6 | Rattus norvegicus sirtuin 6 | ***11*** | -0.35 | 1.97 | 1.06 |
| LOC302363 | Rattus norvegicus similar to Sh3bgrl protein | ***11*** | -0.18 | 2.14 | 1.25 |
| Jag1 | Rattus norvegicus jagged 1 | ***11*** | 0.02 | 1.65 | 1.22 |
| Phf3 | Rattus norvegicus PHD finger protein 3 | ***11*** | 0.06 | 1.55 | 1.04 |
| LOC311120 | Rattus norvegicus similar to ribosomal protein L15 | ***11*** | 0.14 | 1.57 | 1.02 |
| MGC72560 | Rattus norvegicus Unknown | ***11*** | 0.07 | 1.67 | 1.04 |
| Runx1 | Rattus norvegicus runt related transcription factor 1 | ***11*** | 0.29 | 1.67 | 1.05 |
| Prdx5 | Rattus norvegicus peroxiredoxin 5 | ***11*** | 0.08 | 1.68 | 0.85 |
| LOC364381 | Rattus norvegicus similar to ribosomal protein S24 | ***11*** | 0.27 | 1.51 | 0.87 |
| LOC287132 | Rattus norvegicus similar to U1 snRNP-specific protein C | ***11*** | -0.26 | 1.6 | 0.36 |
| Tnfrsf12a | Rattus norvegicus tumor necrosis factor receptor superfamily, member 12a | ***11*** | -0.21 | 1.59 | 0.42 |
| Tnpo3 | Rattus norvegicus transportin 3 | ***11*** | 0.03 | 1.54 | 0.37 |
| LOC363441 | Rattus norvegicus similar to NADH dehydrogenase | ***11*** | 0.07 | 1.57 | 0.58 |
| LOC498212 | Rattus norvegicus similar to High mobility group protein 1 | ***11*** | -0.18 | 1.78 | 0.6 |
| Sema3f | Rattus norvegicus sema domain, immunoglobulin domain | ***11*** | -0.09 | 1.78 | 0.52 |
| Ecm1 | Rattus norvegicus extracellular matrix protein 1 | ***11*** | -0.04 | 1.78 | 0.63 |
| Slc38a4 | Rattus norvegicus amino acid transport system A3 | ***11*** | 0.07 | 2.03 | 0.36 |
| Ddah1 | Rattus norvegicus dimethylarginine dimethylaminohydrolase 1 | ***11*** | 0.26 | 1.62 | 0.24 |
| Actr2 | Rattus norvegicus ARP2 actin-related protein 2 homolog | ***11*** | 0.33 | 1.7 | 0.12 |
| Tead3 | Rattus norvegicus TEA domain family member 3 | ***11*** | 0.37 | 1.66 | 0.14 |
| MGC72942 | Rattus norvegicus similar to CG6105-PA | ***11*** | 0.11 | 1.8 | 0.64 |
| Serpinf1 | Rattus norvegicus serine | ***11*** | 0.31 | 1.81 | 0.6 |
| LOC499660 | Rattus norvegicus similar to Cornifin alpha | ***11*** | 0.36 | 1.87 | 0.66 |
| Commd3 | Rattus norvegicus Unknown | ***11*** | 0.48 | 1.68 | 0.56 |
| Lcn2 | Rattus norvegicus lipocalin 2 | ***11*** | -0.68 | 4.34 | 1.51 |
| Grem1 | Rattus norvegicus gremlin 1 homolog, cysteine knot superfamily | ***11*** | -0.52 | 4.07 | 1.07 |
| LOC292273 | Rattus norvegicus similar to Hnrpa3 protein | ***11*** | -0.06 | 2.89 | 1.38 |
| LOC314434 | Rattus norvegicus similar to 60S ribosomal protein L9 | ***11*** | 0.41 | 2.99 | 1.19 |
| Fbln2 | Rattus norvegicus fibulin 2 | ***11*** | 0.42 | 2.68 | 1.36 |
| Ass | Rattus norvegicus argininosuccinate synthetase | ***11*** | 0.41 | 3.11 | 2.08 |
| Gstp2 | Rattus norvegicus glutathione S-transferase, pi 2 | ***11*** | -1.42 | 4.32 | -0.9 |
| LOC500721 | Rattus norvegicus LOC500721 | ***11*** | -1.17 | 3.88 | 0.07 |
| S100a6 | Rattus norvegicus S100 calcium binding protein A6 | ***11*** | -0.58 | 3.51 | -0.16 |
| S100a4 | Rattus norvegicus S100 calcium-binding protein A4 | ***11*** | 0.29 | 4.13 | 0.22 |
|  |  |  |  |  |  |
| LOC498062 | Rattus norvegicus similar to RIKEN cDNA 1190017O12 | ***19*** | -0.3 | 1.91 | -0.29 |
| Rps7 | Rattus norvegicus ribosomal protein S7 | ***19*** | -0.2 | 1.94 | -0.04 |
| RGD1310991 | Rattus norvegicus similar to arsenite inducible RNA associated protein | ***19*** | -0.07 | 1.6 | -0.16 |
| F2r | Rattus norvegicus coagulation factor II | ***19*** | -0.06 | 1.66 | -0.19 |
| Sqrdl | Rattus norvegicus sulfide quinone reductase-like | ***19*** | 0 | 1.81 | -0.05 |
| Ppp3ca | Rattus norvegicus protein phosphatase 3, catalytic subunit, alpha isoform | ***19*** | 0.1 | 1.72 | -0.04 |
| Psmb4 | Rattus norvegicus proteasome | ***19*** | 0.19 | 1.72 | -0.25 |
| LOC288146 | Rattus norvegicus similar to heterogeneous nuclear ribonucleoprotein A3 | ***19*** | 0.23 | 1.77 | -0.26 |
| Myl9 | Rattus norvegicus myosin, light polypeptide 9, regulatory | ***19*** | 0.14 | 1.64 | -0.65 |
| LOC305887 | Rattus norvegicus similar to CG11030-PA | ***19*** | 0.63 | 1.68 | -0.44 |
| Fgfr3 | Rattus norvegicus fibroblast growth factor receptor 3 | ***19*** | 0.67 | 1.87 | -0.79 |
| LOC502854 | Rattus norvegicus similar to ribosomal protein L31 | ***19*** | 0.21 | 2.22 | -0.37 |
| Lgals1 | Rattus norvegicus lectin, galactose binding, soluble 1 | ***19*** | 0.29 | 2.48 | -0.1 |
| LOC501280 | Rattus norvegicus similar to myosin regulatory light chain-like | ***19*** | 0.45 | 2.1 | 0.11 |
| Rab9 | Rattus norvegicus RAB9, member RAS oncogene family | ***19*** | 0.5 | 1.89 | -0.12 |
| Gm1012 | Rattus norvegicus gene model 1012, | ***19*** | 0.53 | 2.25 | -0.2 |
| Cnn1 | Rattus norvegicus calponin 1 | ***19*** | 0.81 | 2.15 | -0.1 |
| LOC500451 | Rattus norvegicus similar to 40S ribosomal protein S20 | ***19*** | 1.13 | 2.33 | -0.19 |
| Ctsl | Rattus norvegicus cathepsin L | ***19*** | 0.97 | 1.86 | 0.02 |
| LOC364828 | Rattus norvegicus similar to 60S ribosomal protein L29 | ***19*** | 1.06 | 1.96 | -0.02 |
| Cst3 | Rattus norvegicus cystatin C | ***19*** | 1.12 | 1.92 | 0.18 |
| Rpl13a | Rattus norvegicus ribosomal protein L13A | ***19*** | 1.31 | 2.04 | 0.03 |
| LOC298126 | Rattus norvegicus similar to ribosomal protein L31 | ***19*** | 1.31 | 2 | 0.03 |
| LOC498360 | Rattus norvegicus similar to ribosomal protein S23 | ***19*** | 1.37 | 2.13 | -0.02 |
| LOC363918 | Rattus norvegicus similar to ribosomal protein L27 | ***19*** | 1.42 | 1.87 | -0.05 |
| Rpl39 | Rattus norvegicus ribosomal protein L39 | ***19*** | 1.31 | 1.84 | 0.28 |
| LOC498523 | Rattus norvegicus similar to 60S ribosomal protein L23a | ***19*** | 1.44 | 1.73 | 0.33 |
| LOC500923 | Rattus norvegicus similar to tumor protein, translationally-controlled 1 | ***19*** | 1.44 | 1.6 | 0.04 |
| LOC364139 | Rattus norvegicus similar to ribosomal protein L21 | ***19*** | 1.11 | 1.56 | -0.29 |
| Rps23 | Rattus norvegicus ribosomal protein S23 | ***19*** | 1.13 | 1.53 | -0.44 |
| LOC362290 | Rattus norvegicus similar to 60S ribosomal protein L7a | ***19*** | 1.51 | 1.35 | -0.42 |
| Rpl27 | Rattus norvegicus ribosomal protein L27 | ***19*** | 1.28 | 1.83 | -0.34 |
| LOC499305 | Rattus norvegicus similar to Finkel-Biskis-Reilly murine sarcoma virus | ***19*** | 1.51 | 1.81 | -0.27 |
| LOC497813 | Rattus norvegicus similar to ribosomal protein S7 | ***19*** | 1.49 | 1.72 | -0.67 |
| Rps3 | Rattus norvegicus ribosomal protein S3 | ***19*** | 1.75 | 1.65 | -0.45 |
| Rps25 | Rattus norvegicus ribosomal protein s25 | ***19*** | 1.59 | 2.07 | -0.52 |
| LOC498555 | Rattus norvegicus similar to 60S acidic ribosomal protein P2 | ***19*** | 1.65 | 1.97 | -0.66 |
| Rps20 | Rattus norvegicus ribosomal protein S20 | ***19*** | 1.77 | 2.01 | -0.59 |
| LOC503110 | Rattus norvegicus similar to ribosomal protein S19 | ***19*** | 1.53 | 1.34 | 0.05 |
| LOC367398 | Rattus norvegicus similar to 60S ribosomal protein L17 | ***19*** | 1.7 | 1.4 | 0.07 |
| Rpl6 | Rattus norvegicus ribosomal protein L6 | ***19*** | 1.76 | 1.36 | 0.01 |
| Rpl7a | Rattus norvegicus ribosomal protein L7a | ***19*** | 1.7 | 1.42 | 0.27 |
| LOC501980 | Rattus norvegicus similar to 40S ribosomal protein S19 | ***19*** | 1.81 | 1.21 | 0.16 |
| Npm1 | Rattus norvegicus nucleophosmin 1 | ***19*** | 1.63 | 0.94 | -0.06 |
| Rps5 | Rattus norvegicus ribosomal protein S5 | ***19*** | 1.7 | 1.12 | -0.13 |
| Rpl17 | Rattus norvegicus ribosomal protein L17 | ***19*** | 1.73 | 1.04 | -0.28 |
| LOC315642 | Rattus norvegicus similar to ribosomal protein L27a | ***19*** | 1.64 | 2.28 | -0.03 |
| LOC307135 | Rattus norvegicus similar to ribosomal protein L34 | ***19*** | 1.71 | 2.19 | -0.02 |
| LOC289715 | Rattus norvegicus similar to ribosomal protein L37 | ***19*** | 1.87 | 2.17 | 0.05 |
| Rpl26 | Rattus norvegicus ribosomal protein L26 | ***19*** | 1.86 | 2.44 | 0.34 |
| Rps8 | Rattus norvegicus ribosomal protein S8 | ***19*** | 1.95 | 2.5 | 0.04 |
| Rps27a | Rattus norvegicus ribosomal protein S27a | ***19*** | 2.11 | 2.57 | -0.01 |
| LOC362181 | Rattus norvegicus similar to Ac2-210 | ***19*** | 1.94 | 2.45 | -0.83 |
| Hnrpdl | Rattus norvegicus heterogeneous nuclear ribonucleoprotein D-like | ***19*** | 2.13 | 2.29 | -0.68 |
| Rpl37a | Rattus norvegicus ribosomal protein L37a | ***19*** | 2.06 | 2.15 | -0.4 |
| Rps27 | Rattus norvegicus ribosomal protein S27 | ***19*** | 2.26 | 2.23 | -0.15 |
| LOC367822 | Rattus norvegicus similar to ribosomal protein L5 | ***19*** | 0.58 | 2.85 | 0.22 |
| LOC289401 | Rattus norvegicus similar to ribosomal protein L31 | ***19*** | 0.93 | 2.7 | 0.54 |
| Rpl35a | Rattus norvegicus ribosomal protein L35a | ***19*** | 1.2 | 2.77 | 0.41 |
| LOC498744 | Rattus norvegicus similar to ribosomal protein L37 | ***19*** | 1.03 | 3.02 | -0.01 |
| Rps17 | Rattus norvegicus ribosomal protein S17 | ***19*** | 1.5 | 3.12 | 0.15 |
| LOC500817 | Rattus norvegicus similar to 40S ribosomal protein S20 | ***19*** | 0.97 | 3.18 | 0.94 |
| LOC299935 | Rattus norvegicus similar to ribosomal protein L31 | ***19*** | 1.52 | 3.09 | 0.81 |
| Eef1b2 | Rattus norvegicus eukaryotic translation elongation factor 1 beta 2 | ***19*** | 0.61 | 2.2 | 0.52 |
| LOC363861 | Rattus norvegicus similar to 60S ribosomal protein L29 | ***19*** | 0.71 | 2.16 | 0.67 |
| LOC501604 | Rattus norvegicus similar to 60S ribosomal protein L7a | ***19*** | 0.96 | 2.06 | 0.4 |
| Btf3 | Rattus norvegicus basic transcription factor 3 | ***19*** | 0.99 | 2.12 | 0.14 |
| Rps9 | Rattus norvegicus ribosomal protein S9 | ***19*** | 1.03 | 2.19 | 0.54 |
| Ppp1r14a | Rattus norvegicus protein phosphatase 1, regulatory | ***19*** | 1.06 | 2.2 | 0.66 |
| LOC366887 | Rattus norvegicus similar to ribosomal protein L31 | ***19*** | 1.15 | 1.97 | 0.5 |
| Tpm4 | Rattus norvegicus tropomyosin 4 | ***19*** | 1.19 | 1.98 | 0.63 |
| Sui1-rs1 | Rattus norvegicus suppressor of initiator codon mutations, related sequence 1 | ***19*** | 1.28 | 2.46 | 0.67 |
| LOC363418 | Rattus norvegicus similar to Ac2-210 | ***19*** | 1.46 | 2.46 | 0.76 |
| LOC364108 | Rattus norvegicus similar to ribosomal protein S17 | ***19*** | 1.56 | 2.74 | 0.64 |
| LOC499133 | Rattus norvegicus similar to 60S ribosomal protein L27a | ***19*** | 1.6 | 2.28 | 0.88 |
| LOC500559 | Rattus norvegicus similar to 40S ribosomal protein S20 | ***19*** | 1.64 | 2.57 | 0.94 |
| Cfl1 | Rattus norvegicus cofilin 1 | ***19*** | 1.76 | 2.51 | 1.36 |
| LOC314556 | Rattus norvegicus similar to ribosomal protein S18 | ***19*** | 1.89 | 2.49 | 1.21 |
| Rpl10 | Rattus norvegicus ribosomal protein L10 | ***19*** | 1.96 | 2.45 | 0.92 |
| Rpl19 | Rattus norvegicus ribosomal protein L19 | ***19*** | 2.07 | 2.65 | 0.99 |
| Fau | Rattus norvegicus Finkel-Biskis-Reilly murine sarcoma virusubiquitously expressed | ***19*** | 1.76 | 2.8 | 0.42 |
| LOC302528 | Rattus norvegicus similar to 60S ribosomal protein L37a | ***19*** | 1.76 | 2.95 | 0.21 |
| LOC498998 | Rattus norvegicus similar to 60S ribosomal protein L26 | ***19*** | 2.08 | 3.25 | 0.71 |
|  |  |  |  |  |  |
| LOC300802 | Rattus norvegicus similar to APH1B homolog | ***30*** | 0 | 1.54 | -1.21 |
| Smad1 | Rattus norvegicus MAD homolog 1 | ***30*** | 0.62 | 1.35 | -1.54 |
| Sat | Rattus norvegicus spermidine/spermine N1-acetyl transferase | ***30*** | 0.65 | 1.63 | -1.38 |
| Gclc | Rattus norvegicus glutamate-cysteine ligase, catalytic subunit | ***30*** | 1.22 | 2.22 | -1.47 |
| Slfn3 | Rattus norvegicus schlafen 3 | ***30*** | 0.23 | 0.84 | -1.75 |
| LOC499554 | Rattus norvegicus similar to ORF2 consensus sequence encoding endonuclease and reverse transcriptase minus RNaseH | ***30*** | 0.29 | 1.03 | -1.98 |
| LOC498644 | Rattus norvegicus similar to Ac1-163 | ***30*** | 0.51 | 0.73 | -2.02 |
| LOC499564 | Rattus norvegicus similar to LRRGT00057 | ***30*** | 0.58 | 0.72 | -2.22 |
| LOC500829 | Rattus norvegicus similar to ORF2 consensus sequence encoding endonuclease and reverse transcriptase minus RNaseH | ***30*** | 0.52 | 0.79 | -1.64 |
| Ddx47 | Rattus norvegicus DEAD | ***30*** | 0.69 | 0.7 | -1.54 |
| Sod1 | Rattus norvegicus superoxide dismutase 1 | ***30*** | 0.85 | 0.45 | -1.69 |
| Enh | Rattus norvegicus enigma homolog | ***30*** | 1.14 | 0.84 | -1.53 |
| Flnc | Rattus norvegicus filamin C, gamma | ***30*** | 1.27 | 0.72 | -1.55 |
| Actn4 | Rattus norvegicus actinin alpha 4 | ***30*** | 1.23 | 1.26 | -1.53 |
|  |  |  |  |  |  |
| Hes1 | Rattus norvegicus hairy and enhancer of split 1 | ***9*** | -1.19 | 2.48 | 3.94 |
| Mfap4 | Rattus norvegicus microfibrillar-associated protein 4 | ***9*** | 0.13 | 2.67 | 4.49 |
| Rhoa | Rattus norvegicus ras homolog gene family, member A | ***9*** | 0.41 | 2 | 3.36 |
| Fstl3 | Rattus norvegicus follistatin-like 3 | ***9*** | 0.91 | 1.53 | 5.5 |
| LOC310648 | Rattus norvegicus similar to glyceraldehyde-3-phosphate dehydrogenase | ***9*** | 1.77 | 1.39 | 3.25 |
| Cmkor1 | Rattus norvegicus chemokine orphan receptor 1 | ***9*** | 1.85 | 1.7 | 3.49 |
| Eno1 | Rattus norvegicus enolase 1, alpha | ***9*** | 2.09 | 1.38 | 3.89 |
| Ptgis | Rattus norvegicus prostaglandin I2 | ***9*** | 1.96 | 3.37 | 4.14 |
|  |  |  |  |  |  |
| MGC94782 | Rattus norvegicus similar to hypothetical protein MGC33926 | ***14*** | -0.2 | 1.35 | 1.71 |
| RGD1307627 | Rattus norvegicus similar to gp25L2 protein | ***14*** | -0.09 | 1.5 | 1.59 |
| Psmc3 | Rattus norvegicus proteasome | ***14*** | -0.04 | 1.24 | 1.51 |
| Ctsd | Rattus norvegicus cathepsin D | ***14*** | 0.16 | 1.06 | 1.81 |
| LOC363865 | Rattus norvegicus similar to tumor protein, translationally-controlled 1 | ***14*** | 0.21 | 1.2 | 1.93 |
| LOC289930 | Rattus norvegicus similar to tumor protein, translationally-controlled 1 | ***14*** | 0.49 | 1.14 | 1.91 |
| Slc1a3 | Rattus norvegicus solute carrier family 1 | ***14*** | 0.63 | 1.05 | 1.79 |
| Slc37a4 | Rattus norvegicus solute carrier family 37 | ***14*** | 0.34 | 1.47 | 1.77 |
| Tex264 | Rattus norvegicus testis expressed gene 264 homolog | ***14*** | 0.55 | 1.38 | 1.87 |
| Arpc1b | Rattus norvegicus actin related protein 2/3 complex, subunit 1B | ***14*** | 0.43 | 1.7 | 1.64 |
| LOC300278 | Rattus norvegicus similar to 40S ribosomal protein S9 | ***14*** | 0.49 | 1.58 | 1.81 |
| LOC302497 | Rattus norvegicus similar to ribosomal protein L10a | ***14*** | 0.56 | 1.6 | 1.68 |
| LOC310512 | Rattus norvegicus similar to expressed sequence C87860 | ***14*** | 0.21 | 1.73 | 2.32 |
| Akr1a1 | Rattus norvegicus aldo-keto reductase family 1, member A1 | ***14*** | 0.21 | 1.86 | 2.13 |
| LOC294781 | Rattus norvegicus similar to 60S ribosomal protein L21 | ***14*** | 0.58 | 1.94 | 1.82 |
| Bgn | Rattus norvegicus biglycan | ***14*** | 0.75 | 2.06 | 2.06 |
| Crabp1 | Rattus norvegicus cellular retinoic acid binding protein I | ***14*** | 0.12 | 1.09 | 2.63 |
| Fbl | Rattus norvegicus fibrillarin | ***14*** | 0.14 | 1.08 | 2.44 |
| Mapre3 | Rattus norvegicus microtubule-associated protein, RP/EB family, member 3 | ***14*** | 0.18 | 1.12 | 2.36 |
| LOC500666 | Rattus norvegicus similar to dapper 1 | ***14*** | 0.32 | 1.01 | 2.4 |
| 0610031j06rik | Rattus norvegicus kidney predominant protein NCU-G1 | ***14*** | 0.35 | 1.29 | 2.12 |
| Mfge8 | Rattus norvegicus milk fat globule-EGF factor 8 protein | ***14*** | 0.61 | 1.28 | 2.36 |
| LOC366689 | Rattus norvegicus similar to ribosomal protein L21 | ***14*** | 1.03 | 0.71 | 1.71 |
| LOC499512 | Rattus norvegicus similar to ATP synthase, H+ transporting, mitochondrial F0 complex, subunit c | ***14*** | 1.28 | 0.75 | 1.55 |
| LOC497882 | Rattus norvegicus similar to ribosomal protein S10 | ***14*** | 1.36 | 0.73 | 1.67 |
| Gabarap | Rattus norvegicus gamma-aminobutyric acid receptor associated protein | ***14*** | 1.33 | 0.61 | 1.77 |
| LOC365416 | Rattus norvegicus similar to ribosomal protein L21 | ***14*** | 1.12 | 0.99 | 1.57 |
| Cox6a1 | Rattus norvegicus cytochrome c oxidase, subunit VIa, polypeptide 1 | ***14*** | 1.31 | 1.25 | 1.55 |
| Pbp | Rattus norvegicus phosphatidylethanolamine binding protein | ***14*** | 1.38 | 1.1 | 1.54 |
| Rpl13 | Rattus norvegicus ribosomal protein L13 | ***14*** | 1.41 | 1.17 | 1.7 |
| Rps2 | Rattus norvegicus ribosomal protein S2 | ***14*** | 1.59 | 0.94 | 1.8 |
| Rpl24 | Rattus norvegicus ribosomal protein L24 | ***14*** | 1.61 | 1 | 1.89 |
| LOC363531 | Rattus norvegicus similar to 40S ribosomal protein S19 | ***14*** | 1.75 | 0.95 | 1.64 |
| Apoe | Rattus norvegicus apolipoprotein E | ***14*** | 1.45 | -0.07 | 1.63 |
| LOC497831 | Rattus norvegicus hypothetical gene supported by NM_175869 | ***14*** | 1.46 | 0.11 | 1.95 |
| Tm4sf9 | Rattus norvegicus transmembrane 4 superfamily member 9 | ***14*** | 1.61 | 0.14 | 1.75 |
| Eif3s5 | Rattus norvegicus eukaryotic translation initiation factor 3, subunit 5 | ***14*** | 1.58 | 0.43 | 1.68 |
| Calr | Rattus norvegicus calreticulin | ***14*** | 1.65 | 0.34 | 1.85 |
| Rpl36 | Rattus norvegicus ribosomal protein L36 | ***14*** | 1.89 | 0.62 | 1.48 |
| Hspa8 | Rattus norvegicus heat shock protein 8 | ***14*** | 1.9 | 0.68 | 1.86 |
| LOC501605 | Rattus norvegicus similar to 40S ribosomal protein S2 | ***14*** | 1.48 | 0.89 | 2.98 |
| Bsg | Rattus norvegicus basigin | ***14*** | 1.58 | 0.69 | 2.16 |
| LOC310585 | Rattus norvegicus similar to Eno1 protein | ***14*** | 2.07 | 0.71 | 2.42 |
| LOC500242 | Rattus norvegicus similar to Poly | ***14*** | 2.25 | 0.5 | 2.25 |
| LOC364343 | Rattus norvegicus similar to RIKEN cDNA A430107P09 gene | ***14*** | 0.3 | 2.05 | 1.14 |
| Rgs14 | Rattus norvegicus regulator of G-protein signaling 14 | ***14*** | 0.4 | 2.06 | 1.36 |
| LOC292539 | Rattus norvegicus similar to 60S ribosomal protein L17 | ***14*** | 0.54 | 2.18 | 1.37 |
| Igf2 | Rattus norvegicus insulin-like growth factor 2 | ***14*** | 0.52 | 1.92 | 1.39 |
| Tgfb3 | Rattus norvegicus transforming growth factor, beta 3 | ***14*** | 0.7 | 2.3 | 1.36 |
| Gmps | Rattus norvegicus guanine monphosphate synthetase | ***14*** | 0.71 | 2.31 | 1.19 |
| Nbl1 | Rattus norvegicus neuroblastoma, suppression of tumorigenicity 1 | ***14*** | 0.76 | 2.37 | 1.27 |
| LOC307416 | Rattus norvegicus similar to Rpl7a protein | ***14*** | 0.66 | 1.78 | 1.05 |
| Plcg1 | Rattus norvegicus phospholipase C, gamma 1 | ***14*** | 0.82 | 1.71 | 0.78 |
| LOC498828 | Rattus norvegicus similar to ribosomal protein L10 | ***14*** | 0.98 | 1.88 | 1.14 |
| Rps13 | Rattus norvegicus ribosomal protein S13 | ***14*** | 1 | 1.92 | 0.91 |
| Eef1d | Rattus norvegicus eukaryotic translation elongation factor 1 delta | ***14*** | 1.07 | 1.56 | 0.93 |
| LOC498293 | Rattus norvegicus similar to basic transcription factor 3 | ***14*** | 1.08 | 1.62 | 1.13 |
| LOC366258 | Rattus norvegicus similar to 60S ribosomal protein L7a | ***14*** | 0.8 | 1.26 | 1.51 |
| Emp3 | Rattus norvegicus epithelial membrane protein 3 | ***14*** | 1.02 | 1.3 | 1.5 |
| LOC301299 | Rattus norvegicus similar to ribosomal protein L10a | ***14*** | 1.05 | 1.53 | 1.38 |
| Lxn | Rattus norvegicus latexin | ***14*** | 0.9 | 1.53 | 1.78 |
| Atf5 | Rattus norvegicus activating transcription factor 5 | ***14*** | 0.95 | 1.59 | 1.79 |
| LOC298495 | Rattus norvegicus similar to ribosomal protein L35a | ***14*** | 1 | 1.72 | 1.62 |
| Gdi2 | Rattus norvegicus GDP dissociation inhibitor 2 | ***14*** | 1.02 | 1.55 | 1.6 |
| Serpine1 | Rattus norvegicus serine | ***14*** | 0.67 | 2.86 | 1.54 |
| Pdlim7 | Rattus norvegicus PDZ and LIM domain 7 | ***14*** | 0.68 | 2.58 | 1.63 |
| Rps12 | Rattus norvegicus ribosomal protein S12 | ***14*** | 0.82 | 2.44 | 0.96 |
| S100a11 | Rattus norvegicus S100 calcium binding protein A11 | ***14*** | 1.15 | 2.74 | 1.09 |
| LOC501203 | Rattus norvegicus similar to Myosin regulatory light chain 2-A, smooth muscle isoform | ***14*** | 1.29 | 2.44 | 1.61 |
| LOC299041 | Rattus norvegicus similar to 60S acidic ribosomal protein P1 | ***14*** | 1.53 | 2.5 | 1.36 |
| LOC366485 | Rattus norvegicus similar to ribosomal protein L36 | ***14*** | 1.16 | 1.88 | 1.67 |
| LOC310360 | Rattus norvegicus similar to eukaryotic translation elongation factor 1 alpha 1 | ***14*** | 1.29 | 1.91 | 1.94 |
| Rpl10a | Rattus norvegicus ribosomal protein L10A | ***14*** | 1.42 | 1.79 | 1.84 |
| LOC366656 | Rattus norvegicus similar to ribosomal protein L10a | ***14*** | 1.44 | 1.94 | 1.78 |
| Nfkbia | Rattus norvegicus nuclear factor of kappa light chain gene enhancer in B-cells inhibitor, alpha | ***14*** | 1.23 | 2.26 | 2.06 |
| LOC366999 | Rattus norvegicus similar to 60S ribosomal protein L29 | ***14*** | 1.03 | 1.72 | 2.26 |
| LOC367339 | Rattus norvegicus similar to 60S ribosomal protein L29 | ***14*** | 1.1 | 1.4 | 2.37 |
| LOC498078 | Rattus norvegicus similar to 60S ribosomal protein L7a | ***14*** | 1.08 | 1.27 | 1.98 |
| LOC302388 | Rattus norvegicus similar to ribosomal protein L19 | ***14*** | 1.12 | 1.92 | 2.54 |
| LOC500645 | Rattus norvegicus similar to 60S ribosomal protein L29 | ***14*** | 1.15 | 1.74 | 2.64 |
| LOC301438 | Rattus norvegicus similar to 40S ribosomal protein S2 | ***14*** | 1.25 | 1.38 | 2.87 |
| LOC498068 | Rattus norvegicus similar to ribosomal protein S2 | ***14*** | 1.4 | 1.48 | 2.64 |
| LOC296582 | Rattus norvegicus similar to ribosomal protein S2 | ***14*** | 1.7 | 0.93 | 2.42 |
| Cd63 | Rattus norvegicus CD63 antigen | ***14*** | 1.82 | 1.09 | 2.15 |
| P4hb | Rattus norvegicus prolyl 4-hydroxylase, beta polypeptide | ***14*** | 1.86 | 1.26 | 2.49 |
| LOC294700 | Rattus norvegicus similar to ribosomal protein L21 | ***14*** | 1.78 | 1.61 | 2.25 |
| Ubb | Rattus norvegicus polyubiquitin | ***14*** | 2.24 | 1.56 | 2.23 |
| Rpl18 | Rattus norvegicus ribosomal protein L18 | ***14*** | 1.76 | 1.6 | 1.48 |
| LOC361061 | Rattus norvegicus hypothetical LOC361061 | ***14*** | 1.85 | 1.77 | 1.34 |
| LOC500929 | Rattus norvegicus similar to Tubulin alpha-2 chain | ***14*** | 1.9 | 1.73 | 1.45 |
| Rps11 | Rattus norvegicus ribosomal protein S11 | ***14*** | 2.05 | 1.86 | 1.45 |
| LOC502629 | Rattus norvegicus similar to 60S ribosomal protein L9 | ***14*** | 1.93 | 1.91 | 1.76 |
| LOC299622 | Rattus norvegicus similar to glyceraldehyde-3-phosphate dehydrogenase | ***14*** | 2.39 | 1.85 | 1.92 |
| LOC502063 | Rattus norvegicus LOC502063 | ***14*** | 2.6 | 1.76 | 1.88 |
| Lamr1 | Rattus norvegicus laminin receptor 1 | ***14*** | 2.65 | 2.1 | 1.92 |
| Cstb | Rattus norvegicus cystatin B | ***14*** | 1.97 | 1.19 | 1.39 |
| LOC291308 | Rattus norvegicus similar to ribosomal protein L21 | ***14*** | 2.11 | 1.1 | 1.46 |
| LOC295439 | Rattus norvegicus similar to ribosomal protein L21 | ***14*** | 2.11 | 1.26 | 1.48 |
| LOC499423 | Rattus norvegicus similar to pyruvate kinase | ***14*** | 2.09 | 1.08 | 1.71 |
| Rpl32 | Rattus norvegicus ribosomal protein L32 | ***14*** | 2.13 | 0.84 | 1.55 |
| LOC499845 | Rattus norvegicus similar to ribosomal protein L21 | ***14*** | 2.17 | 0.9 | 1.66 |
| Rpl21 | Rattus norvegicus ribosomal protein L21 | ***14*** | 2.24 | 1.41 | 1.6 |
| LOC306115 | Rattus norvegicus similar to glyceraldehyde-3-phosphate dehydrogenase | ***14*** | 2.46 | 1.32 | 1.77 |
| Rps10 | Rattus norvegicus ribosomal protein S10 | ***14*** | 2.52 | 1.33 | 1.78 |
| LOC367102 | Rattus norvegicus similar to 40S ribosomal protein S9 | ***14*** | 1.42 | 1.7 | 0.57 |
| Rps15 | Rattus norvegicus ribosomal protein S15 | ***14*** | 1.51 | 1.53 | 0.43 |
| Rpl35 | Rattus norvegicus ribosomal protein L35 | ***14*** | 1.78 | 1.73 | 0.34 |
| LOC499457 | Rattus norvegicus similar to 60S ribosomal protein L7a | ***14*** | 1.8 | 1.7 | 0.51 |
| LOC294748 | Rattus norvegicus similar to Chain , Human Translation Initiation Factor Eif1, Nmr, 29 Structures | ***14*** | 1.87 | 1.72 | 0.54 |
| LOC502302 | Rattus norvegicus similar to 40S ribosomal protein S19 | ***14*** | 1.89 | 1.91 | 0.45 |
| LOC499852 | Rattus norvegicus similar to 60S ribosomal protein L7a | ***14*** | 1.47 | 2.06 | 0.53 |
| Rpl8 | Rattus norvegicus ribosomal protein L8 | ***14*** | 1.53 | 1.99 | 0.47 |
| LOC309408 | Rattus norvegicus similar to ribosomal protein S12 | ***14*** | 1.75 | 2.2 | 0.47 |
| LOC293860 | Rattus norvegicus similar to Filamin A | ***14*** | 1.96 | 1.78 | 0.19 |
| LOC361797 | Rattus norvegicus hypothetical LOC361797 | ***14*** | 2.26 | 1.87 | 0.26 |
| Rps14 | Rattus norvegicus ribosomal protein S14 | ***14*** | 2.19 | 1.68 | -0.02 |
| LOC499906 | Rattus norvegicus similar to Eukaryotic translation initiation factor 1 | ***14*** | 2.25 | 1.6 | 0.17 |
| Uba52 | Rattus norvegicus ubiquitin A-52 residue ribosomal protein fusion product 1 | ***14*** | 1.55 | 1.32 | 0.94 |
| Icam1 | Rattus norvegicus intercellular adhesion molecule 1 | ***14*** | 1.99 | 1.55 | 1.11 |
| LOC302445 | Rattus norvegicus similar to 60S ribosomal protein L32 | ***14*** | 2.03 | 1.4 | 1.14 |
| LOC499803 | Rattus norvegicus similar to 40S ribosomal protein S3 | ***14*** | 2.07 | 1.26 | 1.12 |
| LOC503284 | Rattus norvegicus similar to ribosomal protein L19 | ***14*** | 2.1 | 1.22 | 0.89 |
| Pabpc1 | Rattus norvegicus poly | ***14*** | 2.17 | 1.23 | 0.92 |
| LOC293642 | Rattus norvegicus similar to ribosomal protein L21 | ***14*** | 2.26 | 1.28 | 1.06 |
| Ppia | Rattus norvegicus peptidylprolyl isomerase A | ***14*** | 1.81 | 1.81 | 1.02 |
| LOC500859 | Rattus norvegicus similar to 60S ribosomal protein L7a | ***14*** | 2.01 | 1.81 | 0.95 |
| LOC366193 | Rattus norvegicus similar to 40S ribosomal protein S3a | ***14*** | 2 | 2.13 | 0.98 |
| LOC298785 | Rattus norvegicus similar to ribosomal protein S26 | ***14*** | 2.08 | 2.28 | 0.89 |
| Myd116 | Rattus norvegicus myeloid differentiation primary response gene 116 | ***14*** | 2.14 | 2.29 | 0.57 |
| LOC500885 | Rattus norvegicus similar to 40S ribosomal protein S19 | ***14*** | 2.15 | 2 | 0.71 |
| Rplp1 | Rattus norvegicus ribosomal protein, large, P1 | ***14*** | 2.24 | 1.89 | 0.99 |
| Rpl29 | Rattus norvegicus ribosomal protein L29 | ***14*** | 2.34 | 1.96 | 1.22 |
| Slc3a2 | Rattus norvegicus solute carrier family 3 | ***14*** | 2.39 | 1.65 | 1.02 |
| Rps18 | Rattus norvegicus ribosomal protein S18 | ***14*** | 2.45 | 1.83 | 1.09 |
| Rpl41 | Rattus norvegicus ribosomal protein L41 | ***14*** | 2.6 | 1.93 | 0.89 |
| LOC303815 | Rattus norvegicus similar to 60S ribosomal protein L12 | ***14*** | 1.82 | 1.3 | 0.68 |
| Gnl3 | Rattus norvegicus guanine nucleotide binding protein-like 3 | ***14*** | 2.04 | 1.34 | 0.73 |
| Rpl9 | Rattus norvegicus ribosomal protein L9 | ***14*** | 1.98 | 1.13 | 0.74 |
| Rpl14 | Rattus norvegicus ribosomal protein L14 | ***14*** | 2.19 | 1.07 | 0.66 |
| Ndel1 | Rattus norvegicus nudE nuclear distribution gene E homolog like 1 | ***14*** | 1.96 | 1.29 | 0.5 |
| LOC296870 | Rattus norvegicus similar to ribosomal protein L34 | ***14*** | 2.17 | 1.28 | 0.36 |
| Rpl27a | Rattus norvegicus ribosomal protein L27a | ***14*** | 2.23 | 1.25 | 0.42 |
| LOC500669 | Rattus norvegicus similar to 60S ribosomal protein L7a | ***14*** | 2.22 | 0.99 | 0.37 |
| LOC310365 | Rattus norvegicus similar to ribosomal protein L21 | ***14*** | 2.21 | 0.94 | 1 |
| Tpt1 | Rattus norvegicus tumor protein, translationally-controlled 1 | ***14*** | 2.23 | 0.87 | 0.96 |
| LOC361026 | Rattus norvegicus similar to 60S ribosomal protein L7a | ***14*** | 2.56 | 0.98 | 1.13 |
| Lgals3 | Rattus norvegicus lectin, galactose binding, soluble 3 | ***14*** | 2.33 | 0.73 | 0.71 |
| Car9 | Rattus norvegicus carbonic anhydrase 9 | ***14*** | 2.73 | 0.8 | 0.58 |
| Fn1 | Rattus norvegicus fibronectin 1 | ***14*** | 2.3 | 1.52 | 0.43 |
| LOC304035 | Rattus norvegicus similar to 60S ribosomal protein L7a | ***14*** | 2.4 | 1.52 | 0.63 |
| LOC500547 | Rattus norvegicus similar to 60S ribosomal protein L37a | ***14*** | 2.44 | 1.47 | 0.5 |
| LOC498143 | Rattus norvegicus similar to ribosomal protein L15 | ***14*** | 2.47 | 1.56 | 0.52 |
| LOC501206 | Rattus norvegicus similar to 60S ribosomal protein L5 | ***14*** | 2.68 | 1.4 | 0.78 |
| LOC298169 | Rattus norvegicus similar to actin alpha 1 skeletal muscle protein | ***14*** | 2.81 | 1.61 | 0.47 |
| Gnai2 | Rattus norvegicus guanine nucleotide binding protein, alpha inhibiting 2 | ***14*** | 1.5 | 0.61 | 0.98 |
| Gsk3b | Rattus norvegicus glycogen synthase kinase 3 beta | ***14*** | 1.53 | 0.89 | 0.92 |
| Pttg1ip | Rattus norvegicus pituitary tumor-transforming 1 interacting protein | ***14*** | 1.6 | 0.84 | 1.3 |
| LOC361613 | Rattus norvegicus similar to Protein phosphatase methylesterase 1 | ***14*** | 1.63 | 0.26 | 1.11 |
| LOC498661 | Rattus norvegicus similar to pyruvate kinase | ***14*** | 1.66 | 0.19 | 0.99 |
| Ndfip2 | Rattus norvegicus Nedd4 family interacting protein 2 | ***14*** | 1.76 | 0.6 | 0.88 |
| LOC295472 | Rattus norvegicus similar to ribosomal protein L21 | ***14*** | 1.92 | 0.43 | 0.85 |
| Cd83 | Rattus norvegicus CD83 antigen | ***14*** | 1.53 | 0.33 | 0.44 |
| Hdlbp | Rattus norvegicus high density lipoprotein binding protein | ***14*** | 1.58 | 0.43 | 0.61 |
| Atp5b | Rattus norvegicus ATP synthase, H+ transporting, mitochondrial F1 complex, beta polypeptide | ***14*** | 1.58 | 0.51 | 0.47 |
| MGC94937 | Rattus norvegicus similar to RIKEN cDNA 1110020M19 | ***14*** | 1.83 | 0.39 | 0.61 |
| Slc25a4 | Rattus norvegicus solute carrier family 25 | ***14*** | 1.53 | 0.86 | 0.34 |
| Rabggtb | Rattus norvegicus RAB geranylgeranyl transferase, b subunit | ***14*** | 1.73 | 0.89 | 0.32 |
| Prss23 | Rattus norvegicus protease, serine, 23 | ***14*** | 1.55 | 0.8 | 0.5 |
| Rps3a | Rattus norvegicus ribosomal protein S3a | ***14*** | 1.79 | 0.86 | 0.57 |
| LOC312363 | Rattus norvegicus similar to 60S ribosomal protein L12 | ***14*** | 1.82 | 0.7 | 0.5 |
| Rps4x | Rattus norvegicus ribosomal protein S4, X-linked | ***14*** | 2.09 | 0.63 | 1.23 |
| Cited2 | Rattus norvegicus Cbp/p300-interacting transactivator, with Glu/Asp-rich carboxy-terminal domain, 2 | ***14*** | 2.11 | 0.55 | 1.06 |
| LOC500660 | Rattus norvegicus similar to ribosomal protein L21 | ***14*** | 2.31 | 0.42 | 1.39 |
| LOC500914 | Rattus norvegicus similar to basic transcription factor 3 | ***14*** | 2.1 | 0.08 | 0.7 |
| Aatf | Rattus norvegicus apoptosis antagonizing transcription factor | ***14*** | 2.16 | 0.21 | 0.88 |
| osr1 | Rattus norvegicus oxidative stress responsive 1 | ***14*** | 2.48 | 0.36 | 0.66 |
| Dctn4 | Rattus norvegicus dynactin 4 | ***14*** | 2.5 | 0.27 | 0.77 |
| LOC309161 | Rattus norvegicus similar to Delta-interacting protein A | ***14*** | 2.4 | 0.02 | 1.24 |
| LOC316842 | Rattus norvegicus similar to cDNA sequence BC019776 | ***14*** | 2.59 | 0 | 1.24 |
| LOC288065 | Rattus norvegicus hypothetical LOC288065 | ***14*** | 2.4 | 0.19 | 0.98 |
|  |  |  |  |  |  |
| Eef1a1 | Rattus norvegicus eukaryotic translation elongation factor 1 alpha 1 | ***26*** | 2.88 | 1.57 | 1.16 |
| LOC498618 | Rattus norvegicus similar to glyceraldehyde-3-phosphate dehydrogenase | ***26*** | 3.27 | 1.65 | 1.62 |
| Actb | Rattus norvegicus actin, beta | ***26*** | 3.39 | 1.84 | 0.84 |
| Actg | Rattus norvegicus actin, gamma, cytoplasmic | ***26*** | 3.49 | 1.74 | 0.8 |
| Ctgf | Rattus norvegicus connective tissue growth factor | ***26*** | 3.91 | 2.15 | 1.16 |
| LOC500983 | Rattus norvegicus similar to glyceraldehyde-3-phosphate dehydrogenase | ***26*** | 4.08 | 2.26 | 1.11 |
| Vim | Rattus norvegicus vimentin | ***26*** | 3.98 | 1.96 | 1.02 |
| Mt1a | Rattus norvegicus Metallothionein | ***26*** | 4.02 | 2.54 | 0.92 |
| Fth1 | Rattus norvegicus ferritin, heavy polypeptide 1 | ***26*** | 4.34 | 1.89 | 0.7 |
| Arbp | Rattus norvegicus acidic ribosomal phosphoprotein P0 | ***26*** | 4.02 | 1.52 | 1.31 |
| Aldoa | Rattus norvegicus aldolase A | ***26*** | 4.35 | 1.6 | 2.01 |
| Ddit3 | Rattus norvegicus DNA-damage inducible transcript 3 | ***26*** | 4.21 | 2.31 | 1.87 |
| Atf4 | Rattus norvegicus activating transcription factor 4 | ***26*** | 4.23 | 2.38 | 1.77 |
| LOC290704 | Rattus norvegicus similar to palladin | ***26*** | 3.39 | 0.95 | 0.37 |
| Tagln | Rattus norvegicus transgelin | ***26*** | 3.4 | 1.58 | 0.35 |
| LOC497684 | Rattus norvegicus hypothetical gene supported by NM_017314 | ***26*** | 3.63 | 1.5 | -0.12 |
| LOC363377 | Rattus norvegicus similar to RIKEN cDNA 2410116I05 | ***26*** | 4.2 | 1.19 | -0.12 |
|  |  |  |  |  |  |
| LOC500104 | Rattus norvegicus similar to Glyceraldehyde-3-phosphate dehydrogenase | ***37*** | 3.08 | 2.17 | 2.7 |
| LOC498019 | Rattus norvegicus similar to glyceraldehyde-3-phosphate dehydrogenase | ***37*** | 3.12 | 2.13 | 2.45 |
| LOC498881 | Rattus norvegicus similar to glyceraldehyde-3-phosphate dehydrogenase | ***37*** | 3.21 | 1.8 | 2.83 |
| LOC499433 | Rattus norvegicus similar to glyceraldehyde-3-phosphate dehydrogenase | ***37*** | 3.53 | 1.92 | 2.72 |
| LOC290634 | Rattus norvegicus similar to Glyceraldehyde-3-phosphate dehydrogenase | ***37*** | 3.65 | 1.9 | 2.49 |
| LOC295452 | Rattus norvegicus similar to Glyceraldehyde-3-phosphate dehydrogenase | ***37*** | 3.67 | 2.15 | 2.25 |
| Gapd | Rattus norvegicus glyceraldehyde-3-phosphate dehydrogenase | ***37*** | 3.81 | 1.41 | 3.03 |
| LOC291715 | Rattus norvegicus similar to L-lactate dehydrogenase A chain | ***37*** | 3.98 | 1.46 | 2.83 |
| LOC364048 | Rattus norvegicus similar to macrophage migration inhibitory factor | ***37*** | 3.87 | 1.23 | 2.77 |
| LOC295423 | Rattus norvegicus similar to glyceraldehyde-3-phosphate dehydrogenase | ***37*** | 3.38 | 2.29 | 3.37 |
| LOC364848 | Rattus norvegicus similar to Glyceraldehyde-3-phosphate dehydrogenase | ***37*** | 3.38 | 2.35 | 3.16 |
| LOC365954 | Rattus norvegicus similar to glyceraldehyde-3-phosphate dehydrogenase | ***37*** | 3.39 | 2.25 | 3.25 |
| LOC498099 | Rattus norvegicus similar to glyceraldehyde-3-phosphate dehydrogenase | ***37*** | 4.05 | 2.59 | 2.73 |
| LOC500271 | Rattus norvegicus similar to macrophage migration inhibitory factor | ***37*** | 4.05 | 2.13 | 2.63 |
| Ldha | Rattus norvegicus lactate dehydrogenase A | ***37*** | 4.16 | 2.05 | 2.65 |
| LOC307731 | Rattus norvegicus similar to L-lactate dehydrogenase A chain | ***37*** | 4.23 | 1.76 | 3.29 |
| Pgk1 | Rattus norvegicus phosphoglycerate kinase 1 | ***37*** | 4.67 | 1.66 | 3.18 |
| LOC500965 | Rattus norvegicus similar to L-lactate dehydrogenase A chain | ***37*** | 4.75 | 2.36 | 3.35 |
|  |  |  |  |  |  |
| Flcn | Rattus norvegicus folliculin | ***5*** | 2.9 | 0.86 | 2.26 |
| LOC502770 | Rattus norvegicus similar to glyceraldehyde-3-phosphate dehydrogenase | ***5*** | 2.96 | 1.13 | 2.2 |
| LOC292656 | Rattus norvegicus similar to Macrophage migration inhibitory factor | ***5*** | 3.53 | 1.35 | 2.34 |
| Gadd45b | Rattus norvegicus growth arrest and DNA-damage-inducible 45 beta | ***5*** | 3.5 | 0.7 | 2.33 |
| Hig1 | Rattus norvegicus hypoxia induced gene 1 | ***5*** | 3.71 | 0.78 | 2.38 |
| Gdf15 | Rattus norvegicus growth differentiation factor 15 | ***5*** | 3.78 | 0.57 | 2.34 |
| LOC500506 | Rattus norvegicus similar to glyceraldehyde-3-phosphate dehydrogenase | ***5*** | 3.77 | 0.71 | 1.93 |
| Ppp1r3c | Rattus norvegicus protein phosphatase 1, regulatory | ***5*** | 3.83 | 0.2 | 2.26 |
| Cyr61 | Rattus norvegicus cysteine rich protein 61 | ***5*** | 2.99 | 0.24 | 1.97 |
| Pfkl | Rattus norvegicus phosphofructokinase, liver, B-type | ***5*** | 3.24 | 0.17 | 1.9 |
| Gpi | Rattus norvegicus glucose phosphate isomerase | ***5*** | 3.32 | 0.4 | 1.67 |
| LOC497927 | Rattus norvegicus similar to Phosphoglycerate mutase 1 | ***5*** | 3.17 | -0.39 | 1.64 |
| LOC499178 | Rattus norvegicus LOC499178 | ***5*** | 3.52 | 0.2 | 1.06 |
| LOC366205 | Rattus norvegicus similar to fibronectin leucine rich transmembrane protein 3 | ***5*** | 3.63 | 0.37 | 0.72 |
| Egln1 | Rattus norvegicus EGL nine homolog 1 | ***5*** | 4.2 | -0.14 | 1.95 |
| Hspb1 | Rattus norvegicus heat shock 27kDa protein 1 | ***5*** | 4.47 | 0.08 | 1.54 |
| Ak3l1 | Rattus norvegicus adenylate kinase 3-like 1 | ***5*** | 4.9 | -0.8 | 2.31 |
| Gadd45g | Rattus norvegicus growth arrest and DNA-damage-inducible 45 gamma | ***5*** | 5.18 | -0.75 | 1.38 |
| Mif | Rattus norvegicus macrophage migration inhibitory factor | ***5*** | 4.36 | 0.42 | 1.25 |
| Insig1 | Rattus norvegicus insulin induced gene 1 | ***5*** | 4.87 | 0.46 | 1.34 |
| LOC501140 | Rattus norvegicus similar to BCL2/adenovirus E1B 19 kDa-interacting protein 3 | ***5*** | 4.67 | 0.65 | 1.64 |
| Bnip3 | Rattus norvegicus BCL2/adenovirus E1B 19 kDa-interacting protein 3 | ***5*** | 5 | 0.44 | 1.82 |
| LOC302402 | Rattus norvegicus similar to hypoxia induced gene 1 | ***5*** | 5.25 | 0.24 | 0.88 |
|  |  |  |  |  |  |
| Pygl | Rattus norvegicus liver glycogen phosphorylase | ***40*** | 3.96 | -0.57 | 3.45 |
| LOC500959 | Rattus norvegicus similar to triosephosphate isomerase | ***40*** | 4.29 | 0.17 | 3.03 |
| LOC498731 | Rattus norvegicus similar to Tpi1 protein | ***40*** | 4.53 | 0.39 | 3.4 |
| Tpi1 | Rattus norvegicus triosephosphate isomerase 1 | ***40*** | 4.62 | 0.61 | 3.66 |
| G0s2 | Rattus norvegicus G0/G1 switch gene 2 | ***40*** | 5.11 | -1.98 | 2.88 |
|  |  |  |  |  |  |
| Cebpb | Rattus norvegicus CCAAT/enhancer binding protein | ***24*** | 4.69 | 0.74 | -0.23 |
| Nr4a3 | Rattus norvegicus nuclear receptor subfamily 4, group A, member 3 | ***24*** | 4.76 | 0.56 | -0.62 |
| Timp3 | Rattus norvegicus tissue inhibitor of metalloproteinase 3 | ***24*** | 4.94 | 0.19 | -0.28 |
| LOC500804 | Rattus norvegicus similar to Ferritin light chain | ***24*** | 5.05 | 1.41 | 0.36 |
| LOC501644 | Rattus norvegicus similar to Ferritin light chain 2 | ***24*** | 5.34 | 1.62 | 0.72 |
| LOC499244 | Rattus norvegicus similar to ferritin light chain | ***24*** | 5.37 | 0.9 | 0.5 |
| Ftl1 | Rattus norvegicus ferritin light chain 1 | ***24*** | 5.41 | 1.18 | 0.57 |
| Pim1 | Rattus norvegicus proviral integration site 1 | ***24*** | 6.45 | 1.34 | -0.02 |
| Dusp8 | Rattus norvegicus dual specificity phosphatase 8 | ***24*** | 5.94 | -0.12 | 0.03 |
| Ptp4a1 | Rattus norvegicus protein tyrosine phosphatase 4a1 | ***24*** | 6.36 | 0.39 | 0.01 |
| Bhlhb2 | Rattus norvegicus basic helix-loop-helix domain containing, class B2 | ***24*** | 6.73 | -0.1 | -0.66 |
| Hk2 | Rattus norvegicus hexokinase 2 | ***24*** | 6.49 | 0.42 | 1.03 |
| Vegfa | Rattus norvegicus vascular endothelial growth factor A | ***24*** | 6.54 | -0.45 | 1.18 |
|  |  |  |  |  |  |
| Slc16a3 | Rattus norvegicus monocarboxylate transporter | ***12*** | 3.78 | 0.34 | 6.25 |
|  |  |  |  |  |  |
| Acta1 | Rattus norvegicus actin, alpha 1, skeletal muscle | ***20*** | 8.58 | 1.87 | 3.52 |
