# Supplementary material for: Multiple Oxygen Tension Environments Reveal Diverse Patterns of Transcriptional Regulation in Primary Astrocytes
Source: PLoS One. 2011 Jun 27;6(6):e21638. doi: 10.1371/journal.pone.0021638 (PMC3124552; doi:10.1371/journal.pone.0021638)
Supplement: Table S13 — Venn diagram analysis of canonical signaling pathway analysis for the four classified group clusters. The four-way Venn diagram describes the relationships between the statistically significant canonical signaling pathways populated by the multiple transcripts contained in the four group clusters, ONE, TWO, THREE, FOUR. The intersection subsets, A to O, of significantly populated signaling pathways are depicted in Figure 7. (DOC) [file pone.0021638.s019.doc]

**Table S13. Venn diagram analysis of canonical signaling pathway analysis for the four classified group clusters.** The four-way Venn diagram describes the relationships between the statistically significant canonical signaling pathways populated by the multiple transcripts contained in the four group clusters, ONE, TWO, THREE, FOUR. The intersection subsets, A to O, of significantly populated signaling pathways are depicted in Figure 7.

| **A** |
| --- |
| Axonal Guidance Signaling |
| Cdc42 Signaling |
| Ceramide Signaling |
| Glutathione Metabolism |
| Induction of Apoptosis by HIV1 |
| LPS/IL-1 Mediated Inhibition of RXR Function |
| LXR/RXR Activation |
| PAK Signaling |
| Rac Signaling |
| Regulation of Actin-based Motility by Rho |
| RhoA Signaling |
| TNFR1 Signaling |
| Xenobiotic Metabolism Signaling |
|  |
| **B** |
| Cell Cycle: G1/S Checkpoint Regulation |
| Fructose and Mannose Metabolism |
| GABA Receptor Signaling |
| Galactose Metabolism |
| Glycolysis/Gluconeogenesis |
| Pentose Phosphate Pathway |
| PPAR Signaling |
| Purine Metabolism |
| RAN Signaling |
| Starch and Sucrose Metabolism |
| Taurine and Hypotaurine Metabolism |
| TGF-β Signaling |
|  |
| **C** |
| Activation of IRF by Cytosolic Pattern Recognition Receptors |
| Angiopoietin Signaling |
| Arachidonic Acid Metabolism |
| Caveolar-mediated Endocytosis Signaling |
| CD40 Signaling |
| Chronic Myeloid Leukemia Signaling |
| Circadian Rhythm Signaling |
| Colorectal Cancer Metastasis Signaling |
| Dendritic Cell Maturation |
| Eicosanoid Signaling |
| Endothelin-1 Signaling |
| Erythropoietin Signaling |
| Estrogen Receptor Signaling |
| Estrogen-Dependent Breast Cancer Signaling |
| FAK Signaling |
| Fatty Acid Elongation in Mitochondria |
| Fcγ Receptor-mediated Phagocytosis in Macrophages and Monocytes |
| Germ Cell-Sertoli Cell Junction Signaling |
| Glioblastoma Multiforme Signaling |
| Glucocorticoid Receptor Signaling |
| IL-17 Signaling |
| IL-8 Signaling |
| LPS-stimulated MAPK Signaling |
| Lymphotoxin β Receptor Signaling |
| Mechanisms of Viral Exit from Host Cells |
| Melanoma Signaling |
| MIF Regulation of Innate Immunity |
| Molecular Mechanisms of Cancer |
| Neurotrophin/TRK Signaling |
| p38 MAPK Signaling |
| p53 Signaling |
| Pancreatic Adenocarcinoma Signaling |
| Prostate Cancer Signaling |
| Role of MAPK Signaling in the Pathogenesis of Influenza |
| Role of PI3K/AKT Signaling in the Pathogenesis of Influenza |
| Role of RIG1-like Receptors in Antiviral Innate Immunity |
| Small Cell Lung Cancer Signaling |
|  |
| **D** |
| Androgen Signaling |
| BMP signaling pathway |
| Breast Cancer Regulation by Stathmin1 |
| Calcium-induced T Lymphocyte Apoptosis |
| cAMP-mediated Signaling |
| Cardiac Hypertrophy Signaling |
| CCR3 Signaling in Eosinophils |
| CCR5 Signaling in Macrophages |
| CD27 Signaling in Lymphocytes |
| CD28 Signaling in T Helper Cells |
| CDK5 Signaling |
| Coagulation System |
| Complement System |
| Corticotropin Releasing Hormone Signaling |
| CREB Signaling in Neurons |
| CXCR4 Signaling |
| Dopamine Receptor Signaling |
| Glutamate Receptor Signaling |
| GNRH Signaling |
| IGF-1 Signaling |
| IL-1 Signaling |
| Keratan Sulfate Biosynthesis |
| Melatonin Signaling |
| Nitric Oxide Signaling in the Cardiovascular System |
| Nur77 Signaling in T Lymphocytes |
| Production of Nitric Oxide and Reactive Oxygen Species in Macrophages |
| PXR/RXR Activation |
| RANK Signaling in Osteoclasts |
| Regulation of IL-2 Expression in Activated and Anergic T Lymphocytes |
| Role of NFAT in Cardiac Hypertrophy |
| Role of NFAT in Regulation of the Immune Response |
| SAPK/JNK Signaling |
| Synaptic Long Term Potentiation |
| T Cell Receptor Signaling |
| α-Adrenergic Signaling |
|  |
| **E** |
| Mitochondrial Dysfunction |
|  |
| **F** |
| ATM Signaling |
| Agrin Interactions at Neuromuscular Junction |
| TR/RXR Activation |
| Cellular Effects of Sildenafil (Viagra) |
| B Cell Receptor Signaling |
|  |
| **G** |
| Clathrin-mediated Endocytosis Signaling |
| Hypoxia Signaling in the Cardiovascular System |
| Virus Entry via Endocytic Pathways |
|  |
| **H** |
| Aryl Hydrocarbon Receptor Signaling |
| Integrin Signaling |
|  |
| **I** |
|  |
| **J** |
| Hepatic Fibrosis / Hepatic Stellate Cell Activation |
| Ubiquinone Biosynthesis |
| Glioma Invasiveness Signaling |
| Actin Cytoskeleton Signaling |
| Protein Ubiquitination Pathway |
| Atherosclerosis Signaling |
|  |
| **K** |
| Tight Junction Signaling |
|  |
| **L** |
| HMGB1 Signaling |
| Leukocyte Extravasation Signaling |
| Role of Macrophages, Fibroblasts and Endothelial Cells in Rheumatoid Arthritis |
|  |
| **M** |
|  |
| **N** |
| N-Glycan Biosynthesis |
| Chemokine Signaling |
| Protein Kinase A Signaling |
| Calcium Signaling |
| Role of Osteoblasts, Osteoclasts and Chondrocytes in Rheumatoid Arthritis |
| RAR Activation |
| Hepatic Cholestasis |
|  |
| **O** |
| NRF2-mediated Oxidative Stress Response |
| ILK Signaling |
