# Supplementary material for: Multiple Oxygen Tension Environments Reveal Diverse Patterns of Transcriptional Regulation in Primary Astrocytes
Source: PLoS One. 2011 Jun 27;6(6):e21638. doi: 10.1371/journal.pone.0021638 (PMC3124552; doi:10.1371/journal.pone.0021638)
Supplement: Table S14 — Physiological relevance of group clusters ONE -, TWO -, THREE - and FOUR -unique signaling pathways. For each canonical signaling pathway significantly populated using the group cluster input genes a hybrid score was created that was the generated by multiplication of the negative log10 of the probability of enrichment of the genes in the respective signaling pathway with the gene enrichment ratio relative to a species-specific background geneset. For each significantly-populated signaling pathway the genes from the input dataset that generated the associated hybrid score are denoted. (DOC) [file pone.0021638.s020.doc]

**Table S14. Physiological relevance of group clusters ONE-, TWO-, THREE- and FOUR-unique signaling pathways.** For each canonical signaling pathway significantly populated using the group cluster input genes a hybrid score was created that was the generated by multiplication of the negative log_10_ of the probability of enrichment of the genes in the respective signaling pathway with the gene enrichment ratio relative to a species-specific background geneset. For each significantly-populated signaling pathway the genes from the input dataset that generated the associated hybrid score are denoted.

| **ONE "unique"** | **Hybrid Score** |
| --- | --- |
| **CYTOSKELETAL DYNAMICS** |  |
| ***Cdc42 Signaling*** | 24.5616 |
| MAP2K4 |  |
| MYL9 (includes EG:10398) |  |
| ACTR2 |  |
| WIPF1 |  |
| CFL1 |  |
| EXOC2 |  |
| ARPC1A |  |
| TNK2 |  |
|  |  |
| ***Regulation of Actin-based Motility by Rho*** | 21.0036 |
| MYL9 (includes EG:10398) |  |
| ACTR2 |  |
| WIPF1 |  |
| RHOQ |  |
| CFL1 |  |
| PFN2 |  |
| ARPC1A |  |
|  |  |
| ***RhoA Signaling*** | 13.992 |
| MYL9 (includes EG:10398) |  |
| ACTR2 |  |
| CFL1 |  |
| CFL2 |  |
| PFN2 |  |
| ARPC1A |  |
| MSN |  |
|  |  |
| ***Rac Signaling*** | 8.5116 |
| MAP2K4 |  |
| ACTR2 |  |
| CFL1 |  |
| CFL2 |  |
| CD44 |  |
| ARPC1A |  |
|  |  |
| ***PAK Signaling*** | 7.2631 |
| MAP2K4 |  |
| MYL9 (includes EG:10398) |  |
| CFL1 |  |
| CFL2 |  |
| PDGFRA |  |
|  |  |
| ***Axonal Guidance Signaling*** | 7.733 |
| ACTR2 |  |
| CFL1 |  |
| CXCR4 |  |
| SEMA6A |  |
| PFN2 |  |
| NFATC4 |  |
| FZD1 |  |
| PLXND1 |  |
| MYL9 (includes EG:10398) |  |
| WIPF1 |  |
| CFL2 |  |
| PRKACA |  |
| ARPC1A |  |
| SEMA3F |  |
| PPP3CA |  |
|  |  |
| **CELL SURVIVAL** |  |
| ***Glutathione Metabolism*** | 10.098 |
| GSTM1 |  |
| GSTP1 |  |
| MGST2 |  |
| GCLC |  |
| GSTT2 |  |
|  |  |
| ***Induction of Apoptosis by HIV1*** | 9.1635 |
| MAP2K4 |  |
| CXCR4 |  |
| TNFRSF1A |  |
| TNFRSF11B |  |
|  |  |
| ***TNFR1 Signaling*** | 6.6355 |
| MAP2K4 |  |
| TNFRSF1A |  |
| CASP2 |  |
|  |  |
| ***Xenobiotic Metabolism Signaling*** | 4.2312 |
| MAP2K4 |  |
| CHST2 |  |
| GSTM1 |  |
| GSTP1 |  |
| MGST2 |  |
| GCLC |  |
| GSTT2 |  |
| AHR |  |
| CYP1B1 |  |
| CAMK2G |  |
|  |  |
| **LIPID METABOLISM** |  |
| ***Ceramide Signaling*** | 5.198 |
| MAP2K4 |  |
| TNFRSF1A |  |
| SPHK1 |  |
| TNFRSF11B |  |
|  |  |
| ***LXR/RXR Activation*** | 10.9228 |
| CCL2 |  |
| CCL7 |  |
| TNFRSF1A |  |
| CD14 |  |
| TNFRSF11B |  |
|  |  |
| ***LPS/IL-1 Mediated Inhibition of RXR Function*** | 10.318 |
| MAP2K4 |  |
| CHST2 |  |
| GSTM1 |  |
| GSTP1 |  |
| MGST2 |  |
| TNFRSF1A |  |
| CPT2 |  |
| CD14 |  |
| GSTT2 |  |
| TNFRSF11B |  |
|  |  |
|  |  |
| **TWO "unique"** | **Score** |
|  |  |
| **ENERGY REGULATION** |  |
| ***Glycolysis/Gluconeogenesis*** | 20.831 |
| PGK1 |  |
| AKR1A1 |  |
| GAPDH (includes EG:2597) |  |
| PFKP |  |
| PFKL |  |
| TPI1 |  |
| GALM |  |
| LDHA |  |
|  |  |
| ***Galactose Metabolism*** | 6.6468 |
| AKR1A1 |  |
| UGP2 |  |
| PFKP |  |
| PFKL |  |
|  |  |
| ***Pentose Phosphate Pathway*** | 5.4594 |
| PFKP |  |
| PFKL |  |
| RPIA |  |
|  |  |
| ***Fructose and Mannose Metabolism*** | 4.9956 |
| AKR1A1 |  |
| PFKP |  |
| PFKL |  |
| TPI1 |  |
|  |  |
| ***Starch and Sucrose Metabolism*** | 2.337 |
| UGP2 |  |
| PYGL |  |
| ENPP2 |  |
| SLC3A2 |  |
|  |  |
| ***PPAR Signaling*** | 5.1816 |
| PDGFA |  |
| HSP90AA1 |  |
| CITED2 |  |
| TNFRSF11B |  |
|  |  |
| **NEUROTRANSMISSION** |  |
| ***Taurine and Hypotaurine Metabolism*** | 6.9012 |
| CSAD |  |
| CDO1 |  |
|  |  |
| ***GABA Receptor Signaling*** | 15.8486 |
| UBB |  |
| GABBR2 |  |
| UBQLN1 |  |
| GABARAP |  |
|  |  |
| **RECEPTOR SIGNALING** |  |
|  |  |
| ***RAN Signaling*** | 13.572 |
| KPNA1 |  |
| IPO5 |  |
|  |  |
| ***TGF-β Signaling*** | 6.8926 |
| UBB |  |
| TGFB3 |  |
| BMP7 |  |
| SERPINE1 |  |
|  |  |
| **NUCLEOTIDE METABOLISM** |  |
| ***Cell Cycle: G1/S Checkpoint Regulation*** | 6 |
| MYC |  |
| TGFB3 |  |
| GSK3B |  |
|  |  |
| ***Purine Metabolism*** | 2.378 |
|  |  |
| MPP6 |  |
| ATP5C1 |  |
| GMPS |  |
| ATP5B |  |
| ADK |  |
| SMARCA5 |  |
| AK3L1 |  |
| ENPP2 |  |
| PSMC3 |  |
|  |  |
|  |  |
| **THREE "unique"** | **Score** |
|  |  |
| **IMMUNE REGULATION** |  |
| ***IL-8 Signaling*** | 10.013 |
| VCAM1 |  |
| ICAM1 |  |
| RND3 |  |
| PIK3C3 |  |
| PTGS2 |  |
| CSTB |  |
|  |  |
| ***CD40 Signaling*** | 9.5872 |
| ICAM1 |  |
| NFKBIA |  |
| PIK3C3 |  |
|  |  |
| ***IL-17 Signaling*** | 7.776 |
| CXCL10 |  |
| PIK3C3 |  |
| PTGS2 |  |
|  |  |
| ***Dendritic Cell Maturation*** | 6.456 |
| ICAM1 |  |
| NFKBIA |  |
| PIK3C3 |  |
| ATF4 |  |
| COL18A1 |  |
|  |  |
| ***Role of RIG1-like Receptors in Antiviral Innate Immunity*** | 5.6056 |
| NFKBIA |  |
| IRF3 |  |
|  |  |
| ***Fcγ Receptor-mediated Phagocytosis in Macrophages and Monocytes*** | 4.8708 |
| PLA2G6 |  |
| ACTB |  |
| ACTG1 |  |
|  |  |
| ***MIF Regulation of Innate Immunity*** | 17.8648 |
| PLA2G6 |  |
| NFKBIA |  |
| PTGS2 |  |
|  |  |
| ***Role of MAPK Signaling in the Pathogenesis of Influenza*** | 15.4037 |
| CXCL10 |  |
| LAMB2 |  |
| PLA2G6 |  |
| PTGS2 |  |
|  |  |
| ***Role of PI3K/AKT Signaling in the Pathogenesis of Influenza*** | 7.9395 |
| NFKBIA |  |
| PIK3C3 |  |
| IRF3 |  |
|  |  |
| ***Mechanisms of Viral Exit from Host Cells*** | 7.1435 |
| ACTB |  |
| ACTG1 |  |
|  |  |
| ***LPS-stimulated MAPK Signaling*** | 2.8672 |
| NFKBIA |  |
| PIK3C3 |  |
|  |  |
| **CANCER SIGNALING** |  |
| ***Chronic Myeloid Leukemia Signaling*** | 4.6332 |
| PIK3C3 |  |
| CTBP2 |  |
| MDM2 |  |
|  |  |
| ***Glioblastoma Multiforme Signaling*** | 2.116 |
| RND3 |  |
| PIK3C3 |  |
| MDM2 |  |
|  |  |
| ***Colorectal Cancer Metastasis Signaling*** | 1.8212 |
| RND3 |  |
| PIK3C3 |  |
| DVL1 |  |
| PTGS2 |  |
|  |  |
| ***Molecular Mechanisms of Cancer*** | 1.5544 |
| NFKBIA |  |
| RND3 |  |
| PIK3C3 |  |
| DVL1 |  |
| MDM2 |  |
|  |  |
| ***Estrogen-Dependent Breast Cancer Signaling*** | 3.5178 |
| PIK3C3 |  |
| ATF4 |  |
|  |  |
| ***Pancreatic Adenocarcinoma Signaling*** | 3.9109 |
| PIK3C3 |  |
| MDM2 |  |
| PTGS2 |  |
|  |  |
| ***Small Cell Lung Cancer Signaling*** | 6.5378 |
| NFKBIA |  |
| PIK3C3 |  |
| PTGS2 |  |
|  |  |
| **Melanoma Signaling** | 6.5685 |
| PIK3C3 |  |
| MDM2 |  |
|  |  |
| ***Prostate Cancer Signaling*** | 11.3424 |
| NFKBIA |  |
| PIK3C3 |  |
| ATF4 |  |
| MDM2 |  |
|  |  |
| **CELLULAR SIGNALING** |  |
| ***Arachidonic Acid Metabolism*** | 1.5678 |
| LAMB2 |  |
| PLA2G6 |  |
| PTGS2 |  |
|  |  |
| ***Eicosanoid Signaling*** | 7.4366 |
| LAMB2 |  |
| PLA2G6 |  |
| PTGS2 |  |
|  |  |
| ***Caveolar-mediated Endocytosis Signaling*** | 6.6717 |
| ACTB |  |
| FLOT1 |  |
| ACTG1 |  |
|  |  |
| ***p53 Signaling*** | 5.4442 |
| PLAGL1 |  |
| PIK3C3 |  |
| MDM2 |  |
|  |  |
| ***FAK Signaling*** | 5.16 |
| PIK3C3 |  |
| ACTB |  |
| ACTG1 |  |
|  |  |
| ***p38 MAPK Signaling*** | 4.9608 |
| PLA2G6 |  |
| DDIT3 |  |
| ATF4 |  |
|  |  |
| ***Fatty Acid Elongation in Mitochondria*** | 10.0344 |
| PPT1 |  |
| ECH1 |  |
|  |  |
| **CIRCADIAN/REPRODUCTION** |  |
| ***Germ Cell-Sertoli Cell Junction Signaling*** | 4.4781 |
| RND3 |  |
| PIK3C3 |  |
| ACTB |  |
| ACTG1 |  |
|  |  |
| ***Circadian Rhythm Signaling*** | 9.9925 |
| ATF4 |  |
| CRY1 |  |
|  |  |
| **RECEPTOR SIGNALING** |  |
| ***Glucocorticoid Receptor Signaling*** | 4.708 |
| TAF9 |  |
| VCAM1 |  |
| ICAM1 |  |
| NFKBIA |  |
| PIK3C3 |  |
| POLR2I |  |
|  |  |
| ***Endothelin-1 Signaling*** | 3.4776 |
| LAMB2 |  |
| PLA2G6 |  |
| PIK3C3 |  |
| PTGS2 |  |
|  |  |
| ***Angiopoietin Signaling*** | 3.132 |
| NFKBIA |  |
| PIK3C3 |  |
|  |  |
| ***Erythropoietin Signaling*** | 3.0508 |
| NFKBIA |  |
| PIK3C3 |  |
|  |  |
| ***Neurotrophin/TRK Signaling*** | 3.0438 |
| PIK3C3 |  |
| ATF4 |  |
|  |  |
| ***Estrogen Receptor Signaling*** | 3.683 |
| TAF9 |  |
| CTBP2 |  |
| POLR2I |  |
|  |  |
| ***Activation of IRF by Cytosolic Pattern Recognition Receptors*** | 3.1784 |
| NFKBIA |  |
| IRF3 |  |
|  |  |
| ***Lymphotoxin β Receptor Signaling*** | 11.1684 |
| VCAM1 |  |
| NFKBIA |  |
| PIK3C3 |  |
|  |  |
|  |  |
| **FOUR "unique"** | **Score** |
|  |  |
| **IMMUNE REGULATION** |  |
| ***Role of NFAT in Regulation of the Immune Response*** | 1.92 |
| CALM3 |  |
| JUN |  |
| CALM1 |  |
|  |  |
| ***Regulation of IL-2 Expression in Activated and Anergic T Lymphocytes*** | 7.1595 |
| CALM3 |  |
| JUN |  |
| CALM1 |  |
|  |  |
| ***T Cell Receptor Signaling*** | 5.46 |
| CALM3 |  |
| JUN |  |
| CALM1 |  |
|  |  |
| ***Production of Nitric Oxide and Reactive Oxygen Species in Macrophages*** | 4.7304 |
| JUN |  |
| RHOA |  |
| MAP3K4 |  |
| PPP1R14B |  |
|  |  |
| ***Nur77 Signaling in T Lymphocytes*** | 4.7112 |
| CALM3 |  |
| CALM1 |  |
|  |  |
| ***CD27 Signaling in Lymphocytes*** | 5.5809 |
| JUN |  |
| MAP3K4 |  |
|  |  |
| ***Calcium-induced T Lymphocyte Apoptosis*** | 4.0898 |
| CALM3 |  |
| CALM1 |  |
|  |  |
| ***CD28 Signaling in T Helper Cells*** | 3.9044 |
| CALM3 |  |
| JUN |  |
| CALM1 |  |
|  |  |
| ***IL-1 Signaling*** | 2.1546 |
| JUN |  |
| PRKAR2B |  |
|  |  |
| ***CCR5 Signaling in Macrophages*** | 7.5905 |
| CALM3 |  |
| JUN |  |
| CALM1 |  |
|  |  |
| **CARDIOVASCULAR SIGNALING** |  |
| ***Cardiac Hypertrophy Signaling*** | 7.7558 |
| CALM3 |  |
| JUN |  |
| PRKAR2B |  |
| RHOA |  |
| MAP3K4 |  |
| CALM1 |  |
|  |  |
| ***Nitric Oxide Signaling in the Cardiovascular System*** | 6.8289 |
| CALM3 |  |
| PRKAR2B |  |
| CALM1 |  |
|  |  |
| ***α-Adrenergic Signaling*** | 5.7732 |
| CALM3 |  |
| PRKAR2B |  |
| CALM1 |  |
|  |  |
| ***CCR3 Signaling in Eosinophils*** | 4.5 |
| CALM3 |  |
| RHOA |  |
| CALM1 |  |
|  |  |
| ***Role of NFAT in Cardiac Hypertrophy*** | 1.798 |
| CALM3 |  |
| PRKAR2B |  |
| CALM1 |  |
|  |  |
| **HEMOSTASIS** |  |
| ***Complement System*** | 10.6752 |
| CFB |  |
| CFH |  |
|  |  |
| ***Coagulation System*** | 10.0085 |
| PROS1 |  |
| TFP1 |  |
|  |  |
| **BMP-RELATED SIGNALING** |  |
| ***Keratan Sulfate Biosynthesis*** | 5.9943 |
| B4GALT4 |  |
| B4GALT6 |  |
|  |  |
| ***RANK Signaling in Osteoclasts*** | 12.7185 |
| CALM3 |  |
| JUN |  |
| MAP3K4 |  |
| CALM1 |  |
|  |  |
| ***BMP signaling pathway*** | 3.275 |
| JUN |  |
| PRKAR2B |  |
|  |  |
| **RECEPTOR SIGNALING** |  |
| ***Dopamine Receptor Signaling*** | 2.7735 |
| PRKAR2B |  |
| PPP1R14B |  |
|  |  |
| ***Melatonin Signaling*** | 9.087 |
| CALM3 |  |
| PRKAR2B |  |
| CALM1 |  |
|  |  |
| ***cAMP-mediated Signaling*** | 2.5854 |
| CALM3 |  |
| PRKAR2B |  |
| CALM1 |  |
|  |  |
| ***CREB Signaling in Neurons*** | 2.0196 |
| CALM3 |  |
| PRKAR2B |  |
| CALM1 |  |
|  |  |
| ***Glutamate Receptor Signaling*** | 4.2614 |
| CALM3 |  |
| CALM1 |  |
|  |  |
| ***Synaptic Long Term Potentiation*** | 9.8766 |
| CALM3 |  |
| PRKAR2B |  |
| PPP1R14B |  |
| CALM1 |  |
|  |  |
| ***GNRH Signaling*** | 3.4528 |
| JUN |  |
| PRKAR2B |  |
| MAP3K4 |  |
|  |  |
| ***CXCR4 Signaling*** | 2.556 |
| JUN |  |
| CXCL12 |  |
| RHOA |  |
|  |  |
| ***CDK5 Signaling*** | 2.4921 |
| PRKAR2B |  |
| PPP1R114B |  |
|  |  |
| ***SAPK/JNK Signaling*** | 2.3028 |
| JUN |  |
| MAP3K4 |  |
|  |  |
| ***IGF-1 Signaling*** | 2.26 |
| JUN |  |
| PRKAR2B |  |
|  |  |
| ***Androgen Signaling*** | 7.3948 |
| CALM3 |  |
| JUN |  |
| PRKAR2B |  |
| CALM1 |  |
|  |  |
| ***Corticotropin Releasing Hormone Signaling*** | 7.8498 |
| CALM3 |  |
| JUN |  |
| PRKAR2B |  |
| CALM1 |  |
|  |  |
| ***PXR/RXR Activation*** | 2.926 |
| PRKAR2B |  |
| ABCB9 |  |
|  |  |
| **CANCER SIGNALING** |  |
| ***Breast Cancer Regulation by Stathmin1*** | 5.7814 |
| CALM3 |  |
| PRKAR2B |  |
| RHOA |  |
| RB1CC1 |  |
| PPP1R14B |  |
| CALM1 |  |
