# Supplementary material for: Multiple Oxygen Tension Environments Reveal Diverse Patterns of Transcriptional Regulation in Primary Astrocytes
Source: PLoS One. 2011 Jun 27;6(6):e21638. doi: 10.1371/journal.pone.0021638 (PMC3124552; doi:10.1371/journal.pone.0021638)
Supplement: Table S15 — Highest scoring gene networks present within group clusters ONE , TWO , THREE and FOUR . For each group cluster geneset, functional interaction networks were created using Ingenuity Pathway Analysis (IPA). Network scores are created by the structural strength of the network in addition to the number of focus genes (genes present in network also present in the input experimental dataset: focus genes are indicated in bold). The IPA-predicted primary functions of the network are indicated for each of the four group clusters of genes. (DOC) [file pone.0021638.s021.doc]

**Table S15. Highest scoring gene networks present within group clusters *ONE*, *TWO,* *THREE* and *FOUR*.** For each group cluster geneset, functional interaction networks were created using Ingenuity Pathway Analysis (IPA). Network scores are created by the structural strength of the network in addition to the number of focus genes (genes present in network also present in the input experimental dataset: focus genes are indicated in bold). The IPA-repdicted primary functions of the network are indicated for each of the four group clusters of genes.

| ***ONE*** |  |
| --- | --- |
|  |  |
| **Predicted Functions** |  |
| *Cellular Assembly and Organization* |  |
| *Cellular Function and Maintenance* |  |
| *Protein Synthesis* |  |
|  |  |
| **Score** | **38** |
| **Number of Focus Genes** | **21** |
| Alpha Actinin |  |
| **CCL2** |  |
| **CD44** |  |
| **COL3A1** |  |
| **COL6A3** |  |
| collagen |  |
| Collagen type I |  |
| Collagen type IV |  |
| Collagen(s) |  |
| **CST3** |  |
| **CTSH** |  |
| **CTSL1** |  |
| ERK1/2 |  |
| **FGFR3** |  |
| Focal adhesion kinase |  |
| Integrin |  |
| **ITGA7** |  |
| **ITGB1BP1** |  |
| Laminin |  |
| **LGALS1** |  |
| **LGALS3BP** |  |
| Mek |  |
| Mmp |  |
| Pdgf |  |
| PDGF BB |  |
| Pdgfra-Pdgfrb |  |
| **PPAP2B** |  |
| **PPIC** |  |
| **SEMA3F** |  |
| **SPHK1** |  |
| **SSX2IP** |  |
| **TCEB2** |  |
| **TIMP1** |  |
| **TNFRSF11B** |  |
| **TNK2** |  |
|  |  |
|  |  |
| ***TWO*** |  |
|  |  |
| **Predicted Functions** |  |
| *Cellular Development* |  |
| *Protein Synthesis* |  |
| *Carbohydrate Metabolism* |  |
|  |  |
| **Score** | **57** |
| **Focus Genes** | **31** |
| **ADK** |  |
| Alpha tubulin |  |
| **CAPZA2** |  |
| **CAPZB** |  |
| **COX17** |  |
| **COX6A1** |  |
| **CSDA** |  |
| Cytochrome c oxidase |  |
| **EGLN1** |  |
| **FBLN1** |  |
| **GABARAP** |  |
| **GABARAPL2 (includes EG:11345)** |  |
| **GDI2** |  |
| HISTONE |  |
| **IPO5** |  |
| **LXN** |  |
| **MAPRE3** |  |
| **MCL1** |  |
| **MTPN** |  |
| **MYC** |  |
| **NDEL1** |  |
| **PCM1** |  |
| PFK |  |
| **PFKL** |  |
| **PFKP** |  |
| **PGK1** |  |
| **RPL13** |  |
| **RPL15** |  |
| **RPL41** |  |
| **RPL36 (includes EG:25873)** |  |
| **RPS13** |  |
| **RPS18** |  |
| **RPS12 (includes EG:6206)** |  |
| **RPS4X** |  |
| **SIRT2** |  |
|  |  |
|  |  |
| ***THREE*** |  |
|  |  |
| **Predicted Functions** |  |
| *Metabolic Disease* |  |
| *Renal and Urological Disease* |  |
| *Cell Death* |  |
|  |  |
| **Score** | **43** |
| **Focus Genes** | **21** |
| **ACTB** |  |
| **ACTG1** |  |
| Actin |  |
| **COL18A1** |  |
| Collagen type I |  |
| Collagen type IV |  |
| Collagen(s) |  |
| **COTL1** |  |
| **CTBP2** |  |
| **CTGF** |  |
| **DDIT3** |  |
| **DMD** |  |
| **DVL1** |  |
| ERK1/2 |  |
| F Actin |  |
| Insulin |  |
| **LAMB2** |  |
| Mapk |  |
| Pdgf |  |
| PDGF BB |  |
| PI3K |  |
| **PIK3C3** |  |
| Pkc(s) |  |
| PLA2G6 |  |
| Pld |  |
| Ras homolog |  |
| **RPL8** |  |
| **RPL32** |  |
| **RPS10** |  |
| **RPS14** |  |
| **RPSA** |  |
| **TAF9** |  |
| **TAGLN** |  |
| **TRIO** |  |
| **VIM** |  |
|  |  |
| ***FOUR*** |  |
|  |  |
| **Predicted Functions** |  |
| *Endocrine System Disorders* |  |
| *Cellular Assembly and Organization* |  |
| *Metabolic Disease* |  |
|  |  |
| **Score** | **51** |
| **Focus Genes** | **23** |
| Ap1 |  |
| **CFB** |  |
| **CXCL12** |  |
| **CXCR7** |  |
| **DCN** |  |
| **ENO1** |  |
| Estrogen Receptor |  |
| **FKBP5** |  |
| **GBP2** |  |
| **GORASP2** |  |
| **HES1** |  |
| **HN1** |  |
| **ID1** |  |
| Insulin |  |
| **JUN** |  |
| **KPNA2** |  |
| Mmp |  |
| NFkB (complex) |  |
| **PARP1** |  |
| Pi3-kinase |  |
| **POSTN** |  |
| **PROS1** |  |
| **RECK** |  |
| RNA polymerase II |  |
| Sapk |  |
| **SFPQ** |  |
| **SNRPA** |  |
| T3-TR-RXR |  |
| **TFPI** |  |
| Tgf beta |  |
| **THRSP** |  |
| Ubiquitin |  |
| **UCP2** |  |
| Vegf |  |
| **ZIC2** |  |
