# Supplementary material for: Multiple Oxygen Tension Environments Reveal Diverse Patterns of Transcriptional Regulation in Primary Astrocytes
Source: PLoS One. 2011 Jun 27;6(6):e21638. doi: 10.1371/journal.pone.0021638 (PMC3124552; doi:10.1371/journal.pone.0021638)
Supplement: Table S16 — Number of implicitly correlated genes, from group clusters ONE, TWO, THREE and FOUR , associated with each LSI interrogation term. The number of genes from each group cluster dataset that demonstrated at least an implicit correlation (score ≥0.1) to the specific LSI interrogation term 1–9 is represented. (DOC) [file pone.0021638.s022.doc]

**Table S16. Number of implicitly correlated genes, from group clusters ONe to FOUR, associated with each LSI interrogation term.** The number of genes from each group cluster dataset that demonstrated at least an implicit correlation (score ≥0.1) to the specific LSI interrogation term 1-9 is represented.

|  | **LSI interrogation** | **ONE** | **TWO** | **THREE** | **FOUR** |
| --- | --- | --- | --- | --- | --- |
|  |  |  |  |  |  |
| **1** | Neurodegeneration | 15 | 29 | 7 | 1 |
| **2** | Alzheimer's | 7 | 16 | 4 | 3 |
| **3** | Aging | 22 | 16 | 3 | 4 |
| **4** | Ischemia | 31 | 23 | 8 | 4 |
| **5** | Neuroprotection | 8 | 6 | 1 | 1 |
| **6** | Cognition | 1 | 4 | 0 | 0 |
| **7** | Hyperoxia | 8 | 4 | 0 | 0 |
| **8** | Hypoxia | 17 | 25 | 11 | 3 |
| **9** | Astrocytes | 10 | 10 | 2 | 1 |
