# Supplementary material for: Multiple Oxygen Tension Environments Reveal Diverse Patterns of Transcriptional Regulation in Primary Astrocytes
Source: PLoS One. 2011 Jun 27;6(6):e21638. doi: 10.1371/journal.pone.0021638 (PMC3124552; doi:10.1371/journal.pone.0021638)
Supplement: Table S17 — Mean Latent Semantic Indexing correlation scores for group clusters ONE , TWO , THREE and FOUR . The mean LSI correlation score of all the genes that demonstrated an individual implicit correlation (score ≥0.1) to the specific LSI interrogation term 1–9. (DOC) [file pone.0021638.s023.doc]

**Table S17. Mean Latent Semantic Indexing correlation scores for group clusters ONE, TWO, THREE and FOUR**. The mean LSI correlation score of all the genes that demonstrated an individual implicit correlation (score ≥0.1) to the specific LSI interrogation term 1-9.

|  | **LSI interrogation** | **ONE** | **TWO** | **THREE** | **FOUR** |
| --- | --- | --- | --- | --- | --- |
|  |  |  |  |  |  |
| **1** | Neurodegeneration | 0.144647 | 0.143862 | 0.140714 | 0.125 |
| **2** | Alzheimer's | 0.160571 | 0.212375 | 0.11425 | 0.151 |
| **3** | Aging | 0.129682 | 0.129 | 0.1915 | 0.1395 |
| **4** | Ischemia | 0.131387 | 0.148826 | 0.137 | 0.152 |
| **5** | Neuroprotection | 0.131387 | 0.119167 | 0.107 | 0.106 |
| **6** | Cognition | 0.148 | 0.166 | 0 | 0 |
| **7** | Hyperoxia | 0.118875 | 0.1075 | 0 | 0 |
| **8** | Hypoxia | 0.147529 | 0.1844 | 0.143636 | 0.115333 |
| **9** | Astrocytes | 0.1293 | 0.1743 | 0.137 | 0.105 |
